# Ligand-assisted palladium-catalyzed C–H alkenylation of aliphatic amines for the synthesis of functionalized pyrrolidines†
†Electronic supplementary information (ESI) available: Experimental procedures, characterization data and kinetic details. CCDC 1449203. For ESI and crystallographic data in CIF or other electronic format see DOI: 10.1039/c7sc00468k


**DOI:** 10.1039/c7sc00468k

**Published:** 2017-02-23

**Authors:** Chuan He, Matthew J. Gaunt

**Affiliations:** a Department of Chemistry , University of Cambridge , Lensfield Road , Cambridge , CB2 1EW , UK . Email: mjg32@cam.ac.uk ; http://www-gaunt.ch.cam.ac.uk

## Abstract

The development of a ligand-assisted Pd-catalyzed C–H alkenylation of aliphatic amines is reported. Our studies indicated that an amino-acid-derived ligand renders the C–H bond activation step reversible and promotes the traditionally difficult alkenylation process. The C(sp^3^)–H alkenylation proceeds through a 5-membered-ring cyclopalladation pathway that allows access to complex aliphatic heterocycles that could be useful to practioners of synthetic and medicinal chemistry.

## Abstract 2

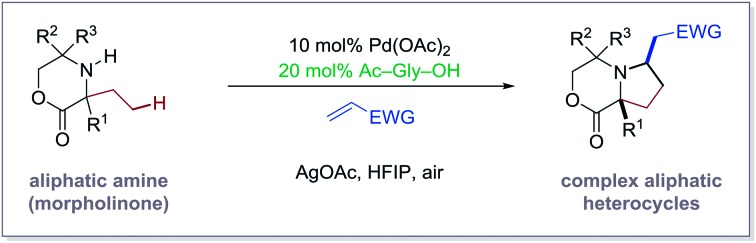
The development of a ligand-assisted Pd-catalyzed C–H alkenylation of aliphatic amines is reported.

## Introduction

The synthesis of architecturally complex aliphatic amines remains an important challenge to synthetic and medicinal chemists because their structural and functional properties are often fundamental to biological activity in many nitrogen-containing molecules.^1^ Recently, palladium-catalyzed C–H functionalization of aliphatic amine derivatives has emerged as a potentially powerful tactic for the synthesis of complex variants of these important molecules.^2^ Central to the continued evolution of these synthetic strategies is the development of new activation modes and transformations on a range of aliphatic amine substrates.

**Fig. 1:**
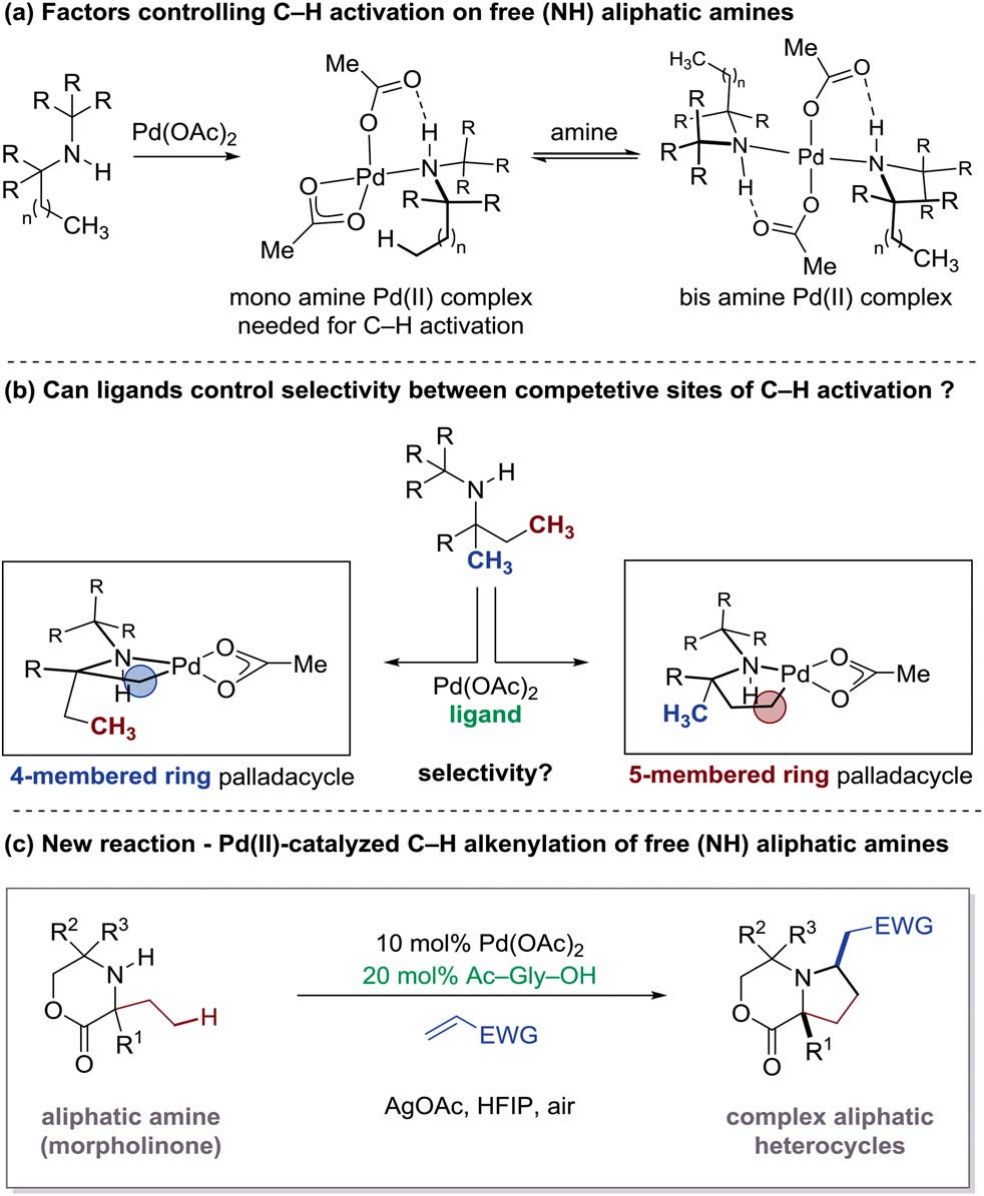
Evolution of a ligand-controlled C–H alkenylation.

Over the last three years, our laboratory has been engaged in the development of C(sp^3^)–H activation reactions guided by the free (NH)-amine.^3^ Central to the success of this strategy has been the steric-induced destabilization of rapidly formed bis-amine Pd(ii) complexes, which leads to higher concentrations of the putative mono-amine Pd(ii) complexes empirically required for C–H bond cleavage. Furthermore, a crucial hydrogen bond between the NH group of the ligated amine and the carbonyl group of the acetate ligand orients the amine such that C–H activation is facilitated (Fig. 1a).3*b* While we have developed a number of distinct transformation based on discrete 4- and 5-membered cyclopalladation pathways, we began to question whether we could control the selectivity of the C–H activation by the action of an external ligand, which could lead to the development of new transformations on aliphatic amine scaffolds (Fig. 1b). Herein, we describe how an amino acid-derived ligand was found to strongly influence the C–H activation on aliphatic amines with competitive sites of reactivity. By rendering the cyclopalladation step reversible, the amino acid ligand facilitates a productive 5-membered ring cyclopalladation pathway, leading to a new C–H alkenylation reaction to generate complex pyrrolidine-based heterocycles (Fig. 1c). The C–H catalytic alkenylation is operationally simple and works well on a range of substituted morpholinone-derived amines and with a variety of electron-deficient alkenes. Furthermore, the process provides access to previously unexplored aliphatic heterocycles that we believe will be of interest as novel amine scaffolds for drug discovery.

## Ligand-controlled reversible C–H activation

The basis for our studies was an observation made during our original work on Pd-catalyzed C–H amination to form aziridines.3*a* The signature reaction of this work exploited a 4-membered ring cyclopalladation pathway on a C–H bond at the β-position to the free (NH) amine motif. However, we were surprised to discover that this unusual cyclopalladation mode predominated even when traditionally more favourable C–H activation pathways were accessible; β-C–H activation *via* a 4-membered ring intermediate was favoured over a more classical 5-membered ring cyclopalladation at a competitive γ-C–H bond (Fig. 3a). Using this scaffold as a starting point for our studies, we elected to investigate this unusual selectivity in the context of a C–H alkenylation reaction.

**Fig. 2:**
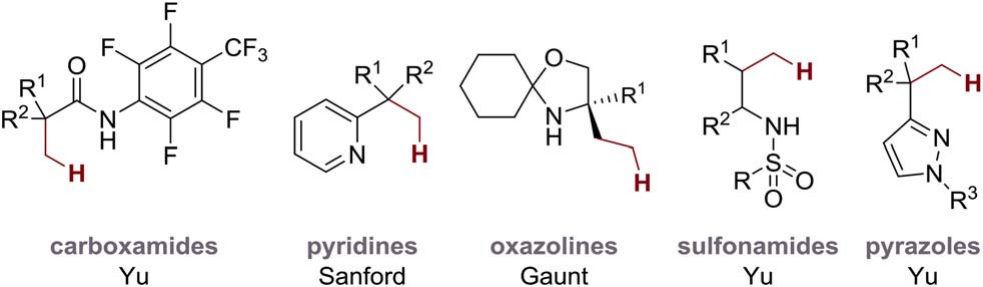
Aliphatic systems capable of C–H alkenylation.

The successful realization of Pd-catalyzed C–H alkenylation reactions has great potential for the synthesis of complex molecules.^4^ Since the seminal works of Fujiwara and Moritani,^5^ many different approaches to C–H alkenylation have been developed, in particular on aromatic substrates.^6^ Central to many of these developments have been the exploitation of Lewis basic directing groups that facilitate the C–H activation step.^7^ Furthermore, the role of activating ligands has extended the scope of available C–H alkenylation reactions to a variety of substrate classes.6i,^8^ In contrast, C–H alkenylation in aliphatic systems remains limited to a small number of examples (Fig. 2).3d,^9^ To date, successful examples of Pd-catalyzed C(sp^3^)–H alkenylation directly using alkenes include Yu's carboxamide-directed olefination to γ-lactams,9*a* Sanford's pyridine-directed olefination to pyridinium salts,9*b* our group's free (NH) amine-directed olefination of amino alcohols derivatives,3*d* and Yu's sulfonamide-directed olefination9*c* and pyrazole-directed olefination.9*d* As a result, the development of new methods for Pd-catalyzed C–H alkenylation remains an ongoing and important goal.

**Fig. 3:**
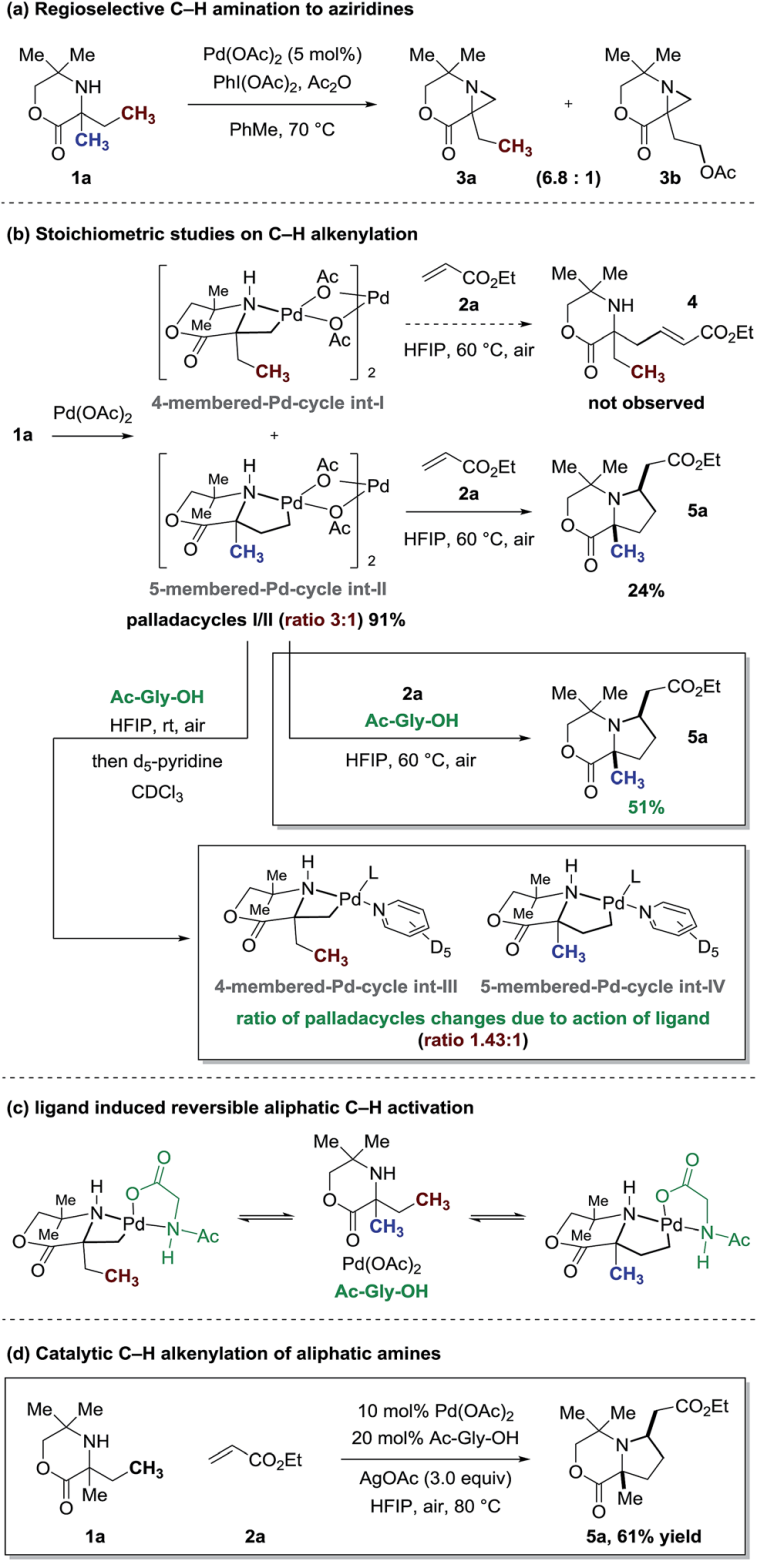
Identification of a ligand controlled reversible C–H activation.

Our studies began with the investigation of a stoichiometric C–H alkenylation process using differentially substituted morpholinone **1a**. Treatment of **1a** with 1.5 equivalents of Pd(OAc)_2_ delivered a 3 : 1 mixture of palladacycles (**int-I** and **int-II**) in favour of the 4-membered ring complex (Fig. 3b), as expected based on the outcome of the reaction shown in Fig. 3a. When this mixture of palladacycles was treated with acrylate **2a** in hexafluoroisopropanol (HFIP) at 60 °C, we did not observe the formation of the expected alkenylation product **4**, but found that the bicyclic pyrrolidine product **5a** was formed in 24% yield; reaction of only the 5-membered ring palladacycle *via* sequential carbopalladation of the alkene, β-hydride elimination to a substituted acrylate and aza-Michael addition gave the pyrrolidine-derived heterocycle **5a**.

Previous work in our group had highlighted an essential role of amino-acid ligands, first introduced by Yu *et al.*,^10^ in securing an effective C–H arylation process on the tetramethylpiperidine scaffold.3*c* Therefore, we treated the mixture of palladacycles formed from morpholinone **1a** with acrylate **2a** in the presence of two equivalents of glycine-derived ligand (Ac-Gly-OH, **6a**, see ESI† for details) and found that the yield of the pyrrolidine product **5a** increased to 51%. This result is surprising because, based on the ratio of observed palladacycles, the maximum theoretical yield for this product should be 25%. Therefore, the increased yield of **5a** suggested that the amino acid ligand could be reversing the preferred cyclopalladation process to the 4-membered complex and channeling the reaction down the 5-membered ring C–H activation pathway to form **5a** (Fig. 3b).

To further investigate this phenomena, we treated the 3 : 1 mixture of palladacycles **int-I/II** with ligand Ac-Gly-OH **6a** at room temperature, but without the acrylate. Then, we quenched the reaction with d_5_-pyridine, which enables directly identification of the palladacycles by ^1^H NMR, and found that the ratio of complexes (4-membered **int-III***vs.* 5-membered **int-IV**) had changed from 3 : 1 to 1.43 : 1 (Fig. 3b). This result strongly suggests that the ligand **6a** is reversing the cyclopalladation process, such that, in the presence of an alkene, the C–H alkenylation *via* the 5-membered ring palladacycle becomes a kinetic trap for the equilibrium, selectively forming **5a**. To the best of our knowledge, this represents the first example of an amino acid-derived ligand controlling the regioselectivity of C–H activation on aliphatic systems (Fig. 3c). We were able to render this specific transformation catalytic in Pd(OAc)_2_ by the addition of AgOAc as terminal oxidant, which led to the formation of pyrrolidine **5a** in 61% yield (Fig. 3d).

## Scope of ligand-assisted C–H alkenylation

While morpholinone **1a** had provided an effective scaffold to probe the ligand controlled selectivity between competitive cyclopalladation pathways, variation of substitution at the carbon atom adjacent to the NH motif would be essential in exploring the scope of the process. To test this, we prepared a range of morpholinones, α,α′-morpholinones displaying the ethyl group needed for (5-membered ring) C–H alkenylation alongside a variety of functionalized substituents (Table 1). First, we selected the α,α-diethyl substituted morpholinone **1b** and found that the conditions identified for the successful reaction of **1a** were effective for this substrate; reaction involving treatment of the amine **1b** with ethyl acrylate **2a** with 10 mol% Pd(OAc)_2_, 20 mol% of ligand Ac-Gly-OH **6a**, AgOAc and with HFIP as the solvent delivered the desired pyrrolidine product **5b** in 86% yield, after isolation by silica gel chromatography. In further assessing the scope of the catalytic reaction, we found that a range of functionalized α,α′-disubstituted morpholinones also underwent smooth ligand assisted C–H alkenylation. Protected hydroxymethyl substituents worked well in 48–78% yields, providing readily manipulable functional groups suitable for down stream modification of varying substitution patterns (**5d–5f**). Longer alkyl chains with remote functional groups, including esters, protected amines, sulfones even an alkyl boronic ester, proved to be excellent substrates for the C–H alkenylation giving the complex pyrrolidines in good yields (**5g–5j**). Changing the substituents on the non-reacting side of the morpholinone substrate to a cyclohexyl motif gave 75% yield (**5k**). Importantly, reducing steric hindrance around the nitrogen atom could also produce the corresponding alkenylation products. By using the chiral morpholinone substrates (**1l** and **1m**), which could be easily assembled from chiral pool amino alcohols, synthetically useful levels of diastereoselective transformation could be achieved to afford the enantiopure pyrrolidine products (**5l** and **5m**). When cyclopropyl substituted morpholinone was subjected to the same reaction conditions, methylene C–H alkenylation took place to form **5n** as a single isomer, albeit in a lower yield. When an aromatic group was present close to the NH motif, the reaction unsurprisingly proceeded through the inherently more facile aryl C–H activation pathway to give exclusively *ortho* sp^2^ C–H alkenylation to **5o** in excellent yields.

**Table 1:** Scope of aliphatic amines^*a*^

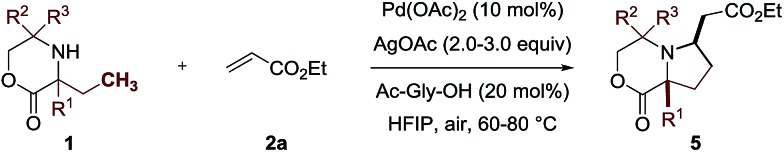
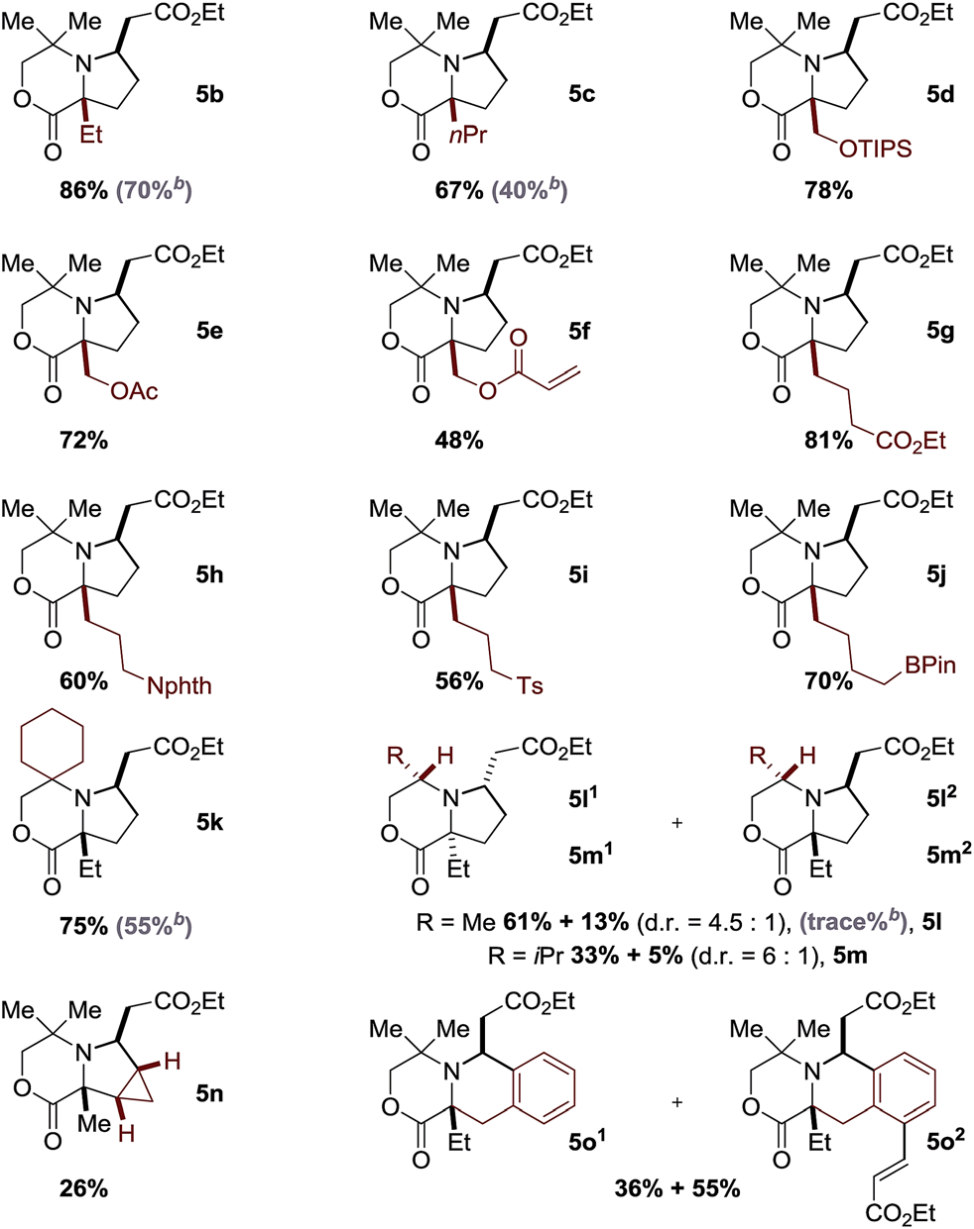

^*a*^Yield of isolated product. See ESI for details. TIPS = triisopropylsilyl, Nphth = phthalimide, Ts = *p*-toluenesulfonyl, BPin = boronic acid pinacol ester.

^*b*^Without ligand Ac-Gly-OH **6a**.

It was interesting to note that even though the reaction worked in some cases in the absence of the amino acid-derived ligand, the yield was significantly increased in its presence. For example, the yield of **5c** was increased by almost 30% in the presence of Ac-Gly-OH **6a**. More strikingly, C–H alkenylation of the chiral morpholinone **1l** to **5l** did not proceed in the absence of the ligand **6a**. Unfortunately, acyclic amine substrates were unsuccessful when tested under the optimal conditions.^11^

We next explored the range of alkenes that could be transferred as part of this process (Table 2). The reaction was readily extended to a variety of acrylates in excellent yields (**5p–5t**). α,β-Unsaturated ketones, amides and even acrolein could also be incorporated into the pyrrolidine scaffold (**5u–5y**). Moreover, vinyl sulfone and vinyl phosphonate also worked well in the C–H alkenylation process, providing useful products (**5z** and **5aa**) with opportunities for further elaboration *via* well-established methods. Again, we found that ligand Ac-Gly-OH **6a** was not necessary for a successful reaction with acrylate coupling partners, but it did have a significant effect on the yield of the reaction with alkene coupling partners such as enones (**5v**), acrylamides (**5x**), vinyl sulfones (**5z**) and vinyl phosphonates (**5aa**). Unfortunately, simple unfunctionalized alkenes or styrenes were not suitable for this transformation under the current conditions either with or without the use of amino acid ligand.

**Table 2:** Scope of alkenes^*a*^

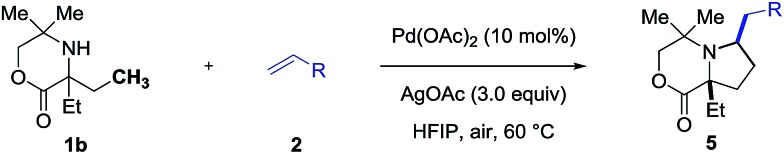
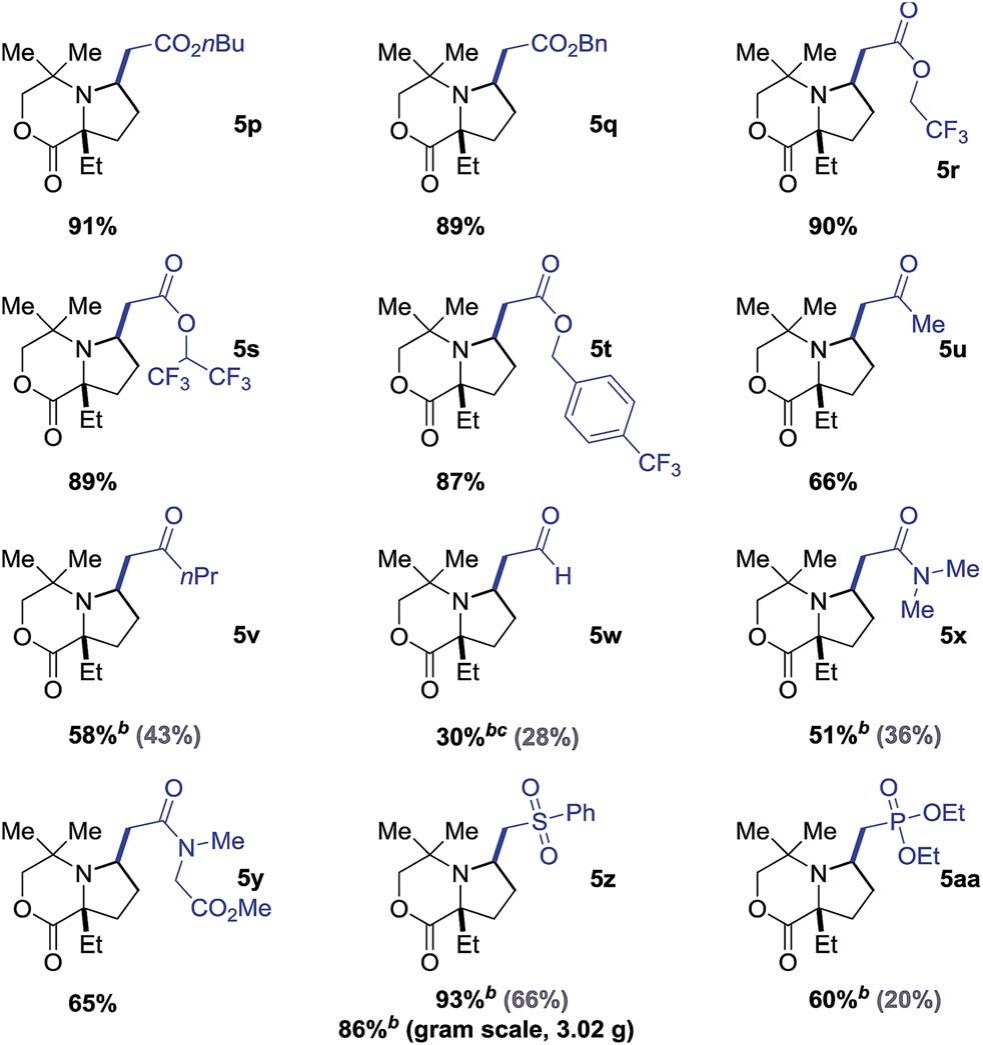

^*a*^Yield of isolated product.

^*b*^With 20 mol% Ac-Gly-OH **6a**.

^*c*^Reaction at room temperature. See ESI for details.

**Scheme 1:**
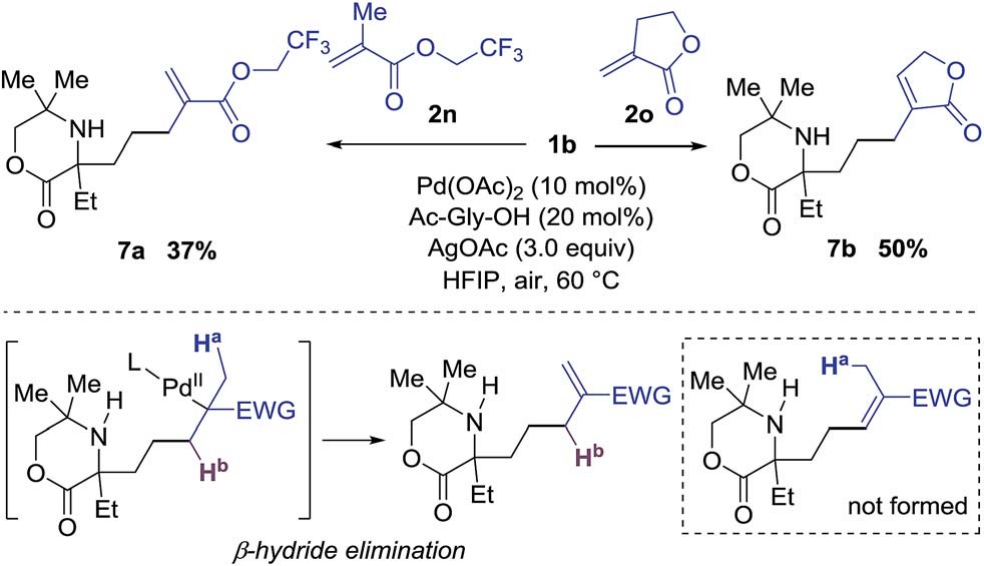
Reaction of di-substituted acrylates.

Interestingly, when di-substituted acrylates **2n** and **2o** were employed in the reaction, the alkenyl products **7a** and **7b** were obtained in moderate yields with no trace of the corresponding aza-Michael products (Scheme 1). These results infer that the β-hydride elimination step takes place in a different sense to the reaction with the simple acrylates shown in Table 2, and that aza-Michael addition to the corresponding azocene is presumably disfavored in comparison to the stability of the alkene product.

## Preliminary mechanistic investigations

Although the amino-acid-derived ligand Ac-Gly-OH **6a** is not essential in this C–H alkenylation reaction, its presence significantly improves the conversion and yield of product for some examples (**5b**, **5c**, **5k**, **5l**, **5v**, **5x**, **5z**, and **5aa**), which leads to an expanded substrate scope. Given the impact of ligand **6a** on the outcome of the catalytic C–H alkenylation reaction and its ability to enable reversible aliphatic C–H bond activation (Fig. 3), we conducted preliminary experiments towards better understanding the mechanism of the new reaction.

**Scheme 2:**
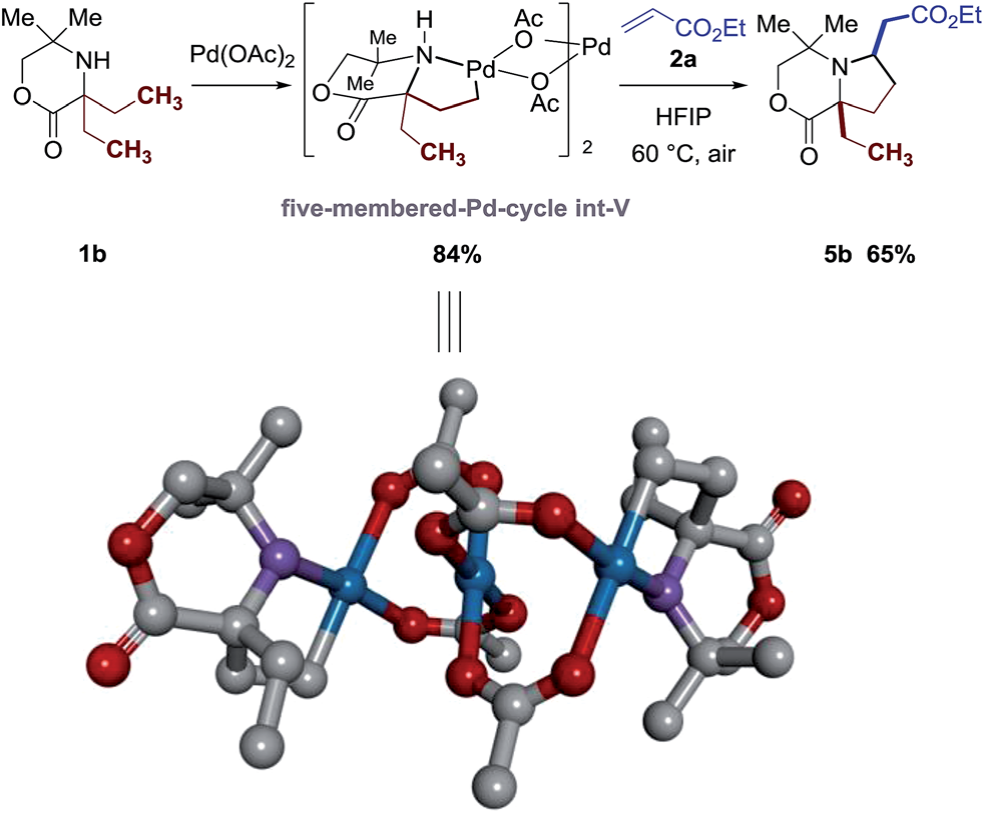
X-ray crystal structure of the amine-palladacycle **int-V** (hydrogens are removed for clarity) and its stoichiometric reaction with acrylate **2a**.

Firstly, we confirmed that the reaction proceeds through an amine-directed C–H bond activation step. Following the procedure in Fig. 3, treatment of morpholinone **1b** with 1.5 equivalents of Pd(OAc)_2_ delivered the anticipated cyclopalladation complex **int-V** after stirring at 60 °C with CHCl_3_ as solvent. The structure of this palladacycle **int-V** was identified by analysis of the X-ray diffraction pattern of a single crystal, confirming that it was the amine motif that was directing the C–H bond cleavage rather than the lactone function. Further reaction with ethyl acrylate **2a** from this palladacycle **int-V** provided the corresponding pyrrolidine **3b** in 65% yield (Scheme 2).

**Fig. 4:**
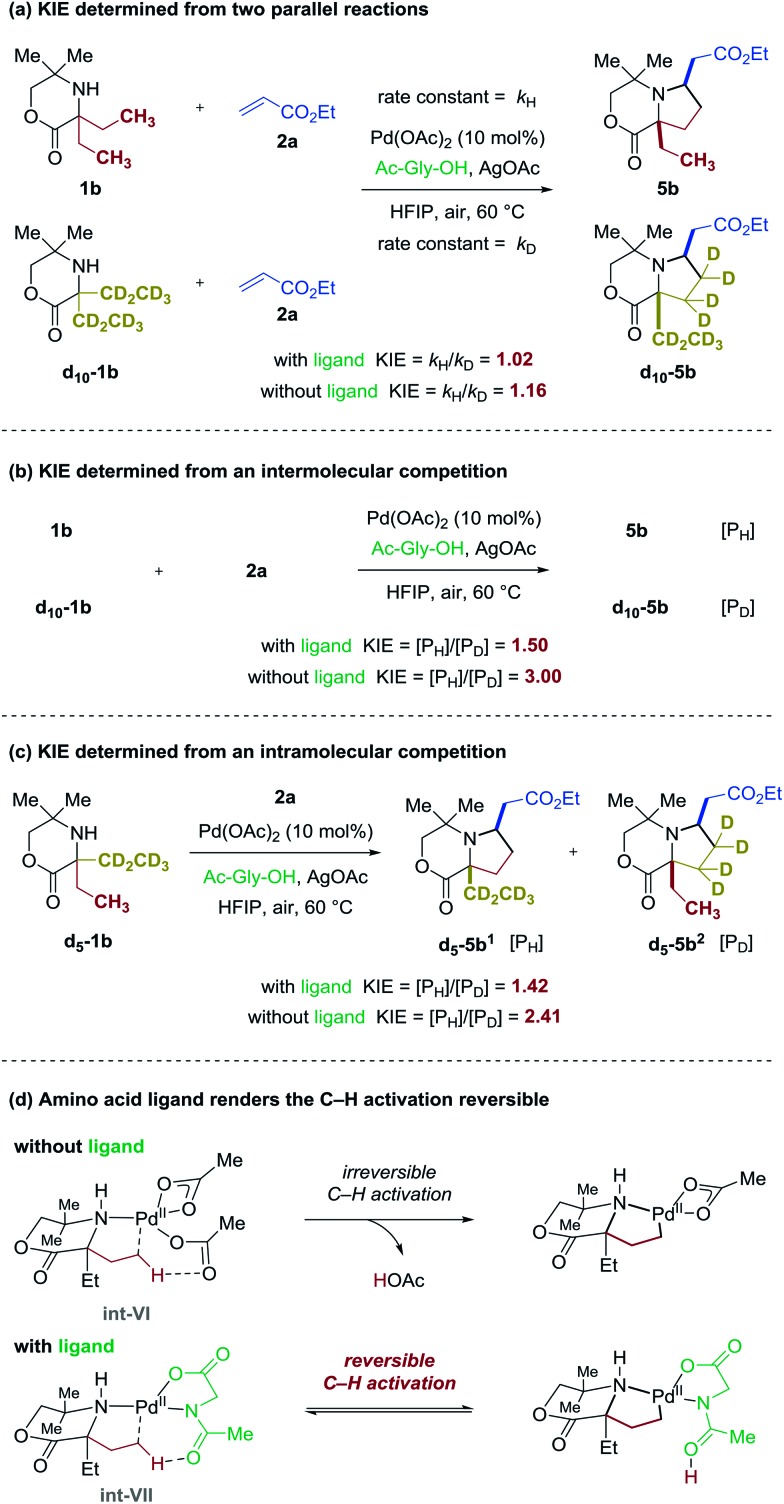
KIE studies of the catalytic C–H alkenylation.

We next investigated some of the basic kinetic parameters of the reaction. Three types of kinetic isotope effect (KIE) experiments were explored with and without the ligand under catalytic conditions.^12^ First, two parallel reactions were carried out to measure the rate constants, one with **1b** fully protiated at the reactive ethyl positions and one with **d_10_-1b** fully deuterated (Fig. 4a). In the presence of ligand Ac-Gly-OH **6a**, the reaction gave rise to a *k*_H_/*k*_D_ value of 1.02. The lack of a kinetic isotope effect rules out C–H bond cleavage occurring during the turnover-limiting step. Meanwhile, a small isotope effect *k*_H_/*k*_D_ = 1.16 was observed in the absence of ligand, which indicated that C–H bond cleavage is not the turnover-limiting step either. Second, same-flask intermolecular competitive reaction of **1b** and **d_10_-1b** was investigated (Fig. 4b). In the absence of ligand, a larger primary isotope effect was observed ([*P*_H_]/[*P*_D_] = 3.00); while in the presence of ligand, a smaller potential isotope effect was observed ([*P*_H_]/[*P*_D_] = 1.50). Third, reaction of **d_5_-1b** containing one protiated ethyl group and one deuterated ethyl group with **2a** was also investigated (Fig. 4c). Similar results were obtained that in the absence of ligand, a larger primary isotope effect was observed ([*P*_H_]/[*P*_D_] = 2.41); while in the presence of ligand, a smaller potential isotope effect was observed ([*P*_H_]/[*P*_D_] = 1.42). These KIE studies indicate that the C–H bond cleavage step is irreversible without ligand, and occurs after a rate-determining step (possibly the dissociation of the bis-amine Pd(ii) complex to the active mono-amine Pd(ii) complex **int-VI**); the loss of AcOH from the Pd(ii) complex during the C–H activation step (Fig. 4d) is likely to be the reason why the C–H cleavage step is irreversible. In the presence of ligand, however, the process could begin with amino acid coordination to the Pd(ii) catalyst followed by association of one molecule of amine substrate to form **int-VII**. The *N*-Ac group serves the role of inner-sphere base need for the C–H bond cleavage step.^13^ The reversible nature of this step could be explained by the retention of the ligated protonated amide in proxinity to the carbon–Pd bond, facilitating re-protonation (Fig. 4d, see ESI Fig. S2† for a detailed mechanism with and without ligand). Taken together, these results are consistent with the observations of the selective C–H alkenylation of **1a** and stoichiometric studies highlighted in Fig. 3.

## Derivatization of the C–H alkenylation products

**Fig. 5:**
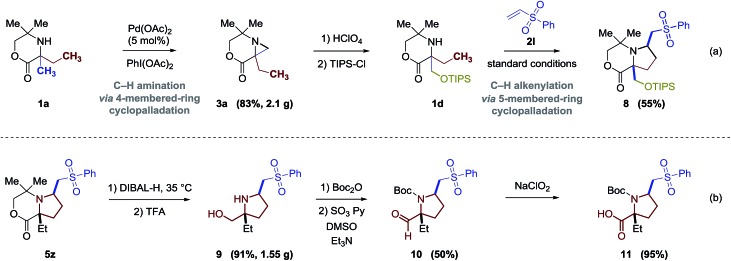
Synthetic applications.

Finally, we sought to explore the scaffold elaboration of the products from this new C–H alkenylation process. To demonstrate the efficacy of sequential C–H bond functionalization strategies, we showed that **1b** can be selectively functionalized using three step process involving two C–H activation steps. Firstly, catalytic C–H amination through a 4-membered-ring cyclopalladation pathway forms the aziridine **3a**; secondly, ring opening of the aziridine provides a functionalized secondary amine **1d**;3*a* and finally, C–H alkenylation through a 5-membered-ring cyclopalladation pathway gives the highly functionalized pyrrolidine derivative **8** (Fig. 5a). To further demonstrate the synthetic versatility of these products, the bicyclic aliphatic heterocycles can be transferred to a series of highly functionalized α-quaternary proline derivatives (Fig. 5b). Reducing the lactone **5z** with DIBAL-H followed by the treatment with trifluoroacetic acid (TFA) cleaves the cyclic framework and affords the prolinol product **9** in 91% yield. Further oxidations under appropriate conditions provide the corresponding aldehyde **10** and amino acid proline derivative **11** in good yields.

## Conclusions

In summary, we have developed a palladium-catalyzed C–H alkenylation of aliphatic amines. This alkenylation reaction proceeds through a 5-membered-ring cyclopalladation pathway that allows alkene insertion and aza-Michael cyclization for the synthesis of various pyrrolidine moieties in good yields with perfect regio- and diastereo-selectivity. The process transforms readily available aliphatic amines into highly functionalized complex aliphatic heterocycles that we believe will prove attractive to practioners of medicinal chemistry as novel scaffolds. We also found that an amino-acid-derived ligand changes the rate-limiting step and enables a reversible aliphatic C–H bond activation, which leads to an expanded substrate scope to be developed on this C–H bond alkenylation. Finally, some non-racemic amino acid-derived ligands were also assessed for the induction of enantioselectivity to this transformation (see ESI Fig. S3†). Although only 12% ee was obtained with Fmoc-(l)-Phe-OH, we believe it is a viable starting point for further development.

## Supplementary Material

Supplementary information

Crystal structure data
