# Supplementary material for: Ligand-assisted palladium-catalyzed C–H alkenylation of aliphatic amines for the synthesis of functionalized pyrrolidines
Source: Chem Sci. 2017 Feb 23;8(5):3586–92. doi: 10.1039/c7sc00468k (PMC6092717; doi:10.1039/c7sc00468k)
Supplement: Supplementary file 1 [file SC-008-C7SC00468K-s001.pdf]

Electronic Supplementary Material (ESI) for Chemical Science.
This journal is © The Royal Society of Chemistry 2017

Ligand-assisted palladium-catalyzed C–H alkenylation of aliphatic amines for the synthesis of functionalized pyrrolidines

Chuan He and Matthew J. Gaunt*

Department of Chemistry, University of Cambridge, Lensfield Road, Cambridge, United Kingdom. CB2 1EW

Supporting Information

1. General Information....................2
2. Starting Materials....................4
3. Palladium Complexes and X-Ray Crystallography....................27
4. C(sp^3)–H Alkenylation Reactions....................30
    4.1 General Procedure for the Stoichiometric C–H Alkenylation 30
    4.2 General Procedure for the Catalytic C–H Alkenylation....................31
    4.3 Catalytic Condition Optimization....................31
    4.4 Data....................32
    4.5 Unsuccessful Morpholinone Amine Substrates....................65
5. Derivatization of the Alkenylation Product....................65
6. Kinetic Isotope Effects....................70
7. Proposed Mechanism....................76
8. Asymmetric C−H Alkenylation Attempt....................77
9. References....................78
10. NMR Spectra 79

1. General Information

Proton nuclear magnetic resonance (^{1}H NMR) spectra were recorded at ambient temperature on a Bruker AM 400 (400 MHz) or an Avance 500 (500 MHz) spectrometer. Chemical shifts (δ) are reported in ppm and quoted to the nearest 0.01 ppm relative to the residual protons in $CDCl_3$ (7.26 ppm), $(CD_3)_2SO$ (2.50 ppm) or CD_3OD (3.31 ppm) and coupling constants (*J*) are quoted in Hertz (Hz). Data are reported as follows: Chemical shift (multiplicity, coupling constants, number of protons). Coupling constants were quoted to the nearest 0.1 Hz and multiplicity reported according to the following convention: s = singlet, d = doublet, t = triplet, q = quartet, qn = quintet, sp = septet, m = multiplet, br = broad. Where coincident coupling constants have been observed, the apparent (app) multiplicity of the proton resonance has been reported.

Carbon nuclear magnetic resonance (^{13}C NMR) spectra were recorded at ambient temperature on a Bruker AM 400 (100 MHz) or an Avance 500 (125 MHz) spectrometer. Chemical shift (δ) was measured in ppm and quoted to the nearest 0.1 ppm relative to the residual solvent peaks in $CDCl_3$ (77.16 ppm), $(CD_3)_2SO$ (39.52 ppm) or CD_3OD (49.00 ppm). DEPT135, nOe experiments and 2-dimensional experiments (COSY, HMBC and HSQC) were used to support assignments where appropriate.

High-resolution mass spectra (HRMS) were measured on a Micromass Q-TOF spectrometer using EI (electron impact) or ES (electrospray ionisation) techniques at the Department of Chemistry, University of Cambridge or at the EPSRC Mass Spectrometry Service at the University of Swansea. Infared (IR) spectra were recorded on a Perkin Elmer 1FT-IR Spectrometer fitted with an ATR sampling accessory as either solids or neat films, either through direct application or deposited in $CHCl_3$, with absorptions reported in wavenumbers (cm^{-1}). Melting points (m.p.) were recorded using a Gallenkamp melting-point apparatus and are reported uncorrected. X-ray crystallography was performed on a Nonius Kappa CCD at the Cambridge University Chemistry X-Ray Laboratory.

Analytical thin layer chromatography (TLC) was performed using pre-coated Merck glass backed silica gel plates (Silica gel 60 F254). Flash column chromatography was undertaken on Fluka or Material Harvest silica gel (230–400 mesh) under a positive pressure of air unless otherwise stated. Visualization was achieved using ultraviolet light (254 nm) and chemical staining with ceric ammonium molybdate or basic potassium permanganate solutions as appropriate. ISOLUTE® SCX-2 was purchased from Biotage, which is a propylsulfonic acid bonded sorbent used for the extraction of basic compounds samples using a strong cation exchange retention mechanism.

Tetrahydrofuran (THF), toluene, hexane, diethyl ether and dichloromethane were dried and distilled using standard methods.[1] 1,2-Dichloroethane (DCE), chloroform, *tert*-amyl alcohol, DMF, 2,2,2-Trifluoroethanol (TFE) and acetone were purchased from Acros or Sigma-Aldrich chemical company. All reagents were purchased at the highest commercial quality and used without further purification. Reactions were carried out under an atmosphere of air unless otherwise stated. All reactions were monitored by TLC, ^{1}H NMR spectra taken from reaction samples (NMR yields determined by ^{1}H NMR with reference to triphenylmethane as an internal standard) or gas chromatography (GC) and gas chromatography mass spectrometry (GC-MS) using a Shimadzu QP2010-SE fitted with a BPX5 column (10 m, 0.1 mm, 0.1 μm film) for FID analysis (triphenylmethane as an internal standard for GC yields) and a SHIM-5MS column (30 m, 0.25 mm, 0.25 μm film) for MS analysis. Chiral HPLC analysis was performed on HP Agilent 1100 or Shimadzu UFLC-XR 2020 apparatus using a Chiralpak AD-H column.

$Pd(OAc)_2$ (Pd 45.9-48.4%, needles) was purchased from Alfa Aesar, alkenes were purchased from Sigma-Aldrich and Alfa Aesar, AgOAc was purchased from Alfa Aesar, 1,1,1,3,3,3-Hexafluoropropan-2-ol (HFIP) (99%) was purchased from Apollo Scientific, Ac-Gly-OH was purchased from Sigma-Aldrich, which were all used without further purification.

2. Starting Materials

3-Ethyl-3,5,5-trimethylmorpholin-2-one (1a)

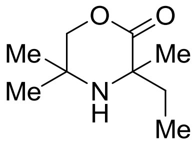

A 250 mL three-necked round bottom flask was charged with 2-amino-2-methyl-1-propanol (5.0 g, 56 mmol), $CHCl_3$ (20 mL) and butan-2-one (40 mL). The flask was equipped with a stir bar, and cooled to 0 ℃ using an ice bath. Powdered NaOH (10.0 g, 0.25 mol) was then added portion-wise at a rate that maintained the internal temperature below 5 ℃. After the addition was complete the reaction mixture was allowed to gradually warm to room temperature overnight. The resulting slurry was filtered on a frit, the white solid was washed with CH_2Cl_2 (100 mL) followed by methanol (200 mL) and the combined filtrates were concentrated *in vacuo* to provide the crude carboxylate. The amorphous solid was treated with a concentrated solution of hydrochloric acid (50 mL) and heated to reflux for 8 hours. The reaction mixture was cooled to room temperature and concentrated *in vacuo*. The residue was cooled to 0 ℃ and basified by the addition of a saturated aqueous solution of $NaHCO_3$. The aqueous solution was extracted with ethyl acetate (3 x 50 mL) and the combined organic extracts were dried ($MgSO_4$), filtered and concentrated *in vacuo*. The crude product was further purified by Kugelrohr distillation to provide the title compound as a colorless oil (6.5 g, 68% yield). IR ν_{max}/cm^{-1} (film): 2972, 1726, 1460, 1379, 1285, 1223, 1117, 1053; ^1H NMR (400 MHz, $CDCl_3$) δ: 4.08 (d, *J* = 10.8 Hz, 1H), 4.04 (d, *J* = 10.8 Hz, 1H), 1.73 (dq, *J* = 14.8, 7.4 Hz, 1H), 1.51 (dq, *J* = 14.8, 7.4 Hz, 1H), 1.33 (s, 3H), 1.18 (s, 3H), 1.09 (s, 3H), 0.89 (t, *J* = 7.4 Hz, 3H); ^{13}C NMR (100 MHz, $CDCl_3$) δ: 174.6, 77.9, 58.2, 48.7, 35.7, 29.1, 26.8, 25.9, 8.4; m/z HRMS (ESI) found $[M+H]^+$ 172.1328, $C_9H_{18}NO_2$ requires 172.1332. Data in agreement with reported procedure.[2]

3,3-Diethyl-5,5-dimethylmorpholin-2-one (1b)

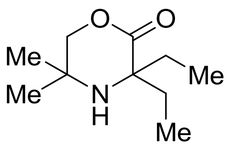

A 250 mL three-necked round bottom flask was charged with 2-amino-2-methyl-1-propanol (5.0 g, 56 mmol), $CHCl_3$ (20 mL) and pentan-3-one (40 mL). The flask was equipped with a stir bar, and cooled to 0 ℃ using an ice bath. Powdered NaOH (10.0 g, 0.25 mol) was then added portion-wise at a rate that maintained the internal temperature below 5 ℃. After the addition was complete the reaction mixture was allowed to gradually warm to room temperature overnight. The resulting slurry was filtered on a frit, the white solid was washed with CH_2Cl_2 (100 mL) followed by methanol (200 mL) and the combined filtrates were concentrated *in vacuo* to provide the crude carboxylate. The amorphous solid was treated with a concentrated solution of hydrochloric acid (50 mL) and heated to reflux for 8 hours. The reaction mixture was cooled to room temperature and concentrated *in vacuo*. The residue was cooled to 0 ℃ and basified by the addition of a saturated aqueous solution of $NaHCO_3$. The aqueous solution was extracted with ethyl acetate (3 x 50 mL) and the combined organic extracts were dried ($MgSO_4$), filtered and concentrated *in vacuo*. The crude product was further purified by Kugelrohr distillation to provide the title compound as a colorless oil (5.9 g, 56% yield). IR ν_{max}/cm^{-1} (film): 2968, 1729, 1461, 1379, 1286, 1217, 1131, 1055; ^1H NMR (400 MHz, $CDCl_3$) δ: 4.10 (s, 2H), 1.75 (dq, *J* = 14.8, 7.4 Hz, 2H), 1.64 (dq, *J* = 14.8, 7.4 Hz, 2H), 1.19 (s, 6H), 0.93 (t, *J* = 7.4 Hz, 6H); ^{13}C NMR (100 MHz, $CDCl_3$) δ: 174.1, 77.4, 61.3, 48.5, 32.9, 26.8, 8.2; m/z HRMS (ESI) found $[M+H]^+$ 186.1484, $C_{10}H_{20}NO_2$ requires 186.1489.

3-Ethyl-5,5-dimethyl-3-propylmorpholin-2-one (1c)

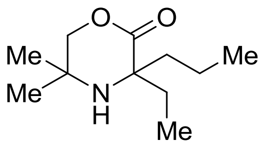

A 250 mL three-necked round bottom flask was charged with 2-amino-2-methyl-1-propanol (2.2 g, 25 mmol), $CHCl_3$ (10 mL) and hexan-3-one (25

mL). The flask was equipped with a stir bar, and cooled to 0 ℃ using an ice bath. Powdered NaOH (5.0 g, 0.125 mol) was then added portion-wise at a rate that maintained the internal temperature below 5 ℃. After the addition was complete the reaction mixture was allowed to gradually warm to room temperature overnight. The resulting slurry was filtered on a frit, the white solid was washed with CH_2Cl_2 (50 mL) followed by methanol (100 mL) and the combined filtrates were concentrated *in vacuo* to provide the crude carboxylate. The amorphous solid was treated with a concentrated solution of hydrochloric acid (30 mL) and heated to reflux for 8 hours. The reaction mixture was cooled to room temperature and concentrated *in vacuo*. The residue was cooled to 0 ℃ and basified by the addition of a saturated aqueous solution of $NaHCO_3$. The aqueous solution was extracted with ethyl acetate (3 x 30 mL) and the combined organic extracts were dried ($MgSO_4$), filtered and concentrated *in vacuo*. The crude product was further purified by Kugelrohr distillation to provide the title compound as a colorless oil (0.6 g, 12% yield). IR ν_{max}/cm^{-1} (film): 2967, 1728, 1463, 1379, 1284, 1213, 1131, 1056; ^{1}H NMR (400 MHz, $CDCl_3$) δ: 4.08 (app s, 2H), 1.79-1.50 (m, 4H), 1.40-1.29 (m, 2H), 1.16 (s, 6H), 0.94-0.86 (m, 6H); ^{13}C NMR (125 MHz, $CDCl_3$) δ: 174.2, 77.4, 61.2, 48.7, 42.6, 33.4, 26.8, 26.7, 17.2, 14.4, 8.3; m/z HRMS (ESI) found $[M+H]^+$ 200.1641, $C_{11}H_{22}NO_2$ requires 200.1645.

6-Ethyl-2,2-dimethyl-4-oxa-1-azabicyclo[4.1.0]heptan-5-one (3a)

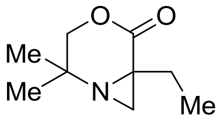

An oven dried 250 mL round bottom flask equipped with stir bar was charged with 3-ethyl-3,5,5-trimethylmorpholin-2-one (2.56 g, 15 mmol), $Pd(OAc)_2$ (168 mg, 0.75 mmol) and iodobenzene diacetate (7.25 g, 22.5 mmol). Then 150 mL toluene was added followed by acetic anhydride (2.83 mL, 30 mmol) and the round bottom flask capped with a glass stopper. The vessel was placed in a pre-heated oil bath at 70 ℃ and stirred for 12 hours. The reaction was then cooled to room temperature, filtered through Celite, eluting with ethyl acetate (100 mL), and concentrated *in vacuo*. The residue was purified by flash column chromatography (70/30 40-60 ℃ Petroleum

Ether/EtOAc) to provide the title compound as a yellow solid (2.1 g, 83% yield). m.p. 42-44 ºC; IR νmax/cm^{-1} (film): 2971, 1720, 1498, 1461, 1382, 1326, 1284, 1258, 1136, 1068, 1043; ^{1}H NMR (400 MHz, $CDCl_3$) δ: 4.00 (d, *J* = 12.2 Hz, 1H), 3.85 (d, *J* = 12.2 Hz, 1H), 2.44 (s, 1H), 2.29 (dq, *J* = 13.8, 7.0 Hz, 1H), 1.94 (s, 1H), 1.30 (s, 3H), 1.19-1.08 (m, 4H), 1.04 (t, *J* = 7.1 Hz, 3H); ^{13}C NMR (100 MHz, $CDCl_3$) δ: 169.9, 71.5, 49.6, 40.9, 32.6, 27.9, 25.8, 24.0, 10.4. Data in agreement with reported procedure.[2]

3-Ethyl-3-(hydroxymethyl)-5,5-dimethylmorpholin-2-one

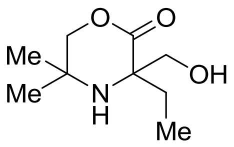

6-Ethyl-2,2-dimethyl-4-oxa-1-azabicyclo[4.1.0]heptan-5-one (0.5 g, 3.0 mmol) was refluxed in a 70% aqueous solution of perchloric acid (10 mL) under a nitrogen atmosphere and behind a blast shield for 16 hours. *[Caution: heating perchloric acid carries the risk of explosion]*. The reaction was cooled to room temperature, transferred to a conical flask and cooled to 0 °C before being carefully neutralised by the addition of solid $NaHCO_3$ to the stirred mixture. The aqueous solution was extracted with CH_2Cl_2 (5 x 20 mL). The combined organics were dried (Na_2SO_4), filtered and concentrated *in vacuo* to provide the crude title compound as a yellow oil (0.45 g, 80% yield) which was used in the next steps without further purification. IR νmax/cm^{-1} (film): 3448, 2971, 1721, 1461, 1380, 1286, 1217, 1051; ^{1}H NMR (400 MHz, $CDCl_3$) δ: 4.11 (s, 2H), 3.58 (d, *J* = 10.9 Hz, 1H), 3.50 (d, *J* = 10.9 Hz, 1H), 2.56 (br, 1H), 1.89-1.61 (m, 2H), 1.19 (s, 6H), 0.93 (t, *J* = 7.5 Hz, 3H); ^{13}C NMR (100 MHz, $CDCl_3$) δ: 174.1, 77.7, 67.1, 61.8, 48.6, 30.8, 26.5, 25.7, 8.2; m/z HRMS (ESI) found $[M+H]^+$ 188.1279, $C_9H_{18}NO_3$ requires 188.1281.

3-Ethyl-5,5-dimethyl-3-((triisopropylsilyloxy)methyl)morpholin-2-one (1d)

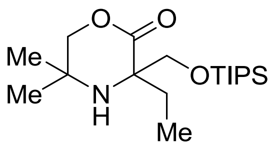

A 10 mL round bottom flask equipped with stir bar was charged with the crude 3-ethyl-3-(hydroxymethyl)-5,5-dimethylmorpholin-2-one (130 mg, 0.7 mmol), imidazole (95.2 mg, 1.4 mmol), triisopropylsilyl chloride (270 mg, 1.4 mmol) and DMF (2 mL). Then the mixture was stirred at room temperature for 24 hours. The reaction was diluted with water (3 mL) and extracted with EtOAc (3 x 10 mL). The combined organic extracts were washed with brine, dried ($MgSO_4$) and concentrated *in vacuo*. The crude material was filtered through SCX-2, washing with methanol and then eluting with ammonia methanol solution (7 M). The ammonia methanol solution filtrate was concentrated *in vacuo* to recover the amine compounds from the crude mixture, which was further purified by flash column chromatography (90/10 40-60 ℃ Petroleum Ether/EtOAc) to provide the title compound as a clear oil (190 mg, 79% yield). IR ν_{max}/cm^{-1} (film): 2942, 1736, 1463, 1222, 1098, 883, 796, 683; ^{1}H NMR (400 MHz, $CDCl_3$) δ: 4.16-4.02 (m, 2H), 3.93 (d, *J* = 9.3 Hz, 1H), 3.62 (d, *J* = 9.3 Hz, 1H), 1.79-1.62 (m, 2H), 1.20 (s, 3H), 1.19 (s, 3H), 1.14-1.02 (m, 21H), 0.93 (t, *J* = 7.5 Hz, 3H); ^{13}C NMR (100 MHz, $CDCl_3$) δ: 173.4, 77.8, 69.2, 63.0, 48.3, 31.8, 26.7, 26.3, 18.10, 18.08, 12.0, 8.5; m/z HRMS (ESI) found $[M+H]^+$ 344.2616, $C_{18}H_{38}NO_3Si$ requires 344.2615.

(3-Ethyl-5,5-dimethyl-2-oxomorpholin-3-yl)methyl acetate (1e)

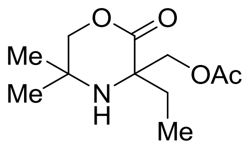

A 10 mL round bottom flask equipped with stir bar was charged with the crude 3-ethyl-3-(hydroxymethyl)-5,5-dimethylmorpholin-2-one (130 mg, 0.7 mmol), triethylamine (212 mg, 2.1 mmol), acetyl chloride (110 mg, 1.4 mmol) and CH_2Cl_2 (2 mL). Then the mixture was stirred at room temperature for 24 hours. The reaction was diluted with water (3 mL) and extracted with CH_2Cl_2 (3 x 10 mL). The combined

organic extracts were washed with brine, dried ($MgSO_4$) and concentrated *in vacuo*. The crude material was purified by flash column chromatography (60/40 40-60 ℃ Petroleum Ether/EtOAc) to provide the title compound as a colorless oil (136 mg, 85% yield). IR ν_{max}/cm^{-1} (film): 2975, 1733, 1461, 1379, 1221, 1134, 1050; ^1H NMR (400 MHz, $CDCl_3$) δ: 4.20 (d, *J* = 11.0 Hz, 1H), 4.15-4.06 (m, 3H), 2.05 (s, 3H), 1.85-1.73 (m, 1H), 1.71-1.61 (m, 1H), 1.20 (s, 3H), 1.17 (s, 3H), 0.95 (t, *J* = 7.5 Hz, 3H); ^{13}C NMR (100 MHz, $CDCl_3$) δ: 171.8, 170.8, 77.5, 69.4, 61.0, 48.7, 32.0, 26.8, 26.4, 21.0, 8.2; m/z HRMS (ESI) found $[M+H]^+$ 230.1387, $C_{11}H_{20}NO_4$ requires 230.1387.

(3-Ethyl-5,5-dimethyl-2-oxomorpholin-3-yl)methyl acrylate (1f)

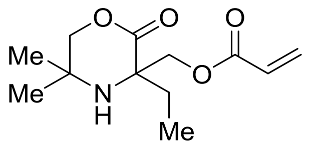

A 10 mL round bottom flask equipped with stir bar was charged with the crude 3-ethyl-3-(hydroxymethyl)-5,5-dimethylmorpholin-2-one (93.5 mg, 0.5 mmol), triethylamine (151 mg, 1.5 mmol), acryloyl chloride (90.5 mg, 1.0 mmol) and CH_2Cl_2 (2 mL). Then the mixture was stirred at room temperature for 24 hours. The reaction was diluted with water (2 mL) and extracted with CH_2Cl_2 (3 x 10 mL). The combined organic extracts were washed with brine, dried ($MgSO_4$) and concentrated *in vacuo*. The crude material was purified by flash column chromatography (60/40 40-60 ℃ Petroleum Ether/EtOAc) to provide the title compound as a yellow oil (75 mg, 62% yield). IR ν_{max}/cm^{-1} (film): 2974, 1726, 1652, 1406, 1183, 1056, 980, 794; ^1H NMR (400 MHz, $CDCl_3$) δ: 6.43 (dd, *J* = 17.3, 1.1 Hz, 1H), 6.13 (dd, *J* = 17.3, 10.4 Hz, 1H), 5.87 (dd, *J* = 10.4, 1.1 Hz, 1H), 4.28 (q, *J* = 11.1 Hz, 2H), 4.13 (q, *J* = 10.8 Hz, 2H), 1.84 (dq, *J* = 15.0, 7.5 Hz, 1H), 1.70 (dq, *J* = 14.7, 7.5 Hz, 1H), 1.21 (s, 3H), 1.19 (s, 3H), 0.99 (t, *J* = 7.5 Hz, 3H); ^{13}C NMR (100 MHz, $CDCl_3$) δ: 171.8, 166.0, 131.7, 128.1, 77.5, 69.5, 61.2, 48.7, 32.1, 26.9, 26.6, 8.3; m/z HRMS (ESI) found $[M+H]^+$ 242.1388, $C_{12}H_{20}NO_4$ requires 242.1387.

4-Benzyl-5,5-dimethylmorpholin-2-one

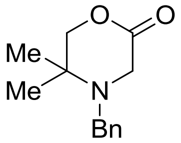

To a solution of 2-amino-2-methylpropan-1-ol (5.4 g, 60.7 mmol) in toluene (130 mL) was added benzaldehyde (8.0 mL, 79.2 mmol). The flask was fitted with a Dean-Stark trap, and the solution was heated at 150 ℃ for 2 hours. The solution was then cooled to room temperature and concentrated *in vacuo*. To a 0 ℃ solution of the residue in EtOH (130 mL) was added $NaBH_4$ (8.0 g, 211.5 mmol) and sufficient 4 N HCl in dioxane to adjust the pH to ca. 2. The mixture was stirred overnight and then concentrated *in vacuo*. The residue was partitioned between 1 N aq. HCl (200 mL) and CH_2Cl_2 (100 mL). The aqueous phase was washed with a fresh portion of CH_2Cl_2 (100 mL), and then adjusted to pH > 13 with 6 N aq. NaOH. The aqueous phase was extracted with CH_2Cl_2 (2 × 150 mL), and the combined organic phase was dried (Na_2SO_4), filtered, and concentrated *in vacuo* to provide 2-(benzylamino)-2-methylpropan-1-ol as a colorless oil (10.7 g, 98% yield).

2-(Benzylamino)-2-methylpropan-1-ol (10.7 g, 59.7 mmol) was dissolved in toluene (80 mL) followed by the addition of *N*-ethyldiisopropylamine (15 mL) and methyl bromoacetate (5.0 mL, 66.2 mmol). The mixture was heated to 50 ℃ overnight. The mixture was allowed to cool to room temperature, diluted with water (100 mL) and extracted with CH_2Cl_2 (3 × 100 mL), dried ($MgSO_4$), filtered and concentrated *in vacuo*. The crude material was purified by flash column chromatography (80/20 40-60 ℃ Petroleum Ether/EtOAc) to provide the title compound as a white solid (10.8 g, 82% yield). m.p. 66-68 ºC; IR ν_{max}/cm^{-1} (film): 2972, 1744, 1384, 1292, 1056, 724, 698; ^{1}H NMR (400 MHz, $CDCl_3$) δ: 7.38-7.19 (m, 5H), 4.10 (s, 2H), 3.56 (s, 2H), 3.25 (s, 2H), 1.21 (s, 6H); ^{13}C NMR (100 MHz, $CDCl_3$) δ: 168.6, 137.8, 128.6, 127.6, 78.7, 53.7, 51.5, 50.4, 19.1; m/z HRMS (ESI) found $[M+H]^+$ 220.1331, $C_{13}H_{18}NO_2$ requires 220.1332.

4-Benzyl-3-ethyl-5,5-dimethylmorpholin-2-one

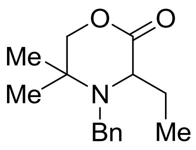

A solution of 4-benzyl-5,5-dimethylmorpholin-2-one (1.31 g, 6.0 mmol) and iodoethane (1.87 g, 12.0 mmol) in THF (30.0 mL) and *N[1]*,*N[1]*,*N[2]*,*N[2]*-tetramethylethane-1,2-diamine (TMEDA) (3.0 mL) was cooled to –78 ℃ and treated with sodium bis(trimethylsilyl)amide (3.0 mL, 2 M, 6.0 mmol) drop wise over 5 min and stirred for 1 h. After warming to room temperature and stirring for an additional 16 hours, the reaction mixture was diluted with EtOAc (30 mL), washed with saturated ammonium chloride solution (15 mL) and brine (2 x 15 mL). The organic phase was dried ($MgSO_4$), filtered, and concentrated *in vacuo*. The crude material was purified by flash column chromatography (85/15 40-60 ℃ Petroleum Ether/EtOAc) to provide the title compound as a white solid (1.05 g, 71% yield). m.p. 70-72 ºC; IR ν_{max}/cm^{-1} (film): 2970, 1736, 1454, 1383, 1224, 1063, 721, 698; ^1H NMR (400 MHz, $CDCl_3$) δ: 7.39-7.16 (m, 5H), 4.24 (d, *J* = 10.7 Hz, 1H), 4.10 (d, *J* = 15.5 Hz, 1H), 3.93 (d, *J* = 10.7 Hz, 1H), 3.51-3.38 (m, 1H), 3.29 (d, *J* = 15.5 Hz, 1H), 1.78-1.59 (m, 1H), 1.38 (dt, *J* = 13.7, 6.8 Hz, 1H), 1.20 (s, 3H), 1.11 (s, 3H), 0.83 (t, *J* = 7.2 Hz, 3H); ^{13}C NMR (100 MHz, $CDCl_3$) δ: 172.1, 141.3, 128.4, 127.7, 127.0, 77.3, 65.2, 55.0, 52.9, 27.1, 24.9, 16.5, 9.2; m/z HRMS (ESI) found $[M+H]^+$ 248.1646, $C_{15}H_{22}NO_2$ requires 248.1645.

Ethyl 4-(3-ethyl-5,5-dimethyl-2-oxomorpholin-3-yl)butanoate (1g)

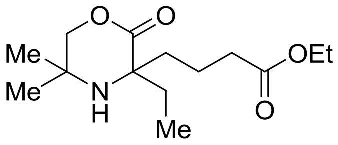

A solution of 4-benzyl-3-ethyl-5,5-dimethylmorpholin-2-one (1.2 g, 4.8 mmol) and allyl iodide (8.1 g, 48.0 mmol) in THF (40 mL) and TMEDA (5 mL) was cooled to –78 ℃ and treated with potassium bis(trimethylsilyl)amide (20 mL, 0.7 M, 14 mmol) drop wise over 10 min and stirred for 1 h. After warming to room temperature and stirring for an additional 16 hours, the reaction mixture was diluted with EtOAc,

washed with saturated ammonium chloride solution and brine. The organic phase was dried ($MgSO_4$), filtered, and concentrated *in vacuo*. The crude material was purified by flash column chromatography (90/10 40-60 ℃ Petroleum Ether/EtOAc) to provide 3-allyl-4-benzyl-3-ethyl-5,5-dimethylmorpholin-2-one as a colorless oil (952 mg, 69% yield).

In a 10 mL vial equipped with stir bar, Grubbs' second generation catalyst (4 mg, 0.0047 mmol) and 3-allyl-4-benzyl-3-ethyl-5,5-dimethylmorpholin-2-one (86.1 mg, 0.3 mmol) were combined, followed by the addition of CH_2Cl_2 (3 mL) and ethyl acrylate (0.275 mL, 3.04 mmol). The vial was quickly subjected to three cycles of vacuum / nitrogen backfill and then sealed with a screw cap and Teflon septum, and heated at 60 ℃ for 24 hours. The reaction mixture was cooled to room temperature, filtered through celite, eluting with ethyl acetate, and concentrated *in vacuo*. The crude material was purified by flash column chromatography (80/20 40-60 ℃ Petroleum Ether/EtOAc) to provide (*E*)-ethyl 4-(4-benzyl-3-ethyl-5,5-dimethyl-2-oxomorpholin-3-yl)but-2-enoate as a colorless oil (79 mg, 74% yield).

A solution of (*E*)-ethyl 4-(4-benzyl-3-ethyl-5,5-dimethyl-2-oxomorpholin-3-yl)but-2-enoate (79 mg, 0.22 mmol) in methyl acetate (3 mL) was subjected to three cycles of vacuum / nitrogen backfill. Palladium on activated carbon (Pd/C, 10 wt. %, 42.4 mg) was added in one portion, the atmosphere was exchanged for hydrogen and the reaction stirred at room temperature overnight. The reaction was filtered through celite and concentrated *in vacuo* to provide the title compound as a colorless oil (54 mg, 91% yield). IR ν_{max}/cm^{-1} (film): 2971, 1728, 1461, 1378, 1180, 1055; ^1H NMR (400 MHz, $CDCl_3$) δ: 4.19-3.98 (m, 4H), 2.36-2.19 (m, 2H), 1.82-1.59 (m, 6H), 1.29-1.11 (m, 9H), 0.91 (t, *J* = 7.3 Hz, 3H); ^{13}C NMR (100 MHz, $CDCl_3$) δ: 173.412, 173.408, 77.4, 61.1, 60.4, 48.9, 39.2, 34.2, 33.1, 26.6, 26.4, 19.2, 14.3, 8.3; m/z HRMS (ESI) found $[M+H]^+$ 272.1855, $C_{14}H_{26}NO_4$ requires 272.1856.

2-(3-(3-Ethyl-5,5-dimethyl-2-oxomorpholin-3-yl)propyl)isoindoline-1,3-dione

(1h)

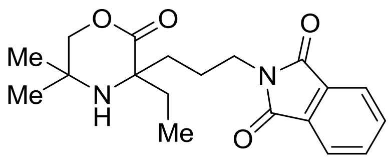

A solution of 2-(3-bromopropyl)isoindoline-1,3-dione (3.00 g, 11.2 mmol) and NaI (8.40 g, 55.8 mmol) in anhydrous acetone (25 mL) was refluxed for 14 hours. Then the solvent was removed, and the resulting residue was extracted (CH_2Cl_2/10% $Na_2S_2CO_3$ solution). The organic phase was dried ($MgSO_4$), filtered, and concentrated *in vacuo* to provide 2-(3-iodopropyl)isoindoline-1,3-dione as a light yellow solid (3.2 g, 91% yield).

A solution of 4-benzyl-3-ethyl-5,5-dimethylmorpholin-2-one (494 mg, 2.0 mmol) and 2-(3-iodopropyl)isoindoline-1,3-dione (3.2 g, 10.0 mmol) in dimethoxyethane (10 mL) and TMEDA (2 mL) was cooled to –78 ℃ and treated with potassium bis(trimethylsilyl)amide (12 mL, 0.5 M, 6 mmol) drop wise over 10 min and stirred for 1 h. After warming to room temperature and stirring for an additional 16 hours, the reaction mixture was diluted with EtOAc (20 mL), washed with saturated ammonium chloride solution (5 mL) and brine (2 x 5 mL). The organic phase was dried ($MgSO_4$), filtered, and concentrated *in vacuo*. The crude material was purified by flash column chromatography (70/30 40-60 ℃ Petroleum Ether/EtOAc) to provide 2-(3-(4-benzyl-3-ethyl-5,5-dimethyl-2-oxomorpholin-3-yl)propyl)isoindoline-1,3-dione as a colorless oil (259 mg, 30% yield).

A solution of 2-(3-(4-benzyl-3-ethyl-5,5-dimethyl-2-oxomorpholin-3-yl)propyl)isoindoline-1,3-dione (170 mg, 0.39 mmol) in MeOH (6 mL) was subjected to three cycles of vacuum / nitrogen backfill. Palladium on activated carbon (Pd/C, 10 wt. %, 170 mg) was added in one portion, the atmosphere was exchanged for hydrogen and the reaction stirred at room temperature overnight. Then additional palladium on activated carbon (Pd/C, 10 wt. %, 170 mg) was added and the reaction stirred at room temperature overnight again. Then the reaction was filtered through celite and concentrated *in vacuo*. The crude material was purified by flash column chromatography (50/50 40-60 ℃

Petroleum Ether/EtOAc) to provide the title compound as a colorless oil (72.3 mg, 54% yield). IR ν_{max}/cm^{-1} (film): 2969, 1704, 1395, 1053, 718; ^{1}H NMR (400 MHz, $CDCl_3$) δ: 7.86-7.77 (m, 2H), 7.74-7.62 (m, 2H), 4.10 (d, *J* = 10.8 Hz, 1H), 4.06 (d, *J* = 10.8 Hz, 1H), 3.72-3.62 (m, 2H), 1.81-1.54 (m, 6H), 1.15 (s, 3H), 1.14 (s, 3H), 0.89 (t, *J* = 7.4 Hz, 3H); ^{13}C NMR (100 MHz, $CDCl_3$) δ: 173.7, 168.5, 134.0, 132.2, 123.3, 77.7, 60.8, 48.6, 38.2, 37.4, 33.2, 26.8, 26.6, 23.1, 8.4; m/z HRMS (ESI) found $[M+H]^+$ 345.1807, $C_{19}H_{25}N_2O_4$ requires 345.1809.

3-Ethyl-5,5-dimethyl-3-(3-tosylpropyl)morpholin-2-one (1i)

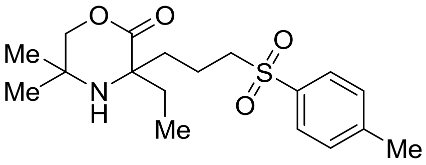

To a solution of ethylmagnesium bromide (3.0 M in diethyl ether, 40 mL, 120 mmol) in anhydrous THF (150 mL) at –78 ℃ was slowly added dropwise a solution of 4-chlorobutanoyl chloride (14.1 g, 100 mmol) in anhydrous THF (30 mL). After stirring for an additional 30 min, the resultant solution was gradually warmed to 0 ℃ and hydrolyzed by adding dilute hydrochloric acid (200 mL). The organic layer was separated and the aqueous layer was extracted with diethyl ether (2 x 200 mL). The combined organic layers were washed with saturated sodium carbonate solution (100 mL) and then with water (100 mL). The extracts were dried over $MgSO_4$, filtered and concentrated *in vacuo* to provide 6-chlorohexan-3-one (11.6 g, 86% yield).

6-Chlorohexan-3-one (4.0 g, 30 mmol) and sodium 4-methylbenzenesulfinate (6.4 g, 36 mmol) were refluxed in EtOH (60 mL) for 10 hours. The reaction mixture was cooled to room temperature, filtered through celite, eluting with ethyl acetate (100 mL), and concentrated *in vacuo*. The residue was purified by flash column chromatography (50/50 40-60 ℃ Petroleum Ether/EtOAc) to provide 6-tosylhexan-3-one (3.88 g, 51% yield).

A round bottom flask was charged with 2-amino-2-methyl-1-propanol (0.5 g, 5.6 mmol), $CHCl_3$ (10 mL), 6-tosylhexan-3-one (3.88 g, 15.2 mmol), benzyltriethylammonium chloride (28.5 mg, 0.125 mmol) and CH_2Cl_2 (100 mL). The

flask was equipped with a stir bar, and cooled to 0 ℃ using an ice bath. Powdered NaOH (3.0 g, 0.075 mol) was then added portion-wise at a rate that maintained the internal temperature below 5 ℃. After the addition was complete the reaction mixture was allowed to gradually warm to room temperature overnight. The resulting slurry was filtered on a frit, the white solid was washed with CH_2Cl_2 (20 mL) followed by methanol (50 mL) and the combined filtrates were concentrated *in vacuo* to provide the crude carboxylate. The amorphous solid was treated with a concentrated solution of hydrochloric acid (20 mL) and heated to reflux for 6 hours. The reaction mixture was cooled to room temperature and concentrated *in vacuo*. The residue was cooled to 0 ℃ and basified by the addition of a saturated aqueous solution of $NaHCO_3$. The aqueous solution was extracted with ethyl acetate (3 x 20 mL) and the combined organic extracts were dried ($MgSO_4$), filtered and concentrated *in vacuo*. The crude product was further purified by flash column chromatography (50/50 40-60 ℃ Petroleum Ether/EtOAc) to provide the title compound as a yellow amorphous solid (40 mg, 2% yield). IR ν_{max}/cm^{-1} (film): 2969, 1729, 1458, 1286, 1146, 816, 721; ^{1}H NMR (400 MHz, $CDCl_3$) δ: 7.76 (d, *J* = 8.1 Hz, 2H), 7.34 (d, *J* = 8.1 Hz, 2H), 4.07 (app s, 2H), 3.16-2.97 (m, 2H), 2.44 (s, 3H), 1.85-1.56 (m, 6H), 1.15 (s, 3H), 1.13 (s, 3H), 0.89 (t, *J* = 7.4 Hz, 3H); ^{13}C NMR (100 MHz, $CDCl_3$) δ: 173.5, 144.8, 136.3, 130.0, 128.2, 77.8, 60.8, 56.5, 48.6, 38.5, 33.4, 26.8, 26.5, 21.7, 17.6, 8.4; m/z HRMS (ESI) found $[M+H]^+$ 354.1735, $C_{18}H_{28}NO_4S$ requires 354.1734.

3-(But-3-enyl)-3-ethyl-5,5-dimethylmorpholin-2-one

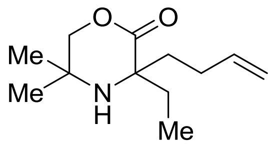

To a 500 mL round bottom flask equipped with stir bar was added pent-4-enoic acid (10.0 g, 100 mmol) and CH_2Cl_2 (250 mL). To this stirred solution was added 1,1'-carbonyl diimidazole (16.2 g, 100 mmol) in one portion, turning the solution yellow and resulting in the evolution of CO_2. The now yellow solution was allowed to stir for 45 minutes. At this time, *N-O*-dimethylhydroxylamine hydrochloride (11.7 g,

120 mmol) was added all at once and the reaction mixture was stirred overnight. The reaction mixture was then quenched with 100 mL of 1 M HCl and stirred vigorously for 10 minutes. After this time, the solution was transferred to a separating funnel and the layers were separated. The aqueous layer was extracted with CH_2Cl_2 (3 x 150 mL). The combine organic layers were washed with water (100 mL) and saturated sodium bicarbonate solution (100 mL). The organic layer was dried ($MgSO_4$) and concentrated *in vacuo* to provide *N*-methoxy-*N*-methylpent-4-enamide (12.1 g, 85% yield).

To a solution of *N*-methoxy-*N*-methylpent-4-enamide (12.1 g, 85 mmol) in anhydrous THF (350 mL) at 0 ℃ was slowly added ethylmagnesium bromide solution (3.0 M in diethyl ether, 35 mL, 105 mmol). The resulting mixture was stirred at 0 ℃ for 5 hours. A saturated aqueous NH_4Cl solution (150 mL) was added to quench the reaction and the reaction mixture was extracted with diethyl ether (3 x 150 mL). The combined organic layers were washed with brine (100 mL), dried ($MgSO_4$), filtered and concentrated *in vacuo* to provide hept-6-en-3-one (9.1 g, 95% yield).

A round bottom flask was charged with 2-amino-2-methyl-1-propanol (1.0 g, 11.2 mmol), $CHCl_3$ (10 mL), hept-6-en-3-one (9.1 g, 81 mmol), benzyltriethylammonium chloride (56.9 mg, 0.25 mmol) and CH_2Cl_2 (100 mL). The flask was equipped with a stir bar, and cooled to 0 ℃ using an ice bath. Powdered NaOH (3.0 g, 0.075 mol) was then added portion-wise at a rate that maintained the internal temperature below 5 ℃. After the addition was complete the reaction mixture was allowed to gradually warm to room temperature overnight. The resulting slurry was filtered on a frit, the white solid was washed with CH_2Cl_2 (30 mL) followed by methanol (100 mL) and the combined filtrates were concentrated *in vacuo* to provide the crude carboxylate. The amorphous solid was then suspended in toluene (250 mL) and camphorsulfonic acid was added (8.7 g, 37.5 mmol). The mixture was subjected to three cycles of vacuum / nitrogen backfill and the heated to reflux for 10 hours. The reaction mixture was cooled to room temperature concentrated *in vacuo* to approximately one quarter of the original volume and then diluted with EtOAc. The suspension was washed with a saturated aqueous solution of $NaHCO_3$. The organic layer was separated and the

aqueous layer was extracted with EtOAc (3 x 50 mL). The combined organic extracts were dried ($MgSO_4$), filtered and concentrated *in vacuo*. The crude material was purified by flash column chromatography (80/20 40-60 ℃ Petroleum Ether/EtOAc) to provide the title compound as a yellow oil (410 mg, 17% yield). IR ν_{max}/cm^{-1} (film): 2968, 1728, 1459, 1379, 1285, 1180, 1130, 1054, 997, 910; ^1H NMR (400 MHz, $CDCl_3$) δ: 5.80 (ddt, *J* = 16.8, 10.2, 6.5 Hz, 1H), 5.11-4.86 (m, 2H), 4.19-4.03 (m, 2H), 2.17-2.06 (m, 2H), 1.85-1.72 (m, 2H), 1.72-1.61 (m, 2H), 1.19 (s, 6H), 0.94 (t, *J* = 7.4 Hz, 3H); ^{13}C NMR (100 MHz, $CDCl_3$) δ: 174.0, 138.2, 115.0, 77.6, 61.0, 48.7, 39.5, 33.4, 28.3, 26.88, 26.87, 8.3; m/z HRMS (ESI) found $[M+H]^+$ 212.1644, $C_{12}H_{22}NO_2$ requires 212.1645.

3-Ethyl-5,5-dimethyl-3-(4-(4,4,5,5-tetramethyl-1,3,2-dioxaborolan-2-yl)butyl)morpholin-2-one (1j)

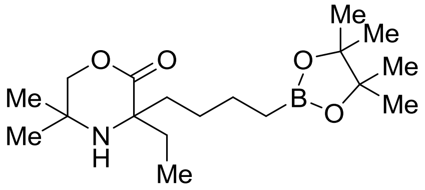

Under N_2, a 10 mL vial was charged with $[IrCODCl]_2$ (4.1 mg, 0.0625 mmol), 1,2-bis(diphenylphosphino)ethane (4.9 mg, 0.1225 mmol) and CH_2Cl_2 (2 mL). 3-(But-3-enyl)-3-ethyl-5,5-dimethylmorpholin-2-one (84 mg, 0.4 mmol) was dissolved in CH_2Cl_2 (0.5 mL) and added to the reaction mixture. The mixture was cooled to 0 ℃ and pinacolborane (90 μL, 0.6 mmol) was added dropwise. The reaction was warmed to room temperature and allowed to stir overnight. Water (1 mL) was added and the mixture was extracted with diethyl ether (3 x 5 mL). The organic layers were collected, dried ($MgSO_4$), filtered and concentrated *in vacuo*. The crude material was purified by flash column chromatography (80/20 40-60 ℃ Petroleum Ether/EtOAc) to provide the title compound as a colorless oil (69 mg, 51% yield). IR ν_{max}/cm^{-1} (film): 2975, 1732, 1372, 1143, 1056, 968, 847; ^1H NMR (400 MHz, $CDCl_3$) δ: 4.14-4.01 (m, 2H), 1.78-1.55 (m, 4H), 1.43-1.26 (m, 4H), 1.21 (s, 12H), 1.15 (s, 3H), 1.15 (s, 3H), 0.89 (t, *J* = 7.4 Hz, 3H), 0.76 (t, *J* = 7.5 Hz, 2H); ^{13}C NMR (100

MHz, $CDCl_3$, C attached to B not observed) δ: 174.2, 83.0, 77.5, 61.1, 48.6, 40.1, 33.2, 26.9, 26.8, 26.4, 24.9, 24.4, 8.3; ^{11}B NMR (128 MHz, $CDCl_3$) δ: 34.0; m/z HRMS (ESI) found $[M+H]^+$ 339.2696, $C_{18}H_{35}{}^{10}BNO_4$ requires 339.2690.

2,2-Diethyl-4-oxa-1-azaspiro[5.5]undecan-3-one (1k)

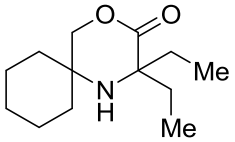

A 250 mL three-necked round bottom flask was charged with (1-aminocyclohexyl)methanol (2.58 g, 20 mmol), $CHCl_3$ (10 mL) and pentan-3-one (25 mL). The flask was equipped with a stir bar, and cooled to 0 ℃ using an ice bath. Powdered NaOH (5.0 g, 0.125 mol) was then added portion-wise at a rate that maintained the internal temperature below 5 ℃. After the addition was complete the reaction mixture was allowed to gradually warm to room temperature overnight. The resulting slurry was filtered on a frit, the white solid was washed with CH_2Cl_2 (50 mL) followed by methanol (100 mL) and the combined filtrates were concentrated *in vacuo* to provide the crude carboxylate. The amorphous solid was treated with a concentrated solution of hydrochloric acid (30 mL) and heated to reflux for 8 hours. The reaction mixture was cooled to room temperature and concentrated *in vacuo*. The residue was cooled to 0 ℃ and basified by the addition of a saturated aqueous solution of $NaHCO_3$. The aqueous solution was extracted with ethyl acetate (3 x 30 mL) and the combined organic extracts were dried ($MgSO_4$), filtered and concentrated *in vacuo*. The crude product was further purified by Kugelrohr distillation to provide the title compound as a colorless oil (1.2 g, 27% yield). IR ν_{max}/cm^{-1} (film): 2928, 1731, 1453, 1394, 1227, 1049; ^1H NMR (400 MHz, $CDCl_3$) δ: 4.11 (s, 2H), 1.72-1.59 (m, 4H), 1.59-1.33 (m, 10H), 0.90 (t, J = 7.4 Hz, 6H); ^{13}C NMR (100 MHz, $CDCl_3$) δ: 174.6, 76.3, 61.2, 49.9, 35.2, 32.8, 25.9, 21.8, 8.2; m/z HRMS (ESI) found $[M+H]^+$ 226.1799, $C_{13}H_{24}NO_2$ requires 226.1802.

(*S*)-3,3-Diethyl-5-methylmorpholin-2-one (1l)

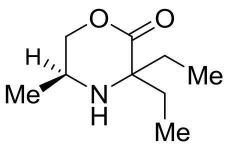

A 250 mL three-necked round bottom flask was charged with L-alaninol (1.8 g, 24 mmol), $CHCl_3$ (10 mL) and pentan-3-one (25 mL). The flask was equipped with a stir bar, and cooled to 0 ℃ using an ice bath. Powdered NaOH (5.0 g, 0.125 mol) was then added portion-wise at a rate that maintained the internal temperature below 5 ℃. After the addition was complete the reaction mixture was allowed to gradually warm to room temperature overnight. The resulting slurry was filtered on a frit, the white solid was washed with CH_2Cl_2 (50 mL) followed by methanol (100 mL) and the combined filtrates were concentrated *in vacuo* to provide the crude carboxylate. The amorphous solid was treated with a concentrated solution of hydrochloric acid (30 mL) and heated to reflux for 12 hours. The reaction mixture was cooled to room temperature and concentrated *in vacuo*. The residue was cooled to 0 ℃ and basified by the addition of a saturated aqueous solution of $NaHCO_3$. The aqueous solution was extracted with ethyl acetate (3 x 30 mL) and the combined organic extracts were dried ($MgSO_4$), filtered and concentrated *in vacuo*. The crude product was further purified by Kugelrohr distillation to provide the title compound as a colorless oil (1.2 g, 30% yield). IR ν_{max}/cm^{-1} (film): 2972, 1726, 1462, 1314, 1218, 1182, 1149; ^1H NMR (400 MHz, $CDCl_3$) δ: 4.20 (dd, *J* = 10.5, 3.1 Hz, 1H), 3.91 (t, *J* = 10.5 Hz, 1H), 3.38-3.23 (m, 1H), 2.00-1.88 (m, 1H), 1.86-1.74 (m, 1H), 1.72-1.59 (m, 1H), 1.53-1.40 (m, 1H), 1.06 (d, *J* = 6.3 Hz, 3H), 1.00-0.85 (m, 6H); ^{13}C NMR (100 MHz, $CDCl_3$) δ: 173.4, 76.1, 63.7, 44.1, 32.22, 32.16, 17.2, 8.7, 8.1; m/z HRMS (ESI) found $[M+H]^+$ 172.1327, $C_9H_{18}NO_2$ requires 172.1332.

(*S*)-3,3-Diethyl-5-isopropylmorpholin-2-one (1m)

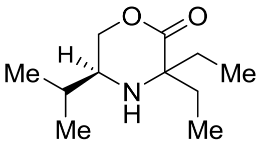

A 250 mL three-necked round bottom flask was charged with L-valinol (2.5 g, 24

mmol), $CHCl_3$ (10 mL) and pentan-3-one (25 mL). The flask was equipped with a stir bar, and cooled to 0 ℃ using an ice bath. Powdered NaOH (5.0 g, 0.125 mol) was then added portion-wise at a rate that maintained the internal temperature below 5 ℃. After the addition was complete the reaction mixture was allowed to gradually warm to room temperature overnight. The resulting slurry was filtered on a frit, the white solid was washed with CH_2Cl_2 (50 mL) followed by methanol (100 mL) and the combined filtrates were concentrated *in vacuo* to provide the crude carboxylate. The amorphous solid was treated with a concentrated solution of hydrochloric acid (30 mL) and heated to reflux for 12 hours. The reaction mixture was cooled to room temperature and concentrated *in vacuo*. The residue was cooled to 0 ℃ and basified by the addition of a saturated aqueous solution of $NaHCO_3$. The aqueous solution was extracted with ethyl acetate (3 x 30 mL) and the combined organic extracts were dried ($MgSO_4$), filtered and concentrated *in vacuo*. The crude product was further purified by flash column chromatography (80/20 40-60 ℃ Petroleum Ether/EtOAc) to provide the title compound as a colorless oil (0.75 g, 16% yield). IR ν_{max}/cm^{-1} (film): 2966, 1726, 1461, 1313, 1219, 1169, 1027, 727; ^1H NMR (400 MHz, $CDCl_3$) δ: 4.33 (dd, *J* = 10.5, 3.2 Hz, 1H), 4.05 (t, *J* = 10.5 Hz, 1H), 2.91-2.81 (m, 1H), 1.95 (dq, *J* = 15.0, 7.5 Hz, 1H), 1.82 (dq, *J* = 14.7, 7.4 Hz, 1H), 1.72-1.55 (m, 2H), 1.50 (dq, *J* = 14.7, 7.4 Hz, 1H), 1.09-0.84 (m, 12H); ^{13}C NMR (100 MHz, $CDCl_3$) δ: 174.0, 74.0, 63.4, 53.8, 32.1, 31.7, 30.4, 19.0, 18.9, 8.7, 7.8; m/z HRMS (ESI) found $[M+H]^+$ 200.1642, $C_{11}H_{22}NO_2$ requires 200.1645.

3-Cyclopropyl-3,5,5-trimethylmorpholin-2-one (1n)

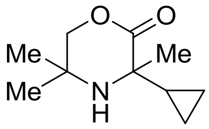

A 250 mL three-necked round bottom flask was charged with 2-amino-2-methyl-1-propanol (2.5 g, 28 mmol), $CHCl_3$ (10 mL) and 1-cyclopropylethanone (30 mL). The flask was equipped with a stir bar, and cooled to 0 ℃ using an ice bath. Powdered NaOH (5.0 g, 0.125 mol) was then added

portion-wise at a rate that maintained the internal temperature below 5 ℃. After the addition was complete the reaction mixture was allowed to gradually warm to room temperature overnight. The resulting slurry was filtered on a frit, the white solid was washed with CH_2Cl_2 (50 mL) followed by methanol (100 mL) and the combined filtrates were concentrated *in vacuo* to provide the crude carboxylate. The amorphous solid was treated with a concentrated solution of hydrochloric acid (30 mL) and heated to reflux for 8 hours. The reaction mixture was cooled to room temperature and concentrated *in vacuo*. The residue was cooled to 0 ℃ and basified by the addition of a saturated aqueous solution of $NaHCO_3$. The aqueous solution was extracted with ethyl acetate (3 x 30 mL) and the combined organic extracts were dried ($MgSO_4$), filtered and concentrated *in vacuo*. The crude product was further purified by Kugelrohr distillation to provide the title compound as a colorless oil (2.0 g, 39% yield). IR ν_{max}/cm^{-1} (film): 2973, 1727, 1471, 1379, 1230, 1105, 1048, 843, 712; ^1H NMR (400 MHz, $CDCl_3$) δ: 4.18 (d, *J* = 10.6 Hz, 1H), 4.00 (d, *J* = 10.6 Hz, 1H), 1.40 (s, 3H), 1.22 (s, 3H), 1.20-1.12 (m, 1H), 1.10 (s, 3H), 0.57-0.48 (m, 1H), 0.46-0.34 (m, 2H), 0.34-0.20 (m, 1H); ^{13}C NMR (100 MHz, $CDCl_3$) δ: 174.6, 77.6, 56.7, 49.0, 29.1, 26.9, 26.2, 21.8, 1.3, 0.6; m/z HRMS (ESI) found $[M+H]^+$ 184.1328, $C_{10}H_{18}NO_2$ requires 184.1332.

3-Benzyl-3-ethyl-5,5-dimethylmorpholin-2-one (1o)

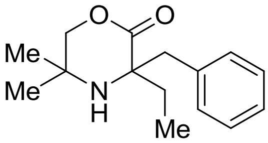

A 100 mL three-necked round bottom flask was charged with 2-amino-2-methyl-1-propanol (1.0 g, 11.2 mmol), $CHCl_3$ (30 mL) and 1-phenylbutan-2-one (5.0 g, 33.7 mmol). The flask was equipped with a stir bar, and cooled to 0 ℃ using an ice bath. Powdered NaOH (3.0 g, 0.075 mol) was then added portion-wise at a rate that maintained the internal temperature below 5 ℃. After the addition was complete the reaction mixture was allowed to gradually warm to room temperature overnight. The resulting slurry was filtered on a frit, the white solid was

washed with CH_2Cl_2 (30 mL) followed by methanol (100 mL) and the combined filtrates were concentrated *in vacuo* to provide the crude carboxylate. The amorphous solid was treated with a concentrated solution of hydrochloric acid (30 mL) and heated to reflux for 8 hours. The reaction mixture was cooled to room temperature and concentrated *in vacuo*. The residue was cooled to 0 °C and basified by the addition of a saturated aqueous solution of $NaHCO_3$. The aqueous solution was extracted with ethyl acetate (3 x 30 mL) and the combined organic extracts were dried ($MgSO_4$), filtered and concentrated *in vacuo*. The crude product was further purified by flash column chromatography (80/20 40-60 °C Petroleum Ether/EtOAc) to provide the title compound as an amorphous solid (250 mg, 9% yield). IR ν_{max}/cm^{-1} (film): 2929, 1732, 1456, 1379, 1288, 1176, 702; ^1H NMR (400 MHz, $CDCl_3$) δ: 7.38-7.16 (m, 5H), 3.83 (d, J = 10.6 Hz, 1H), 3.31 (d, J = 10.6 Hz, 1H), 3.14 (d, J = 13.2 Hz, 1H), 2.78 (d, J = 13.2 Hz, 1H), 1.90 (dq, J = 14.8, 7.4 Hz, 1H), 1.69-1.57 (m, 1H), 1.14 (s, 3H), 1.05-0.94 (m, 6H); ^{13}C NMR (100 MHz, $CDCl_3$) δ: 174.1, 136.5, 131.0, 128.4, 127.3, 77.3, 62.6, 48.2, 46.4, 34.8, 26.6, 26.4, 8.5; m/z HRMS (ESI) found $[M+H]^+$ 248.1646, $C_{15}H_{22}NO_2$ requires 248.1645.

3-Ethyl-5,5-dimethyl-3-phenylmorpholin-2-one

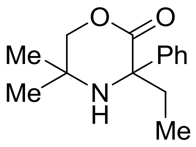

A 250 mL three-necked round bottom flask was charged with 2-amino-2-methyl-1-propanol (2.2 g, 25 mmol), $CHCl_3$ (20 mL) and propiophenone (20 mL). The flask was equipped with a stir bar, and cooled to 0 °C using an ice bath. Powdered NaOH (5.0 g, 0.125 mol) was then added portion-wise at a rate that maintained the internal temperature below 5 °C. After the addition was complete the reaction mixture was allowed to gradually warm to room temperature overnight. The resulting slurry was filtered on a frit, the white solid was washed with CH_2Cl_2 (50 mL) followed by methanol (100 mL) and the combined filtrates were concentrated *in vacuo* to provide the crude carboxylate. The amorphous solid was treated with a

concentrated solution of hydrochloric acid (30 mL) and heated to reflux for 8 hours. The reaction mixture was cooled to room temperature and concentrated *in vacuo*. The residue was cooled to 0 ℃ and basified by the addition of a saturated aqueous solution of $NaHCO_3$. The aqueous solution was extracted with ethyl acetate (3 x 30 mL) and the combined organic extracts were dried ($MgSO_4$), filtered and concentrated *in vacuo*. The crude material was purified by flash column chromatography (85/15 40-60 ℃ Petroleum Ether/EtOAc) to provide the title compound as a white solid (1.0 g, 17% yield). m.p. 62-64 ℃; IR ν_{max}/cm^{-1} (film): 2968, 1734, 1470, 1374, 1285, 1191, 1054, 898, 701; ^{1}H NMR (400 MHz, $CDCl_3$) δ: 7.63-7.53 (m, 2H), 7.39-7.30 (m, 2H), 7.30-7.24 (m, 1H), 3.75 (d, *J* = 11.5 Hz, 1H), 3.70 (d, *J* = 11.5 Hz, 1H), 1.95 (dq, *J* = 14.7, 7.4 Hz, 1H), 1.77 (dq, *J* = 14.7, 7.4 Hz, 1H), 1.28 (s, 3H), 1.14 (s, 3H), 0.82 (t, *J* = 7.4 Hz, 3H); ^{13}C NMR (125 MHz, $CDCl_3$) δ: 173.1, 142.0, 128.6, 127.7, 126.0, 73.3, 64.7, 50.8, 35.5, 28.5, 27.4, 8.6; m/z HRMS (ESI) found [M+H]$^+$ 234.1487, $C_{14}H_{20}NO_2$ requires 234.1489.

3-Isopropyl-3,5,5-trimethylmorpholin-2-one

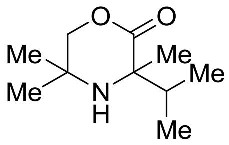

A 250 mL three-necked round bottom flask was charged with 2-amino-2-methyl-1-propanol (2.5 g, 28 mmol), $CHCl_3$ (20 mL) and 3-methylbutan-2-one (30 mL). The flask was equipped with a stir bar, and cooled to 0 ℃ using an ice bath. Powdered NaOH (5.0 g, 0.125 mol) was then added portion-wise at a rate that maintained the internal temperature below 5 ℃. After the addition was complete the reaction mixture was allowed to gradually warm to room temperature overnight. The resulting slurry was filtered on a frit, the white solid was washed with CH_2Cl_2 (50 mL) followed by methanol (100 mL) and the combined filtrates were concentrated *in vacuo* to provide the crude carboxylate. The amorphous solid was treated with a concentrated solution of hydrochloric acid (30 mL) and heated to reflux for 8 hours. The reaction mixture was cooled to room temperature and concentrated *in*

vacuo. The residue was cooled to 0 ℃ and basified by the addition of a saturated aqueous solution of $NaHCO_3$. The aqueous solution was extracted with ethyl acetate (3 x 30 mL) and the combined organic extracts were dried ($MgSO_4$), filtered and concentrated *in vacuo*. The crude product was further purified by Kugelrohr distillation to provide the title compound as a colorless oil (1.2 g, 23% yield); IR ν_{max}/cm^{-1} (film): 2970, 1727, 1471, 1377, 1285, 1226, 1136, 1060; ^1H NMR (400 MHz, $CDCl_3$) δ: 4.11 (d, *J* = 10.6 Hz, 1H), 4.00 (d, *J* = 10.6 Hz, 1H), 2.01-1.88 (m, 1H), 1.38 (s, 3H), 1.25 (s, 3H), 1.11 (s, 3H), 0.94 (d, *J* = 6.8 Hz, 3H), 0.88 (d, *J* = 6.8 Hz, 3H); ^{13}C NMR (125 MHz, $CDCl_3$) δ: 174.9, 77.7, 61.0, 48.5, 38.5, 27.9, 27.2, 25.9, 17.9, 16.1; m/z HRMS (ESI) found $[M+H]^+$ 186.1486, $C_{10}H_{20}NO_2$ requires 186.1489.

5,5-Dimethyl-3,3-dipropylmorpholin-2-one

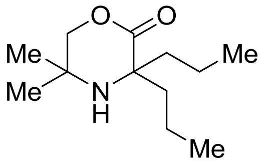

A 250 mL three-necked round bottom flask was charged with 2-amino-2-methyl-1-propanol (1.78 g, 20 mmol), $CHCl_3$ (10 mL) and 4-heptanone (25 mL). The flask was equipped with a stir bar, and cooled to 0 ℃ using an ice bath. Powdered NaOH (5.0 g, 0.125 mol) was then added portion-wise at a rate that maintained the internal temperature below 5 ℃. After the addition was complete the reaction mixture was allowed to gradually warm to room temperature overnight. The resulting slurry was filtered on a frit, the white solid was washed with CH_2Cl_2 (50 mL) followed by methanol (100 mL) and the combined filtrates were concentrated *in vacuo* to provide the crude carboxylate. The amorphous solid was treated with a concentrated solution of hydrochloric acid (30 mL) and heated to reflux for 8 hours. The reaction mixture was cooled to room temperature and concentrated *in vacuo*. The residue was cooled to 0 ℃ and basified by the addition of a saturated aqueous solution of $NaHCO_3$. The aqueous solution was extracted with ethyl acetate (3 x 30 mL) and the combined organic extracts were dried ($MgSO_4$), filtered and concentrated *in vacuo*.

The crude product was further purified by Kugelrohr distillation to provide the title compound as a colorless oil (0.93 g, 22% yield). IR ν_{max}/cm^{-1} (film): 2960, 1729, 1467, 1378, 1283, 1182, 1058, 897, 808, 731; ^1H NMR (400 MHz, $CDCl_3$) δ: 4.05 (s, 2H), 1.67-1.50 (m, 4H), 1.37-1.27 (m, 4H), 1.13 (s, 6H), 0.87 (t, J = 7.3 Hz, 6H); ^{13}C NMR (100 MHz, $CDCl_3$) δ: 174.3, 77.6, 61.0, 48.7, 43.3, 26.9, 17.2, 14.5; m/z HRMS (ESI) found $[M+H]^+$ 214.1801, $C_{12}H_{24}NO_2$ requires 214.1802.

3,3-Diethyl-5,5-dimethylmorpholin-2-one (d_5, d_5) (d_{10}-1b)

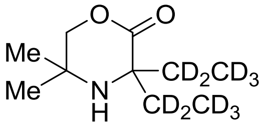

A solution of 4-benzyl-5,5-dimethylmorpholin-2-one (438 mg, 2.0 mmol) and iodoethane-d_5 (1.61 g, 10.0 mmol) in THF (10 mL) and TMEDA (2 mL) was cooled to –78 ℃ and treated with potassium bis(trimethylsilyl)amide (15 mL, 0.7 M in toluene, 10.5 mmol) drop wise over 10 min and stirred for 1 h. After warming to room temperature and stirring for an additional 16 hours, the reaction mixture was diluted with EtOAc (20 mL), washed with saturated ammonium chloride solution (5 mL) and brine (2 x 5 mL). The organic phase was dried ($MgSO_4$), filtered, and concentrated *in vacuo*. The crude material was purified by flash column chromatography (90/10 40-60 ℃ Petroleum Ether/EtOAc) to provide 4-benzyl-3,3-diethyl-5,5-dimethylmorpholin-2-one (d_5, d_5) as a colorless oil (407.3 mg, 71% yield).

A solution of 4-benzyl-3,3-diethyl-5,5-dimethylmorpholin-2-one (d_5, d_5) (331.2 mg, 1.16 mmol) in methyl acetate (8 mL) was subjected to three cycles of vacuum / nitrogen backfill. Palladium on activated carbon (Pd/C, 10 wt. %, 123.1 mg) was added in one portion, the atmosphere was exchanged for hydrogen and the reaction stirred at room temperature overnight. The reaction was filtered through celite and concentrated *in vacuo* to provide the title compound as a colorless oil (222.4 mg, 98% yield). IR ν_{max}/cm^{-1} (film): 2973, 1726, 1470, 1378, 1223, 1052; ^1H NMR (500 MHz, $CDCl_3$) δ: 4.11 (s, 2H), 1.21 (s, 6H); ^{13}C NMR (125 MHz, $CDCl_3$) δ: 174.2, 77.4, 61.2,

48.7, 31.8 (qn, *J* = 19.0 Hz), 26.8, 7.3 (sp, *J* = 19.0 Hz); m/z HRMS (ESI) found $[M+H]^+$ 196.2114, $C_{10}H_{10}D_{10}NO_2$ requires 196.2116.

3,3-Diethyl-5,5-dimethylmorpholin-2-one (d_5) (d_5-1b)

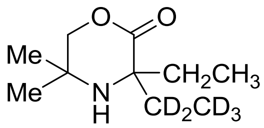

A solution of 4-benzyl-3-ethyl-5,5-dimethylmorpholin-2-one (494 mg, 2.0 mmol) and iodoethane-d_5 (2.4 g, 15.0 mmol) in THF (10 mL) and TMEDA (2 mL) was cooled to –78 °C and treated with potassium bis(trimethylsilyl)amide (15 mL, 0.7 M, 10.5 mmol) drop wise over 10 min and stirred for 1 h. After warming to room temperature and stirring for an additional 16 hours, the reaction mixture was diluted with EtOAc (20 mL), washed with saturated ammonium chloride solution (5 mL) and brine (2 x 5 mL). The organic phase was dried ($MgSO_4$), filtered, and concentrated *in vacuo*. The crude material was purified by flash column chromatography (80/20 40-60 °C Petroleum Ether/EtOAc) to provide 4-benzyl-3,3-diethyl-5,5-dimethylmorpholin-2-one (d_5) as a colorless oil (515 mg, 92% yield).

A solution of 4-benzyl-3,3-diethyl-5,5-dimethylmorpholin-2-one (d_5) (515 mg, 1.84 mmol) in methyl acetate (8 mL) was subjected to three cycles of vacuum / nitrogen backfill. Palladium on activated carbon (Pd/C, 10 wt. %, 195 mg) was added in one portion, the atmosphere was exchanged for hydrogen and the reaction stirred at room temperature overnight. The reaction was filtered through celite and concentrated *in vacuo* to provide the title compound as a colorless oil (317 mg, 91% yield). IR ν_{max}/cm^{-1} (film): 2971, 1730, 1462, 1379, 1227, 1056; ^1H NMR (400 MHz, $CDCl_3$) δ: 4.11 (s, 2H), 1.82-1.70 (m, 1H), 1.67-1.58 (m, 1H), 1.20 (s, 6H), 0.93 (t, *J* = 7.4 Hz, 3H); ^{13}C NMR (100 MHz, $CDCl_3$) δ: 174.2, 77.4, 61.3, 48.7, 32.9, 31.9 (qn, *J* = 19.2 Hz), 26.8, 8.3, 7.3 (sp, *J* = 19.2 Hz); m/z HRMS (ESI) found $[M+H]^+$ 191.1799, $C_{10}H_{15}D_5NO_2$ requires 191.1802.

3. Palladium Complexes and X-Ray Crystallography

Formation of the four-membered ring palladacycle int-I and five-membered ring palladacycle int-II mixture

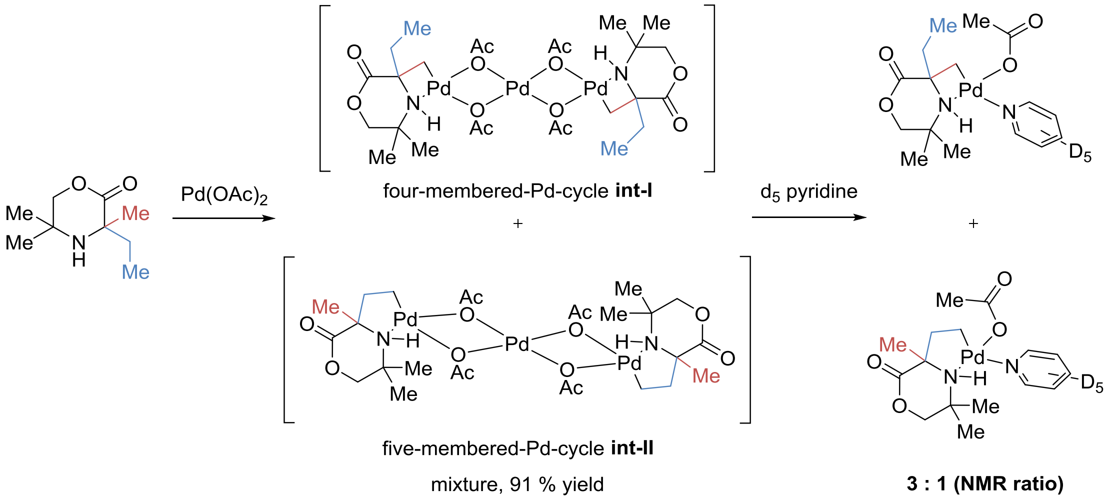

3-Ethyl-3,5,5-trimethylmorpholin-2-one (85.6 mg, 0.5 mmol) and $Pd(OAc)_2$ (168.4 mg, 0.75 mmol) were stirred in $CDCl_3$ (4 mL) at 35 ℃ for 20 hours under air. The solution was cooled to room temperature and filtered through celite, eluting with $CHCl_3$. The solvent was then removed *in vacuo* and the residue was re-dissolved in a minimum amount of $CHCl_3$. This solution was added dropwise to 15 mL of 40-60 ℃ petroleum ether and the resulting precipitate was filtered, rinsed with 40-60 ℃ petroleum ether and dried *in vacuo* to afford a green solid (205 mg, 91% yield). IR ν_{max}/cm^{-1} (film): 2973, 1738, 1573, 1395, 1255, 1056, 698.

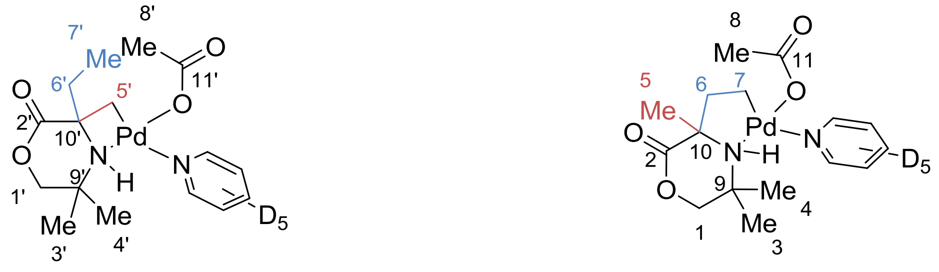

four-membered-Pd-cycle **3 : 1 (NMR ratio)** five-membered-Pd-cycle

Resolved NMR spectra were obtained by treating the green solid with deuterated pyridine (2 drops added to 15 mg of this green solid in $CDCl_3$) to generate the corresponding pyridine complex. The NMR results indicated the formation of a four-membered ring palladacycle as well as a five-membered ring palladacycle with

3:1 ratio in this C–H activation reaction. ^{1}H NMR (500 MHz, $CDCl_3$) δ: four-membered-Pd-cycle **int-I**: 4.51-4.28 ($H_{1'}$, m, 1H), 4.07-4.03 ($H_{1'}$, m, 1H), 2.40-2.31 ($H_{6'}$, m, 1H), 2.10 ($H_{6'}$, dq, *J* = 14.9, 7.4 Hz, 1H), 1.81 ($H_{8'}$, s, 3H), 1.67 ($H_{3'}$ or $H_{4'}$, s, 3H), 1.19 ($H_{3'}$ or $H_{4'}$, s, 3H), 1.08 ($H_{7'}$, t, *J* = 7.4 Hz, 3H), 0.71 ($H_{5'}$, d, *J* = 6.9 Hz, 1H), 0.67 ($H_{5'}$, d, *J* = 6.9 Hz, 1H). Five-membered-Pd-cycle **int-II**: 4.25 (H_1, d, *J* = 11.7 Hz, 1H), 4.18-4.13 (H_1, m, 1H), 2.31-2.25 (H_6, m, 1H), 2.03-1.96 (H_7, m, 1H), 1.95 (H_5, s, 3H), 1.94 (H_8, s, 3H), 1.79-1.75 (H_7, m, 1H), 1.72 (H_3 or H_4, s, 3H), 1.52-1.46 (H_6, m, 1H), 1.32 (H_3 or H_4, s, 3H). ^{13}C NMR (125 MHz, $CDCl_3$) δ: 179.1, 178.3, 172.1, 171.6, 151.3 (t, *J* = 28.1 Hz), 149.5 (t, *J* = 26.9 Hz), 138.1 (t, *J* = 24.4 Hz), 135.5 (t, *J* = 24.4 Hz), 124.3 (t, *J* = 25.6 Hz), 123.2 (t, *J* = 25.0 Hz), 76.6, 74.6, 74.4, 68.5, 53.9, 52.6, 45.6, 33.4, 26.3, 26.0, 25.5, 25.0, 24.3, 23.3, 22.7, 18.3, 8.5, -6.7.

Stoichiometric reaction between the palladacycles (int-I/II) mixture with amino-acid-derived ligand

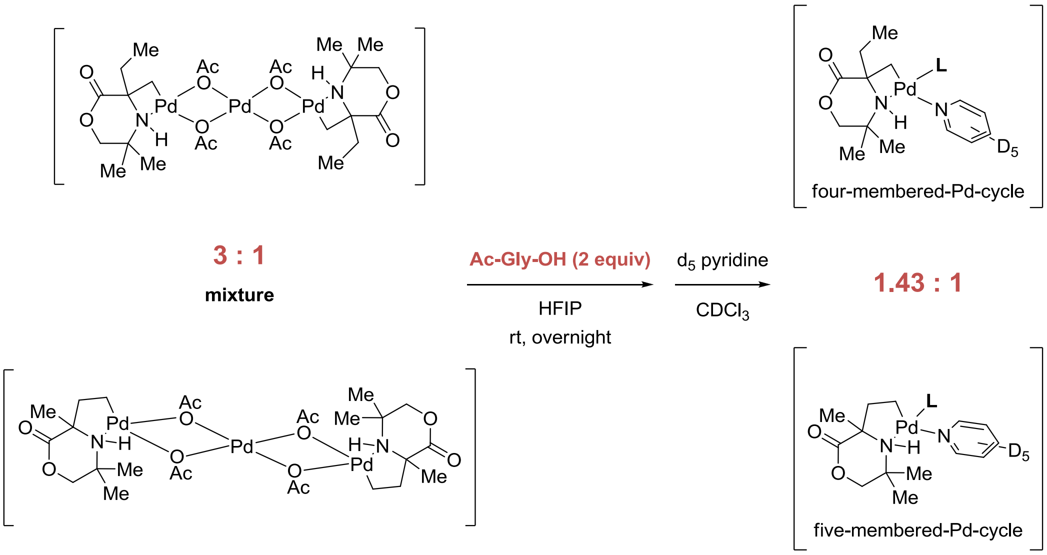

In a 10 mL vial equipped with stir bar, palladacycles (**int-I/II**) mixture (17.9 mg, 0.02 mmol) and Ac-Gly-OH (4.7 mg, 0.04 mmol) were added, followed by the addition of 1,1,1,3,3,3-Hexafluoropropan-2-ol (HFIP) (0.5 mL). Then the vial was sealed under air with a screw cap and Teflon septum, and stirred at room temperature overnight. The solvent was then removed *in vacuo* and the residue was dissolved in deuterated

chloroform and treated with a couple of drops of deuterated pyridine to generate the monomeric pyridine complex. The crude NMR result indicated that the ratio between the four-membered ring palladacycle and the five-membered ring palladacycle changed to 1.43:1.

Trinuclear five-membered ring palladacycle int-V

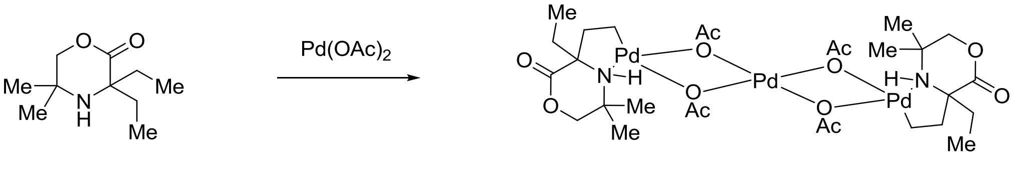

3,3-Diethyl-5,5-dimethylmorpholin-2-one (92.5 mg, 0.5 mmol) and $Pd(OAc)_2$ (168.4 mg, 0.75 mmol) were stirred in $CHCl_3$ (4 mL) at 60 ℃ for 9 hours under air. The solution was cooled to room temperature and filtered through celite, eluting with $CHCl_3$. The solvent was then removed *in vacuo* and the residue was re-dissolved in a minimum amount of $CHCl_3$. This solution was added dropwise to 40-60 ℃ petroleum ether (15 mL) and the resulting precipitate was filtered, rinsed with 40-60 ℃ petroleum ether and dried *in vacuo* to afford the title palladacycle as a green solid (192 mg, 84% yield). IR ν_{max}/cm^{-1} (film): 2974, 1736, 1577, 1394, 1258, 1066, 698. Crystals were grown by vial-in-vial diffusion of diethyl ether into a CH_2Cl_2 solution of the palladacycle at room temperature. X-Ray crystal structure is deposited in the Cambridge Crystallographic Data Centre CCDC 1449203.

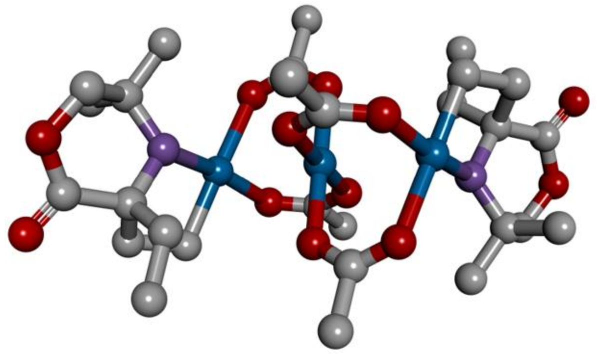

Figure S1. X-ray crystal structure of trinuclear five-membered ring palladacycle **int-V**. Hydrogens are removed for clarity.

Mononuclear five-membered ring palladacycle-*d*$_5$-pyridine complex

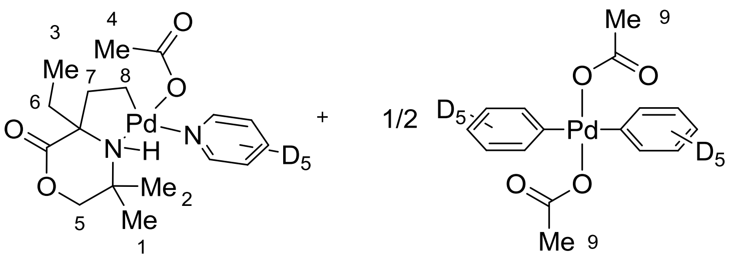

Resolved NMR spectra were obtained by treating the trinuclear palladacycle **int-V** with deuterated pyridine (2 drops added to 15 mg of palladacycle in $CDCl_3$) to generate the corresponding pyridine complex. ^1H NMR (400 MHz, $CDCl_3$) δ: 4.16 (H_5, d, J = 11.8 Hz, 1H), 4.11 (H_5, d, J = 11.8 Hz, 1H), 2.91-2.83 (H_6, m, 1H), 2.50-2.42 (H_7, m, 1H), 2.09-1.97 (H_8 and H_6, m, 2H), 1.91 (H_9, s, 3H), 1.81 (H_4, s, 3H), 1.74 (H_1 or H_2, s, 3H), 1.73-1.67 (H_8, m, 1H), 1.42-1.36 (H_7, m, 1H), 1.35 (H_1 or H_2, s, 3H), 1.16 (H_3, t, J = 7.4 Hz, 3H); ^{13}C NMR (100 MHz, $CDCl_3$) δ: 179.4, 178.3, 170.7, 151.3 (t, J = 28.5 Hz), 149.5 (t, J = 27.0 Hz), 137.9 (t, J = 26.0 Hz), 135.5 (t, J = 24.5 Hz), 124.4 (t, J = 25.0 Hz), 123.2 (t, J = 24.5 Hz), 76.6, 73.9, 53.9, 45.2, 31.6, 25.4, 25.3, 24.1, 23.3, 18.9, 9.0.

4. C(sp^3)–H Alkenylation Reactions

4.1 General Procedure for the Stoichiometric C–H Alkenylation

In a 10 mL vial equipped with stir bar, palladacycle (0.02 mmol), Ac-Gly-OH (4.7 mg, 0.04 mmol) were added, followed by the addition of 1,1,1,3,3,3-Hexafluoropropan-2-ol (HFIP) (0.5 mL) and ethyl acrylate (12 mg, 0.12 mmol). Then the vial was sealed under air with a screw cap and Teflon septum, and placed in a pre-heated oil bath at 60 ℃ stirred for 2 hours. The reaction mixture was cooled to room temperature and filtered through celite, eluting with ethyl acetate. Yields were determined by ^1H NMR against triphenylmethane as internal standard.

4.2 General Procedure for the Catalytic C–H Alkenylation

In a 10 mL vial equipped with stir bar, $Pd(OAc)_2$ (4.5 mg, 0.02 mmol), Ac-Gly-OH (4.7 mg, 0.04 mmol), AgOAc (100.1 mg, 0.6 mmol) were combined, followed by the addition of 1,1,1,3,3,3-Hexafluoropropan-2-ol (HFIP) (1 mL), amine substrates (0.2 mmol), and alkenes (0.4 mmol). Then the vial was sealed under air with a screw cap and Teflon septum, placed in a pre-heated oil bath at the described temperature and stirred for the stated time. The reaction mixture was cooled to room temperature, filtered through celite, eluting with ethyl acetate, and concentrated *in vacuo*. The crude product was purified by flash column chromatography (silica gel) under the stated conditions to provide the pure product.

4.3 Catalytic Condition Optimization

Table S1. Optimization for catalytic C–H alkenylation.[a]

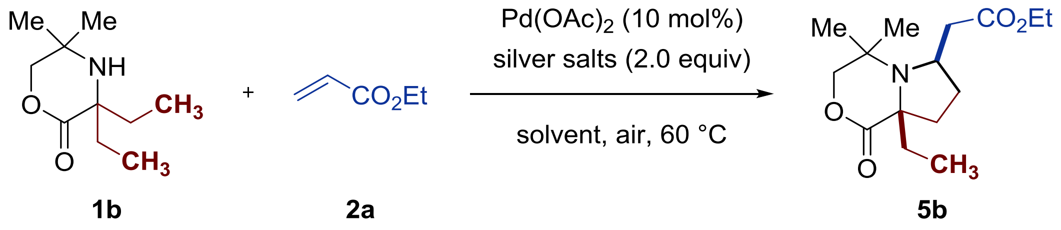

Entry	Oxidant	Solvent	Additive	Yield (%)
1	Ag_2CO_3	HFIP	-	34
2	Ag_2O	HFIP	-	1
3	AgOAc	HFIP	-	**70**
4	O_2	HFIP	-	23
5	AgOAc	HFIP	no Pd	0
6	AgOAc	*t*-amyl-OH	-	trace
7	AgOAc	TFE	-	30
8	AgOAc	toluene	-	2
9	AgOAc	THF	-	0
10	AgOAc	DMF	-	0
11	AgOAc	DCE	-	4
**12**	AgOAc	HFIP	Ac-Gly-OH (20 mol%)	**86 (86)**

[a] Reactions performed with 0.1 mmol of **1b** and 0.2 mmol of **2a**; yields of **5b** are determined by GC against triphenylmethane as internal standard, yield in bracket is isolated yield.

4.4 Data

Ethyl 2-(8a-ethyl-4,4-dimethyl-1-oxohexahydro-1*H*-pyrrolo[2,1-*c*][1,4]oxazin-6-yl) acetate (5b)

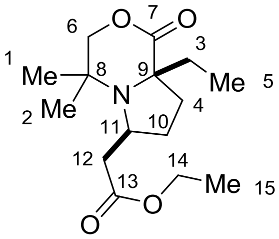

Prepared according to general procedure using 3,3-diethyl-5,5-dimethylmorpholin-2-one (18.5 mg, 0.1 mmol), ethyl acrylate (20 mg, 0.2 mmol), $Pd(OAc)_2$ (2.2 mg, 0.01 mmol), Ac-Gly-OH (2.3 mg, 0.02 mmol), AgOAc (33.4 mg, 0.2 mmol) in hexafluoroisopropanol (1 mL) at 60 ℃ for 24 hours. Then the reaction mixture was cooled to room temperature, filtered through celite, eluting with ethyl acetate, and concentrated *in vacuo*. The crude product was purified by flash column chromatography (80/20 40-60 ℃ Petroleum Ether/EtOAc) to provide the title compound as a colorless oil (24.4 mg, 86% yield, single diastereomer, *cis*). IR ν_{max}/cm^{-1} (film): 2975, 1729, 1464, 1373, 1280, 1121, 1059, 1023; ^{1}H NMR (500 MHz, $CDCl_3$) δ: 4.20-4.05 (H_6 and H_{14}, m, 3H), 3.85 (H_6, d, J = 10.8 Hz, 1H), 3.71-3.62 (H_{11}, m, 1H), 2.59 (H_{12}, dd, J = 15.0, 4.7 Hz, 1H), 2.31 (H_{12}, dd, J = 15.0, 9.6 Hz, 1H), 2.15-2.04 (H_4, m, 1H), 2.04-1.94 (H_4 and H_{10}, m, 2H), 1.75-1.58 (H_3, m, 2H), 1.58-1.45 (H_{10}, m, 1H), 1.25 (H_{15}, t, J = 7.1 Hz, 3H), 1.12 (H_1 or H_2, s, 3H), 1.08 (H_1 or H_2, s, 3H), 0.92 (H_5, t, J = 7.5 Hz, 3H); ^{13}C NMR (125 MHz, $CDCl_3$) δ: 175.0 (C_7), 171.9 (C_{13}), 77.0 (C_6), 70.5 (C_9), 60.5 (C_{14}), 57.0 (C_{11}), 53.1 (C_8), 45.0 (C_{12}), 36.2 (C_4), 35.8 (C_3), 29.5 (C_{10}), 26.1 (C_1 or C_2), 18.4 (C_1 or C_2), 14.4 (C_{15}), 9.2 (C_5); m/z HRMS (ESI) found $[M+H]^+$ 284.1858, $C_{15}H_{26}NO_4$ requires 284.1856.

2D NOESY experiment:

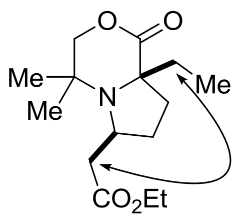

Ethyl 2-(4,4,8a-trimethyl-1-oxohexahydro-1*H*-pyrrolo[2,1-*c*][1,4]oxazin-6-yl) acetate (5a)

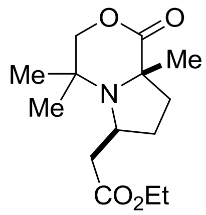

Prepared according to general procedure using 3-ethyl-3,5,5-trimethylmorpholin-2-one (34.2 mg, 0.2 mmol), ethyl acrylate (60.1 mg, 0.6 mmol), $Pd(OAc)_2$ (4.5 mg, 0.02 mmol), Ac-Gly-OH (4.7 mg, 0.04 mmol), AgOAc (100.1 mg, 0.6 mmol) in hexafluoroisopropanol (1 mL) at 80 ℃ for 36 hours. Then the reaction mixture was cooled to room temperature, filtered through celite, eluting with ethyl acetate, and concentrated *in vacuo*. The crude product was purified by flash column chromatography (80/20 40-60 ℃ Petroleum Ether/EtOAc) to provide the title compound as a colorless oil (32.8 mg, 61% yield, single diastereomer, *cis*). IR ν_{max}/cm^{-1} (film): 2977, 1729, 1467, 1371, 1281, 1124, 1031, 757; ^{1}H NMR (400 MHz, $CDCl_3$) δ: 4.18-4.10 (m, 3H), 3.93 (d, *J* = 10.8 Hz, 1H), 3.67-3.56 (m, 1H), 2.61 (dd, *J* = 14.9, 4.6 Hz, 1H), 2.34 (dd, *J* = 14.9, 9.3 Hz, 1H), 2.22-2.14 (m, 1H), 2.04-1.87 (m, 2H), 1.67-1.56 (m, 1H), 1.39 (s, 3H), 1.26 (t, *J* = 7.1 Hz, 3H), 1.12 (s, 3H), 1.07 (s, 3H); ^{13}C NMR (100 MHz, $CDCl_3$) δ: 176.1, 171.9, 76.9, 66.1, 60.5, 57.3, 53.5, 44.7, 37.3, 31.2, 29.8, 26.3, 18.4, 14.4; m/z HRMS (ESI) found $[M+H]^+$ 270.1701, $C_{14}H_{24}NO_4$ requires 270.1700.

2D NOESY experiment:

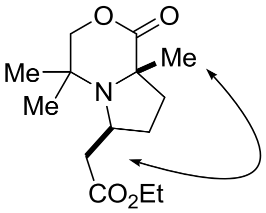

Ethyl 2-(4,4-dimethyl-1-oxo-8a-propylhexahydro-1*H*-pyrrolo[2,1-*c*][1,4] oxazin-6-yl)acetate (5c)

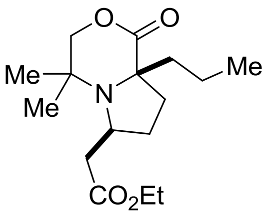

Prepared according to general procedure using 3-ethyl-5,5-dimethyl-3-propylmorpholin-2-one (39.9 mg, 0.2 mmol), ethyl acrylate (60.1 mg, 0.6 mmol), $Pd(OAc)_2$ (4.5 mg, 0.02 mmol), Ac-Gly-OH (4.7 mg, 0.04 mmol), AgOAc (100.1 mg, 0.6 mmol) in hexafluoroisopropanol (1 mL) at 80 ℃ for 36 hours. Then the reaction mixture was cooled to room temperature, filtered through celite, eluting with ethyl acetate, and concentrated *in vacuo*. The crude product was purified by flash column chromatography (80/20 40-60 ℃ Petroleum Ether/EtOAc) to provide the title compound as a colorless oil (39.8 mg, 67% yield, single diastereomer, *cis*). IR ν_{max}/cm^{-1} (film): 2966, 1729, 1466, 1374, 1281, 1125, 1058; ^{1}H NMR (400 MHz, $CDCl_3$) δ: 4.19-4.08 (m, 3H), 3.85 (d, *J* = 10.7 Hz, 1H), 3.72-3.60 (m, 1H), 2.59 (dd, *J* = 15.0, 4.7 Hz, 1H), 2.31 (dd, *J* = 15.0, 9.7 Hz, 1H), 2.17-1.93 (m, 3H), 1.65-1.48 (m, 4H), 1.30-1.20 (m, 4H), 1.11 (s, 3H), 1.07 (s, 3H), 0.89 (t, *J* = 7.2 Hz, 3H); ^{13}C NMR (100 MHz, $CDCl_3$) δ: 175.2, 171.9, 77.0, 70.0, 60.5, 57.0, 53.1, 45.5, 45.1, 36.7, 29.6, 26.1, 18.4, 18.1, 14.4, 14.3; m/z HRMS (ESI) found $[M+Na]^+$ 320.1833, $C_{16}H_{27}NNaO_4$ requires 320.1832.

2D NOESY experiment:

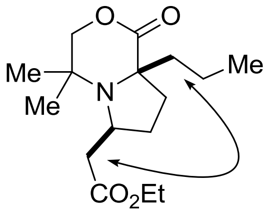

Ethyl 2-(4,4-dimethyl-1-oxo-8a-((triisopropylsilyloxy)methyl)hexahydro-1*H*-pyrrolo[2,1-*c*][1,4]oxazin-6-yl)acetate (5d)

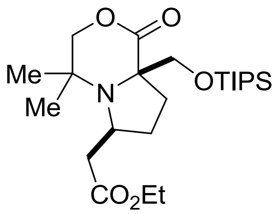

Prepared according to general procedure using 3-ethyl-5,5-dimethyl-3-((triisopropylsilyloxy)methyl)morpholin-2-one (68.7 mg, 0.2 mmol), ethyl acrylate (60.1 mg, 0.6 mmol), $Pd(OAc)_2$ (4.5 mg, 0.02 mmol), Ac-Gly-OH (4.7 mg, 0.04 mmol), AgOAc (100.1 mg, 0.6 mmol) in hexafluoroisopropanol (1 mL) at 80 ℃ for 36 hours. Then the reaction mixture was cooled to room temperature, filtered through celite, eluting with ethyl acetate, and concentrated *in vacuo*. The crude product was purified by flash column chromatography (80/20 40-60 ℃ Petroleum Ether/EtOAc) to provide the title compound as a pale oil (68.9 mg, 78% yield, single diastereomer, *cis*). IR ν_{max}/cm^{-1} (film): 2944, 1733, 1464, 1384, 1288, 1179, 1108, 882, 798, 751, 683; ^1H NMR (500 MHz, $CDCl_3$) δ: 4.47 (d, *J* = 10.3 Hz, 1H), 4.12 (q, *J* = 7.1 Hz, 2H), 3.81-3.69 (m, 3H), 3.57 (d, *J* = 9.0 Hz, 1H), 2.62 (dd, *J* = 15.0, 4.6 Hz, 1H), 2.28 (dd, *J* = 15.0, 9.8 Hz, 1H), 2.20-2.12 (m, 1H), 2.02-1.88 (m, 2H), 1.48-1.37 (m, 1H), 1.25 (t, *J* = 7.1 Hz, 3H), 1.15-0.94 (m, 27H); ^{13}C NMR (125 MHz, $CDCl_3$) δ: 174.6, 171.9, 76.9, 72.7, 71.9, 60.5, 57.5, 53.0, 44.9, 34.0, 29.8, 26.1, 18.7, 18.08, 18.06, 14.4, 12.0; m/z HRMS (ESI) found $[M+H]^+$ 464.2790, $C_{23}H_{43}NNaO_5Si$ requires 464.2803.

2D NOESY experiment:

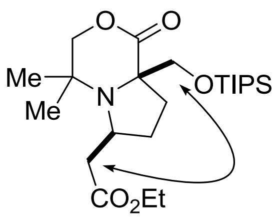

Ethyl 2-(8a-(acetoxymethyl)-4,4-dimethyl-1-oxohexahydro-1*H*-pyrrolo[2,1-*c*][1,4]oxazin-6-yl)acetate (5e)

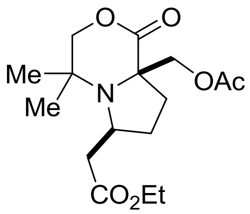

Prepared according to general procedure using (3-ethyl-5,5-dimethyl-2-oxomorpholin-3-yl)methyl acetate (45.9 mg, 0.2 mmol), ethyl acrylate (60.1 mg, 0.6 mmol), $Pd(OAc)_2$ (4.5 mg, 0.02 mmol), Ac-Gly-OH (4.7 mg, 0.04 mmol), AgOAc (100.1 mg, 0.6 mmol) in hexafluoroisopropanol (1 mL) at 80 ℃ for 36 hours. Then the reaction mixture was cooled to room temperature, filtered through celite, eluting with ethyl acetate, and concentrated *in vacuo*. The crude product was purified by flash column chromatography (70/30 40-60 ℃ Petroleum Ether/EtOAc) to provide the title compound as a colorless oil (47.1 mg, 72% yield, single diastereomer, *cis*). IR ν_{max}/cm^{-1} (film): 2975, 1730, 1468, 1375, 1218, 1037, 734; ^1H NMR (500 MHz, $CDCl_3$) δ: 4.25 (d, *J* = 10.9 Hz, 1H), 4.20 (d, *J* = 10.9 Hz, 1H), 4.10 (q, *J* = 7.1 Hz, 2H), 3.90 (dd, *J* = 10.9, 6.6 Hz, 2H), 3.76-3.67 (m, 1H), 2.58 (dd, *J* = 15.3, 4.5 Hz, 1H), 2.35 (dd, *J* = 15.3, 9.7 Hz, 1H), 2.17-2.01 (m, 6H), 1.60-1.47 (m, 1H), 1.24 (t, *J* = 7.1 Hz, 3H), 1.12 (s, 3H), 1.10 (s, 3H); ^{13}C NMR (125 MHz, $CDCl_3$) δ: 173.2, 171.7, 170.5, 77.1, 69.5, 69.0, 60.5, 57.3, 53.2, 44.4, 34.0, 29.6, 26.1, 21.0, 18.7, 14.3; m/z HRMS (ESI) found $[M+H]^+$ 350.1571, $C_{16}H_{25}NNaO_6$ requires 350.1574.

2D NOESY experiment:

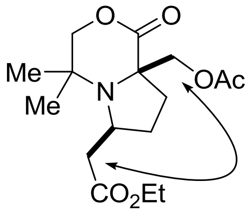

(6-(2-Ethoxy-2-oxoethyl)-4,4-dimethyl-1-oxohexahydro-1*H*-pyrrolo[2,1-*c*][1,4]oxazin-8a-yl)methyl acrylate (5f)

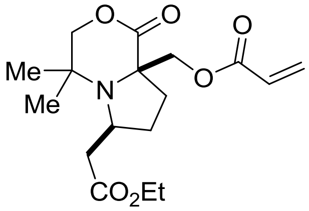

Prepared according to general procedure using (3-ethyl-5,5-dimethyl-2-oxomorpholin-3-yl)methyl acrylate (24.1 mg, 0.1 mmol), ethyl acrylate (30.1 mg, 0.3 mmol), $Pd(OAc)_2$ (2.2 mg, 0.01 mmol), Ac-Gly-OH (2.3 mg, 0.02 mmol), AgOAc (50.1 mg, 0.3 mmol) in hexafluoroisopropanol (1 mL) at 80 ℃ for 36 hours. Then the reaction mixture was cooled to room temperature, filtered through celite, eluting with ethyl acetate, and concentrated *in vacuo*. The crude product was purified by flash column chromatography (70/30 40-60 ℃ Petroleum Ether/EtOAc) to provide the title compound as a colorless oil (16.3 mg, 48% yield, single diastereomer, *cis*). IR ν_{max}/cm^{-1} (film): 2933, 1731, 1469, 1289, 1180, 985; ^1H NMR (400 MHz, $CDCl_3$) δ: 6.44 (dd, *J* = 17.3, 1.3 Hz, 1H), 6.14 (dd, *J* = 17.3, 10.4 Hz, 1H), 5.88 (dd, *J* = 10.4, 1.3 Hz, 1H), 4.34 (d, *J* = 11.0 Hz, 1H), 4.26 (d, *J* = 10.7 Hz, 1H), 4.12 (q, *J* = 7.1 Hz, 2H), 4.02 (d, *J* = 11.0 Hz, 1H), 3.91 (d, *J* = 10.7 Hz, 1H), 3.81-3.65 (m, 1H), 2.59 (dd, *J* = 15.4, 4.4 Hz, 1H), 2.40 (dd, *J* = 15.3, 9.8 Hz, 1H), 2.18-2.07 (m, 3H), 1.59-1.51 (m, 1H), 1.25 (t, *J* = 7.1 Hz, 3H), 1.12 (s, 3H), 1.12 (s, 3H); ^{13}C NMR (100 MHz, $CDCl_3$) δ: 173.2, 171.8, 165.6, 131.9, 128.1, 77.4, 69.5, 69.1, 60.6, 57.4, 53.2, 44.5, 34.1, 29.7, 26.0, 18.6, 14.4; m/z HRMS (ESI) found $[M+H]^+$ 340.1755, $C_{17}H_{26}NO_6$ requires 340.1755.

2D NOESY experiment:

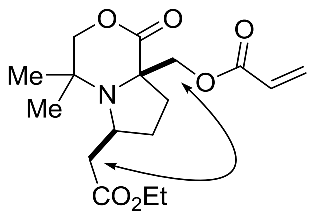

Ethyl 4-(6-(2-ethoxy-2-oxoethyl)-4,4-dimethyl-1-oxohexahydro-1*H*-pyrrolo [2,1-*c*][1,4]oxazin-8a-yl)butanoate (5g)

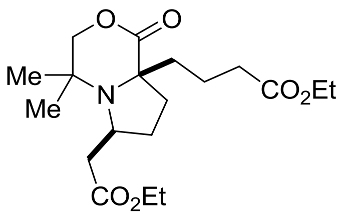

Prepared according to general procedure using ethyl 4-(3-ethyl-5,5-dimethyl-2-oxomorpholin-3-yl)butanoate (54.2 mg, 0.2 mmol), ethyl acrylate (60.1 mg, 0.6 mmol), $Pd(OAc)_2$ (4.5 mg, 0.02 mmol), Ac-Gly-OH (4.7 mg, 0.04 mmol), AgOAc (100.1 mg, 0.6 mmol) in hexafluoroisopropanol (1 mL) at 80 ℃ for 24 hours. Then the reaction mixture was cooled to room temperature, filtered through celite, eluting with ethyl acetate, and concentrated *in vacuo*. The crude product was purified by flash column chromatography (65/35 40-60 ^{o}C Petroleum Ether/EtOAc) to provide the title compound as a colorless oil (59.8 mg, 81% yield, single diastereomer, *cis*). IR ν_{max}/cm^{-1} (film): 2976, 1727, 1465, 1373, 1281, 1154, 1111, 1057; ^{1}H NMR (500 MHz, $CDCl_3$) δ: 4.16 (d, *J* = 10.8 Hz, 1H), 4.14-4.05 (m, 4H), 3.85 (d, *J* = 10.8 Hz, 1H), 3.70-3.59 (m, 1H), 2.58 (dd, *J* = 15.1, 4.6 Hz, 1H), 2.35-2.22 (m, 3H), 2.14-2.06 (m, 1H), 2.05-1.95 (m, 2H), 1.86-1.77 (m, 1H), 1.69-1.57 (m, 2H), 1.57-1.46 (m, 2H), 1.28-1.20 (m, 6H), 1.10 (s, 3H), 1.06 (s, 3H); ^{13}C NMR (125 MHz, $CDCl_3$) δ: 174.8, 173.2, 171.8, 77.0, 69.7, 60.5, 60.4, 57.0, 53.1, 45.0, 42.1, 36.6, 34.1, 29.5, 26.0, 20.3, 18.4, 14.34, 14.32; m/z HRMS (ESI) found $[M+H]^+$ 370.2223, $C_{19}H_{32}NO_6$ requires 370.2224.

2D NOESY experiment:

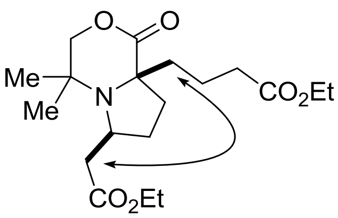

Ethyl 2-(8a-(3-(1,3-dioxoisoindolin-2-yl)propyl)-4,4-dimethyl-1-oxohexahydro-1*H*-pyrrolo[2,1-*c*][1,4]oxazin-6-yl)acetate (5h)

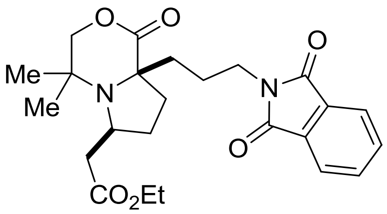

Prepared according to general procedure using 2-(3-(3-ethyl-5,5-dimethyl-2-oxomorpholin-3-yl)propyl)isoindoline-1,3-dione (34.4 mg, 0.1 mmol), ethyl acrylate (30.1 mg, 0.3 mmol), $Pd(OAc)_2$ (2.2 mg, 0.01 mmol), Ac-Gly-OH (2.3 mg, 0.02 mmol), AgOAc (50.1 mg, 0.3 mmol) in hexafluoroisopropanol (1 mL) at 80 ℃ for 36 hours. Then the reaction mixture was cooled to room temperature, filtered through celite, eluting with ethyl acetate, and concentrated *in vacuo*. The crude product was purified by flash column chromatography (50/30 40-60 ^{o}C Petroleum Ether/EtOAc) to provide the title compound as a colorless oil (26.4 mg, 60% yield, single diastereomer, *cis*). IR ν_{max}/cm^{-1} (film): 2973, 1709, 1396, 1284, 1179, 1066, 721; ^{1}H NMR (500 MHz, $CDCl_3$) δ: 7.85-7.81 (m, 2H), 7.74-7.67 (m, 2H), 4.19 (d, *J* = 10.8 Hz, 1H), 4.09 (q, *J* = 7.1 Hz, 2H), 3.86 (d, *J* = 10.8 Hz, 1H), 3.75-3.61 (m, 3H), 2.56 (dd, *J* = 15.1, 4.6 Hz, 1H), 2.25 (dd, *J* = 15.1, 9.8 Hz, 1H), 2.14-2.06 (m, 1H), 2.06-1.94 (m, 2H), 1.91-1.81 (m, 1H), 1.79-1.71 (m, 1H), 1.69-1.63 (m, 2H), 1.57-1.47 (m, 1H), 1.24 (t, *J* = 7.1 Hz, 3H), 1.11 (s, 3H), 1.07 (s, 3H); ^{13}C NMR (125 MHz, $CDCl_3$) δ: 174.6, 171.6, 168.5, 134.1, 132.2, 123.4, 77.0, 69.7, 60.5, 57.1, 53.2, 45.0, 40.0, 38.0, 36.8, 29.5, 26.0, 24.3, 18.5, 14.4; m/z HRMS (ESI) found $[M+H]^{+}$ 443.2173, $C_{24}H_{31}N_2O_6$ requires 443.2177.

2D NOESY experiment:

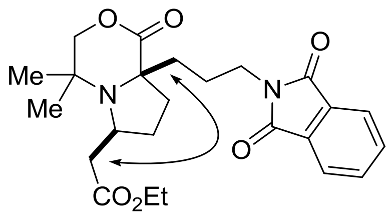

Ethyl 2-(4,4-dimethyl-1-oxo-8a-(3-tosylpropyl)hexahydro-1H-pyrrolo[2,1-*c*][1,4]oxazin-6-yl)acetate (5i)

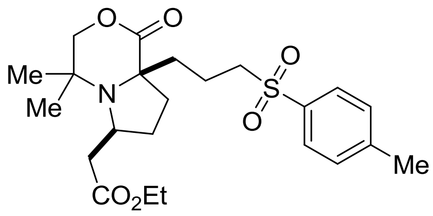

Prepared according to general procedure using 3-ethyl-5,5-dimethyl-3-(3-tosylpropyl)morpholin-2-one (35.3 mg, 0.1 mmol), ethyl acrylate (30.1 mg, 0.3 mmol), $Pd(OAc)_2$ (2.2 mg, 0.01 mmol), Ac-Gly-OH (2.3 mg, 0.02 mmol), AgOAc (50.1 mg, 0.3 mmol) in hexafluoroisopropanol (1 mL) at 80 ℃ for 36 hours. Then the reaction mixture was cooled to room temperature, filtered through celite, eluting with ethyl acetate, and concentrated *in vacuo*. The crude product was purified by flash column chromatography (50/50 40-60 ℃ Petroleum Ether/EtOAc) to provide the title compound as an amorphous solid (25.3 mg, 56% yield, single diastereomer, *cis*). IR ν_{max}/cm^{-1} (film): 2934, 1731, 1464, 1285, 1142, 820, 722; ^1H NMR (500 MHz, $CDCl_3$) δ: 7.77 (d, *J* = 8.1 Hz, 2H), 7.36 (d, *J* = 8.1 Hz, 2H), 4.20-4.04 (m, 3H), 3.86 (d, *J* = 10.9 Hz, 1H), 3.71-3.60 (m, 1H), 3.05 (t, *J* = 7.4 Hz, 2H), 2.56 (dd, *J* = 15.1, 4.5 Hz, 1H), 2.45 (s, 3H), 2.28 (dd, *J* = 15.1, 9.8 Hz, 1H), 2.14-1.99 (m, 2H), 1.99-1.86 (m, 2H), 1.82-1.63 (m, 3H), 1.57-1.43 (m, 1H), 1.27 (t, *J* = 7.1 Hz, 3H), 1.11 (s, 3H), 1.07 (s, 3H); ^{13}C NMR (125 MHz, $CDCl_3$) δ: 174.4, 171.7, 144.9, 136.2, 130.1, 128.2, 77.1, 69.6, 60.6, 57.1, 56.1, 53.2, 44.9, 41.0, 36.7, 29.5, 26.0, 21.8, 18.6, 18.4, 14.4; m/z HRMS (ESI) found $[M+H]^+$ 452.2098, $C_{23}H_{34}NO_6S$ requires 452.2101.

2D NOESY experiment:

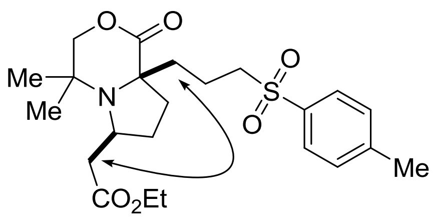

Ethyl 2-(4,4-dimethyl-1-oxo-8a-(4-(4,4,5,5-tetramethyl-1,3,2-dioxaborolan-2-yl) butyl)hexahydro-1*H*-pyrrolo[2,1-*c*][1,4]oxazin-6-yl)acetate (5j)

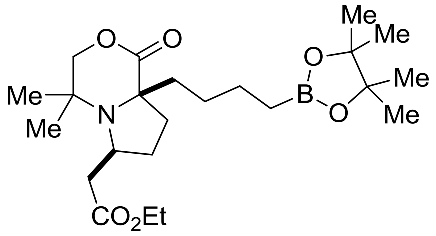

Prepared according to general procedure using 3-ethyl-5,5-dimethyl-3-(4-(4,4,5,5-tetramethyl-1,3,2-dioxaborolan-2-yl)butyl)morpholin-2-one (33.9 mg, 0.1 mmol), ethyl acrylate (30.1 mg, 0.3 mmol), Pd(OAc)$_2$ (2.2 mg, 0.01 mmol), Ac-Gly-OH (2.3 mg, 0.02 mmol), AgOAc (50.1 mg, 0.3 mmol) in hexafluoroisopropanol (1 mL) at 80 ℃ for 36 hours. Then the reaction mixture was cooled to room temperature, filtered through celite, eluting with ethyl acetate, and concentrated *in vacuo*. The crude product was purified by flash column chromatography (80/20 40-60 ℃ Petroleum Ether/EtOAc) to provide the title compound as a colorless oil (30.6 mg, 70% yield, single diastereomer, *cis*). IR ν_{max}/cm^{-1} (film): 2977, 1732, 1465, 1372, 1144, 1066, 915, 729; ^{1}H NMR (500 MHz, CDCl$_3$) δ: 4.17 (d, *J* = 10.8 Hz, 1H), 4.12 (q, *J* = 7.1 Hz, 2H), 3.83 (d, *J* = 10.8 Hz, 1H), 3.69-3.60 (m, 1H), 2.58 (dd, *J* = 15.1, 4.6 Hz, 1H), 2.30 (dd, *J* = 15.1, 9.9 Hz, 1H), 2.14-2.05 (m, 1H), 2.05-1.92 (m, 2H), 1.64-1.57 (m, 2H), 1.54-1.44 (m, 2H), 1.42-1.34 (m, 2H), 1.26 (t, *J* = 7.1 Hz, 3H), 1.24-1.20 (m, 1H), 1.23 (s, 12H), 1.10 (s, 3H), 1.06 (s, 3H), 0.77 (t, *J* = 7.7 Hz, 2H); ^{13}C NMR (125 MHz, CDCl$_3$) δ: 175.1, 171.9, 83.1, 77.0, 69.9, 60.5, 57.0, 53.1, 45.2, 43.0, 36.7, 29.6, 27.3, 26.1, 25.0, 24.3, 18.4, 14.4, 11.2 (br); ^{11}B NMR (128 MHz, CDCl$_3$) δ: 33.7; m/z HRMS (ESI) found $[M+H]^+$ 437.3057, $C_{23}H_{41}{}^{10}BNO_6$ requires 437.3058.

2D NOESY experiment:

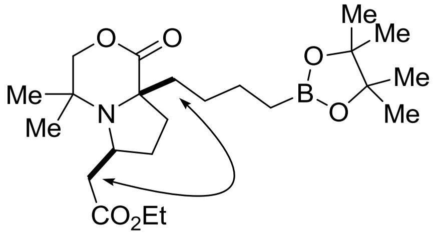

Ethyl 2-(8a'-ethyl-1'-oxohexahydrospiro[cyclohexane-1,4'-pyrrolo[2,1-*c*][1,4]oxazine]-6'-yl)acetate (5k)

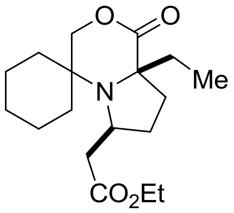

Prepared according to general procedure using 2,2-diethyl-4-oxa-1-azaspiro[5.5]undecan-3-one (45.1 mg, 0.2 mmol), ethyl acrylate (60.1 mg, 0.6 mmol), $Pd(OAc)_2$ (4.5 mg, 0.02 mmol), Ac-Gly-OH (4.7 mg, 0.04 mmol), AgOAc (100.1 mg, 0.6 mmol) in hexafluoroisopropanol (1 mL) at 80 ℃ for 36 hours. Then the reaction mixture was cooled to room temperature, filtered through celite, eluting with ethyl acetate, and concentrated *in vacuo*. The crude product was purified by flash column chromatography (80/20 40-60 ℃ Petroleum Ether/EtOAc) to provide the title compound as a colorless oil (48.5 mg, 75% yield, single diastereomer, *cis*). IR ν_{max}/cm^{-1} (film): 2934, 1729, 1456, 1373, 1286, 1187, 1120, 1057, 971; ^{1}H NMR (400 MHz, $CDCl_3$) δ: 4.48 (d, *J* = 11.1 Hz, 1H), 4.12 (q, *J* = 7.1 Hz, 2H), 3.98 (d, *J* = 11.1 Hz, 1H), 3.89-3.77 (m, 1H), 2.54 (dd, *J* = 15.1, 4.7 Hz, 1H), 2.31 (dd, *J* = 15.1, 9.9 Hz, 1H), 2.13-1.92 (m, 3H), 1.74-1.61 (m, 6H), 1.56-1.45 (m, 4H), 1.37-1.23 (m, 5H), 1.20-1.11 (m, 1H), 0.93 (t, *J* = 7.5 Hz, 3H); ^{13}C NMR (100 MHz, $CDCl_3$) δ: 175.2, 172.0, 71.7, 70.4, 60.5, 55.6, 55.3, 45.6, 36.9, 35.7, 34.8, 29.6, 26.0, 25.6, 23.00, 22.98, 14.4, 9.2; m/z HRMS (ESI) found $[M+Na]^+$ 346.1989, $C_{18}H_{29}NNaO_4$ requires 346.1989.

2D NOESY experiment:

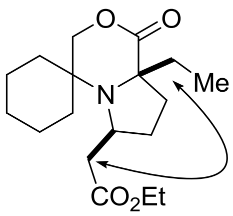

Ethyl 2-(8a-ethyl-4-methyl-1-oxohexahydro-1*H*-pyrrolo[2,1-*c*][1,4]oxazin-6-yl) acetate (5l)

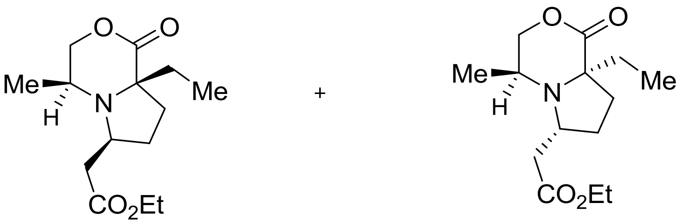

Prepared according to general procedure using (*S*)-3,3-diethyl-5-methylmorpholin-2-one (34.2 mg, 0.2 mmol), ethyl acrylate (60.1 mg, 0.6 mmol), $Pd(OAc)_2$ (4.5 mg, 0.02 mmol), Ac-Gly-OH (4.7 mg, 0.04 mmol), AgOAc (100.1 mg, 0.6 mmol) in hexafluoroisopropanol (1 mL) at 80 ℃ for 36 hours. Then the reaction mixture was cooled to room temperature, filtered through celite, eluting with ethyl acetate, and concentrated *in vacuo*. The crude products were purified by flash column chromatography to provide the title compounds.

Ethyl 2-((4*S*,6*S*,8a*S*)-8a-ethyl-4-methyl-1-oxohexahydro-1*H*-pyrrolo [2,1-*c*][1,4]oxazin-6-yl)acetate (5l[1])

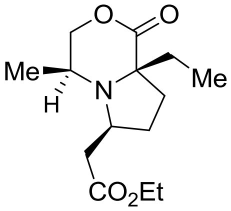

Isolated by flash column chromatography (80/20 40-60 ℃ Petroleum Ether/EtOAc) provided the title compound as a colorless oil (27.5 mg (pure) + 5.4 mg (mixed with another isomer), 61% yield). IR ν_{max}/cm^{-1} (film): 2975, 1729, 1462, 1335, 1183, 1060, 1031; ^{1}H NMR (400 MHz, $CDCl_3$) δ: 4.19-3.97 (m, 4H), 3.42-3.30 (m, 1H), 2.97-2.85 (m, 1H), 2.57 (dd, *J* = 15.0, 4.8 Hz, 1H), 2.38-2.25 (m, 2H), 2.00-1.82 (m, 2H), 1.72-1.61 (m, 2H), 1.61-1.48 (m, 1H), 1.24 (t, *J* = 7.1 Hz, 3H), 1.09 (d, *J* = 6.3 Hz,

3H), 0.92 (t, *J* = 7.4 Hz, 3H); ^{13}C NMR (100 MHz, $CDCl_3$) δ: 174.3, 171.8, 71.1, 71.0, 63.7, 60.5, 54.2, 43.3, 34.9, 34.7, 29.2, 18.4, 14.3, 9.0; m/z HRMS (ESI) found $[M+H]^+$ 270.1700, $C_{14}H_{24}NO_4$ requires 270.1700.

2D NOESY experiment:

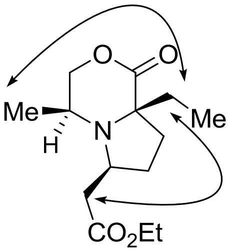

Ethyl 2-((4*S*,6*R*,8a*R*)-8a-ethyl-4-methyl-1-oxohexahydro-1*H*-pyrrolo [2,1-*c*][1,4]oxazin-6-yl)acetate (5l[2])

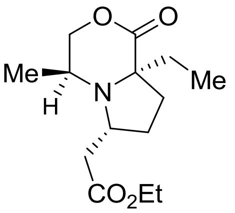

Isolated by flash column chromatography (80/20 40-60 ℃ Petroleum Ether/EtOAc) provided the title compound as a colorless oil (7.1 mg (mixed with another isomer), 13% yield). IR ν_{max}/cm^{-1} (film): 2975, 1729, 1462, 1335, 1183, 1060, 1031; ^{1}H NMR (500 MHz, $CDCl_3$) δ: 4.30 (app d, *J* = 5.6 Hz, 2H), 4.15-4.11 (m, 2H), 3.69-3.62 (m, 1H), 3.50-3.41 (m, 1H), 2.44 (dd, *J* = 15.1, 4.5 Hz, 1H), 2.33-2.28 (m, 1H), 2.11-2.03 (m, 1H), 2.02-1.93 (m, 2H), 1.79-1.70 (m, 2H), 1.65-1.61 (m, 1H), 1.26 (t, *J* = 7.1 Hz, 3H), 1.14 (d, *J* = 6.9 Hz, 3H), 0.95 (t, *J* = 7.4 Hz, 3H); ^{13}C NMR (125 MHz, $CDCl_3$) δ: 174.9, 172.0, 71.4, 71.0, 63.8, 60.5, 54.9, 47.6, 33.7, 31.3, 29.5, 18.5, 14.8, 8.6; m/z HRMS (ESI) found $[M+H]^+$ 270.1700, $C_{14}H_{24}NO_4$ requires 270.1700.

2D NOESY experiment:

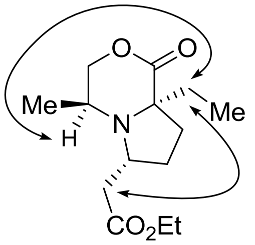

Ethyl 2-(8a-ethyl-4-isopropyl-1-oxohexahydro-1*H*-pyrrolo[2,1-*c*][1,4]oxazin-6-yl) acetate (5m)

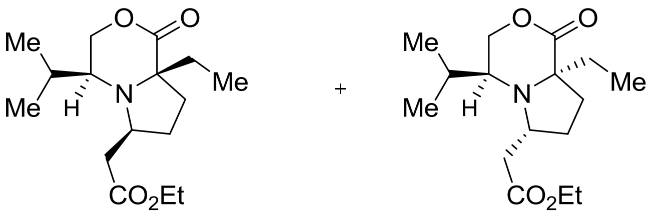

Prepared according to general procedure using (*S*)-3,3-diethyl-5-isopropylmorpholin-2-one (39.9 mg, 0.2 mmol), ethyl acrylate (60.1 mg, 0.6 mmol), $Pd(OAc)_2$ (4.5 mg, 0.02 mmol), Ac-Gly-OH (4.7 mg, 0.04 mmol), AgOAc (100.1 mg, 0.6 mmol) in hexafluoroisopropanol (1 mL) at 80 ℃ for 36 hours. Then the reaction mixture was cooled to room temperature, filtered through celite, eluting with ethyl acetate, and concentrated *in vacuo*. The crude products were purified by flash column chromatography to provide the title compounds.

Ethyl 2-((4*S*,6*S*,8a*S*)-8a-ethyl-4-isopropyl-1-oxohexahydro-1*H*-pyrrolo[2,1-*c*][1,4]oxazin-6-yl)acetate (5m^1)

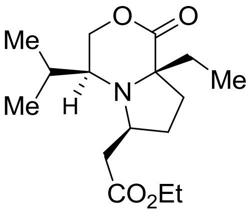

Isolated by flash column chromatography (80/20 40-60 ℃ Petroleum Ether/EtOAc) provided the title compound as a colorless oil (15.5 mg (pure) + 3.8 mg (mixed with another isomer), 33% yield). IR ν_{max}/cm^{-1} (film): 2963, 1730, 1465, 1368, 1300, 1179, 1055; ^{1}H NMR (500 MHz, $CDCl_3$) δ: 4.29-4.20 (m, 2H), 4.11 (q, *J* = 7.1 Hz, 2H), 3.39-3.30 (m, 1H), 2.60-2.49 (m, 2H), 2.42-2.31 (m, 2H), 1.96-1.80 (m, 3H), 1.79-1.66 (m, 2H), 1.62-1.54 (m, 1H), 1.25 (t, *J* = 7.1 Hz, 3H), 1.00-0.90 (m, 9H); ^{13}C NMR (125 MHz, $CDCl_3$) δ: 174.1, 171.9, 70.2, 65.4, 64.4, 64.1, 60.6, 42.3, 35.1, 34.4, 29.5, 29.3, 19.7, 17.1, 14.3, 9.0; m/z HRMS (ESI) found [M+H]$^+$ 298.2012, $C_{16}H_{28}NO_4$ requires 298.2013.

2D NOESY experiment:

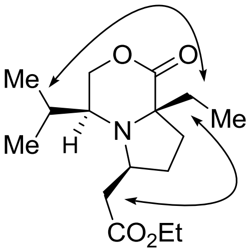

Ethyl 2-((4*S*,6*R*,8a*R*)-8a-ethyl-4-isopropyl-1-oxohexahydro-1*H*-pyrrolo [2,1-*c*][1,4]oxazin-6-yl)acetate (5m²)

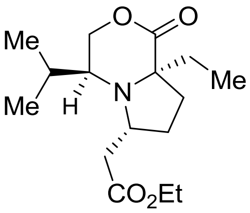

Isolated by flash column chromatography (80/20 40-60 ℃ Petroleum Ether/EtOAc) provided the title compound as a colorless oil (2.8 mg (mixed with another isomer), 5% yield). IR ν_{max}/cm^{-1} (film): 2963, 1730, 1465, 1368, 1300, 1179, 1055; ^{1}H NMR (500 MHz, $CDCl_3$) δ: 4.50 (app t, *J* = 11.5 Hz, 1H), 4.37 (dd, *J* = 11.5, 3.6 Hz, 1H), 4.12 (q, *J* = 7.1 Hz, 2H), 3.73-3.66 (m, 1H), 2.84-2.77 (m, 1H), 2.49 (dd, *J* = 15.4, 3.4 Hz, 1H), 2.32 (dd, *J* = 11.5, 6.7 Hz, 1H), 2.13-2.07 (m, 1H), 1.97-1.92 (m, 1H), 1.92-1.54 (m, 5H), 1.26 (t, *J* = 7.1 Hz, 3H), 1.11 (d, *J* = 6.4 Hz, 3H), 0.98-0.88 (m, 6H); ^{13}C NMR (125 MHz, $CDCl_3$) δ: 175.4, 172.0, 72.3, 68.7, 60.6, 57.8, 54.0, 42.8, 32.4, 29.5, 28.5, 27.6, 21.4, 19.5, 14.4, 7.8; m/z HRMS (ESI) found $[M+H]^+$ 298.2012, $C_{16}H_{28}NO_4$ requires 298.2013.

2D NOESY experiment:

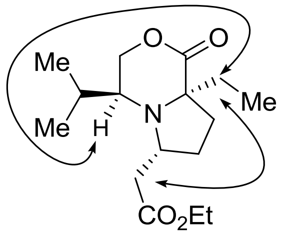

(5n)

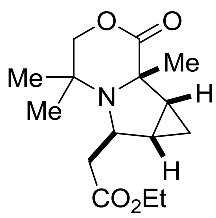

Prepared according to general procedure using 3-cyclopropyl-3,5,5-trimethylmorpholin-2-one (36.6 mg, 0.2 mmol), ethyl acrylate (60.1 mg, 0.6 mmol), Pd(OAc)$_2$ (4.5 mg, 0.02 mmol), Ac-Gly-OH (4.7 mg, 0.04 mmol), AgOAc (100.1 mg, 0.6 mmol) in hexafluoroisopropanol (1 mL) at 80 ℃ for 36 hours. Then the reaction mixture was cooled to room temperature, filtered through celite, eluting with ethyl acetate, and concentrated *in vacuo*. The crude product was purified by flash column chromatography (80/20 40-60 ℃ Petroleum Ether/EtOAc) to provide the title compound as an amorphous solid (14.6 mg, 26% yield). IR ν_{max}/cm^{-1} (film): 2977, 1727, 1465, 1370, 1283, 1177, 1095, 1045; ^{1}H NMR (500 MHz, CDCl$_3$) δ: 4.24-4.18 (m, 1H), 4.13 (q, *J* = 7.1 Hz, 2H), 4.06 (d, *J* = 11.7 Hz, 1H), 3.87-3.80 (m, 1H), 2.57 (dd, *J* = 15.4, 3.5 Hz, 1H), 2.34 (dd, *J* = 15.4, 10.0 Hz, 1H), 2.27-2.20 (m, 1H), 1.82-1.72 (m, 1H), 1.47 (s, 3H), 1.25 (t, *J* = 7.1 Hz, 3H), 1.23 (s, 3H), 1.12 (s, 3H), 0.54-0.46 (m, 2H); ^{13}C NMR (125 MHz, CDCl$_3$) δ: 175.0, 171.7, 74.6, 65.4, 60.5, 58.8, 52.5, 41.7, 28.4, 27.5, 27.2, 23.4, 21.8, 14.4, 5.9; m/z HRMS (ESI) found [M+H]$^+$ 282.1694, $C_{15}H_{24}NO_4$ requires 282.1700.

2D NOESY experiment:

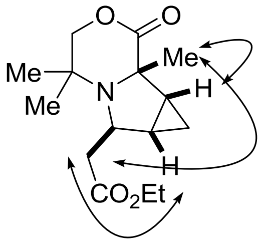

Ethyl 2-(11a-ethyl-4,4-dimethyl-1-oxo-1,3,4,6,11,11a-hexahydro-[1,4]oxazino[4,3-*b*]isoquinolin-6-yl)acetate (5o^1) and (*E*)-ethyl 3-(6-(2-ethoxy-2-oxoethyl)-11a-ethyl-4,4-dimethyl-1-oxo-1,3,4,6,11,11a-hexahydro-[1,4]oxazino[4,3-*b*]isoquinolin-10-yl)acrylate (5o^2)

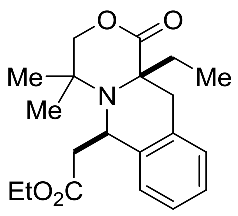 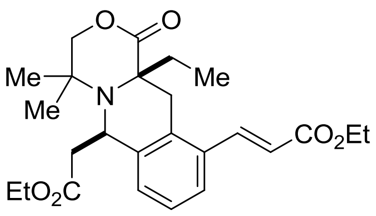

Prepared according to general procedure using 3-benzyl-3-ethyl-5,5-dimethylmorpholin-2-one (49.5 mg, 0.2 mmol), ethyl acrylate (60.1 mg, 0.6 mmol), $Pd(OAc)_2$ (4.5 mg, 0.02 mmol), Ac-Gly-OH (4.7 mg, 0.04 mmol), AgOAc (100.1 mg, 0.6 mmol) in hexafluoroisopropanol (1 mL) at 80 ℃ for 36 hours. Then the reaction mixture was cooled to room temperature, filtered through celite, eluting with ethyl acetate, and concentrated *in vacuo*. The crude products were purified by flash column chromatography to provide the title compounds.

Ethyl 2-(11a-ethyl-4,4-dimethyl-1-oxo-1,3,4,6,11,11a-hexahydro-[1,4] oxazino[4,3-*b*]isoquinolin-6-yl)acetate (5o[1])

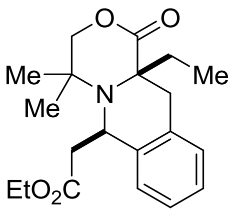

Isolated by flash column chromatography (85/15 40-60 ℃ Petroleum Ether/EtOAc) provided the title compound as an amorphous solid (24.8 mg, 36% yield, single diastereomer, *cis*). IR ν_{max}/cm^{-1} (film): 2975, 1730, 1461, 1371, 1281, 1143, 1064, 760; ^1H NMR (500 MHz, $CDCl_3$) δ: 7.26-7.08 (m, 4H), 4.57 (t, *J* = 7.5 Hz, 1H), 4.34 (d, *J* = 11.2 Hz, 1H), 4.20 (dq, *J* = 10.8, 7.1 Hz, 1H), 4.10 (dq, *J* = 10.8, 7.1 Hz, 1H), 3.78 (d, *J* = 11.2 Hz, 1H), 3.72 (d, *J* = 15.7 Hz, 1H), 2.78 (dd, *J* = 14.9, 8.0 Hz, 1H), 2.65 (d, *J* = 15.7 Hz, 1H), 2.57 (dd, *J* = 14.9, 7.0 Hz, 1H), 1.99-1.80 (m, 2H), 1.26 (t, *J* = 7.1 Hz, 3H), 1.24 (s, 3H), 0.99 (t, *J* = 7.5 Hz, 3H), 0.74 (s, 3H); ^{13}C NMR (125 MHz, $CDCl_3$) δ: 173.0, 171.7, 140.5, 133.6, 128.8, 127.4, 126.9, 125.5, 75.5, 64.5, 60.7, 55.3, 54.4, 43.5, 38.0, 35.3, 26.1, 21.9, 14.4, 8.8; m/z HRMS (ESI) found $[M+Na]^+$ 368.1833, $C_{20}H_{27}NNaO_4$ requires 368.1832.

2D NOESY experiment:

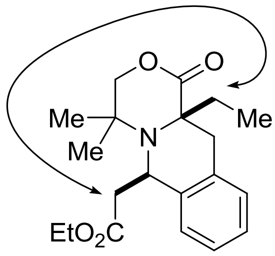

(*E*)-ethyl 3-(6-(2-ethoxy-2-oxoethyl)-11a-ethyl-4,4-dimethyl-1-oxo-1,3,4,6,11,11a-hexahydro-[1,4]oxazino[4,3-*b*]isoquinolin-10-yl)acrylate (5o[2])

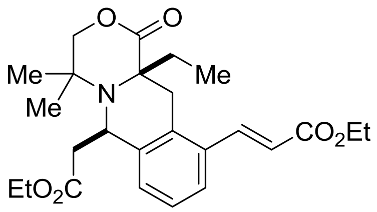

Isolated by flash column chromatography (70/30 40-60 ℃ Petroleum Ether/EtOAc) provided the title compound as a colorless oil (48.8 mg, 55% yield, single diastereomer, *cis*). IR ν_{max}/cm^{-1} (film): 2976, 1711, 1634, 1461, 1369, 1280, 1161, 1036, 804, 735; ^{1}H NMR (500 MHz, $CDCl_3$) δ: 8.17 (d, *J* = 15.9 Hz, 1H), 7.47-7.40 (m, 1H), 7.20-7.12 (m, 2H), 6.35 (d, *J* = 15.9 Hz, 1H), 4.58 (t, *J* = 7.5 Hz, 1H), 4.35 (d, *J* = 11.3 Hz, 1H), 4.27 (q, *J* = 7.1 Hz, 2H), 4.19 (dq, *J* = 10.8, 7.1 Hz, 1H), 4.09 (dq, *J* = 10.8, 7.1 Hz, 1H), 4.00 (d, *J* = 15.9 Hz, 1H), 3.78 (d, *J* = 11.3 Hz, 1H), 2.79 (dd, *J* = 14.9, 7.7 Hz, 1H), 2.56 (dd, *J* = 14.9, 7.2 Hz, 1H), 2.54-2.43 (m, 1H), 1.92 (q, *J* = 7.5 Hz, 2H), 1.34 (t, *J* = 7.1 Hz, 3H), 1.29-1.19 (m, 6H), 1.02 (t, *J* = 7.5 Hz, 3H), 0.75 (s, 3H); ^{13}C NMR (125 MHz, $CDCl_3$) δ: 172.7, 171.6, 166.9, 141.6, 141.3, 133.7, 133.2, 127.3, 126.8, 125.7, 121.0, 75.3, 64.7, 60.8, 60.6, 55.4, 54.5, 43.4, 37.8, 31.0, 26.1, 22.0, 14.5, 14.4, 8.9; m/z HRMS (ESI) found $[M+H]^+$ 444.2369, $C_{25}H_{34}NO_6$ requires 444.2381.

2D NOESY experiment:

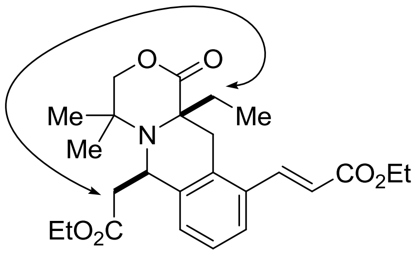

Butyl 2-(8a-ethyl-4,4-dimethyl-1-oxohexahydro-1*H*-pyrrolo[2,1-*c*][1,4]oxazin-6-yl) acetate (5p)

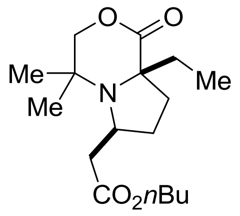

Prepared according to general procedure using 3,3-diethyl-5,5-dimethylmorpholin-2-one (37 mg, 0.2 mmol), butyl acrylate (51.3 mg, 0.4 mmol), $Pd(OAc)_2$ (4.5 mg, 0.02 mmol), AgOAc (100.1 mg, 0.6 mmol) in hexafluoroisopropanol (1 mL) at 60 ℃ for 24 hours. Then the reaction mixture was cooled to room temperature, filtered through celite, eluting with ethyl acetate, and concentrated *in vacuo*. The crude product was purified by flash column chromatography (80/20 40-60 ℃ Petroleum Ether/EtOAc) to provide the title compound as a colorless oil (56.7 mg, 91% yield, single diastereomer, *cis*). IR ν_{max}/cm^{-1} (film): 2961, 1730, 1463, 1382, 1280, 1121, 1060, 992; ^{1}H NMR (500 MHz, $CDCl_3$) δ: 4.17 (d, *J* = 10.7 Hz, 1H), 4.11-4.03 (m, 2H), 3.86 (d, *J* = 10.7 Hz, 1H), 3.71-3.63 (m, 1H), 2.60 (dd, *J* = 15.0, 4.7 Hz, 1H), 2.32 (dd, *J* = 15.0, 9.6 Hz, 1H), 2.16-2.07 (m, 1H), 2.06-1.95 (m, 2H), 1.74-1.48 (m, 5H), 1.44-1.32 (m, 2H), 1.12 (s, 3H), 1.09 (s, 3H), 0.97-0.89 (m, 6H); ^{13}C NMR (125 MHz, $CDCl_3$) δ: 175.0, 172.0, 77.0, 70.5, 64.4, 57.1, 53.1, 45.1, 36.2, 35.8, 30.8, 29.6, 26.1, 19.3, 18.4, 13.9, 9.3; m/z HRMS (ESI) found $[M+H]^+$ 312.2171, $C_{17}H_{30}NO_4$ requires 312.2169.

2D NOESY experiment:

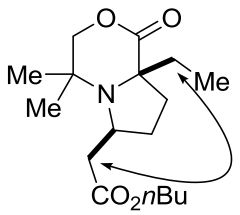

Benzyl 2-(8a-ethyl-4,4-dimethyl-1-oxohexahydro-1*H*-pyrrolo[2,1-*c*][1,4] oxazin-6-yl)acetate (5q)

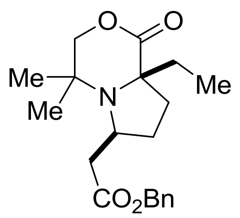

Prepared according to general procedure using 3,3-diethyl-5,5-dimethylmorpholin-2-one (37 mg, 0.2 mmol), benzyl acrylate (64.9 mg, 0.4 mmol), $Pd(OAc)_2$ (4.5 mg, 0.02 mmol), AgOAc (100.1 mg, 0.6 mmol) in hexafluoroisopropanol (1 mL) at 60 ℃ for 24 hours. Then the reaction mixture was cooled to room temperature, filtered through celite, eluting with ethyl acetate, and concentrated *in vacuo*. The crude product was purified by flash column chromatography (80/20 40-60 ℃ Petroleum Ether/EtOAc) to provide the title compound as a colorless oil (61.5 mg, 89% yield, single diastereomer, *cis*). IR ν_{max}/cm^{-1} (film): 2935, 1730, 1457, 1380, 1280, 1121, 1056, 993, 738, 697; ^{1}H NMR (500 MHz, $CDCl_3$) δ: 7.45-7.28 (m, 5H), 5.13 (d, *J* = 12.3 Hz, 1H), 5.09 (d, *J* = 12.3 Hz, 1H), 4.16 (d, *J* = 10.7 Hz, 1H), 3.85 (d, *J* = 10.7 Hz, 1H), 3.75-3.64 (m, 1H), 2.65 (dd, *J* = 15.1, 4.8 Hz, 1H), 2.38 (dd, *J* = 15.1, 9.5 Hz, 1H), 2.15-2.07 (m, 1H), 2.04-1.94 (m, 2H), 1.73-1.49 (m, 3H), 1.10 (s, 3H), 1.08 (s, 3H), 0.92 (t, *J* = 7.5 Hz, 3H); ^{13}C NMR (125 MHz, $CDCl_3$) δ: 174.9, 171.7, 135.9, 128.7, 128.5, 128.5, 77.0, 70.5, 66.4, 57.0, 53.1, 44.9, 36.2, 35.8, 29.6, 26.1, 18.4, 9.2; m/z HRMS (ESI) found $[M+H]^+$ 346.2013, $C_{20}H_{28}NO_4$ requires 346.2013.

2D NOESY experiment:

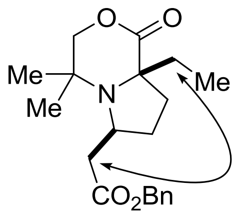

2,2,2-Trifluoroethyl 2-(8a-ethyl-4,4-dimethyl-1-oxohexahydro-1*H*-pyrrolo[2,1-*c*][1,4]oxazin-6-yl)acetate (5r)

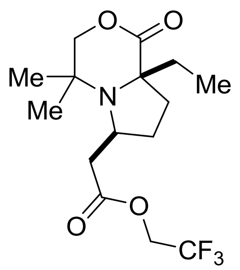

Prepared according to general procedure using 3,3-diethyl-5,5-dimethylmorpholin-2-one (37 mg, 0.2 mmol), 2,2,2-trifluoroethyl acrylate (61.6 mg, 0.4 mmol), $Pd(OAc)_2$ (4.5 mg, 0.02 mmol), AgOAc (100.1 mg, 0.6 mmol) in hexafluoroisopropanol (1 mL) at 60 ℃ for 24 hours. Then the reaction mixture was cooled to room temperature, filtered through celite, eluting with ethyl acetate, and concentrated *in vacuo*. The crude product was purified by flash column chromatography (80/20 40-60 ℃ Petroleum Ether/EtOAc) to provide the title compound as a colorless oil (60.7 mg, 90% yield, single diastereomer, *cis*). IR ν_{max}/cm^{-1} (film): 2934, 1737, 1278, 1160, 1056, 977; ^{1}H NMR (500 MHz, $CDCl_3$) δ: 4.54-4.38 (m, 2H), 4.18 (d, *J* = 10.7 Hz, 1H), 3.86 (d, *J* = 10.7 Hz, 1H), 3.74-3.64 (m, 1H), 2.70 (dd, *J* = 15.2, 4.8 Hz, 1H), 2.44 (dd, *J* = 15.2, 9.4 Hz, 1H), 2.20-2.08 (m, 1H), 2.07-1.95 (m, 2H), 1.77-1.59 (m, 2H), 1.58-1.49 (m, 1H), 1.12 (s, 3H), 1.09 (s, 3H), 0.93 (t, *J* = 7.5 Hz, 3H); ^{13}C NMR (125 MHz, $CDCl_3$) δ: 174.7, 170.2, 123.0 (q, *J* = 275.5 Hz), 76.9, 70.6, 60.3 (q, *J* = 36.4 Hz), 56.9, 53.2, 44.1, 36.1, 35.8, 29.5, 26.1, 18.4, 9.2; ^{19}F NMR (376 MHz, $CDCl_3$) δ: -73.7; m/z HRMS (ESI) found $[M+H]^+$ 338.1573, $C_{15}H_{23}F_3NO_4$ requires 338.1574.

2D NOESY experiment:

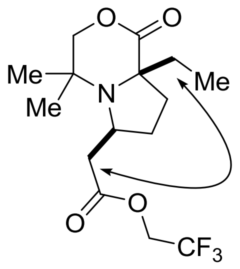

1,1,1,3,3,3-Hexafluoropropan-2-yl 2-(8a-ethyl-4,4-dimethyl-1-oxohexahydro-1*H*-pyrrolo[2,1-*c*][1,4]oxazin-6-yl)acetate (5s)

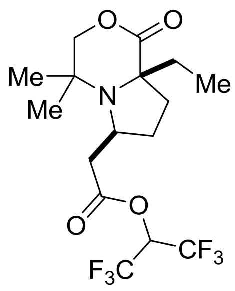

Prepared according to general procedure using 3,3-diethyl-5,5-dimethylmorpholin-2-one (37 mg, 0.2 mmol), 1,1,1,3,3,3-hexafluoropropan-2-yl acrylate (88.8 mg, 0.4 mmol), $Pd(OAc)_2$ (4.5 mg, 0.02 mmol), AgOAc (100.1 mg, 0.6 mmol) in hexafluoroisopropanol (1 mL) at 60 ℃ for 24 hours. Then the reaction mixture was cooled to room temperature, filtered through celite, eluting with ethyl acetate, and concentrated *in vacuo*. The crude product was purified by flash column chromatography (80/20 40-60 ℃ Petroleum Ether/EtOAc) to provide the title compound as a colorless oil (72.1 mg, 89% yield, single diastereomer, *cis*). IR ν_{max}/cm^{-1} (film): 2934, 1776, 1738, 1386, 1282, 1196, 1107, 906, 690; ^{1}H NMR (400 MHz, $CDCl_3$) δ: 5.76 (sp, *J* = 6.1 Hz, 1H), 4.18 (d, *J* = 10.8 Hz, 1H), 3.87 (d, *J* = 10.8 Hz, 1H), 3.78-3.63 (m, 1H), 2.82 (dd, *J* = 15.4, 4.6 Hz, 1H), 2.53 (dd, *J* = 15.4, 9.6 Hz, 1H), 2.21-2.09 (m, 1H), 2.07-1.94 (m, 2H), 1.79-1.59 (m, 2H), 1.58-1.46 (m, 1H), 1.13 (s, 3H), 1.10 (s, 3H), 0.93 (t, *J* = 7.5 Hz, 3H); ^{13}C NMR (100 MHz, $CDCl_3$) δ: 174.5, 168.4, 120.5 (q, *J* = 280.1 Hz), 76.8, 70.6, 66.5 (sp, *J* = 34.5 Hz), 56.8, 53.3, 43.7, 36.0, 35.7, 29.4, 26.0, 18.4, 9.2; ^{19}F NMR (376 MHz, $CDCl_3$) δ: -73.2; m/z HRMS (ESI) found $[M+H]^+$ 406.1440, $C_{16}H_{22}F_6NO_4$ requires 406.1448.

2D NOESY experiment:

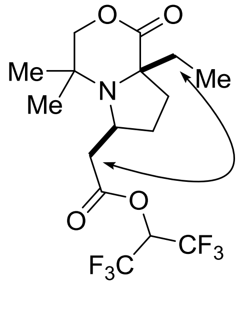

4-(Trifluoromethyl)benzyl 2-(8a-ethyl-4,4-dimethyl-1-oxohexahydro-1*H*-pyrrolo

[2,1-*c*][1,4]oxazin-6-yl)acetate (5t)

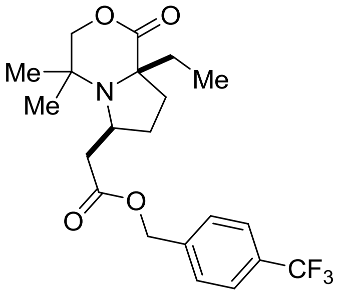

Prepared according to general procedure using 3,3-diethyl-5,5-dimethylmorpholin-2-one (37 mg, 0.2 mmol), 4-(trifluoromethyl)benzyl acrylate (92.1 mg, 0.4 mmol), $Pd(OAc)_2$ (4.5 mg, 0.02 mmol), AgOAc (100.1 mg, 0.6 mmol) in hexafluoroisopropanol (1 mL) at 60 ℃ for 24 hours. Then the reaction mixture was cooled to room temperature, filtered through celite, eluting with ethyl acetate, and concentrated *in vacuo*. The crude product was purified by flash column chromatography (80/20 40-60 ℃ Petroleum Ether/EtOAc) to provide the title compound as a colorless oil (71.8 mg, 87% yield, single diastereomer, *cis*). IR ν_{max}/cm^{-1} (film): 2934, 1733, 1324, 1120, 1065, 1017, 822, 738; ^1H NMR (400 MHz, $CDCl_3$) δ: 7.62 (d, J = 8.1 Hz, 2H), 7.46 (d, J = 8.1 Hz, 2H), 5.24-5.08 (m, 2H), 4.15 (d, J = 10.8 Hz, 1H), 3.85 (d, J = 10.8 Hz, 1H), 3.76-3.61 (m, 1H), 2.67 (dd, J = 15.1, 4.7 Hz, 1H), 2.40 (dd, J = 15.1, 9.5 Hz, 1H), 2.18-2.06 (m, 1H), 2.05-1.92 (m, 2H), 1.75-1.47 (m, 3H), 1.10 (s, 3H), 1.07 (s, 3H), 0.92 (t, J = 7.5 Hz, 3H); ^{13}C NMR (100 MHz, $CDCl_3$) δ: 174.8, 171.5, 139.9, 130.6 (q, J = 32.3 Hz), 128.4, 125.7 (q, J = 3.7 Hz), 124.1 (q, J = 270.4 Hz), 76.9, 70.5, 65.4, 57.0, 53.1, 44.8, 36.1, 35.8, 29.6, 26.1, 18.4, 9.2; ^{19}F NMR (376 MHz, $CDCl_3$) δ: -62.7; m/z HRMS (ESI) found $[M+H]^+$ 414.1878, $C_{21}H_{27}F_3NO_4$ requires 414.1887.

2D NOESY experiment:

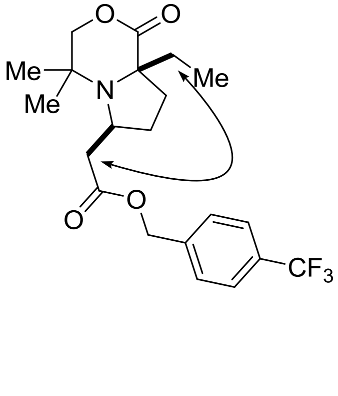

8a-Ethyl-4,4-dimethyl-6-(2-oxopropyl)hexahydro-1*H*-pyrrolo[2,1-*c*][1,4]oxazin-1-one (5u)

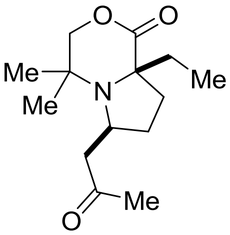

Prepared according to general procedure using 3,3-diethyl-5,5-dimethylmorpholin-2-one (37 mg, 0.2 mmol), but-3-en-2-one (28.1 mg, 0.4 mmol), $Pd(OAc)_2$ (4.5 mg, 0.02 mmol), AgOAc (100.1 mg, 0.6 mmol) in hexafluoroisopropanol (1 mL) at 60 ℃ for 24 hours. Then the reaction mixture was cooled to room temperature, filtered through celite, eluting with ethyl acetate, and concentrated *in vacuo*. The crude product was purified by flash column chromatography (75/25 40-60 ℃ Petroleum Ether/EtOAc) to provide the title compound as a colorless oil (33.4 mg, 66% yield, single diastereomer, *cis*). IR ν_{max}/cm^{-1} (film): 2934, 1733, 1709, 1464, 1371, 1281, 1122, 1056, 912, 730; ^{1}H NMR (500 MHz, $CDCl_3$) δ: 4.15 (d, *J* = 10.7 Hz, 1H), 3.85 (d, *J* = 10.7 Hz, 1H), 3.75-3.64 (m, 1H), 2.75 (dd, *J* = 17.0, 3.9 Hz, 1H), 2.51 (dd, *J* = 17.0, 9.7 Hz, 1H), 2.13 (s, 3H), 2.11-2.02 (m, 2H), 2.00-1.90 (m, 1H), 1.76-1.64 (m, 1H), 1.63-1.54 (m, 1H), 1.40-1.29 (m, 1H), 1.07 (s, 3H), 1.07 (s, 3H), 0.93 (t, *J* = 7.5 Hz, 3H); ^{13}C NMR (125 MHz, $CDCl_3$) δ: 207.9, 175.0, 77.1, 70.4, 56.0, 54.6, 53.1, 36.3, 35.9, 31.1, 30.1, 26.1, 18.3, 9.3; m/z HRMS (ESI) found $[M+H]^+$ 254.1752, $C_{14}H_{24}NO_3$ requires 254.1751.

2D NOESY experiment:

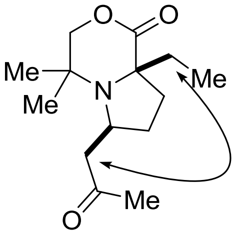

8a-Ethyl-4,4-dimethyl-6-(2-oxopentyl)hexahydro-1*H*-pyrrolo[2,1-*c*][1,4]oxazin-1-one (5v)

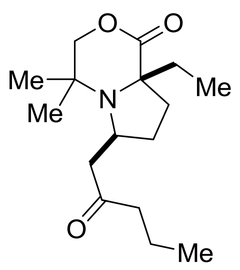

Prepared according to general procedure using 3,3-diethyl-5,5-dimethylmorpholin-2-one (37 mg, 0.2 mmol), hex-1-en-3-one (39.2 mg, 0.4 mmol), $Pd(OAc)_2$ (4.5 mg, 0.02 mmol), Ac-Gly-OH (4.7 mg, 0.04 mmol), AgOAc (100.1 mg, 0.6 mmol) in hexafluoroisopropanol (1 mL) at 60 ℃ for 24 hours. Then the reaction mixture was cooled to room temperature, filtered through celite, eluting with ethyl acetate, and concentrated *in vacuo*. The crude product was purified by flash column chromatography (75/25 40-60 ℃ Petroleum Ether/EtOAc) to provide the title compound as a colorless oil (32.6 mg, 58% yield, single diastereomer, *cis*). IR ν_{max}/cm^{-1} (film): 2965, 1736, 1707, 1463, 1372, 1280, 1122, 1057, 1018, 739; ^1H NMR (500 MHz, $CDCl_3$) δ: 4.16 (d, *J* = 10.7 Hz, 1H), 3.85 (d, *J* = 10.7 Hz, 1H), 3.75-3.67 (m, 1H), 2.71 (dd, *J* = 16.8, 4.0 Hz, 1H), 2.47 (dd, *J* = 16.8, 9.6 Hz, 1H), 2.41-2.31 (m, 2H), 2.13-2.01 (m, 2H), 1.99-1.92 (m, 1H), 1.73-1.64 (m, 1H), 1.62-1.55 (m, 3H), 1.37-1.29 (m, 1H), 1.07 (s, 6H), 0.98-0.86 (m, 6H); ^{13}C NMR (125 MHz, $CDCl_3$) δ: 210.2, 175.0, 77.1, 70.4, 56.1, 53.7, 53.1, 46.0, 36.4, 35.9, 30.1, 26.1, 18.3, 17.3, 13.9, 9.3; m/z HRMS (ESI) found $[M+H]^+$ 282.2063, $C_{16}H_{28}NO_3$ requires 282.2064.

2D NOESY experiment:

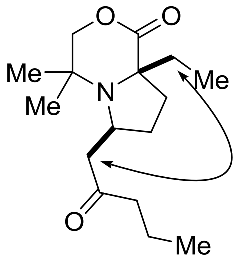

2-(8a-Ethyl-4,4-dimethyl-1-oxohexahydro-1*H*-pyrrolo[2,1-*c*][1,4]oxazin-6-yl) acetaldehyde (5w)

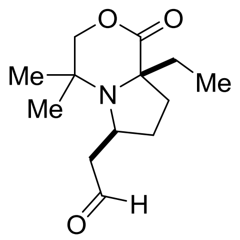

Prepared according to general procedure using 3,3-diethyl-5,5-dimethylmorpholin-2-one (37 mg, 0.2 mmol), acrolein (22.5 mg, 0.4 mmol), $Pd(OAc)_2$ (4.5 mg, 0.02 mmol), Ac-Gly-OH (4.7 mg, 0.04 mmol), AgOAc (100.1 mg, 0.6 mmol) in hexafluoroisopropanol (1 mL) at room temperature for 24 hours. Then the reaction mixture was filtered through celite, eluting with ethyl acetate, and concentrated *in vacuo*. The crude product was purified by flash column chromatography (75/25 40-60 ℃ Petroleum Ether/EtOAc) to provide the title compound as a colorless oil (14.6 mg, 31% yield, single diastereomer, *cis*). IR ν_{max}/cm^{-1} (film): 2971, 1719, 1463, 1383, 1281, 1123, 1056, 740; ^{1}H NMR (400 MHz, $CDCl_3$) δ: 9.79 (t, *J* = 1.8 Hz, 1H), 4.19 (d, *J* = 10.8 Hz, 1H), 3.86 (d, *J* = 10.8 Hz, 1H), 3.81-3.70 (m, 1H), 2.72 (ddd, *J* = 16.8, 4.5, 1.8 Hz, 1H), 2.56 (ddd, *J* = 16.8, 8.8, 1.8 Hz, 1H), 2.22-1.92 (m, 3H), 1.74-1.59 (m, 2H), 1.53-1.43 (m, 1H), 1.11 (s, 3H), 1.09 (s, 3H), 0.93 (t, *J* = 7.5 Hz, 3H); ^{13}C NMR (100 MHz, $CDCl_3$) δ: 201.9, 174.8, 76.9, 70.4, 55.0, 53.9, 53.3, 36.0, 35.8, 30.1, 26.3, 18.4, 9.3; m/z HRMS (ESI) found $[M+Na]^+$ 262.1417, $C_{13}H_{21}NNaO_3$ requires 262.1414.

2D NOESY experiment:

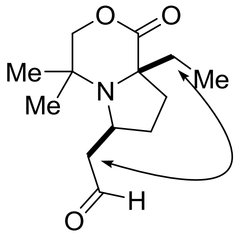

2-(8a-Ethyl-4,4-dimethyl-1-oxohexahydro-1*H*-pyrrolo[2,1-*c*][1,4]oxazin-6-yl)-*N*,*N*-dimethylacetamide (5x)

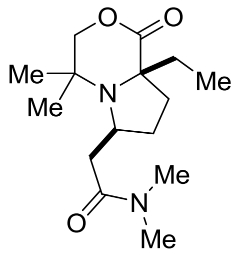

Prepared according to general procedure using 3,3-diethyl-5,5-dimethylmorpholin-2-one (37 mg, 0.2 mmol), *N*,*N*-dimethylacrylamide (39.6 mg, 0.4 mmol), $Pd(OAc)_2$ (4.5 mg, 0.02 mmol), Ac-Gly-OH (4.7 mg, 0.04 mmol), AgOAc (100.1 mg, 0.6 mmol) in hexafluoroisopropanol (1 mL) at 60 ℃ for 24 hours. Then the reaction mixture was cooled to room temperature, filtered through celite, eluting with ethyl acetate, and concentrated *in vacuo*. The crude product was purified by flash column chromatography using an eluent of EtOAc to provide the title compound as an amorphous solid (28.8 mg, 51% yield, single diastereomer, *cis*). IR ν_{max}/cm^{-1} (film): 2969, 1733, 1635, 1463, 1397, 1280, 1120, 1055, 801; ^{1}H NMR (500 MHz, $CDCl_3$) δ: 4.15 (d, J = 10.7 Hz, 1H), 3.85 (d, J = 10.7 Hz, 1H), 3.82-3.73 (m, 1H), 3.00 (s, 3H), 2.92 (s, 3H), 2.58 (dd, J = 15.7, 4.1 Hz, 1H), 2.34 (dd, J = 15.7, 9.6 Hz, 1H), 2.17-2.04 (m, 2H), 2.01-1.90 (m, 1H), 1.75-1.57 (m, 2H), 1.48-1.35 (m, 1H), 1.09 (s, 3H), 1.08 (s, 3H), 0.94 (t, J = 7.5 Hz, 3H); ^{13}C NMR (125 MHz, $CDCl_3$) δ: 175.1, 171.5, 77.2, 70.5, 57.5, 53.1, 44.0, 37.5, 36.4, 35.9, 35.3, 30.2, 26.0, 18.3, 9.3; m/z HRMS (ESI) found $[M+H]^+$ 283.2016, $C_{15}H_{27}N_2O_3$ requires 283.2016.

2D NOESY experiment:

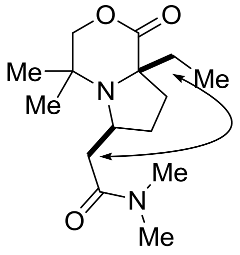

Methyl 2-(2-(8a-ethyl-4,4-dimethyl-1-oxohexahydro-1*H*-pyrrolo[2,1-*c*][1,4] oxazin-6-yl)-*N*-methylacetamido)acetate (3.6:1 rotamers) (5y)

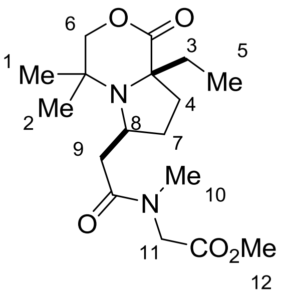

Prepared according to general procedure using 3,3-diethyl-5,5-dimethylmorpholin-2-one (37 mg, 0.2 mmol), methyl 2-(*N*-methylacrylamido)acetate (62.9 mg, 0.4 mmol), $Pd(OAc)_2$ (4.5 mg, 0.02 mmol), AgOAc (100.1 mg, 0.6 mmol) in hexafluoroisopropanol (1 mL) at 60 ℃ for 24 hours. Then the reaction mixture was cooled to room temperature, filtered through celite, eluting with ethyl acetate, and concentrated *in vacuo*. The crude product was purified by flash column chromatography (50/50 40-60 ℃ Petroleum Ether/EtOAc) to provide the title compound as an amorphous solid (44.3 mg, 65% yield, 3.6:1 rotamers). IR ν_{max}/cm^{-1} (film): 2975, 1737, 1646, 1462, 1401, 1281, 1208, 1121, 1056; ^{1}H NMR (500 MHz, $CDCl_3$) δ: major one: 4.16 (H_6, d, *J* = 10.9 Hz, 1H), 4.09 (H_{11}, d, *J* = 3.5 Hz, 2H), 3.85 (H_6, d, *J* = 10.9 Hz, 1H), 3.81-3.77 (H_8, m, 1H), 3.71 (H_{12}, s, 3H), 3.06 (H_{10}, s, 3H), 2.64 (H_9, dd, *J* = 15.9, 3.9 Hz, 1H), 2.42 (H_9, dd, *J* = 15.9, 9.9 Hz, 1H), 2.18-2.01 (H_7 and H_4, m, 2H), 2.00-1.89 (H_4, m, 1H), 1.75-1.59 (H_3, m, 2H), 1.49-1.35 (H_7, m, 1H), 1.11 (H_1 or H_2, s, 3H), 1.07 (H_1 or H_2, s, 3H), 0.93 (H_5, t, *J* = 7.5 Hz, 3H), minor one: 4.13 ($H_{6'}$, d, *J* = 10.9 Hz, 1H), 4.09-3.98 ($H_{11'}$, m, 2H), 3.84 ($H_{6'}$, d, *J* = 11.0 Hz, 1H), 3.77-3.73 ($H_{8'}$, m, 1H), 3.76 ($H_{12'}$, s, 3H), 2.94 ($H_{10'}$, s, 3H), 2.51 ($H_{9'}$, dd, *J* = 15.8, 4.0 Hz, 1H), 2.24 ($H_{9'}$, dd, *J* = 15.8, 9.5 Hz, 1H), 2.18-2.01 ($H_{7'}$ and $H_{4'}$, m, 2H), 2.00-1.89 ($H_{4'}$, m, 1H), 1.59-1.52 ($H_{3'}$, m, 2H), 1.49-1.35 ($H_{7'}$, m, 1H), 1.07 ($H_{1'}$ or $H_{2'}$, s, 3H), 1.06 ($H_{1'}$ or $H_{2'}$, s, 3H), 0.91 ($H_{5'}$, t, *J* = 7.3 Hz, 3H); ^{13}C NMR (125 MHz, $CDCl_3$) δ: major one: 175.0, 172.2, 169.9, 77.1, 70.5, 57.2, 53.1, 52.2, 49.3, 43.7, 36.8, 36.3, 35.9, 30.0, 26.0, 18.3, 9.3, minor one: 174.9, 171.9, 169.6, 77.1, 70.5, 57.3, 52.6, 51.6, 49.3, 43.6, 36.3, 35.8, 34.8, 30.1, 25.9, 18.3, 9.3; m/z HRMS (ESI) found $[M+H]^+$ 341.2068, $C_{17}H_{29}N_2O_5$ requires 341.2071.

2D NOESY experiment:

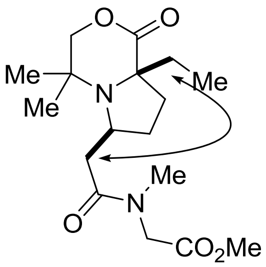

8a-Ethyl-4,4-dimethyl-6-(phenylsulfonylmethyl)hexahydro-1*H*-pyrrolo[2,1-*c*][1,4]oxazin-1-one (5z)

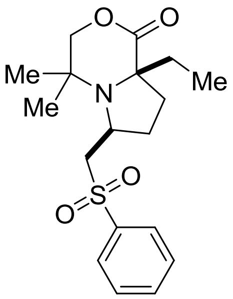

Prepared according to general procedure using 3,3-diethyl-5,5-dimethylmorpholin-2-one (37 mg, 0.2 mmol), vinylsulfonylbenzene (67.3 mg, 0.4 mmol), $Pd(OAc)_2$ (4.5 mg, 0.02 mmol), Ac-Gly-OH (4.7 mg, 0.04 mmol), AgOAc (100.1 mg, 0.6 mmol) in hexafluoroisopropanol (1 mL) at 60 ℃ for 24 hours. Then the reaction mixture was cooled to room temperature, filtered through celite, eluting with ethyl acetate, and concentrated *in vacuo*. The crude product was purified by flash column chromatography (60/40 40-60 ℃ Petroleum Ether/EtOAc) to provide the title compound as a white solid (65.4 mg, 93% yield, single diastereomer, *cis*). For the gram scale reaction, a 250 mL round bottom flask equipped with stir bar was charged with 3,3-diethyl-5,5-dimethylmorpholin-2-one (1.85 g, 10.0 mmol), vinylsulfonylbenzene (3.36 g, 20.0 mmol), $Pd(OAc)_2$ (224.5 mg, 1.0 mmol), Ac-Gly-OH (234.2 mg, 2.0 mmol), AgOAc (5.01 g, 30.0 mmol), and hexafluoroisopropanol (50 mL). The round bottom flask was then fitted with a reflux condenser under air and placed in a pre-heated oil bath at 80 ℃ for 48 hours. Then the reaction mixture was cooled to room temperature, filtered through celite, eluting with ethyl acetate, and concentrated *in vacuo*. The crude product was purified by flash column chromatography (60/40 40-60 ℃ Petroleum Ether/EtOAc) to provide the title

compound as a white solid (3.018 g, 86% yield, single diastereomer, *cis*). m.p. 120-122 ℃; IR ν_{max}/cm^{-1} (film): 2973, 1733, 1447, 1304, 1145, 1058, 739, 689; ^{1}H NMR (400 MHz, $CDCl_3$) δ: 7.99-7.88 (m, 2H), 7.68 (t, *J* = 7.5 Hz, 1H), 7.59 (t, *J* = 7.5 Hz, 2H), 4.10 (d, *J* = 10.8 Hz, 1H), 3.82 (d, *J* = 10.8 Hz, 1H), 3.75-3.64 (m, 1H), 3.32 (dd, *J* = 14.0, 2.0 Hz, 1H), 3.12 (dd, *J* = 14.0, 10.6 Hz, 1H), 2.16-1.90 (m, 3H), 1.67-1.45 (m, 3H), 1.00 (s, 3H), 0.98 (s, 3H), 0.88 (t, *J* = 7.4 Hz, 3H); ^{13}C NMR (100 MHz, $CDCl_3$) δ: 174.3, 139.8, 134.1, 129.6, 128.0, 76.8, 70.0, 65.6, 54.7, 53.5, 36.5, 35.6, 30.0, 25.5, 18.2, 9.2; m/z HRMS (ESI) found $[M+H]^+$ 352.1577, $C_{18}H_{26}NO_4S$ requires 352.1577.

2D NOESY experiment:

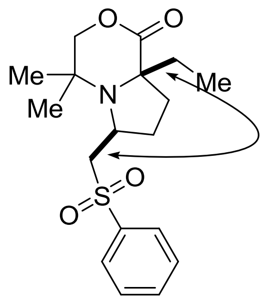

Diethyl (8a-ethyl-4,4-dimethyl-1-oxohexahydro-1*H*-pyrrolo[2,1-*c*][1,4]oxazin-6-yl)methylphosphonate (5aa)

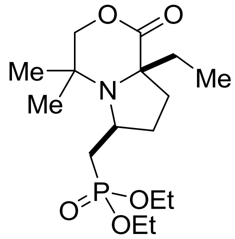

Prepared according to general procedure using 3,3-diethyl-5,5-dimethylmorpholin-2-one (37 mg, 0.2 mmol), diethyl vinylphosphonate (65.6 mg, 0.4 mmol), $Pd(OAc)_2$ (4.5 mg, 0.02 mmol), Ac-Gly-OH (4.7 mg, 0.04 mmol), AgOAc (100.1 mg, 0.6 mmol) in hexafluoroisopropanol (1 mL) at 60 ℃ for 24 hours. Then the reaction mixture was cooled to room temperature, filtered through celite, eluting with ethyl acetate. The filtrates were further filtered through SCX-2, washing with methanol and then eluting with ammonia methanol

solution (7 M). The ammonia methanol solution filtrate was concentrated *in vacuo* to recover the amine compounds from the crude mixture. The mixture of amines was then purified by flash column chromatography using an eluent of EtOAc to provide the title compound as a colorless oil (42.1 mg, 60% yield, single diastereomer, *cis*). IR ν_{max}/cm^{-1} (film): 2976, 1735, 1463, 1385, 1213, 1122, 1020, 958, 799; ^{1}H NMR (400 MHz, $CDCl_3$) δ: 4.19-4.00 (m, 5H), 3.84 (d, *J* = 10.7 Hz, 1H), 3.63-3.52 (m, 1H), 2.17-1.97 (m, 4H), 1.88-1.77 (m, 1H), 1.73-1.52 (m, 3H), 1.32 (t, *J* = 7.1 Hz, 6H), 1.13 (s, 3H), 1.06 (s, 3H), 0.91 (t, *J* = 7.5 Hz, 3H); ^{13}C NMR (100 MHz, $CDCl_3$) δ: 174.9 (d, *J* = 1.2 Hz), 77.1, 70.2 (d, *J* = 1.0 Hz), 61.6 (d, *J* = 6.3 Hz), 61.5 (d, *J* = 6.3 Hz), 55.1 (d, *J* = 1.6 Hz), 53.3, 37.5 (d, *J* = 130.2 Hz), 36.5, 35.6, 30.3, 26.0, 18.3, 16.63, 16.57, 9.2; ^{31}P NMR (162 MHz, $CDCl_3$) δ: 28.6; m/z HRMS (ESI) found $[M+H]^+$ 348.1934, $C_{16}H_{31}NO_5P$ requires 348.1934.

2D NOESY experiment:

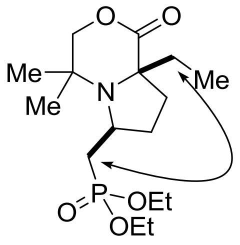

2,2,2-Trifluoroethyl 5-(3-ethyl-5,5-dimethyl-2-oxomorpholin-3-yl)-2-methylenepentanoate (7a)

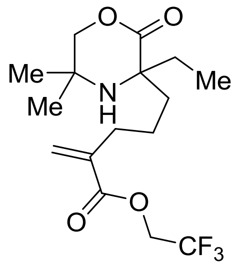

Prepared according to general procedure using 3,3-diethyl-5,5-dimethylmorpholin-2-one (37 mg, 0.2 mmol), 2,2,2-trifluoroethyl methacrylate (67.2 mg, 0.4 mmol), $Pd(OAc)_2$ (4.5 mg, 0.02 mmol), Ac-Gly-OH (4.7 mg, 0.04 mmol), AgOAc (100.1 mg, 0.6 mmol) in hexafluoroisopropanol (1 mL) at 60 ℃ for 24 hours. Then the reaction mixture was cooled to room temperature,

filtered through celite, eluting with ethyl acetate, and concentrated *in vacuo*. The crude product was purified by flash column chromatography (75/25 40-60 ℃ Petroleum Ether/EtOAc) to provide the title compound as a colorless oil (26.0 mg, 37% yield). IR ν_{max}/cm^{-1} (film): 2971, 1730, 1380, 1282, 1132, 1054, 976, 811, 667; ^1H NMR (500 MHz, $CDCl_3$) δ: 6.27 (d, *J* = 1.2 Hz, 1H), 5.69 (d, *J* = 1.2 Hz, 1H), 4.54 (d, *J* = 8.4 Hz, 1H), 4.51 (d, *J* = 8.4 Hz, 1H), 4.09 (s, 2H), 2.32 (t, *J* = 7.0 Hz, 2H), 1.79-1.50 (m, 6H), 1.17 (s, 6H), 0.92 (t, *J* = 7.4 Hz, 3H); ^{13}C NMR (125 MHz, $CDCl_3$) δ: 174.0, 165.4, 138.8, 127.6, 123.2 (q, *J* = 275.5 Hz), 77.6, 61.0, 60.7 (q, *J* = 36.3 Hz), 48.6, 39.6, 33.3, 32.0, 26.8, 26.7, 22.5, 8.3; ^{19}F NMR (376 MHz, $CDCl_3$) δ: -73.7; m/z HRMS (ESI) found $[M+H]^+$ 352.1728, $C_{16}H_{25}F_3NO_4$ requires 352.1730.

3-Ethyl-5,5-dimethyl-3-(3-(2-oxo-2,5-dihydrofuran-3-yl)propyl)morpholin-2-one (7b)

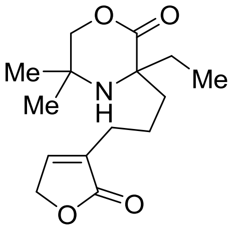

Prepared according to general procedure using 3,3-diethyl-5,5-dimethylmorpholin-2-one (37 mg, 0.2 mmol), 3-methylenedihydrofuran-2(3*H*)-one (39.2 mg, 0.4 mmol), $Pd(OAc)_2$ (4.5 mg, 0.02 mmol), Ac-Gly-OH (4.7 mg, 0.04 mmol), AgOAc (100.1 mg, 0.6 mmol) in hexafluoroisopropanol (1 mL) at 60 ℃ for 24 hours. Then the reaction mixture was cooled to room temperature, filtered through celite, eluting with ethyl acetate, and concentrated *in vacuo*. The crude product was purified by flash column chromatography (70/30 40-60 ℃ Petroleum Ether/EtOAc) to provide the title compound as a colorless oil (28.1 mg, 50% yield). IR ν_{max}/cm^{-1} (film): 2968, 1726, 1458, 1379, 1285, 1202, 1052, 830, 730; ^1H NMR (500 MHz, $CDCl_3$) δ: 7.19-7.12 (m, 1H), 4.76 (dd, *J* = 3.6, 1.6 Hz, 2H), 4.09 (s, 2H), 2.29 (td, *J* = 7.0, 1.6 Hz, 2H), 1.80-1.55 (m, 6H), 1.17 (s, 6H), 0.91 (t, *J* = 7.4 Hz, 3H); ^{13}C NMR (125 MHz, $CDCl_3$) δ: 174.5, 174.1, 144.8, 133.9, 77.7, 70.3, 61.0, 48.7, 39.6, 33.5, 26.79, 26.76, 25.5,

21.8, 8.3; m/z HRMS (ESI) found $[M+H]^+$ 282.1699, $C_{15}H_{24}NO_4$ requires 282.1700.

4,4-Dimethyl-6-(phenylsulfonylmethyl)-8a-((triisopropylsilyloxy)methyl)hexahydro-1*H*-pyrrolo[2,1-*c*][1,4]oxazin-1-one (8)

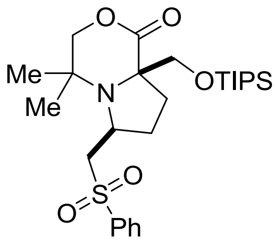

Prepared according to general procedure using 3-ethyl-5,5-dimethyl-3-((triisopropylsilyloxy)methyl)morpholin-2-one (240.5 mg, 0.7 mmol), vinylsulfonylbenzene (235.5 mg, 1.4 mmol), $Pd(OAc)_2$ (15.8 mg, 0.07 mmol), Ac-Gly-OH (16.5 mg, 0.14 mmol), AgOAc (350 mg, 2.1 mmol) in hexafluoroisopropanol (5 mL) at 80 °C for 36 hours. Then the reaction mixture was cooled to room temperature, filtered through celite, eluting with ethyl acetate, and concentrated *in vacuo*. The crude product was purified by flash column chromatography (70/30 40-60 °C Petroleum Ether/EtOAc) to provide the title compound as a pale solid (196.3 mg, 55% yield, single diastereomer, *cis*). m.p. 108-110 °C; IR ν_{max}/cm^{-1} (film): 2945, 1737, 1464, 1306, 1147, 999, 882, 792, 736, 687; ^{1}H NMR (400 MHz, $CDCl_3$) δ: 7.96-7.88 (m, 2H), 7.69 (t, *J* = 7.4 Hz, 1H), 7.60 (t, *J* = 7.6 Hz, 2H), 4.37 (d, *J* = 10.4 Hz, 1H), 3.80-3.68 (m, 3H), 3.45 (d, *J* = 9.1 Hz, 1H), 3.34 (dd, *J* = 14.1, 1.8 Hz, 1H), 3.13 (dd, *J* = 14.1, 10.6 Hz, 1H), 2.14-2.07 (m, 1H), 2.05-1.92 (m, 2H), 1.64-1.55 (m, 1H), 1.23-0.80 (m, 27H); ^{13}C NMR (100 MHz, $CDCl_3$) δ: 173.9, 139.8, 134.1, 129.6, 128.0, 76.6, 72.1, 71.5, 65.2, 55.3, 53.4, 33.9, 30.3, 25.5, 18.5, 18.0, 18.0, 12.0; m/z HRMS (ESI) found $[M+H]^+$ 510.2694, $C_{26}H_{44}NO_5SSi$ requires 510.2704.

2D NOESY experiment:

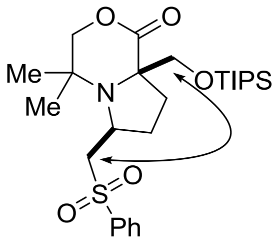

4.5 Unsuccessful Morpholinone Amine Substrates

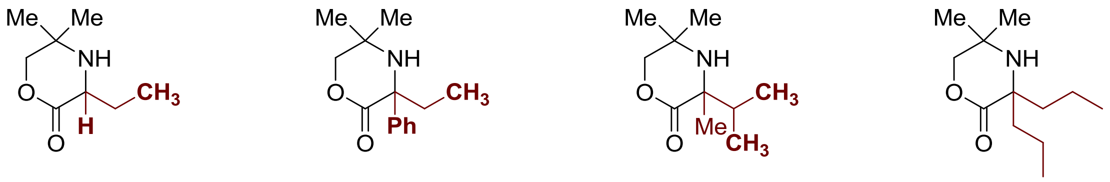

Scheme S1. Unsuccessful morpholinone amine substrates.

5. Derivatization of the Alkenylation Product

2-(2-Ethyl-2-(hydroxymethyl)-5-(phenylsulfonylmethyl)pyrrolidin-1-yl)-2-methyl propan-1-ol

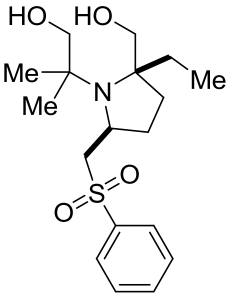

To a solution of 8a-ethyl-4,4-dimethyl-6-(phenylsulfonylmethyl)hexahydro-1*H*-pyrrolo[2,1-*c*][1,4]oxazin-1-one (2.11 g, 6.0 mmol) in THF (120 mL) at room temperature under nitrogen was dropwise a solution of diisobutylaluminum hydride in THF (1.0 M, 24 mL, 24.0 mmol). The mixture was stirred at 35 ℃ overnight, then quenched with H_2O (20 mL). A white precipitate formed, which was separated by filtration, washed with THF (50 mL) and EtOAc (3 × 50 mL). The filtrate was dried ($MgSO_4$), filtered and concentrated *in vacuo* to provide the title compound as a white solid (2.03 g, 95%

yield). m.p. 106-108 ℃; IR ν_{max}/cm^{-1} (film): 3396, 2964, 1447, 1295, 1141, 1083, 748, 689; ^{1}H NMR (500 MHz, $CDCl_3$) δ: 7.93-7.85 (m, 2H), 7.68-7.60 (m, 1H), 7.60-7.52 (m, 2H), 4.00 (dd, *J* = 9.3, 7.3 Hz, 1H), 3.62 (d, *J* = 11.2 Hz, 1H), 3.54-3.36 (m, 4H), 3.33 (d, *J* = 14.1 Hz, 1H), 3.24 (d, *J* = 11.2 Hz, 1H), 3.00 (dd, *J* = 14.1, 9.9 Hz, 1H), 2.12-1.99 (m, 1H), 1.86-1.74 (m, 2H), 1.67-1.55 (m, 1H), 1.50 (dq, *J* = 14.6, 7.4 Hz, 1H), 1.31 (dq, *J* = 14.6, 7.4 Hz, 1H), 1.11 (s, 3H), 1.10 (s, 3H), 0.82 (t, *J* = 7.4 Hz, 3H); ^{13}C NMR (125 MHz, $CDCl_3$) δ: 140.3, 133.8, 129.5, 128.0, 70.0, 69.8, 69.0, 62.4, 58.2, 55.1, 34.7, 30.6, 29.6, 28.1, 24.6, 8.7; m/z HRMS (ESI) found $[M+H]^+$ 356.1891, $C_{18}H_{30}NO_4S$ requires 356.1890.

2D NOESY experiment:

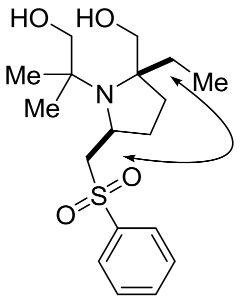

(2-Ethyl-5-(phenylsulfonylmethyl)pyrrolidin-2-yl)methanol (9)

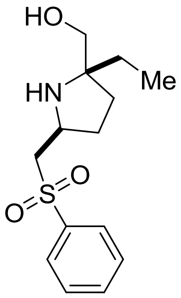

A mixture of 2-(2-ethyl-2-(hydroxymethyl)-5-(phenylsulfonylmethyl)pyrrolidin-1-yl)-2-methylpropan-1-ol (2.03 g, 5.7 mmol) and trifluoroacetic acid (20 mL) was heated at 80 ℃ for 1.5 hours. Then the reaction mixture was cooled to room temperature, and concentrated *in vacuo*. The residue was dissolved in CH_2Cl_2 (20 mL) and further filtered through SCX-2, washing with methanol and then eluting with ammonia methanol solution (7 M). The ammonia methanol solution filtrate was concentrated *in*

vacuo to provide the pure title compound as a white solid (1.55 g, 96% yield). m.p. 100-102 ℃; IR ν_{max}/cm^{-1} (film): 3376, 2966, 1448, 1304, 1144, 1085, 748, 689; ^1H NMR (400 MHz, $CDCl_3$) δ: 8.00-7.84 (m, 2H), 7.70-7.61 (m, 1H), 7.61-7.51 (m, 2H), 3.58-3.45 (m, 1H), 3.35-3.15 (m, 4H), 2.48 (br, 2H), 2.07-1.92 (m, 1H), 1.66-1.34 (m, 5H), 0.85 (t, *J* = 7.5 Hz, 3H); ^{13}C NMR (100 MHz, $CDCl_3$) δ: 139.5, 134.0, 129.5, 128.2, 66.2, 65.9, 62.1, 52.6, 32.4, 31.5, 30.9, 9.0; m/z HRMS (ESI) found $[M+H]^+$ 284.1316, $C_{14}H_{22}NO_3S$ requires 284.1315.

Tert-butyl 2-ethyl-2-(hydroxymethyl)-5-(phenylsulfonylmethyl) pyrrolidine-1-carboxylate

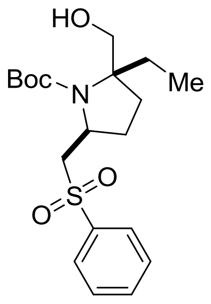

To a solution of (2-ethyl-5-(phenylsulfonylmethyl)pyrrolidin-2-yl)methanol (283.4 mg, 1.0 mmol) in dioxane (2 mL) and water (2 mL) cooled in an ice bath was added Boc anhydride (545.6 mg, 2.5 mmol), followed by $NaHCO_3$ (168.0 mg, 2.0 mmol). After stirring at room temperature overnight, the reaction mixture was then diluted with EtOAc (20 mL) and washed with brine (2 × 5 mL), dried ($MgSO_4$), filtered and concentrated *in vacuo*. The crude material was purified by flash column chromatography (60/40 40-60 °C Petroleum Ether/EtOAc) to provide the title compound as a thick colorless oil (198.2 mg, 52% yield). IR ν_{max}/cm^{-1} (film): 3464, 2972, 1677, 1367, 1306, 1145, 1084, 729, 689; ^1H NMR (400 MHz, $CDCl_3$) δ: 7.90 (d, *J* = 7.5 Hz, 2H), 7.67 (t, *J* = 7.2 Hz, 1H), 7.58 (t, *J* = 7.5 Hz, 2H), 4.33-4.18 (m, 2H), 3.76-3.61 (m, 2H), 3.52 (d, *J* = 13.3 Hz, 1H), 3.02 (t, *J* = 12.2 Hz, 1H), 2.20-2.00 (m, 2H), 2.00-1.82 (m, 2H), 1.77-1.63 (m, 2H), 1.35 (s, 9H), 0.82 (t, *J* = 7.3 Hz, 3H); ^{13}C NMR (100 MHz, $CDCl_3$) δ: 154.9, 140.0, 134.1, 129.6, 128.1, 81.4, 69.0, 68.9, 58.9, 55.2, 32.3, 28.5, 27.1, 26.6, 8.8; m/z HRMS (ESI) found $[M+H]^+$ 384.1839,

$C_{19}H_{30}NO_5S$ requires 384.1839.

Tert-butyl 2-ethyl-2-formyl-5-(phenylsulfonylmethyl)pyrrolidine-1-carboxylate (1.1:1 rotamers) (10)

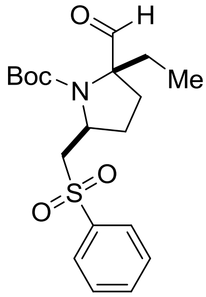

To a solution of *tert*-butyl 2-ethyl-2-(hydroxymethyl)-5-(phenylsulfonylmethyl)pyrrolidine-1-carboxylate (38.3 mg, 0.1 mmol) in DMSO (0.5 mL) cooled in an ice bath was added Et_3N (40.5 mg, 0.4 mmol), followed by SO_3.pyridine (31.8 mg, 2.0 mmol). After stirring at room temperature for 1 hour, the reaction mixture was then diluted with EtOAc (10 mL) and washed with brine (2 × 5 mL), dried ($MgSO_4$), filtered and concentrated *in vacuo*. The crude material was purified by flash column chromatography (80/20 40-60 °C Petroleum Ether/EtOAc) to provide the title compound as a thick colorless oil (36.1 mg, 95% yield, 1.1:1 rotamers). IR ν_{max}/cm^{-1} (film): 2974, 1733, 1691, 1367, 1306, 1145, 1086, 746, 690; ^1H NMR (400 MHz, $CDCl_3$) δ: major one: 9.40 (s, 1H), 7.99-7.87 (m, 2H), 7.72-7.63 (m, 1H), 7.63-7.54 (m, 2H), 4.43-4.25 (m, 1H), 3.96 (dd, *J* = 13.4, 2.1 Hz, 1H), 3.10-2.92 (m, 1H), 2.23-1.72 (m, 6H), 1.35 (s, 9H), 0.86 (t, *J* = 7.5 Hz, 3H), minor one: 9.53 (s, 1H), 7.99-7.87 (m, 2H), 7.72-7.63 (m, 1H), 7.63-7.54 (m, 2H), 4.43-4.25 (m, 1H), 3.55 (d, *J* = 13.4 Hz, 1H), 3.10-2.92 (m, 1H), 2.23-1.72 (m, 6H), 1.31 (s, 9H), 0.83 (t, *J* = 7.5 Hz, 3H); ^{13}C NMR (100 MHz, $CDCl_3$) δ: 200.4, 199.3, 153.21, 153.15, 139.7, 139.6, 134.2, 134.0, 129.7, 129.5, 128.2, 128.1, 81.7, 81.3, 72.8, 72.5, 59.2, 58.3, 55.3, 54.9, 31.5, 29.9, 28.4, 28.2, 27.2, 27.0, 25.3, 24.4, 8.2, 8.0; m/z HRMS (ESI) found $[M+H]^+$ 382.1682, $C_{19}H_{28}NO_5S$ requires 382.1683.

1-(*Tert*-butoxycarbonyl)-2-ethyl-5-(phenylsulfonylmethyl)pyrrolidine-2-

carboxylic acid (1.64:1 rotamers) (11)

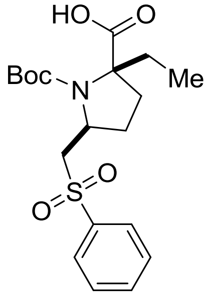

To a solution of *tert*-butyl 2-ethyl-2-formyl-5-(phenylsulfonylmethyl)pyrrolidine-1-carboxylate (66.0 mg, 0.173 mmol) in *t*-BuOH/H_2O (3.5 : 1; 4.5 mL) was added NaH_2PO_4 (66.0 mg, 0.55 mmol), and the reaction mixture was cooled to 0 ℃. Then 2-methyl-2-butene (2 M in THF, 0.5 mL, 1.0 mmol) and $NaClO_2$ (123.0 mg, 1.36 mmol) were added sequentially and stirred at room temperature for 4 hours. The reaction mixture was then diluted with brine (5 mL) and extracted with EtOAc (2 x 10 mL). The aqueous phase was further acidified with HCl (0.5 M) to pH 3.0 and extracted with CH_2Cl_2 (3 x 5 mL). The combined organic phase was dried ($MgSO_4$), filtered and concentrated *in vacuo*. The crude material was purified by flash column chromatography (80/20 CH_2Cl_2/MeOH) to provide the title compound as a white foam (65.3 mg, 95% yield, 1.64:1 rotamers). IR ν_{max}/cm^{-1} (film): 2978, 1697, 1368, 1306, 1159, 1086, 734, 689; ^{1}H NMR (400 MHz, $CDCl_3$) δ: major one: 8.65 (br, 1H), 7.97-7.88 (m, 2H), 7.70-7.63 (m, 1H), 7.62-7.53 (m, 2H), 4.34-4.23 (m, 1H), 3.48 (d, *J* = 13.2 Hz, 1H), 3.10-2.91 (m, 1H), 2.23-1.81 (m, 6H), 1.31 (s, 9H), 0.77 (t, *J* = 7.5 Hz, 3H), minor one: 8.65 (br, 1H), 7.97-7.88 (m, 2H), 7.70-7.63 (m, 1H), 7.62-7.53 (m, 2H), 4.44-4.34 (m, 1H), 3.76 (dd, *J* = 13.5, 1.9 Hz, 1H), 3.10-2.91 (m, 1H), 2.23-1.81 (m, 6H), 1.33 (s, 9H), 0.80 (t, *J* = 7.5 Hz, 3H); ^{13}C NMR (100 MHz, $CDCl_3$) δ: major one: 180.6, 154.4, 139.7, 134.2, 129.7, 128.2, 82.0, 70.2, 58.8, 55.3, 32.3, 28.4, 26.5, 26.1, 8.3, minor one: 178.1, 153.6, 139.7, 133.9, 129.5, 128.1, 81.1, 69.0, 58.2, 55.4, 34.1, 28.3, 26.8, 26.1, 8.2; m/z HRMS (ESI) found [M-H]$^-$ 396.1477, $C_{19}H_{26}NO_6S$ requires 396.1486.

6. Kinetic Isotope Effects

KIE Determined from Two Parallel Reactions

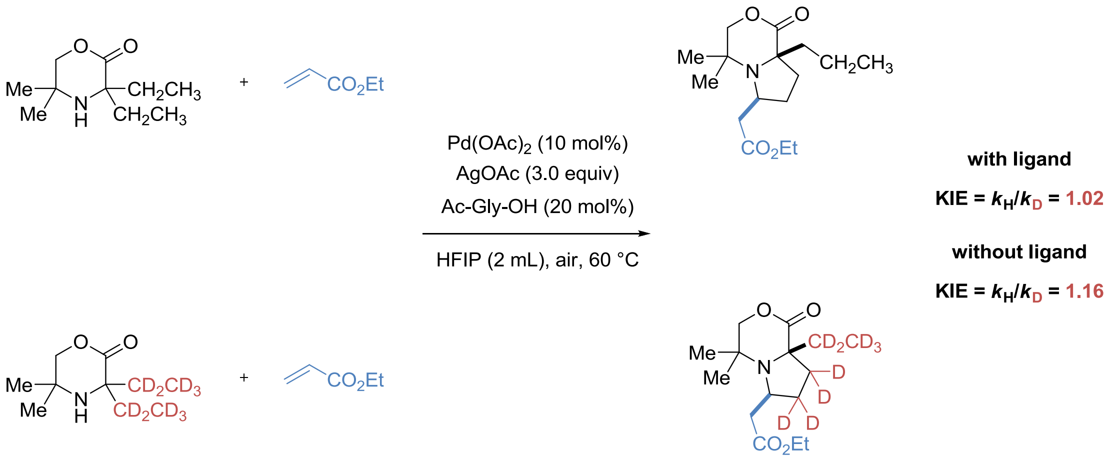

According to the general procedure, 3,3-diethyl-5,5-dimethylmorpholin-2-one (18.5 mg, 0.1 mmol) or 3,3-diethyl-5,5-dimethylmorpholin-2-one (d_5, d_5) (19.5 mg, 0.1 mmol), ethyl acrylate (20.0 mg, 0.2 mmol), $Pd(OAc)_2$ (2.2 mg, 0.01 mmol), with or without ligand Ac-Gly-OH (2.3 mg, 0.02 mmol), and AgOAc (50.1 mg, 0.3 mmol) were stirred in hexafluoroisopropanol (2 mL) at 60 ℃. Aliquots were taken at the indicated time intervals. The yield was determined by ^1H NMR using triphenylmethane as the internal standard.

		15 min	30 min	45 min	60 min	75min	90min
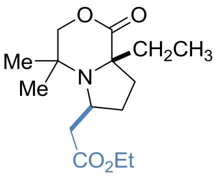	with ligand	**11%**	**15%**	**19%**	**23%**	**26%**	**30%**
	NMR yields						
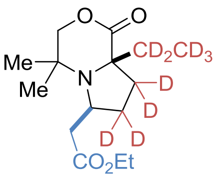	with ligand	**10%**	**14%**	**18%**	**22%**	**24%**	**29%**

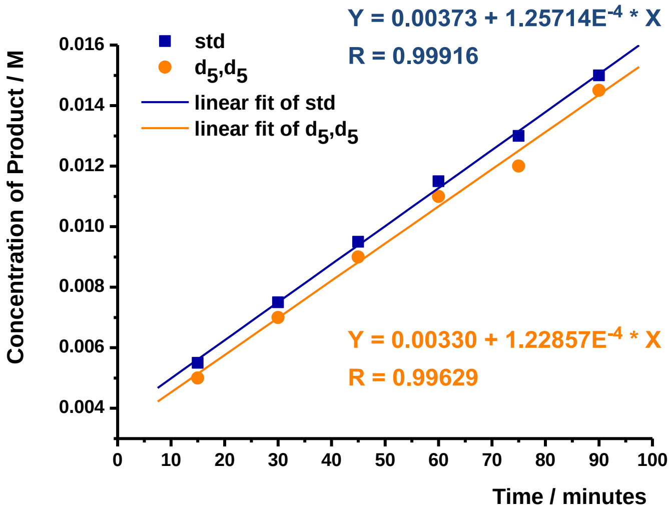

KIE = k_H/k_D = **1.02**

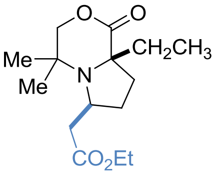

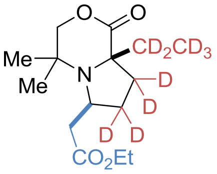

	15 min	30 min	45 min	60 min	75 min	90 min
without ligand	**9%**	**15%**	**18%**	**21%**	**25%**	**27%**
NMR yields						
without ligand	**8%**	**13%**	**16%**	**18%**	**21%**	**24%**

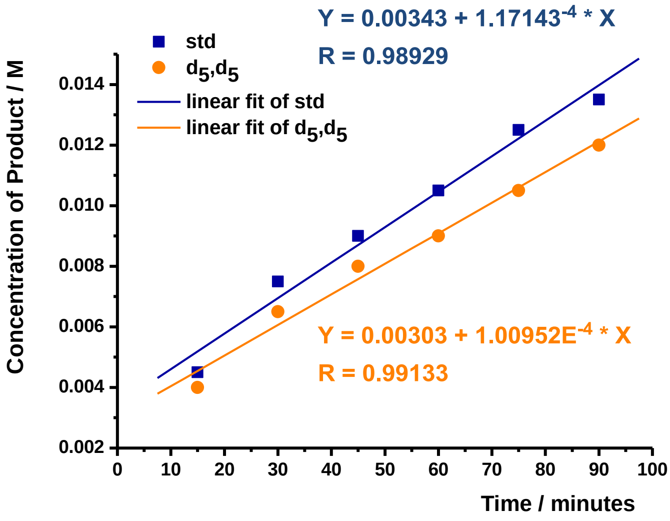

KIE = k_H/k_D = **1.16**

Ethyl 2-(8a-ethyl-4,4-dimethyl-1-oxohexahydro-1*H*-pyrrolo[2,1-*c*][1,4] oxazin-6-yl)acetate (d_5, d_5) (d_{10}-5b)

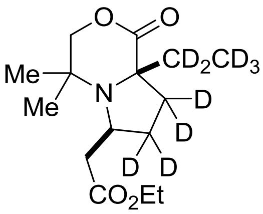

IR ν_{max}/cm^{-1} (film): 2976, 1729, 1471, 1373, 1282, 1176, 1070; ^{1}H NMR (400 MHz, $CDCl_3$) δ: 4.21-4.07 (m, 3H), 3.86 (d, *J* = 10.7 Hz, 1H), 3.66 (dd, *J* = 9.5, 4.8 Hz, 1H), 2.59 (dd, *J* = 15.0, 4.8 Hz, 1H), 2.31 (dd, *J* = 15.0, 9.5 Hz, 1H), 1.26 (t, *J* = 7.1 Hz, 3H), 1.12 (s, 3H), 1.08 (s, 3H); ^{13}C NMR (125 MHz, $CDCl_3$) δ: 175.1, 171.9, 77.0, 70.2, 60.6, 56.9, 53.1, 45.0, 35.4 (qn, *J* = 20.2 Hz), 34.8 (qn, *J* = 19.5 Hz), 28.7 (qn, *J* = 19.7 Hz), 26.1, 18.4, 14.4, 8.2 (sp, *J* = 18.7 Hz); m/z HRMS (ESI) found $[M+H]^+$ 293.2421, $C_{15}H_{17}D_9NO_4$ requires 293.2421.

KIE Determined from an Intermolecular Competition

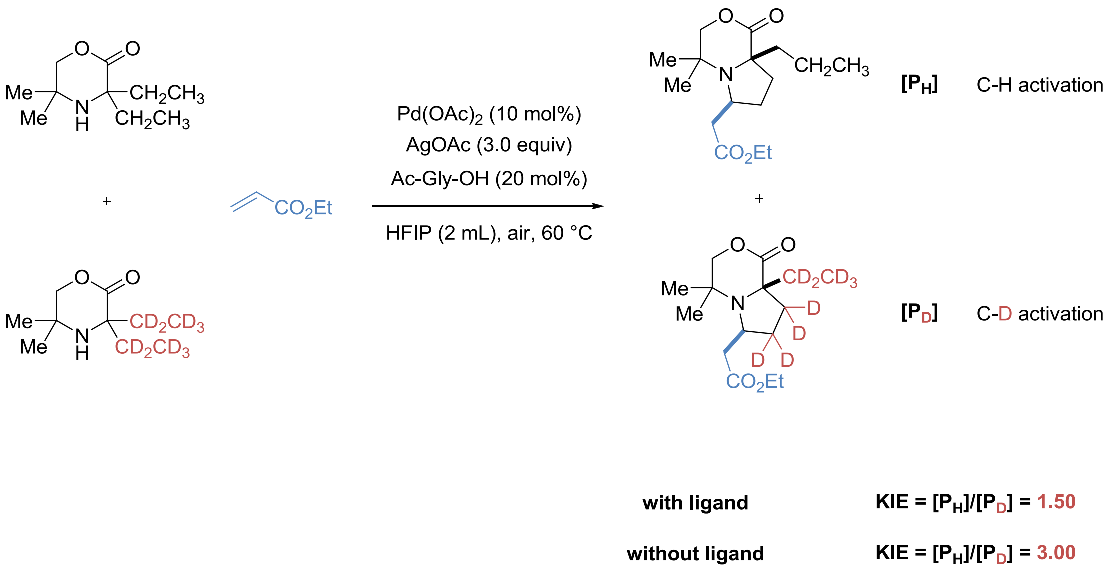

According to the general procedure, 3,3-diethyl-5,5-dimethylmorpholin-2-one (18.5 mg, 0.1 mmol) and 3,3-diethyl-5,5-dimethylmorpholin-2-one (d_5, d_5) (19.5 mg, 0.1

mmol), ethyl acrylate (10.0 mg, 0.1 mmol), $Pd(OAc)_2$ (2.2 mg, 0.01 mmol), with or without ligand Ac-Gly-OH (2.3 mg, 0.02 mmol), and AgOAc (50.1 mg, 0.3 mmol) were stirred in hexafluoroisopropanol (2 mL) at 60 ℃ for 24 hours. Then the reaction mixture was cooled to room temperature, filtered through celite, eluting with ethyl acetate, and concentrated *in vacuo*. The crude material was purified by flash column chromatography (70/30 40-60 ℃ Petroleum Ether/EtOAc) to provide the mixed alkenylation products for ^1H NMR analysis.

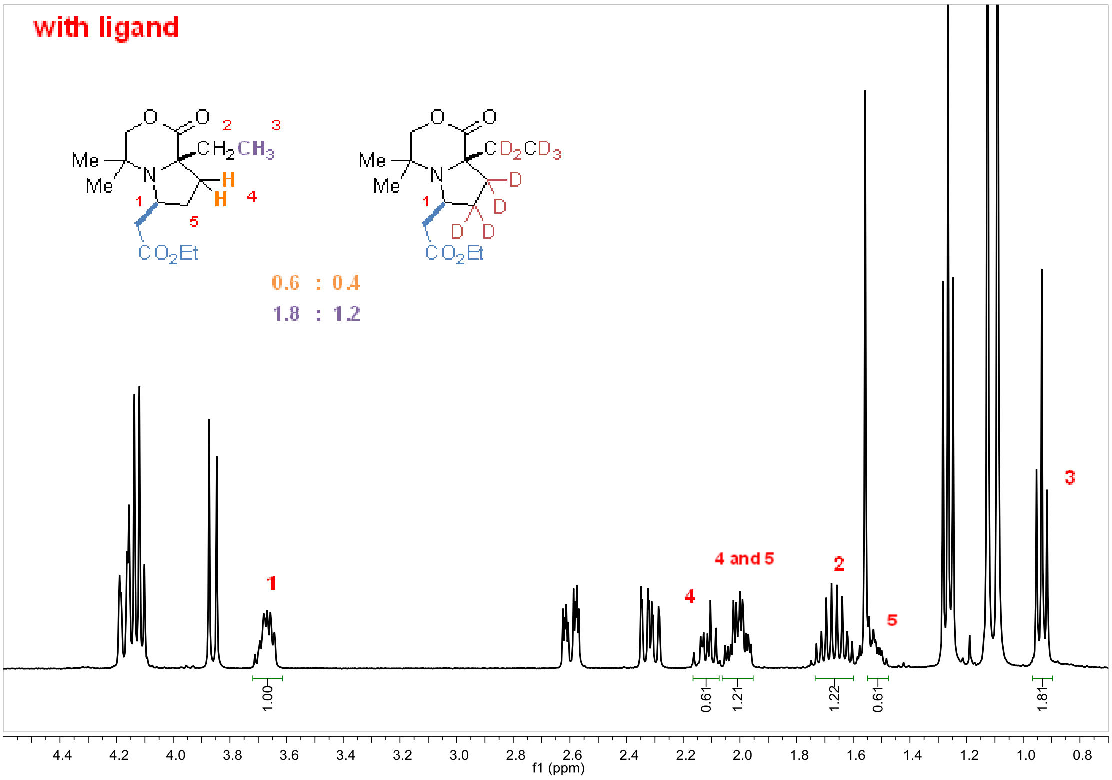

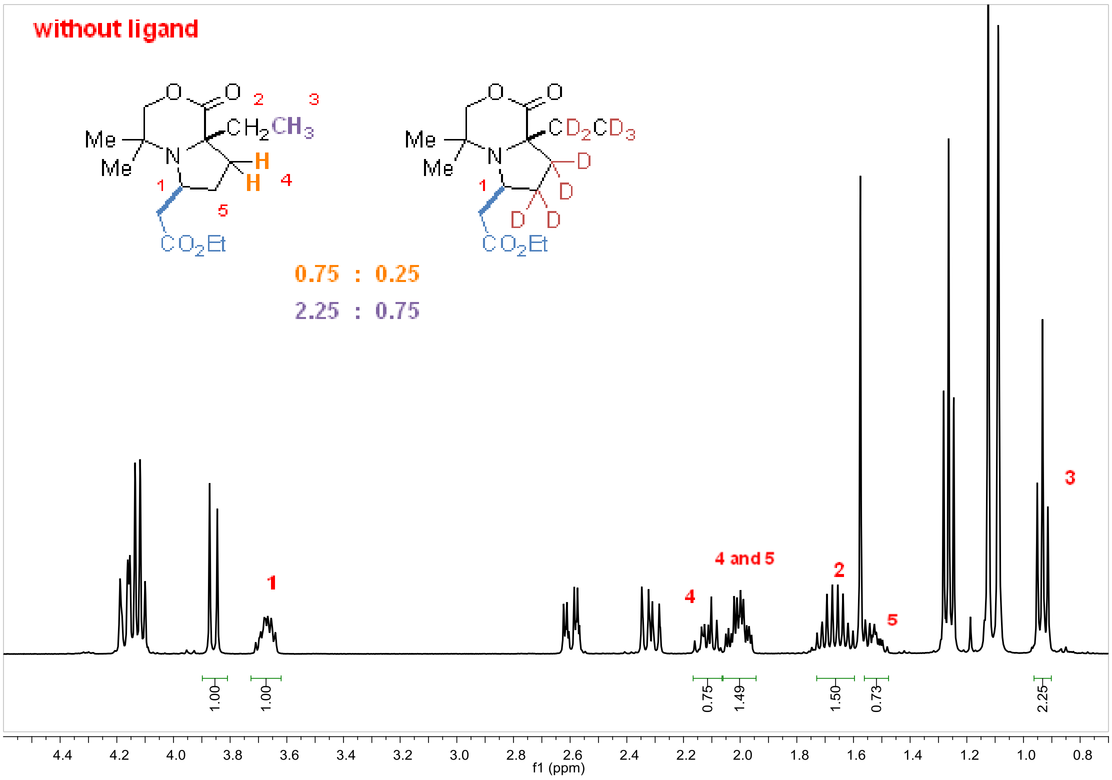

KIE Determined from an Intramolecular Competition

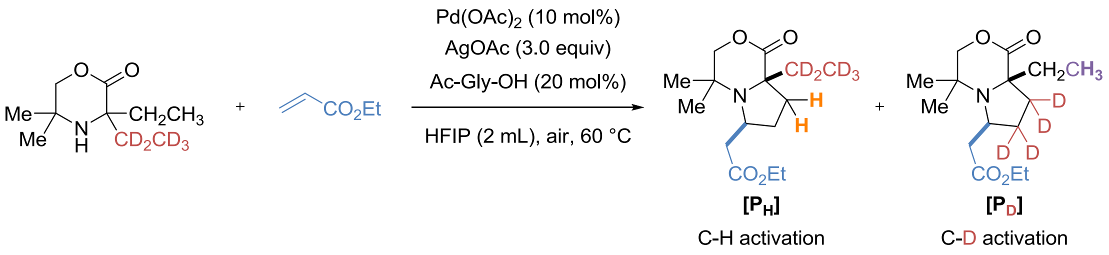

with ligand KIE = $[P_H]/[P_D]$ = 1.42

without ligand KIE = $[P_H]/[P_D]$ = 2.41

According to the general procedure, 3,3-diethyl-5,5-dimethylmorpholin-2-one (d_5) (19.0 mg, 0.1 mmol), ethyl acrylate (20.0 mg, 0.2 mmol), $Pd(OAc)_2$ (2.2 mg, 0.01 mmol), with or without ligand Ac-Gly-OH (2.3 mg, 0.02 mmol), and AgOAc (50.1 mg, 0.3 mmol) were stirred in hexafluoroisopropanol (2 mL) at 60 ℃ for 24 hours. Then the reaction mixture was cooled to room temperature, filtered through celite, eluting with ethyl acetate, and concentrated *in vacuo*. The crude material was purified by flash column chromatography (70/30 40-60 ℃ Petroleum Ether/EtOAc) to provide the mixed alkenylation products for ^1H NMR analysis.

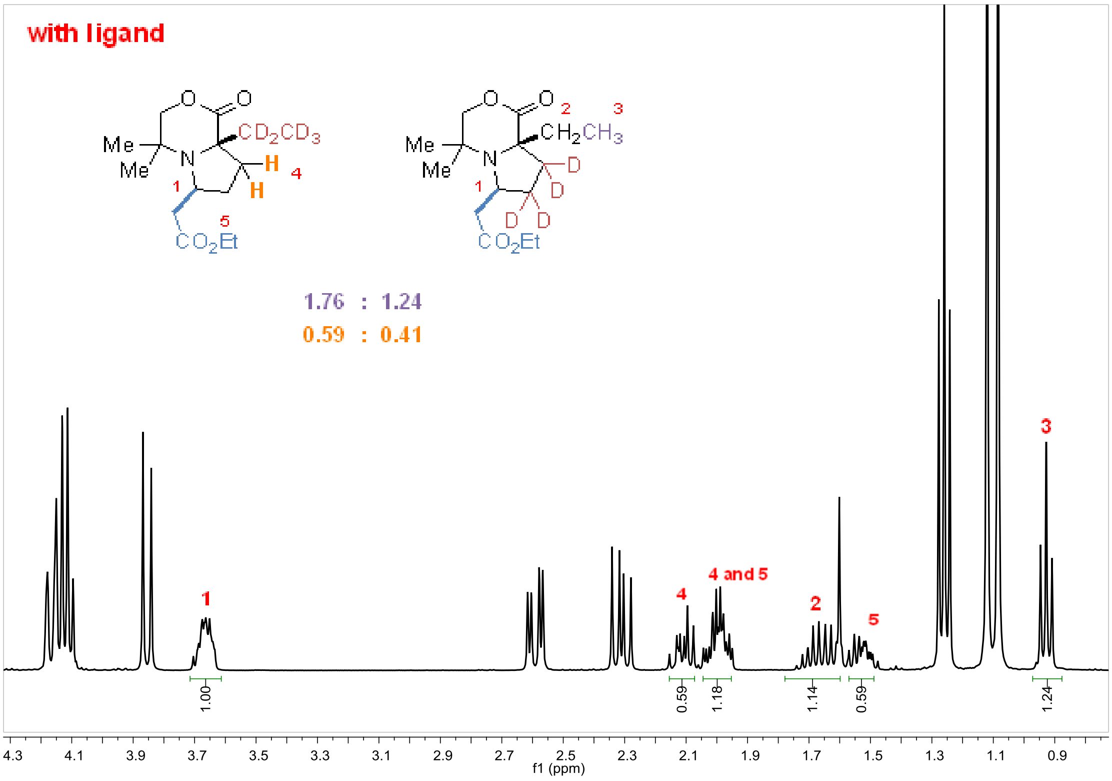
with ligand
Me
Me
N
O
O
CD2CD3
H
H
4
1
5
CO2Et
2
3
CH2CH3
D
D
D
D
1.76 : 1.24
0.59 : 0.41
1
4
4 and 5
2
5
3
1.00
0.59
1.18
1.14
0.59
1.24
f1 (ppm)

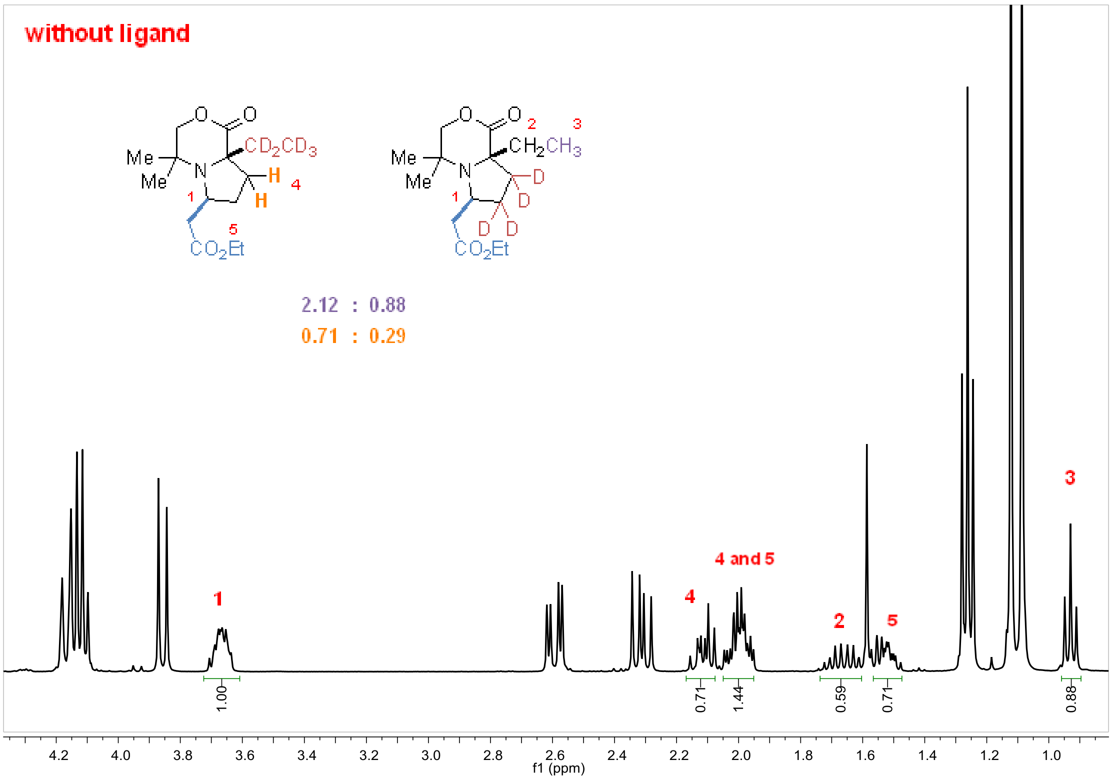
without ligand
Me
Me
N
O
O
CD2CD3
H
H
4
1
5
CO2Et
2
3
CH2CH3
D
D
D
D
2.12 : 0.88
0.71 : 0.29
1
4
4 and 5
2
5
3
1.00
0.71
1.44
0.59
0.71
0.88
f1 (ppm)

7. Proposed Mechanism

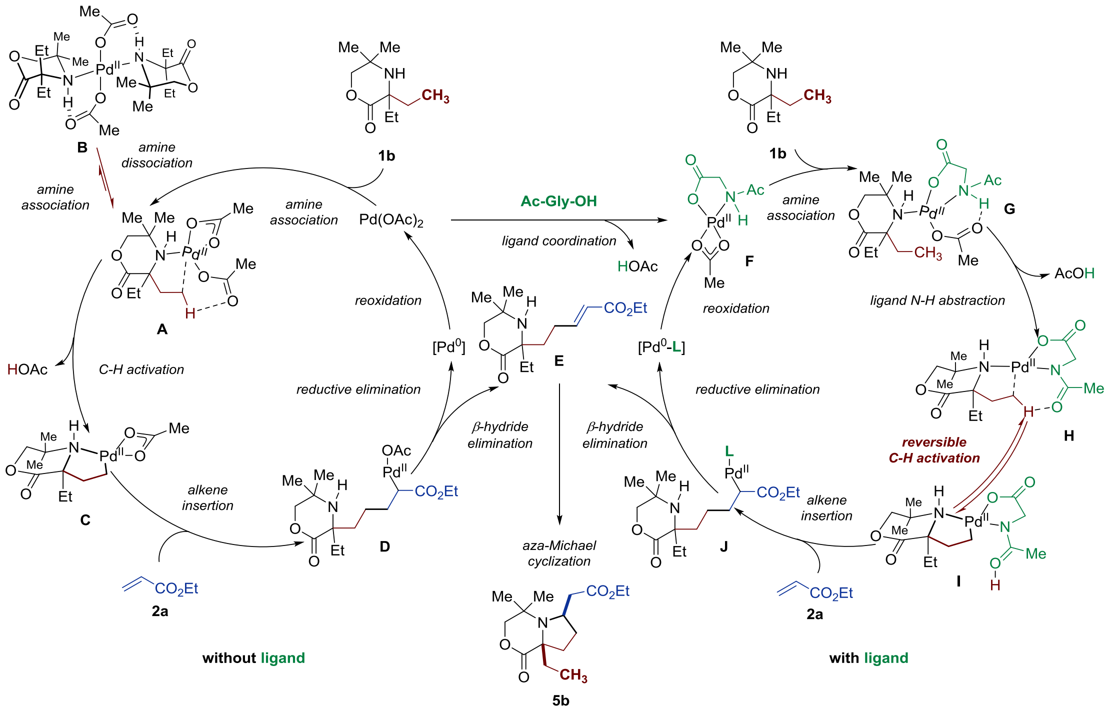

Figure S2. Proposed mechanism of the C–H alkenylation with and without ligand.

Based on the preliminary mechanistic studies, a proposed Pd(II)/Pd(0) catalytic cycle of this C–H alkenylation is outlined in Figure S2. Firstly, in the absence of amino acid ligand, the amine association with $Pd(OAc)_2$ forms the bis-amine Pd(II)-complex **B**. Owing to the steric hindrance around the Pd(II) center, this weaker binding would therefore facilitate the release of one of the amines to create the essential vacant coordination site, giving the mono-amine Pd(II) complex **A** which can undergo subsequent C–H activation. And this amine dissociation process is probably the rate-determining step. Then C–H activation takes place affording the key five-membered-Pd-cycle **C**, which is an irreversible step. After that, coordination and insertion of alkene into the palladacycle gives the intermediate **D**, followed by the β-hydride elimination providing the transient alkenylation product **E** which would further undergo aza-Michael cyclization to give the pyrrolidine product. Finally, reductive elimination from Pd(II) hydride gives Pd(0) species, which can be oxidized by Ag(I) salt to regenerate the active Pd (II) catalyst. While in the presence of amino

acid ligand, the process begins with ligand coordination and amine association, giving the precatalyst **G**. Based on the recent mechanistic studies by Wu, Houk, Yu and Musaev,[3] the precatalyst **G** would undergo the "ligand N−H bond cleavage and subsequent C−H activation". The amino acid here acts as a weakly coordinated monoanionic ligand that stabilizes the precatalyst **G** as well as a soft electron donor (from the *N*-terminus) and bidentately coordinated dianionic ligand after the N−H abstraction that forms the catalytically active species **H**. This bidentately coordinated dianionic ligand in intermediate **H** may act as the proton acceptor for the C−H activation via an inner-sphere concerted metalation deprotonation (CMD) mechanism giving the Pd-cycle species **I**, which is a reversible process. The rate-determining step may be the ligand N−H bond abstraction that occurs right before the C−H activation. Then similar alkene coordination/insertion takes place, followed by β-hydride elimination providing the transient alkenylation product **E**, which would also further undergo aza-Michael cyclization to provide the final pyrrolidine product.

8. Asymmetric C−H Alkenylation Attempt

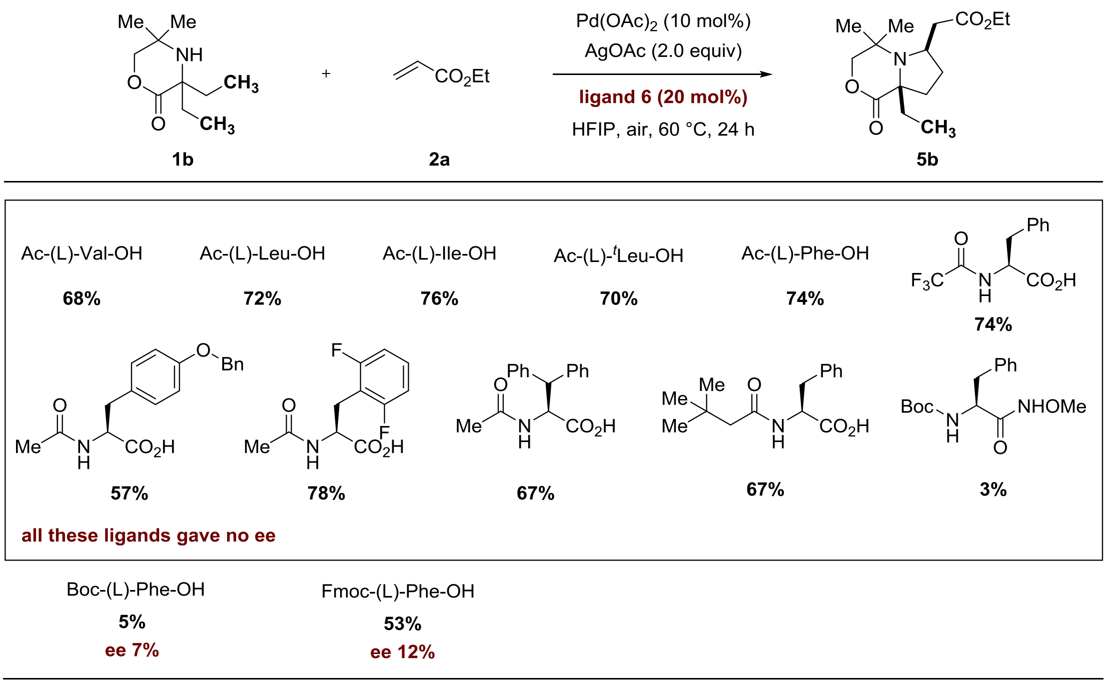

Figure S3. Asymmetric C–H alkenylation attempt.

9. References

1. D. D. Perrin and W. L. F. Armarego, Purification of Laboratory Chemicals, Pergamon Press, Oxford, 1997.

2. A. McNally, B. Haffemayer, B. S. L. Collins and M. J. Gaunt, *Nature*, 2014, **510**, 129-133.

3. (a) G.-J. Cheng, Y.-F. Yang, P. Liu, P. Chen, T.-Y. Sun, G. Li, X. Zhang, K. N. Houk, J.-Q. Yu and Y.-D. Wu, *J. Am. Chem. Soc.*, 2014, **136**, 894-897; (b) D. G. Musaev, T. M. Figg and A. L. Kaledin, *Chem. Soc. Rev.*, 2014, **43**, 5009-5031; (c) B. E. Haines and D. G. Musaev, *ACS Catal.*, 2015, **5**, 830-840.

10. NMR Spectra

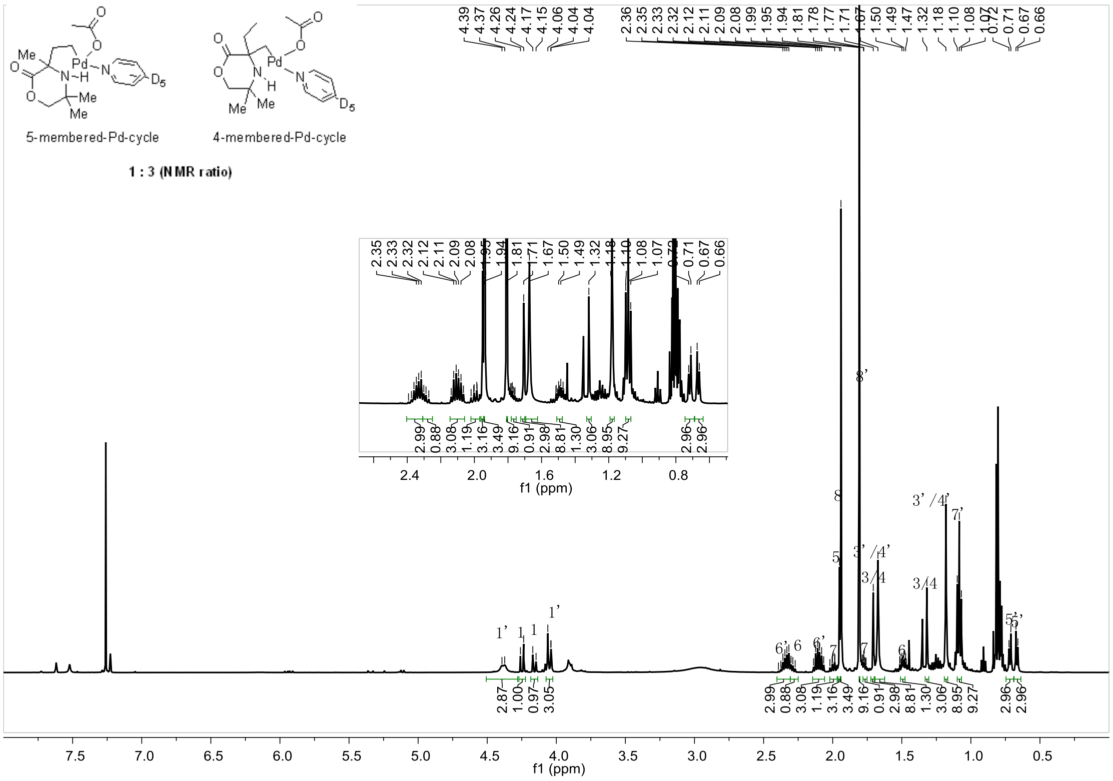

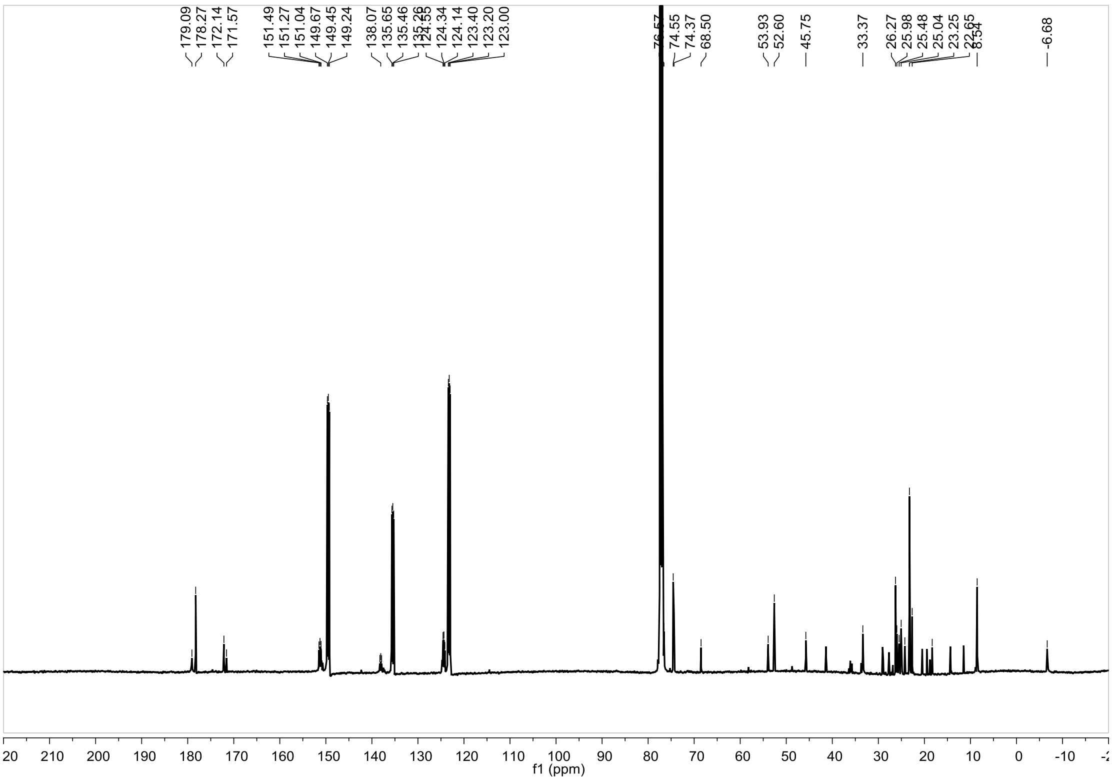

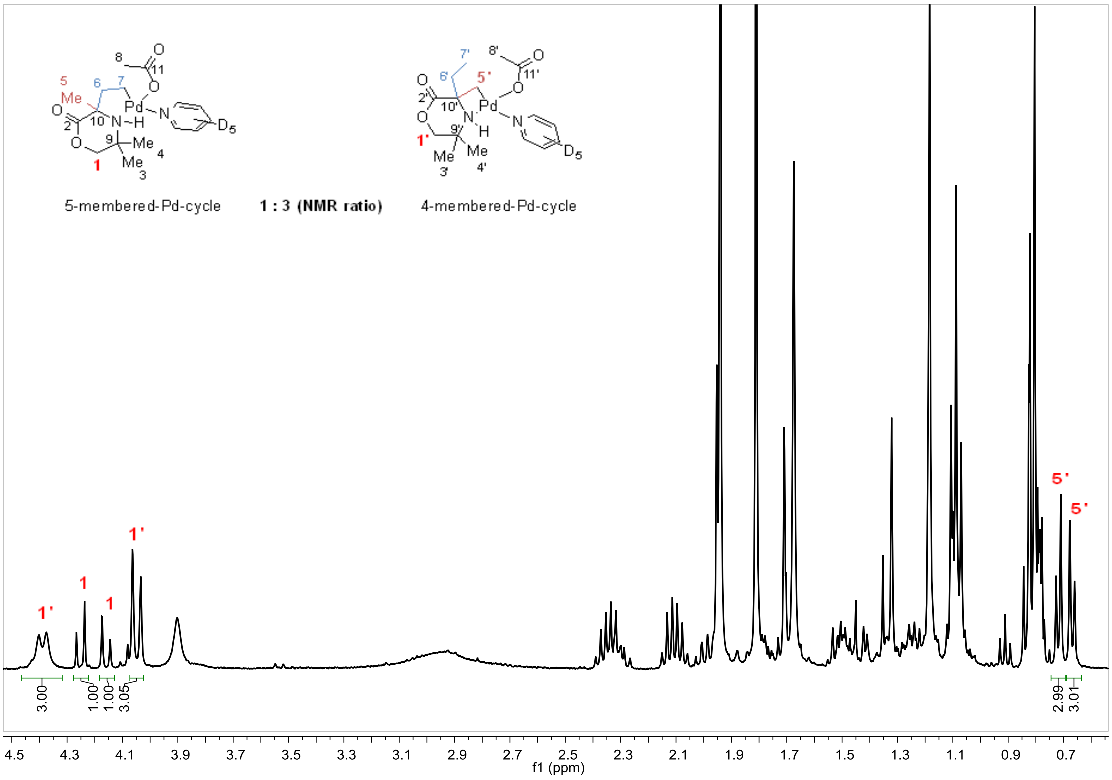
5-membered-Pd-cycle
1 : 3 (NMR ratio)
4-membered-Pd-cycle
f1 (ppm)

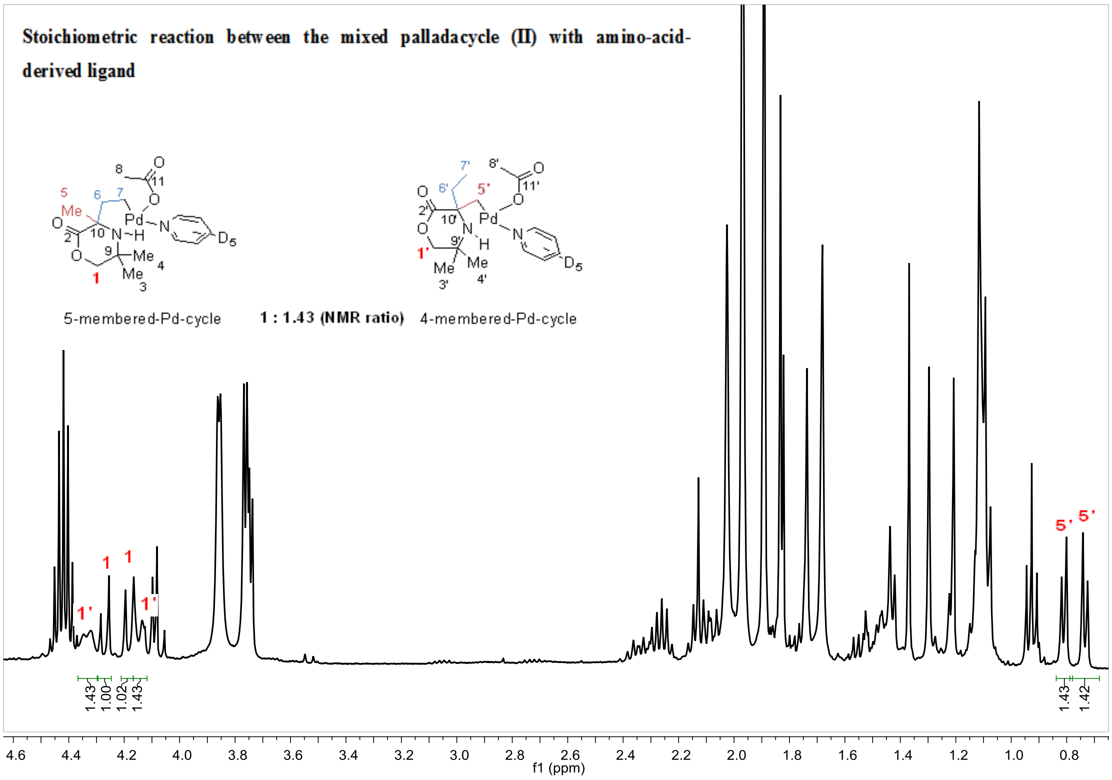
Stoichiometric reaction between the mixed palladacycle (II) with amino-acid-derived ligand
5-membered-Pd-cycle
1 : 1.43 (NMR ratio)
4-membered-Pd-cycle
f1 (ppm)

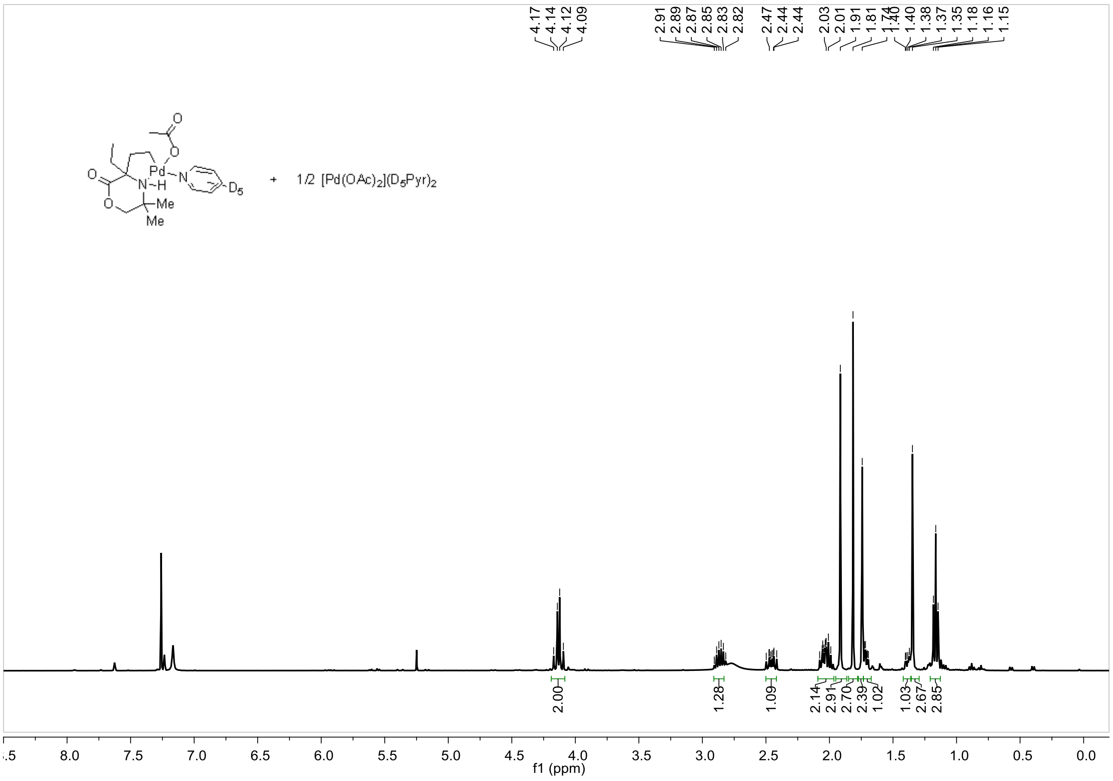
4.17
4.14
4.12
4.09
2.91
2.89
2.87
2.85
2.83
2.82
2.47
2.44
2.44
2.03
2.01
1.91
1.81
1.40
1.38
1.37
1.35
1.18
1.16
1.15
+ 1/2 [Pd(OAc)2](D5Pyr)2
2.00
1.28
1.09
2.14
2.91
2.70
2.39
1.02
1.03
2.67
2.85
f1 (ppm)

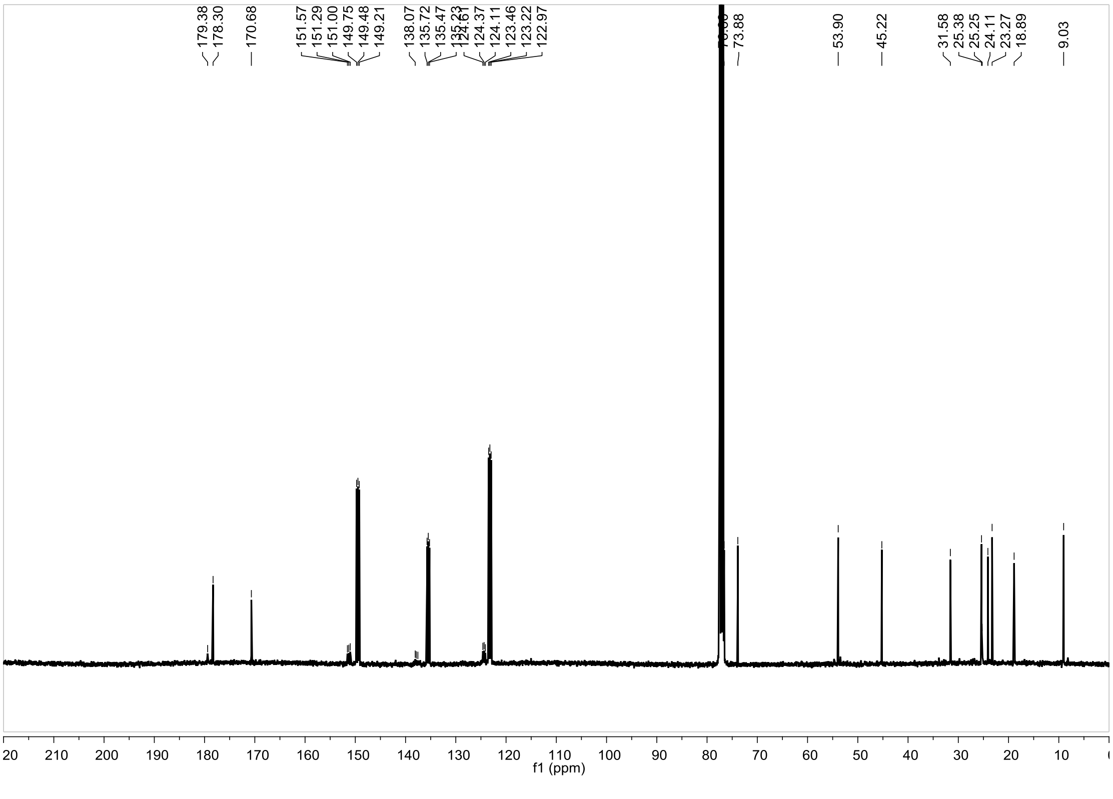
179.38
178.30
170.68
151.57
151.29
151.00
149.75
149.48
149.21
138.07
135.72
135.47
124.37
124.11
123.46
123.22
122.97
73.88
53.90
45.22
31.58
25.38
25.25
24.11
23.27
18.89
9.03
f1 (ppm)

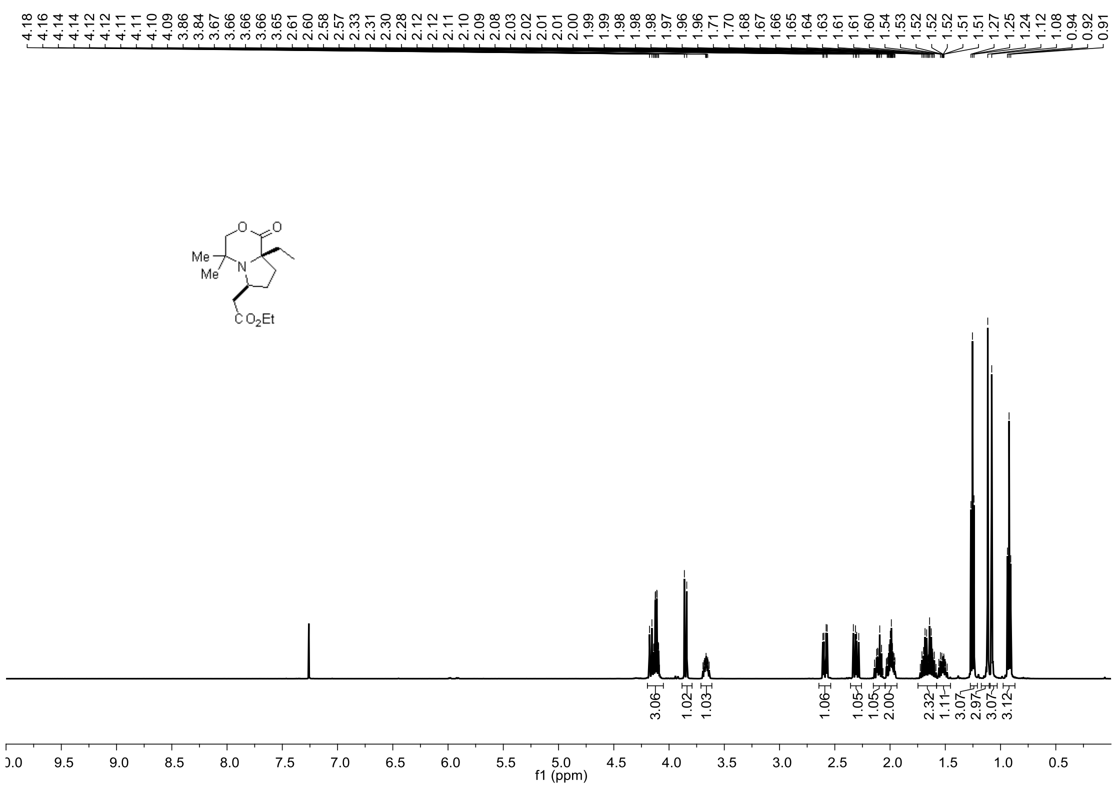
4.18
4.16
4.14
4.14
4.12
4.12
4.11
4.11
4.10
4.09
3.86
3.84
3.67
3.66
3.66
3.66
3.65
2.61
2.60
2.58
2.57
2.33
2.31
2.30
2.28
2.12
2.12
2.11
2.10
2.09
2.08
2.03
2.02
2.01
2.01
2.00
1.99
1.99
1.98
1.98
1.98
1.97
1.96
1.96
1.71
1.70
1.68
1.67
1.66
1.65
1.64
1.63
1.61
1.61
1.60
1.54
1.53
1.52
1.52
1.52
1.51
1.51
1.27
1.25
1.24
1.12
1.08
0.94
0.92
0.91
Me
Me
N
O
O
CO2Et
3.06
1.02
1.03
1.06
1.05
1.05
2.00
2.32
1.11
3.07
2.97
3.07
3.12
f1 (ppm)

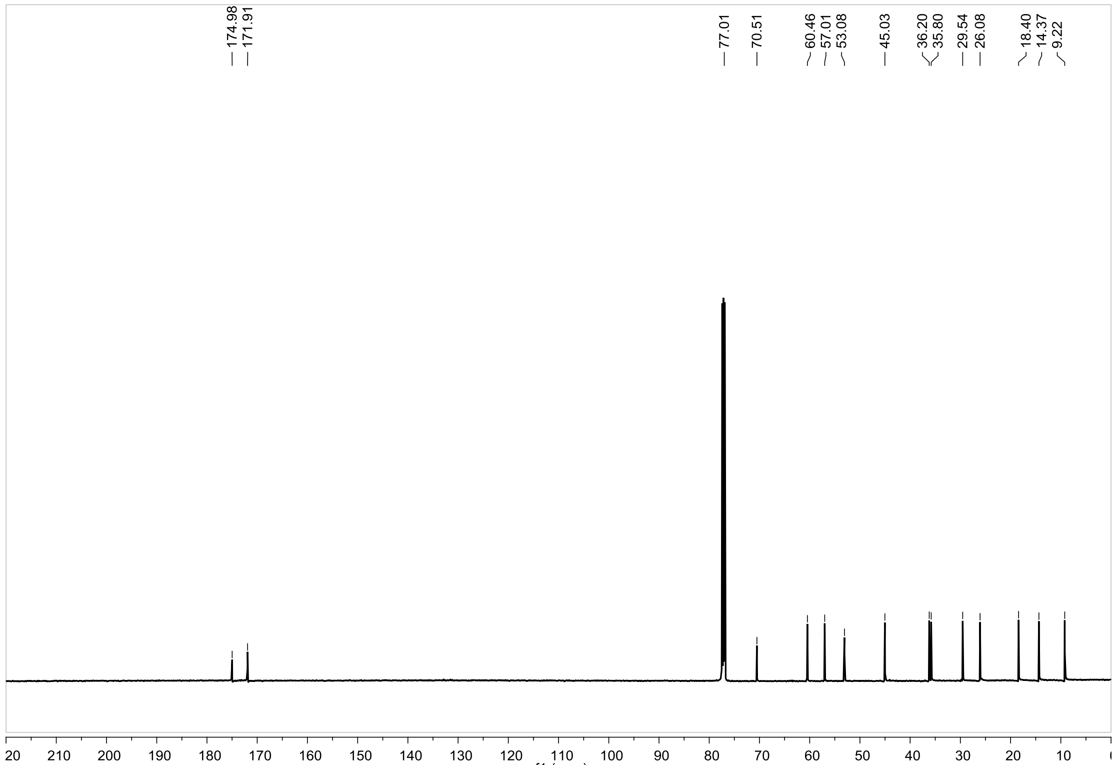
174.98
171.91
77.01
70.51
60.46
57.01
53.08
45.03
36.20
35.80
29.54
26.08
18.40
14.37
9.22
f1 (ppm)

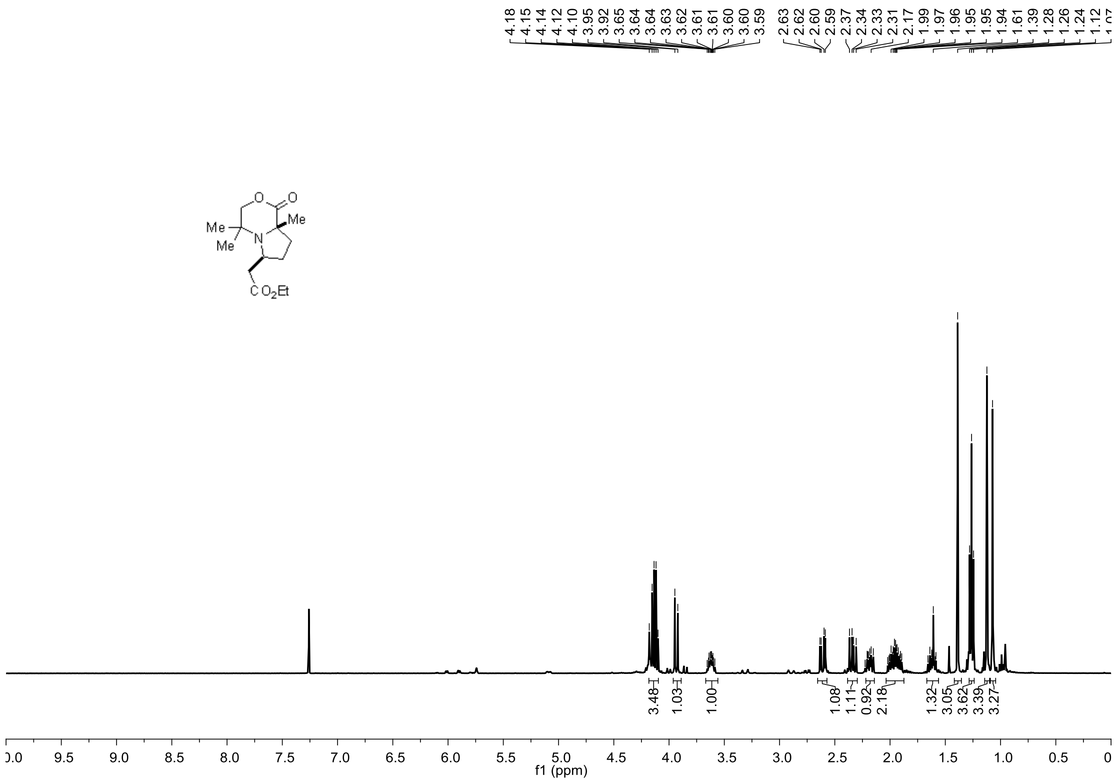
4.18 4.15 4.14 4.12 4.10 3.95 3.92 3.65 3.64 3.63 3.62 3.61 3.61 3.60 3.60 3.59 2.63 2.62 2.60 2.59 2.37 2.34 2.33 2.31 2.17 1.99 1.97 1.96 1.95 1.95 1.94 1.61 1.39 1.28 1.26 1.24 1.12
Me
Me
N
O
O
Me
CO_2Et
3.48 1.03 1.00 1.08 1.11 0.92 2.18 1.32 3.05 3.62 3.39 3.27
9.5 9.0 8.5 8.0 7.5 7.0 6.5 6.0 5.5 5.0 4.5 4.0 3.5 3.0 2.5 2.0 1.5 1.0 0.5 0
f1 (ppm)

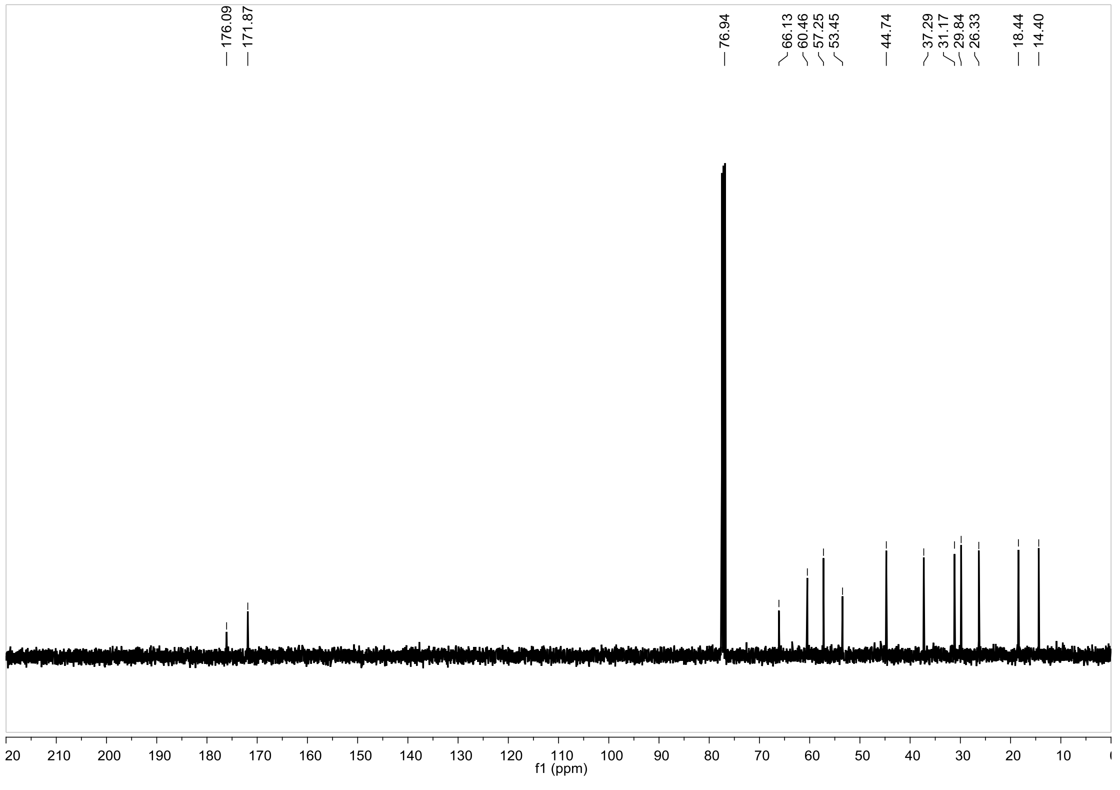
176.09 171.87 76.94 66.13 60.46 57.25 53.45 44.74 37.29 31.17 29.84 26.33 18.44 14.40
210 200 190 180 170 160 150 140 130 120 110 100 90 80 70 60 50 40 30 20 10
f1 (ppm)

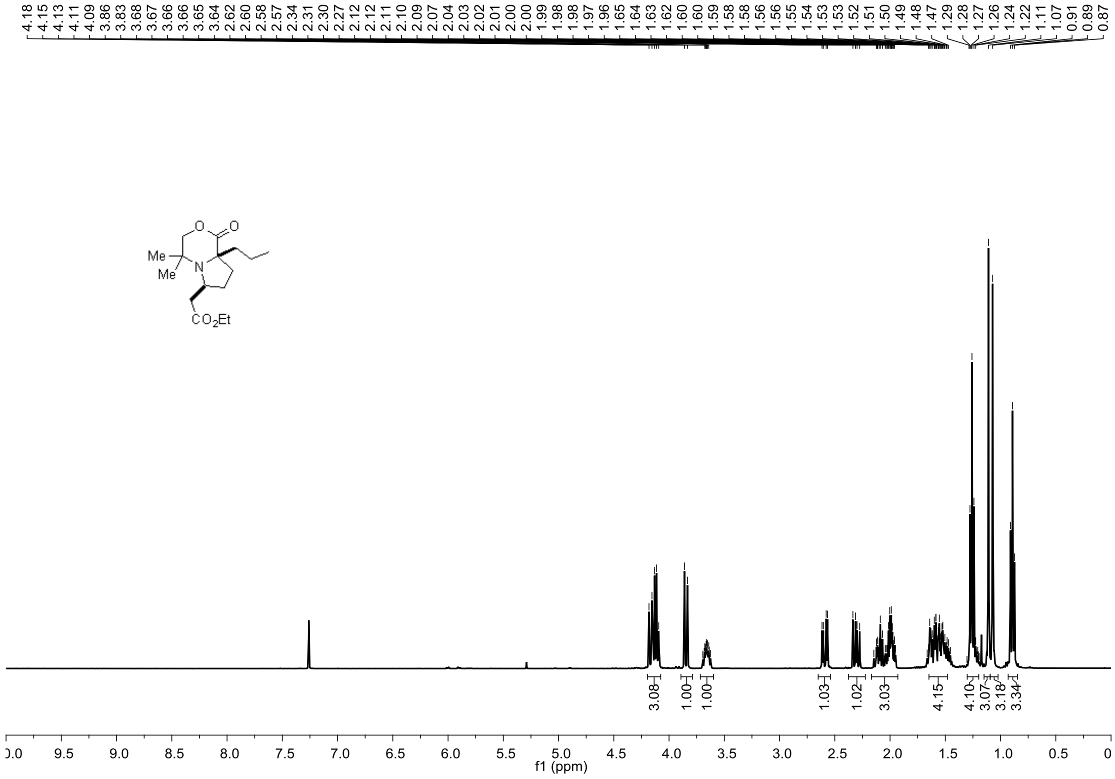
4.18
4.15
4.13
4.11
4.09
3.86
3.83
3.68
3.67
3.66
3.65
3.64
2.62
2.60
2.58
2.57
2.34
2.31
2.30
2.27
2.12
2.12
2.11
2.10
2.09
2.07
2.04
2.03
2.02
2.01
2.00
2.00
1.99
1.98
1.98
1.97
1.96
1.65
1.64
1.63
1.62
1.60
1.60
1.59
1.58
1.58
1.56
1.56
1.55
1.54
1.53
1.53
1.52
1.51
1.50
1.49
1.48
1.47
1.29
1.28
1.27
1.26
1.24
1.22
1.11
1.07
0.91
0.89
0.87
O
O
Me
Me
N
CO_2Et
3.08
1.00
1.00
1.03
1.02
3.03
4.15
4.10
3.07
3.18
3.34
f1 (ppm)

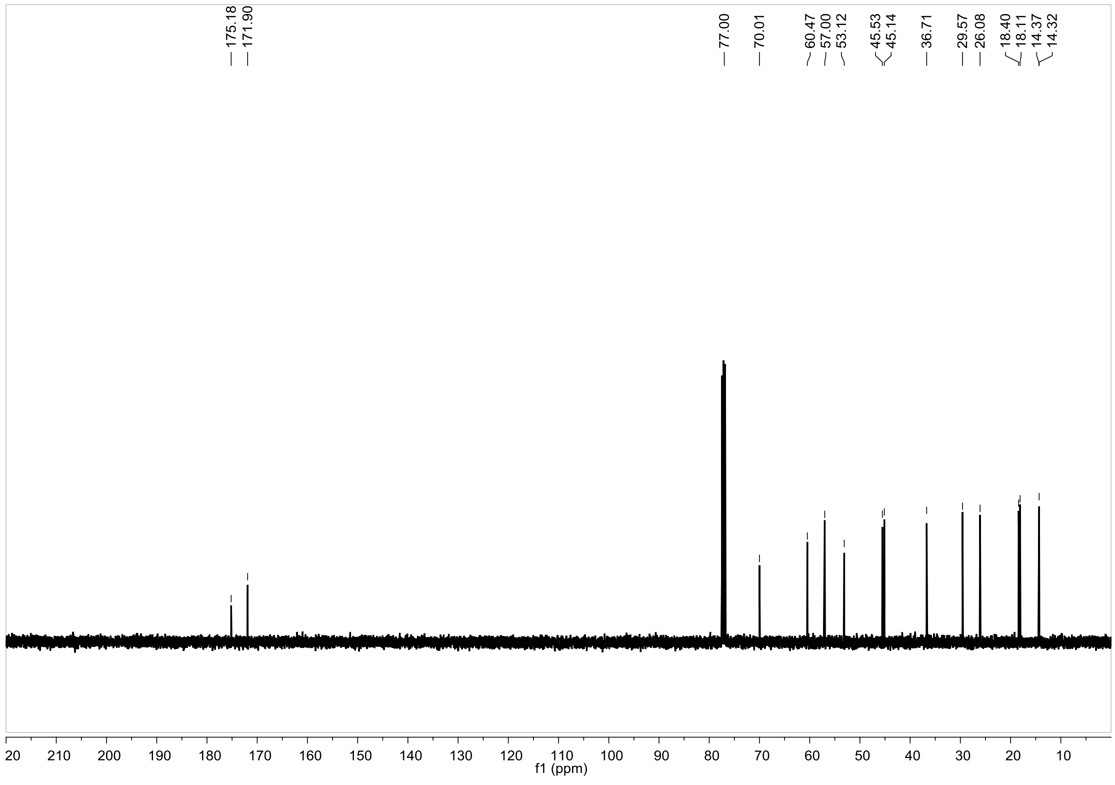
175.18
171.90
77.00
70.01
60.47
57.00
53.12
45.53
45.14
36.71
29.57
26.08
18.40
18.11
14.37
14.32
f1 (ppm)

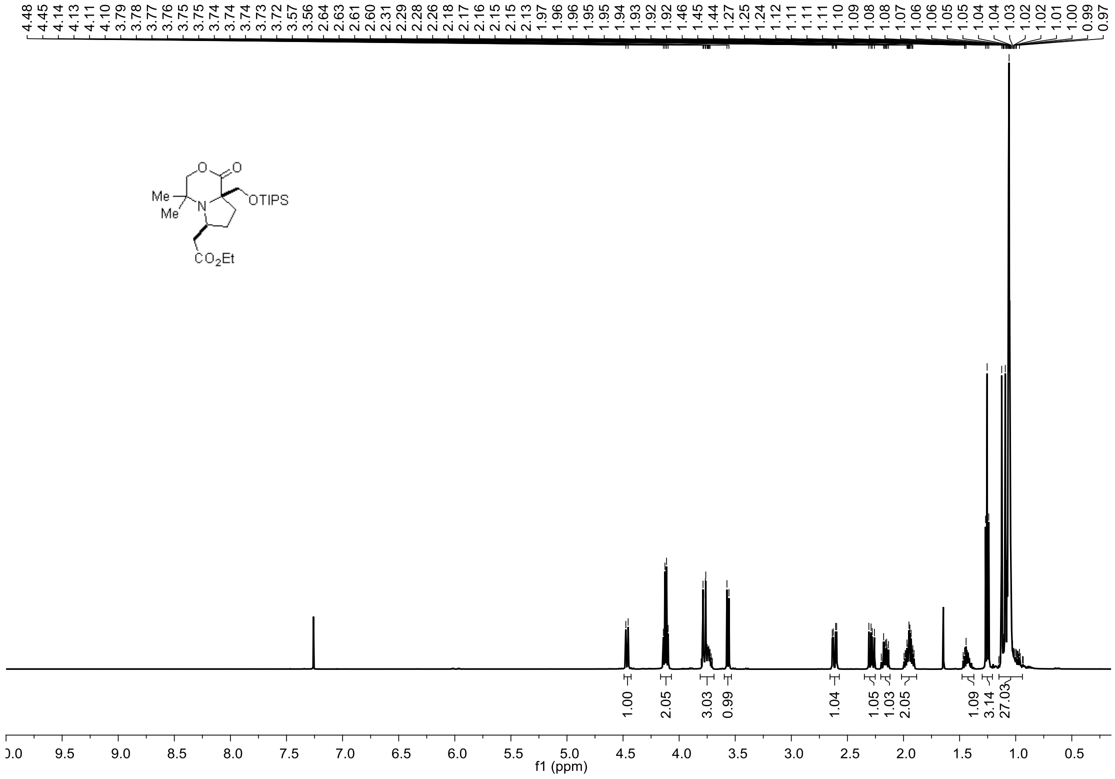
4.48 4.45 4.14 4.13 4.11 4.10 3.79 3.78 3.77 3.76 3.75 3.75 3.74 3.74 3.74 3.73 3.72 3.57 3.56 2.64 2.63 2.61 2.60 2.31 2.29 2.28 2.26 2.18 2.17 2.16 2.15 2.15 2.13 1.97 1.96 1.96 1.95 1.95 1.94 1.93 1.93 1.92 1.92 1.46 1.45 1.44 1.27 1.25 1.24 1.12 1.11 1.11 1.11 1.10 1.09 1.08 1.08 1.07 1.06 1.06 1.06 1.05 1.05 1.04 1.04 1.03 1.03 1.02 1.02 1.01 1.00 0.99 0.97
Me
Me
N
O
O
OTIPS
CO2Et
1.00 2.05 3.03 0.99 1.04 1.05 1.03 2.05 1.09 3.14 27.03
f1 (ppm)

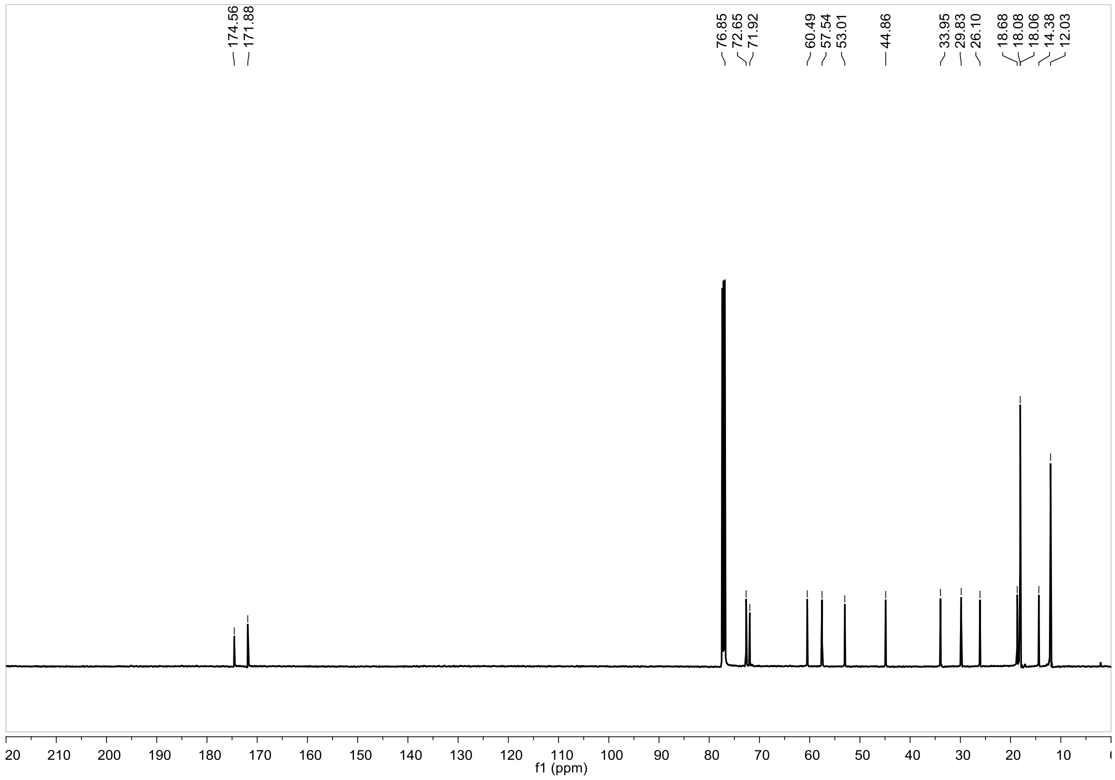
174.56 171.88
76.85 72.65 71.92
60.49 57.54 53.01
44.86
33.95 29.83 26.10
18.68 18.08 18.06 14.38 12.03
f1 (ppm)

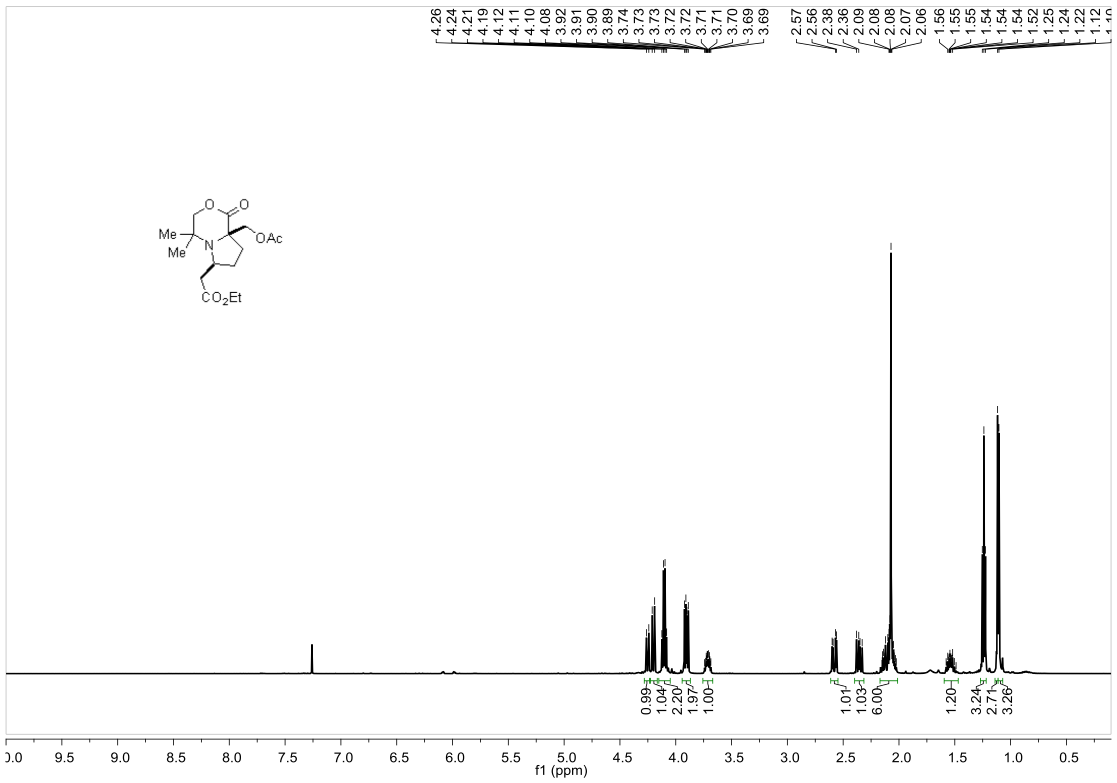
4.26
4.24
4.21
4.19
4.12
4.11
4.10
4.08
3.92
3.91
3.90
3.89
3.74
3.73
3.73
3.72
3.72
3.71
3.71
3.70
3.69
3.69
2.57
2.56
2.38
2.36
2.09
2.08
2.08
2.08
2.07
2.06
1.56
1.55
1.55
1.54
1.54
1.54
1.52
1.25
1.24
1.22
1.12
O
O
Me
Me
N
OAc
CO2Et
0.99
1.04
2.20
1.97
1.00
1.01
1.03
6.00
1.20
3.24
2.71
3.26
f1 (ppm)

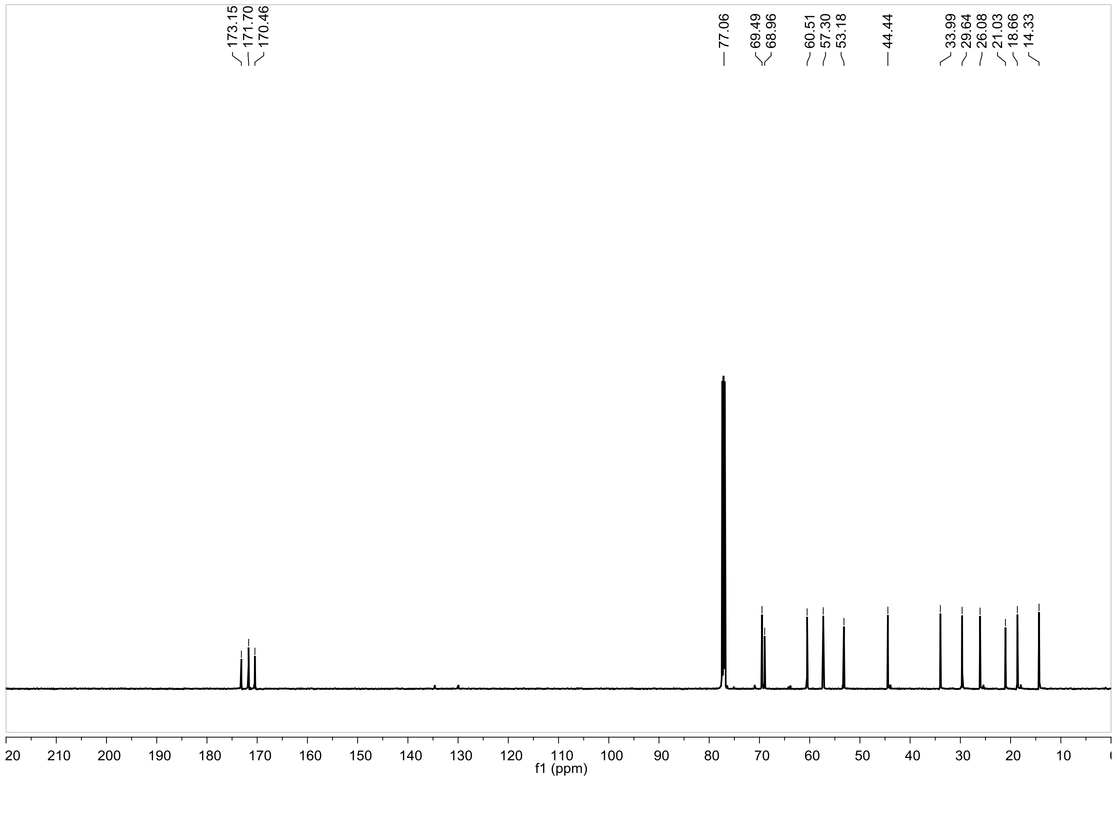
173.15
171.70
170.46
77.06
69.49
68.96
60.51
57.30
53.18
44.44
33.99
29.64
26.08
21.03
18.66
14.33
f1 (ppm)

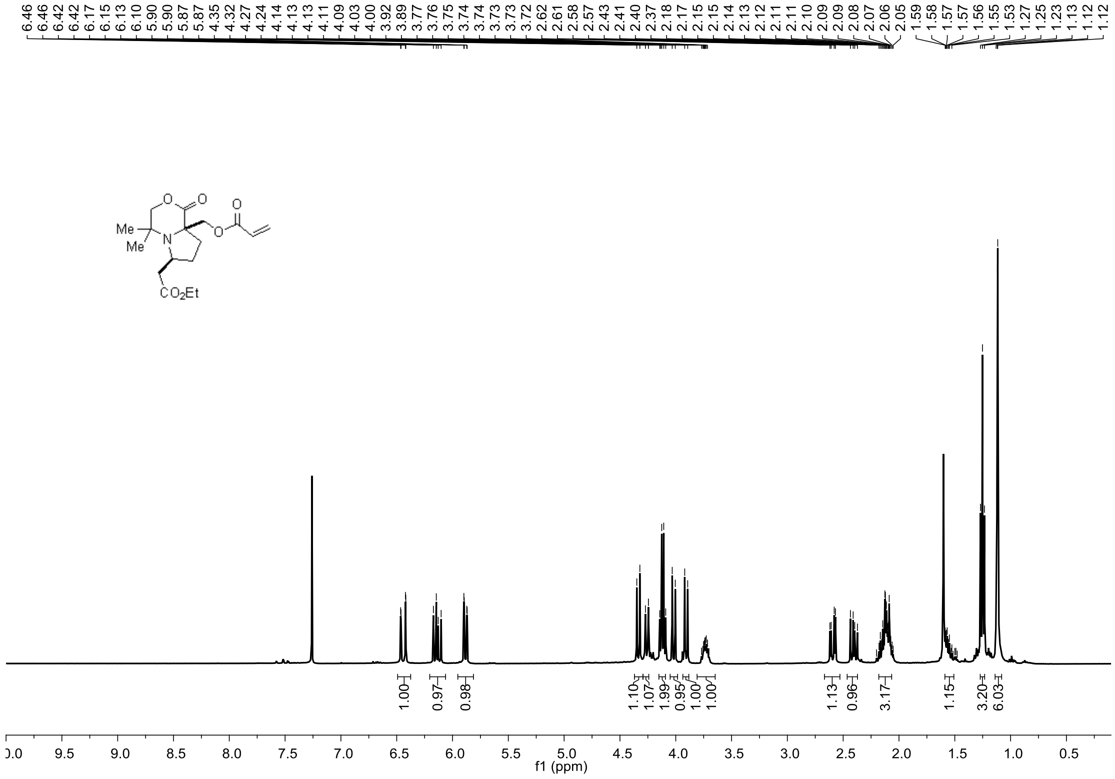
Me
Me
N
O
O
O
O
CO2Et
f1 (ppm)

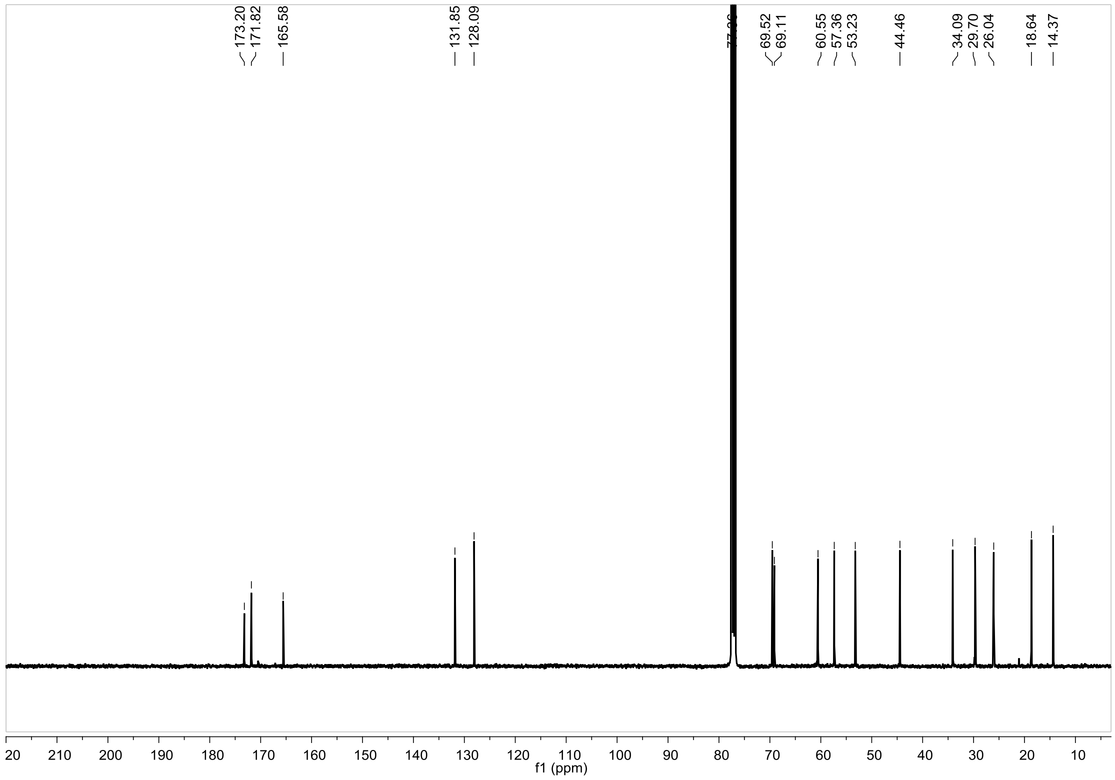
173.20
171.82
165.58
131.85
128.09
69.52
69.11
60.55
57.36
53.23
44.46
34.09
29.70
26.04
18.64
14.37
f1 (ppm)

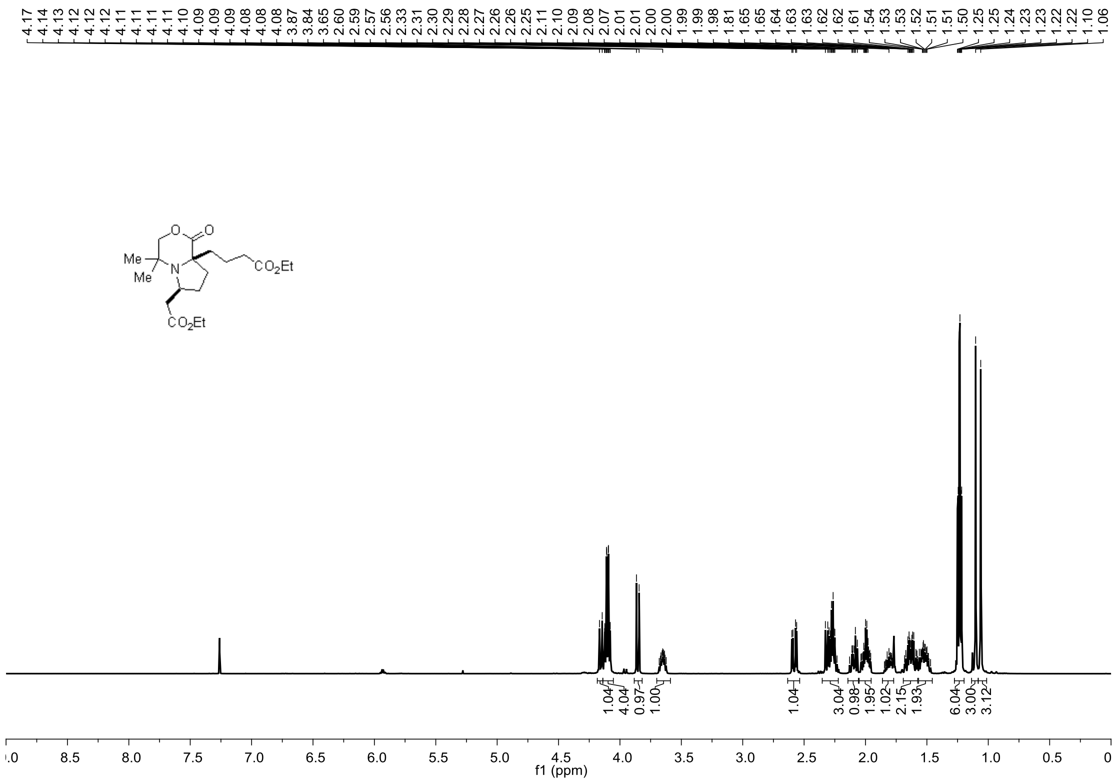
4.17 4.14 4.13 4.12 4.12 4.11 4.11 4.11 4.10 4.09 4.09 4.08 4.08 4.08 3.87 3.84 3.65 2.60 2.59 2.57 2.56 2.33 2.31 2.30 2.29 2.28 2.27 2.26 2.26 2.25 2.11 2.10 2.09 2.08 2.07 2.01 2.01 2.00 2.00 1.99 1.99 1.98 1.81 1.65 1.64 1.63 1.63 1.62 1.62 1.61 1.54 1.53 1.53 1.52 1.51 1.51 1.50 1.25 1.25 1.24 1.23 1.23 1.22 1.22 1.10 1.06
Me
Me
N
O
O
CO2Et
CO2Et
1.04 4.04 0.97 1.00 1.04 3.04 0.98 1.95 1.02 2.15 1.93 6.04 3.00 3.12
8.5 8.0 7.5 7.0 6.5 6.0 5.5 5.0 4.5 4.0 3.5 3.0 2.5 2.0 1.5 1.0 0.5 0
f1 (ppm)

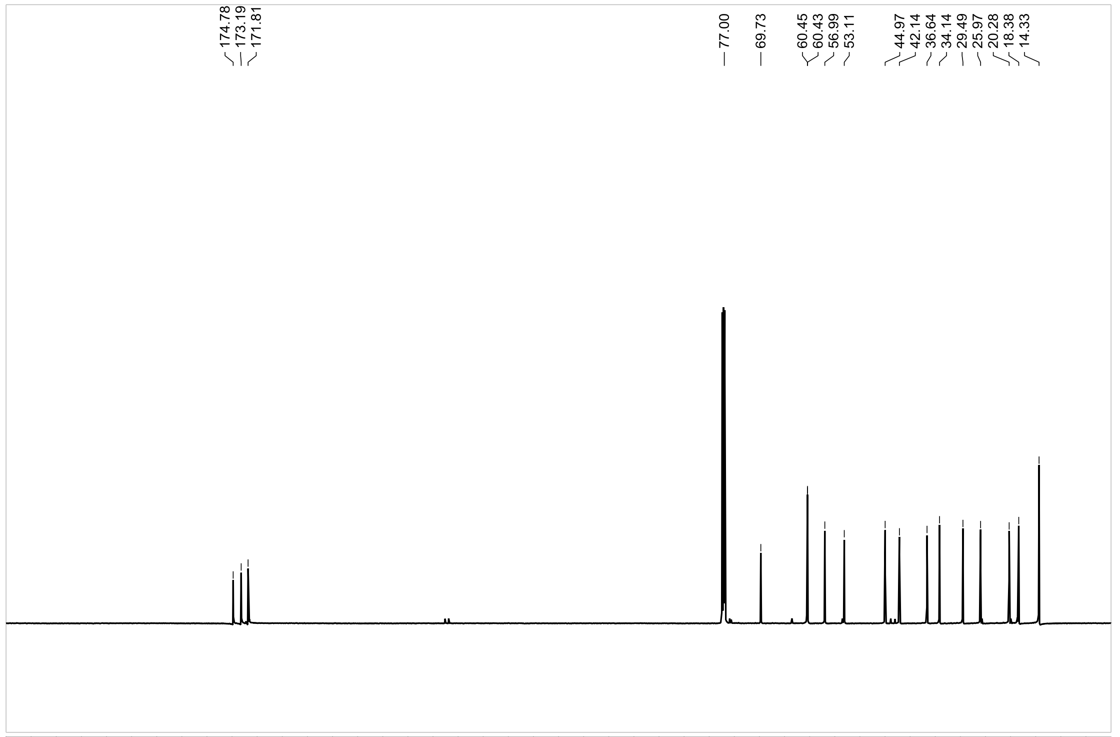
174.78 173.19 171.81
77.00
69.73
60.45 60.43 56.99 53.11
44.97 42.14 36.64 34.14 29.49 25.97 20.28 18.38 14.33
210 200 190 180 170 160 150 140 130 120 110 100 90 80 70 60 50 40 30 20 10
f1 (ppm)

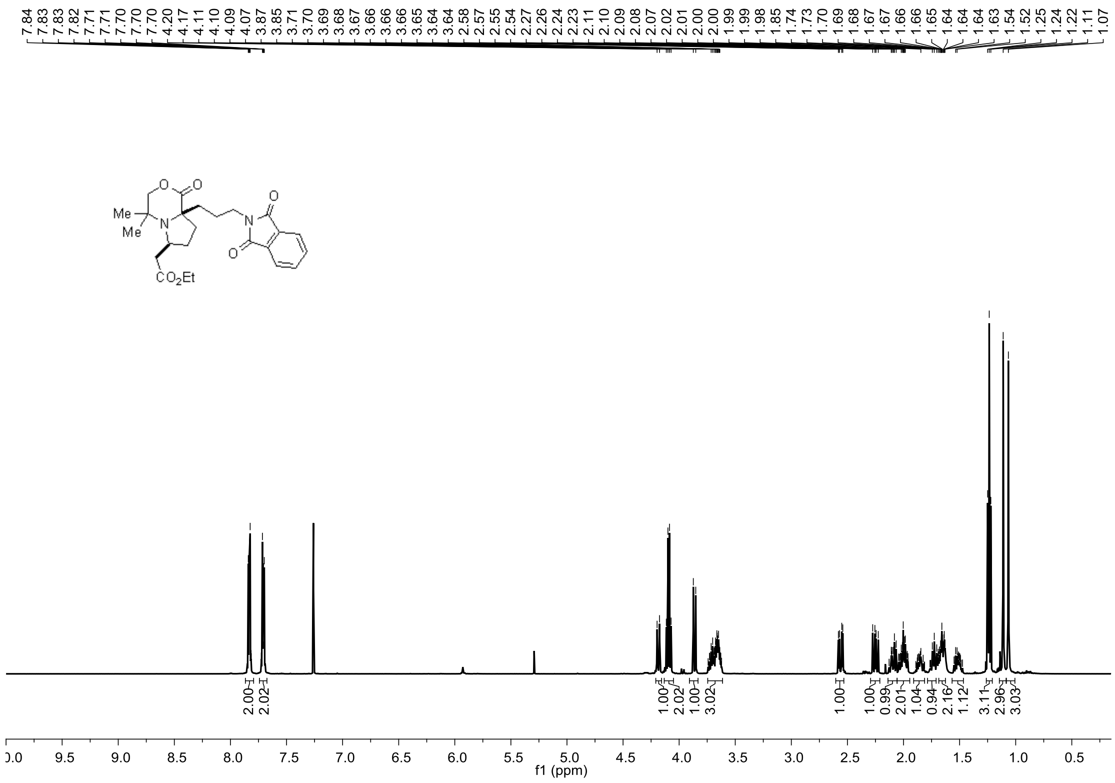
7.84
7.83
7.83
7.82
7.71
7.71
7.70
7.70
4.20
4.17
4.11
4.10
4.09
4.07
3.87
3.85
3.71
3.70
3.69
3.68
3.67
3.66
3.66
3.66
3.65
3.64
3.64
2.58
2.57
2.55
2.54
2.27
2.26
2.24
2.23
2.11
2.10
2.09
2.08
2.07
2.02
2.01
2.00
2.00
1.99
1.99
1.98
1.85
1.74
1.73
1.70
1.69
1.68
1.67
1.67
1.66
1.66
1.65
1.64
1.64
1.64
1.63
1.54
1.52
1.25
1.24
1.22
1.11
1.07
Me
Me
N
O
O
N
O
O
CO2Et
2.00
2.02
1.00
2.02
1.00
3.02
1.00
1.00
0.99
2.01
1.04
0.94
2.16
1.12
3.11
2.96
3.03
f1 (ppm)

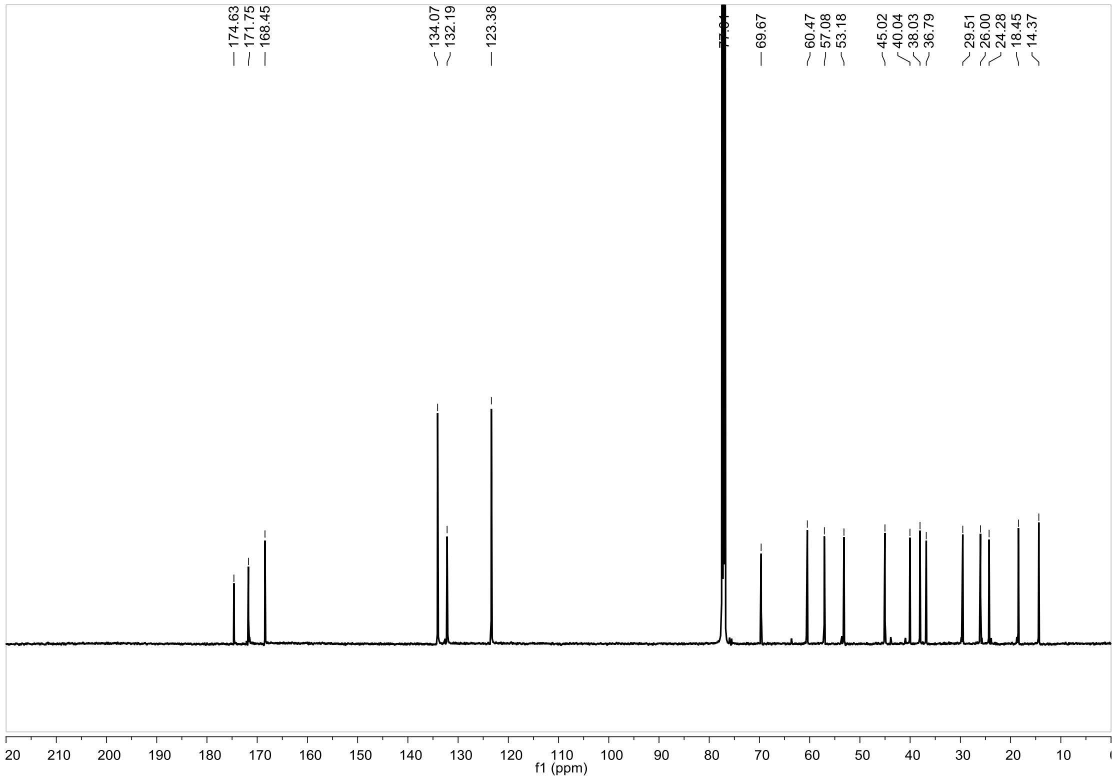
174.63
171.75
168.45
134.07
132.19
123.38
69.67
60.47
57.08
53.18
45.02
40.04
38.03
36.79
29.51
26.00
24.28
18.45
14.37
f1 (ppm)

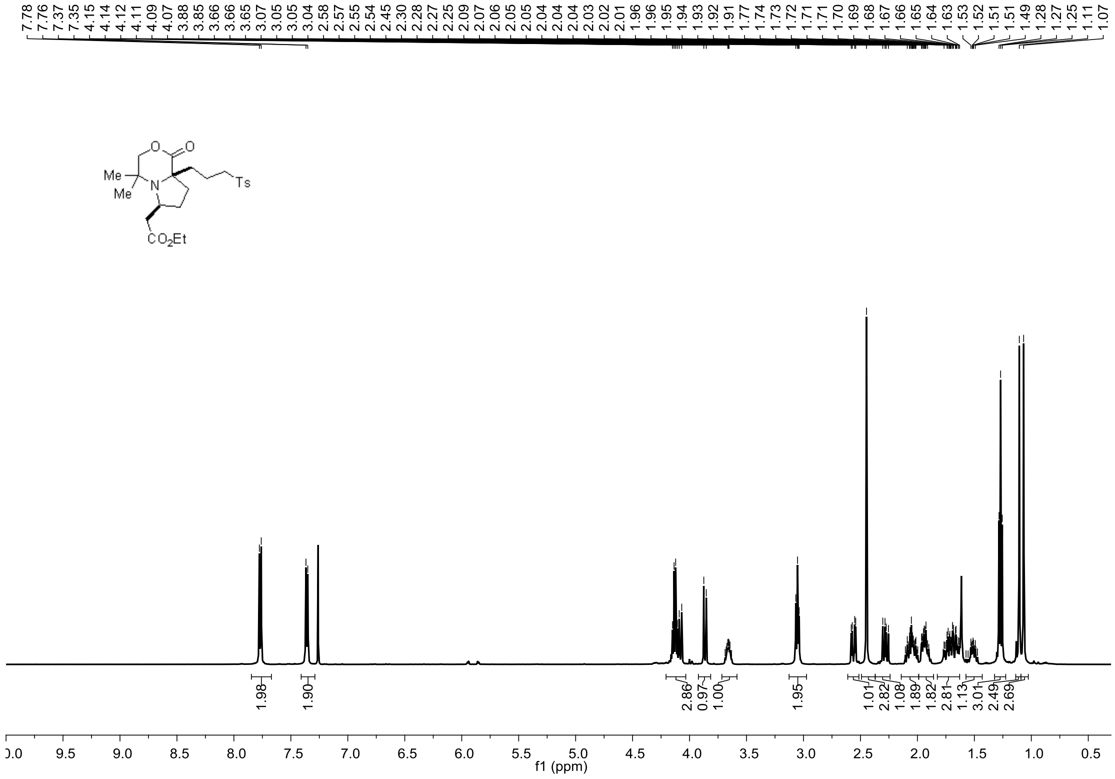
O
O
Me
Me
N
Ts
CO2Et
7.78
7.76
7.37
7.35
4.15
4.14
4.12
4.11
4.09
4.07
3.88
3.85
3.66
3.65
3.07
3.05
3.05
3.04
2.58
2.57
2.55
2.54
2.45
2.30
2.28
2.27
2.25
2.09
2.07
2.06
2.05
2.04
2.04
2.04
2.03
2.02
2.01
1.96
1.95
1.94
1.93
1.92
1.91
1.77
1.74
1.73
1.72
1.71
1.71
1.70
1.69
1.68
1.67
1.66
1.65
1.64
1.63
1.53
1.52
1.51
1.51
1.49
1.28
1.27
1.25
1.11
1.07
1.98
1.90
2.86
0.97
1.00
1.95
1.01
2.82
1.08
1.89
1.82
2.81
1.13
3.01
2.49
2.69
9.5
9.0
8.5
8.0
7.5
7.0
6.5
6.0
5.5
5.0
4.5
4.0
3.5
3.0
2.5
2.0
1.5
1.0
0.5
f1 (ppm)

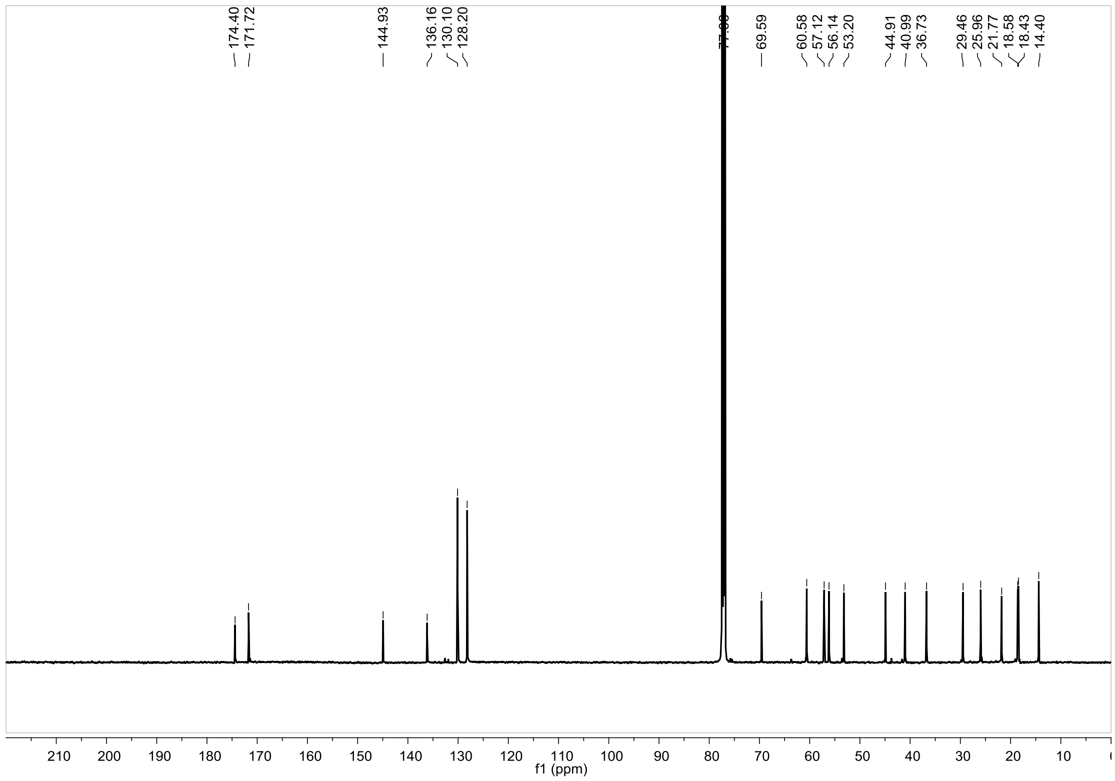
174.40
171.72
144.93
136.16
130.10
128.20
[illegible]
69.59
60.58
57.12
56.14
53.20
44.91
40.99
36.73
29.46
25.96
21.77
18.58
18.43
14.40
210
200
190
180
170
160
150
140
130
120
110
100
90
80
70
60
50
40
30
20
10
f1 (ppm)

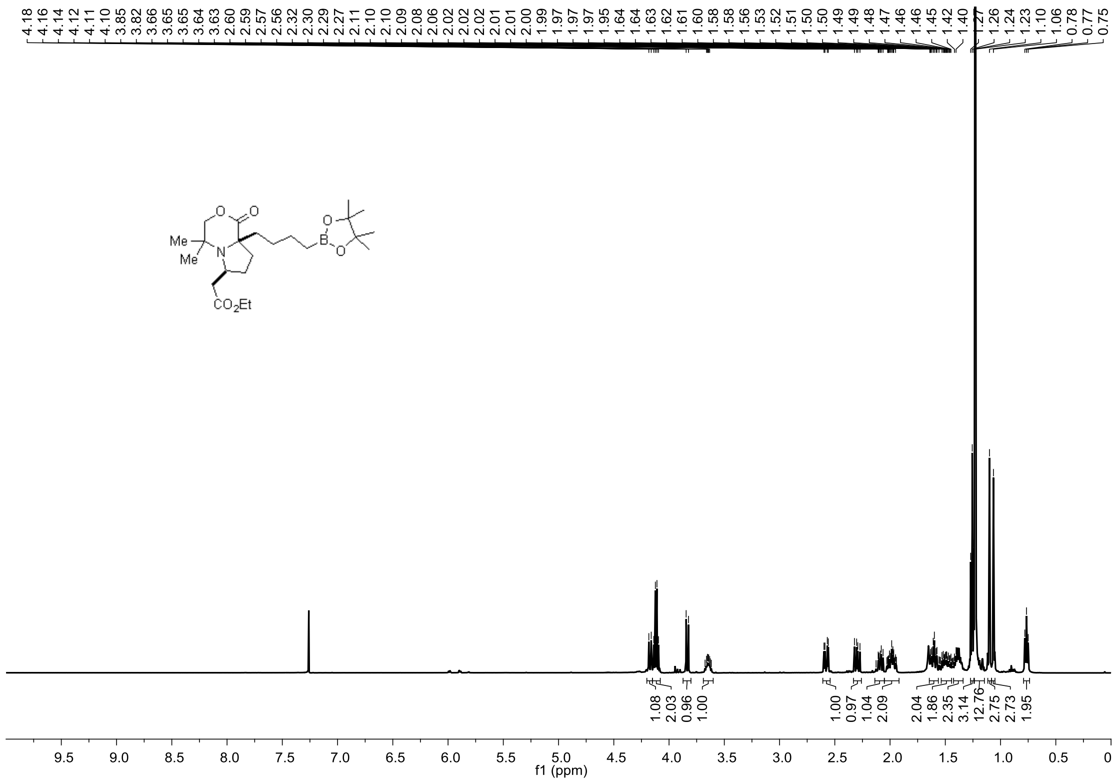
4.18
4.16
4.14
4.12
4.11
4.10
3.85
3.82
3.66
3.65
3.65
3.64
3.63
2.60
2.59
2.57
2.56
2.32
2.30
2.29
2.27
2.11
2.10
2.09
2.08
2.06
2.02
2.02
2.02
2.01
2.01
2.00
1.99
1.97
1.97
1.95
1.64
1.63
1.62
1.61
1.60
1.58
1.58
1.56
1.53
1.52
1.51
1.50
1.50
1.49
1.49
1.48
1.47
1.46
1.46
1.45
1.42
1.40
1.27
1.26
1.24
1.23
1.10
1.06
0.78
0.77
0.75
Me
Me
CO2Et
1.08
2.03
0.96
1.00
1.00
0.97
1.04
2.09
2.04
1.86
2.35
3.14
12.76
2.75
2.73
1.95
9.5 9.0 8.5 8.0 7.5 7.0 6.5 6.0 5.5 5.0 4.5 4.0 3.5 3.0 2.5 2.0 1.5 1.0 0.5 0
f1 (ppm)

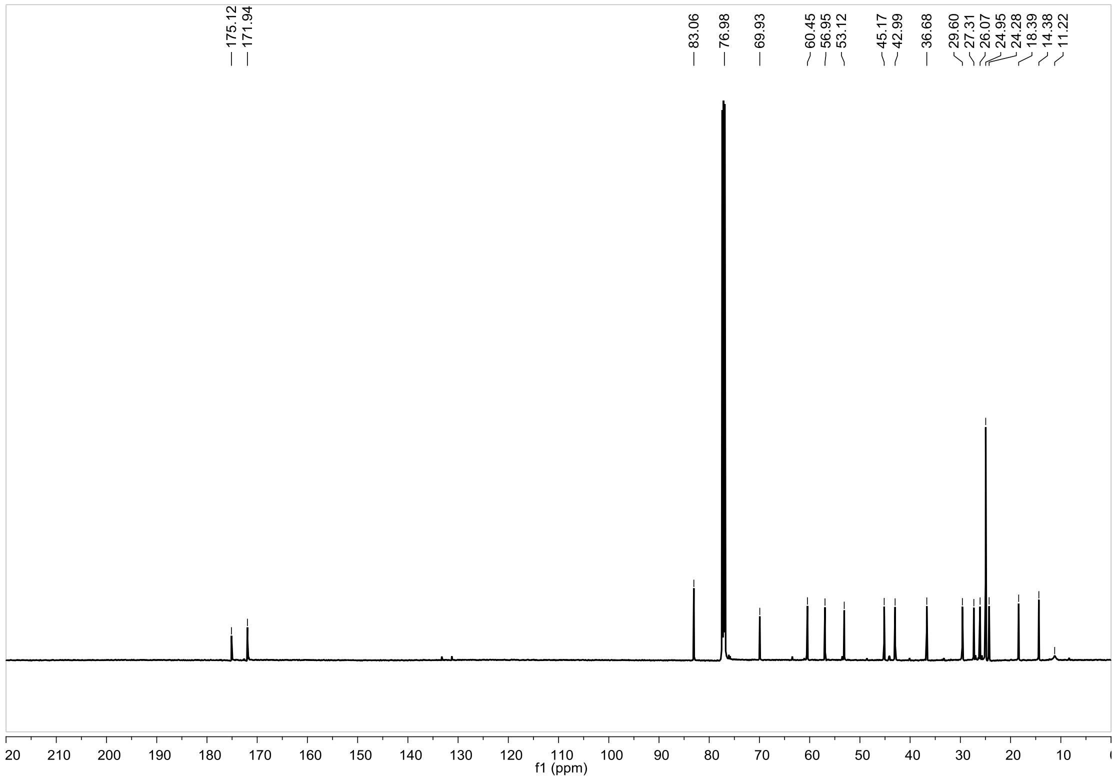
175.12
171.94
83.06
76.98
69.93
60.45
56.95
53.12
45.17
42.99
36.68
29.60
27.31
26.07
24.95
24.28
18.39
14.38
11.22
20 210 200 190 180 170 160 150 140 130 120 110 100 90 80 70 60 50 40 30 20 10
f1 (ppm)

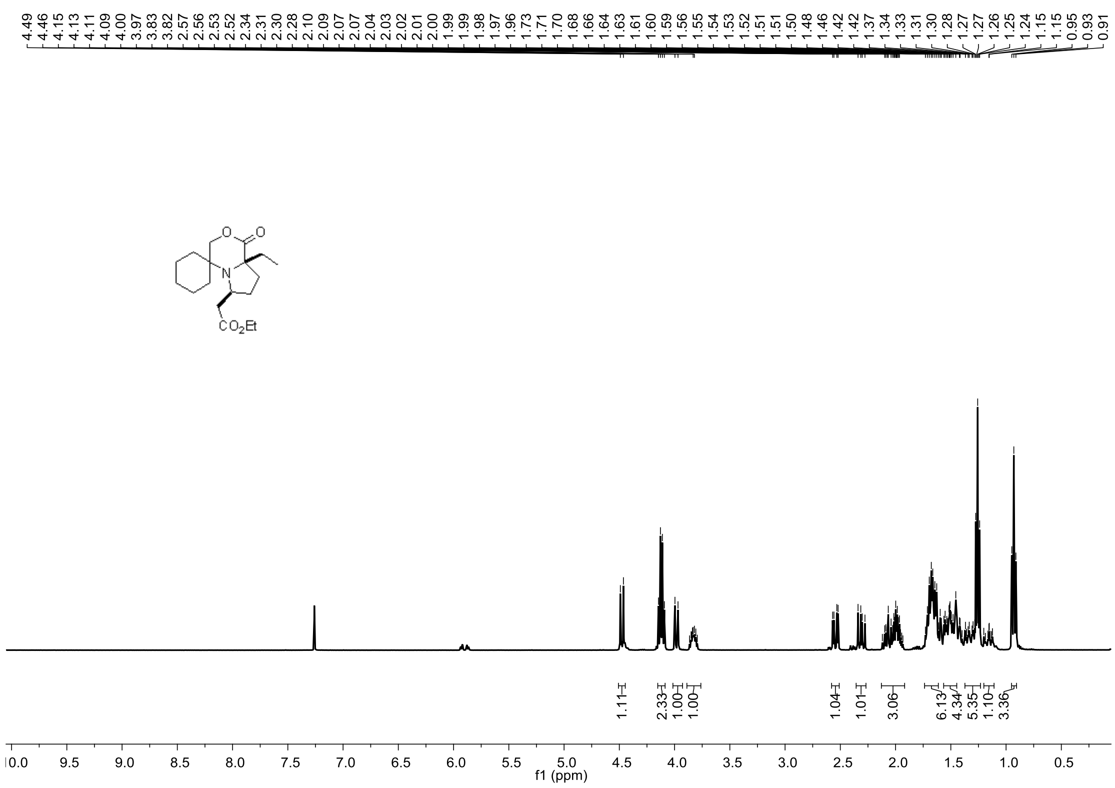
4.49 4.46 4.15 4.13 4.11 4.09 4.00 3.97 3.83 3.82 2.57 2.56 2.53 2.52 2.34 2.31 2.30 2.28 2.10 2.09 2.07 2.07 2.04 2.03 2.02 2.01 2.00 1.99 1.98 1.97 1.96 1.73 1.71 1.70 1.68 1.66 1.64 1.63 1.61 1.60 1.59 1.56 1.55 1.54 1.53 1.52 1.51 1.51 1.50 1.48 1.46 1.42 1.42 1.37 1.34 1.33 1.31 1.30 1.28 1.27 1.27 1.26 1.25 1.24 1.15 1.15 0.95 0.93 0.91
O
O
N
CO_2Et
1.11 2.33 1.00 1.00 1.04 1.01 3.06 6.13 4.34 5.35 1.10 3.36
f1 (ppm)

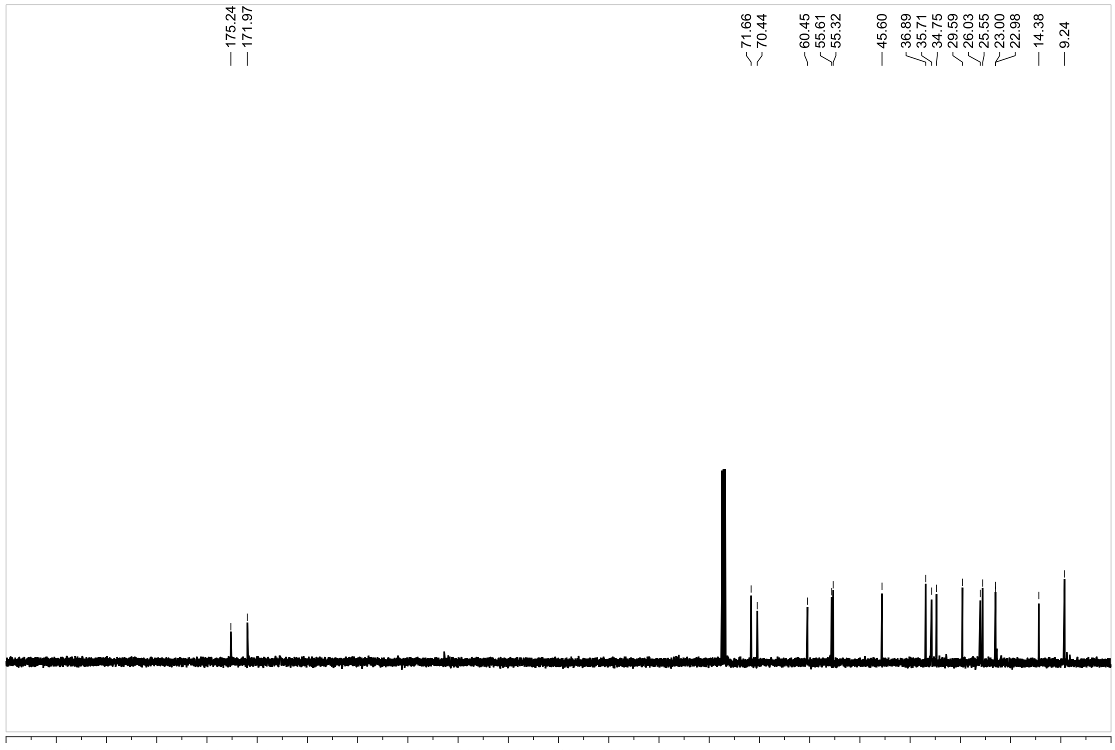
175.24 171.97
71.66 70.44
60.45 55.61 55.32
45.60
36.89 35.71 34.75 29.59 26.03 25.55 23.00 22.98
14.38
9.24
f1 (ppm)

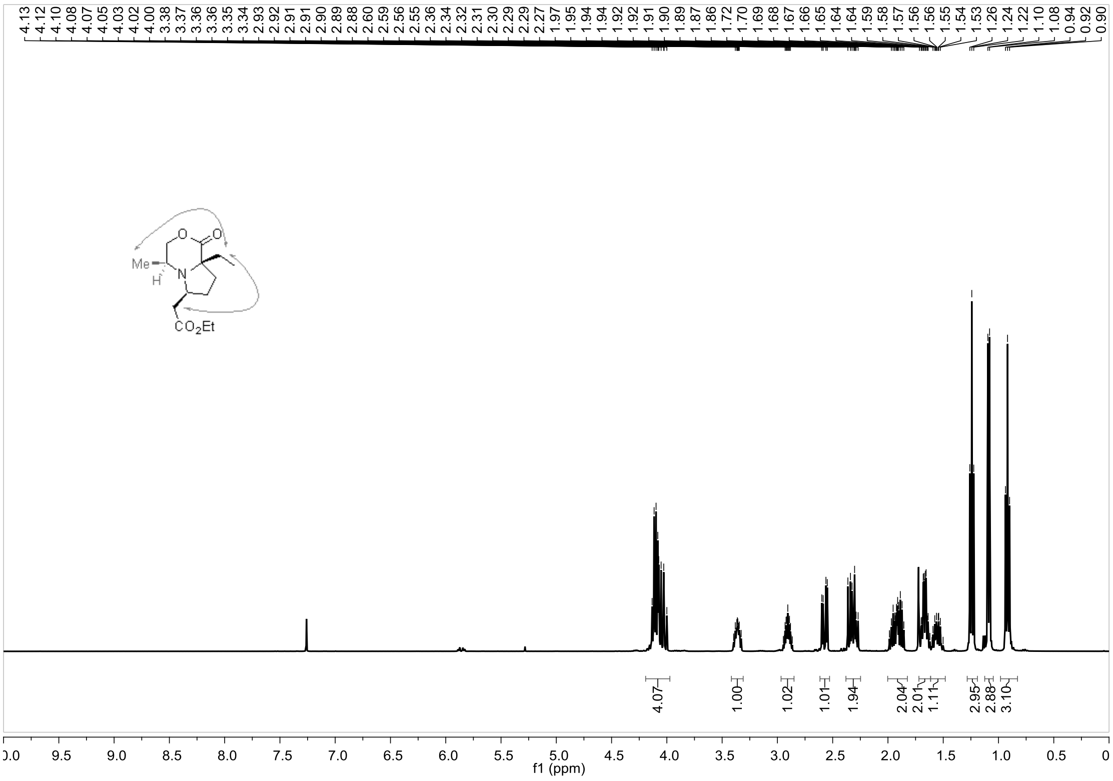
4.13 4.12 4.10 4.08 4.07 4.05 4.03 4.02 4.00 3.38 3.37 3.36 3.36 3.35 3.34 2.93 2.92 2.91 2.91 2.90 2.89 2.88 2.60 2.59 2.56 2.55 2.36 2.34 2.32 2.31 2.30 2.29 2.29 2.27 1.97 1.95 1.94 1.94 1.92 1.92 1.91 1.90 1.89 1.87 1.86 1.72 1.70 1.69 1.68 1.67 1.66 1.65 1.64 1.64 1.59 1.58 1.57 1.56 1.56 1.55 1.54 1.53 1.26 1.24 1.22 1.10 1.08 0.94 0.92 0.90
Me
H
N
O
O
CO2Et
4.07
1.00
1.02
1.01
1.94
2.04
2.01
1.11
2.95
2.88
3.10
f1 (ppm)

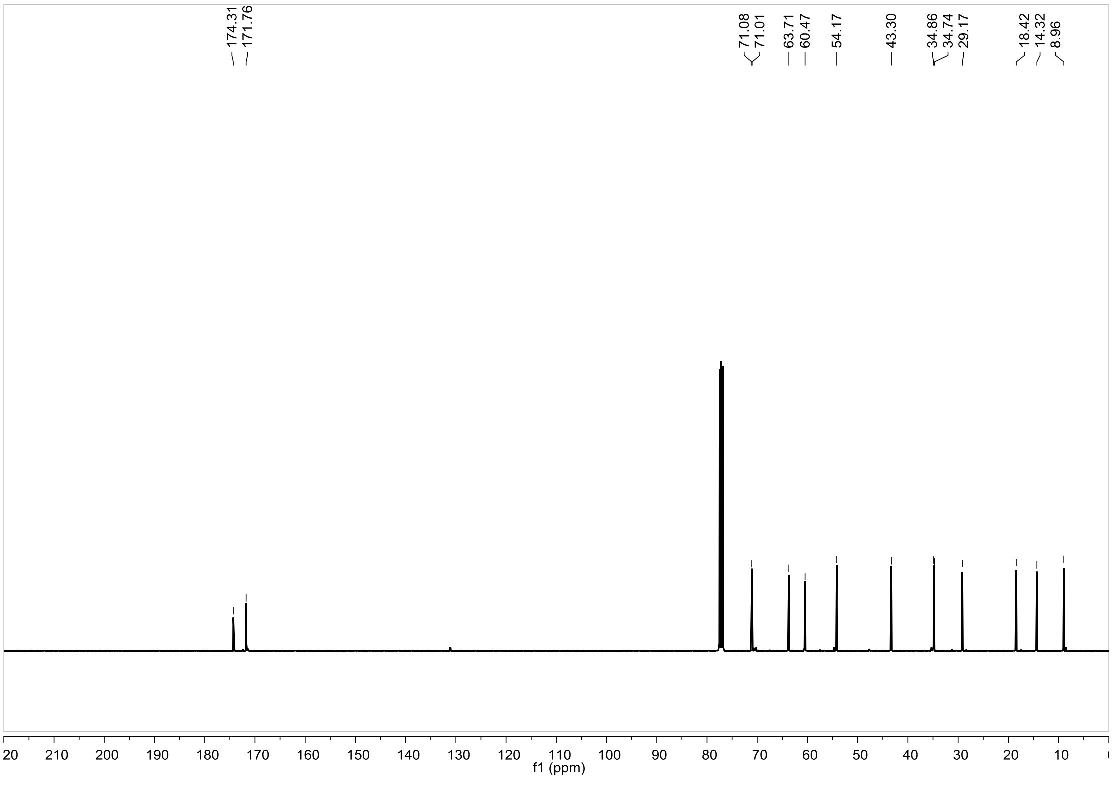
174.31
171.76
71.08
71.01
63.71
60.47
54.17
43.30
34.86
34.74
29.17
18.42
14.32
8.96
f1 (ppm)

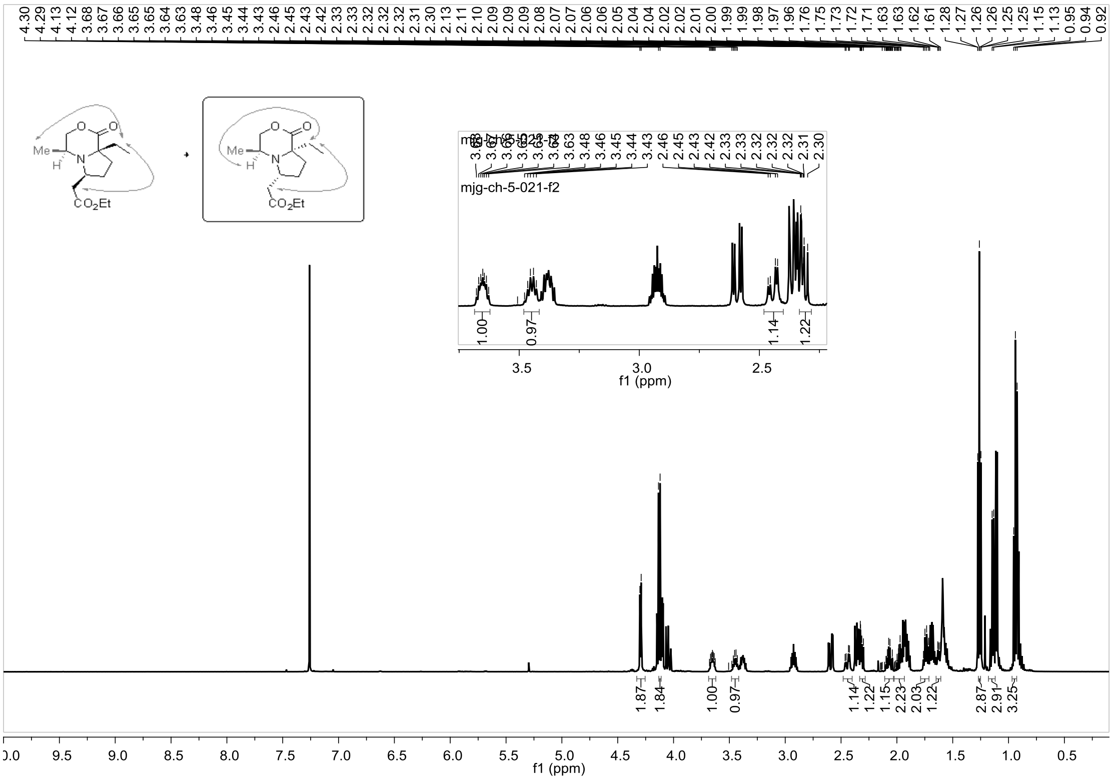

4.30 4.29 4.13 4.12 3.68 3.67 3.66 3.65 3.65 3.64 3.63 3.48 3.46 3.45 3.44 3.43 2.46 2.45 2.43 2.42 2.33 2.33 2.32 2.32 2.32 2.31 2.30 2.13 2.11 2.10 2.09 2.09 2.09 2.08 2.07 2.07 2.06 2.06 2.05 2.04 2.04 2.02 2.02 2.01 2.00 1.99 1.99 1.98 1.97 1.96 1.76 1.75 1.73 1.72 1.71 1.63 1.63 1.62 1.61 1.28 1.27 1.26 1.26 1.25 1.25 1.15 1.13 0.95 0.94 0.92
Me
H
N
O
CO_2Et
mjg-ch-5-021-f2
1.00 0.97 1.14 1.22
f1 (ppm)
1.87 1.84 1.00 0.97 1.14 1.22 1.15 2.23 2.03 1.22 2.87 2.91 3.25
f1 (ppm)

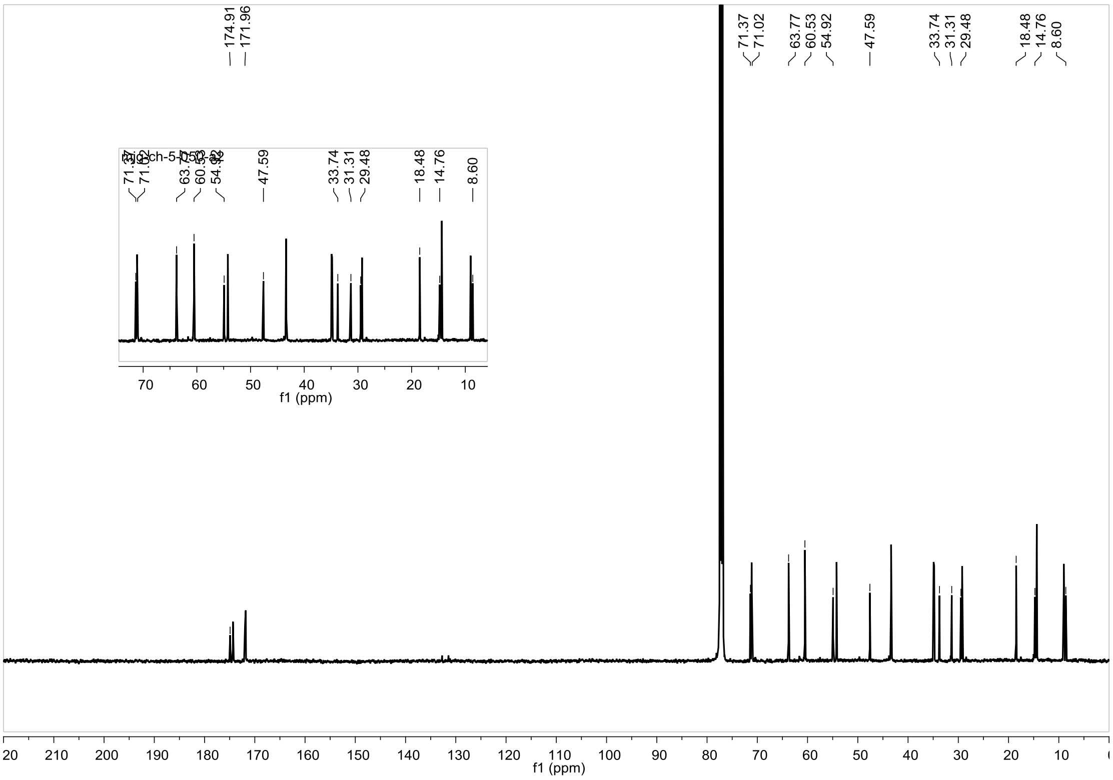

174.91 171.96 71.37 71.02 63.77 60.53 54.92 47.59 33.74 31.31 29.48 18.48 14.76 8.60
47.59 33.74 31.31 29.48 18.48 14.76 8.60
f1 (ppm)
f1 (ppm)

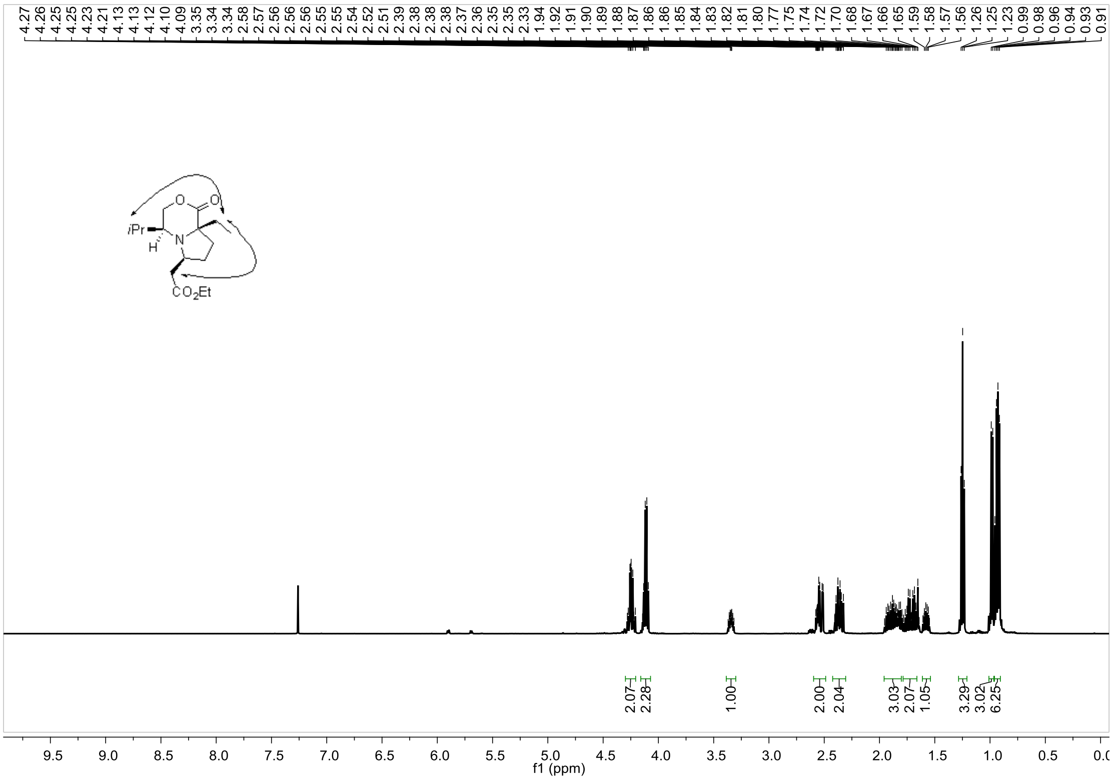
4.27 4.26 4.25 4.25 4.23 4.21 4.13 4.12 4.10 4.09 3.35 3.34 3.34 2.58 2.57 2.56 2.56 2.56 2.55 2.55 2.54 2.52 2.51 2.39 2.38 2.38 2.38 2.37 2.36 2.35 2.35 2.33 1.94 1.92 1.91 1.90 1.89 1.88 1.87 1.86 1.86 1.85 1.84 1.83 1.82 1.81 1.80 1.77 1.75 1.74 1.72 1.70 1.68 1.67 1.66 1.65 1.59 1.58 1.57 1.56 1.26 1.25 1.23 0.99 0.98 0.96 0.94 0.93 0.91
iPr
H
N
O
O
CO2Et
2.07 2.28 1.00 2.00 2.04 3.03 2.07 1.05 3.29 3.02 6.25
9.5 9.0 8.5 8.0 7.5 7.0 6.5 6.0 5.5 5.0 4.5 4.0 3.5 3.0 2.5 2.0 1.5 1.0 0.5 0.0
f1 (ppm)

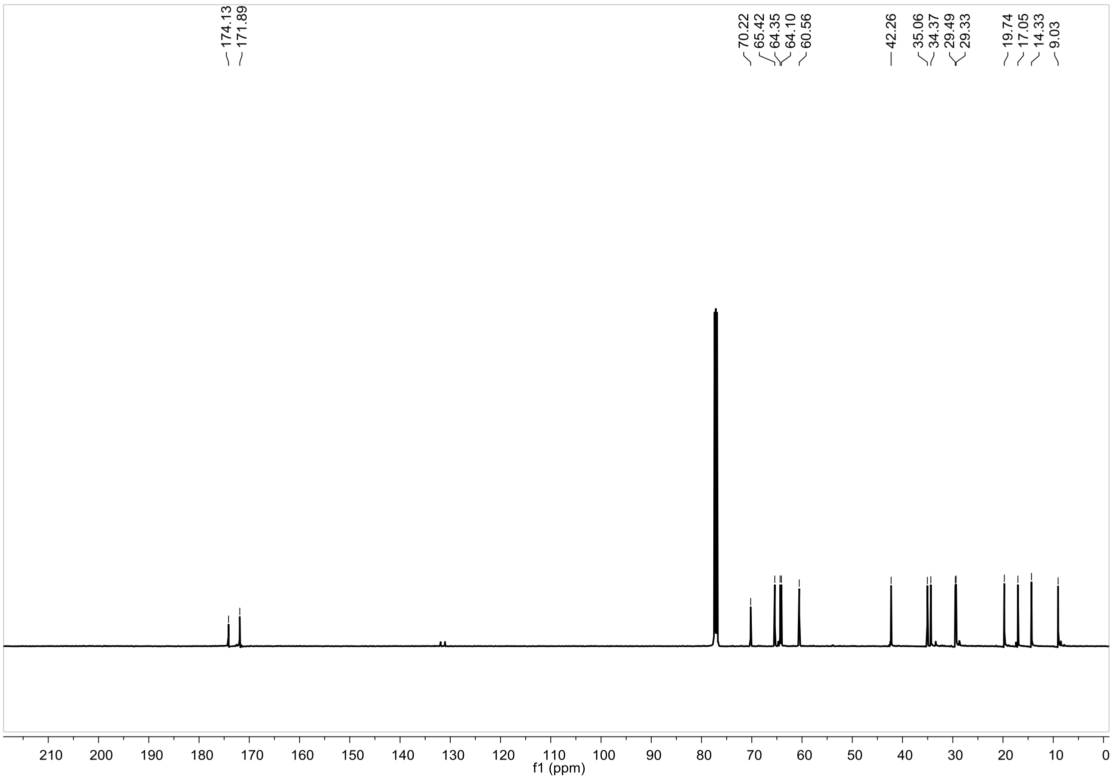
174.13 171.89
70.22 65.42 64.35 64.10 60.56
42.26 35.06 34.37 29.49 29.33
19.74 17.05 14.33 9.03
210 200 190 180 170 160 150 140 130 120 110 100 90 80 70 60 50 40 30 20 10 0
f1 (ppm)

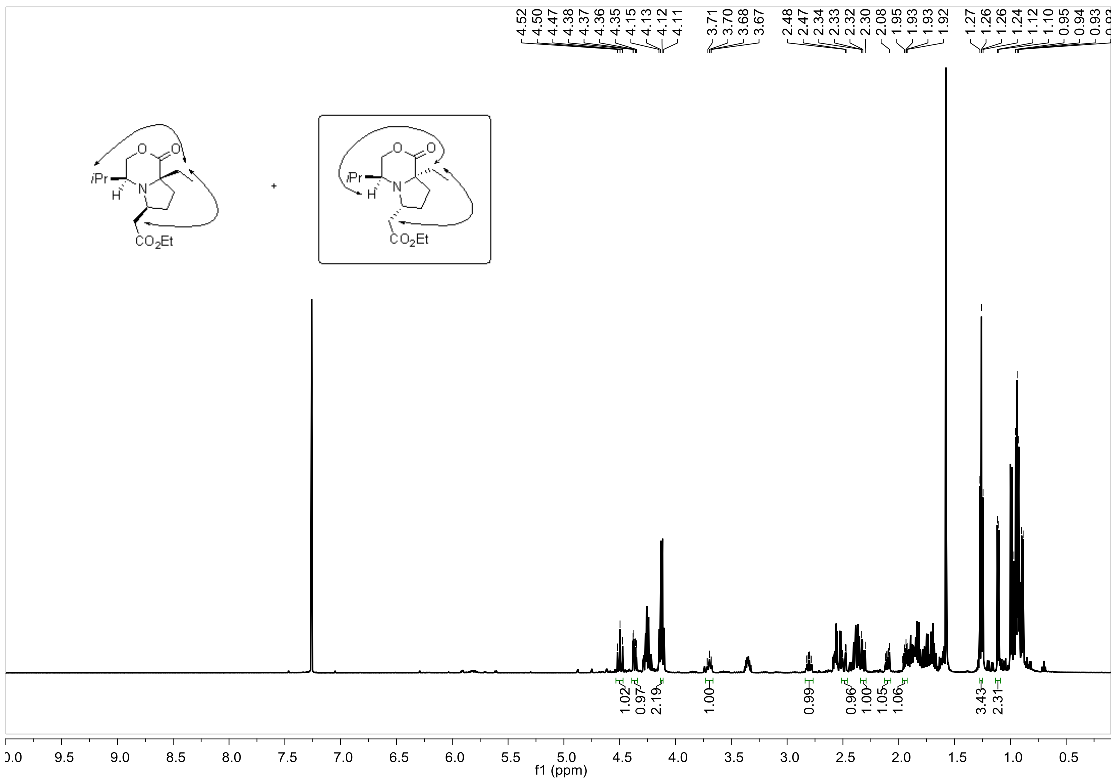

iPr
H
N
O
CO2Et
f1 (ppm)

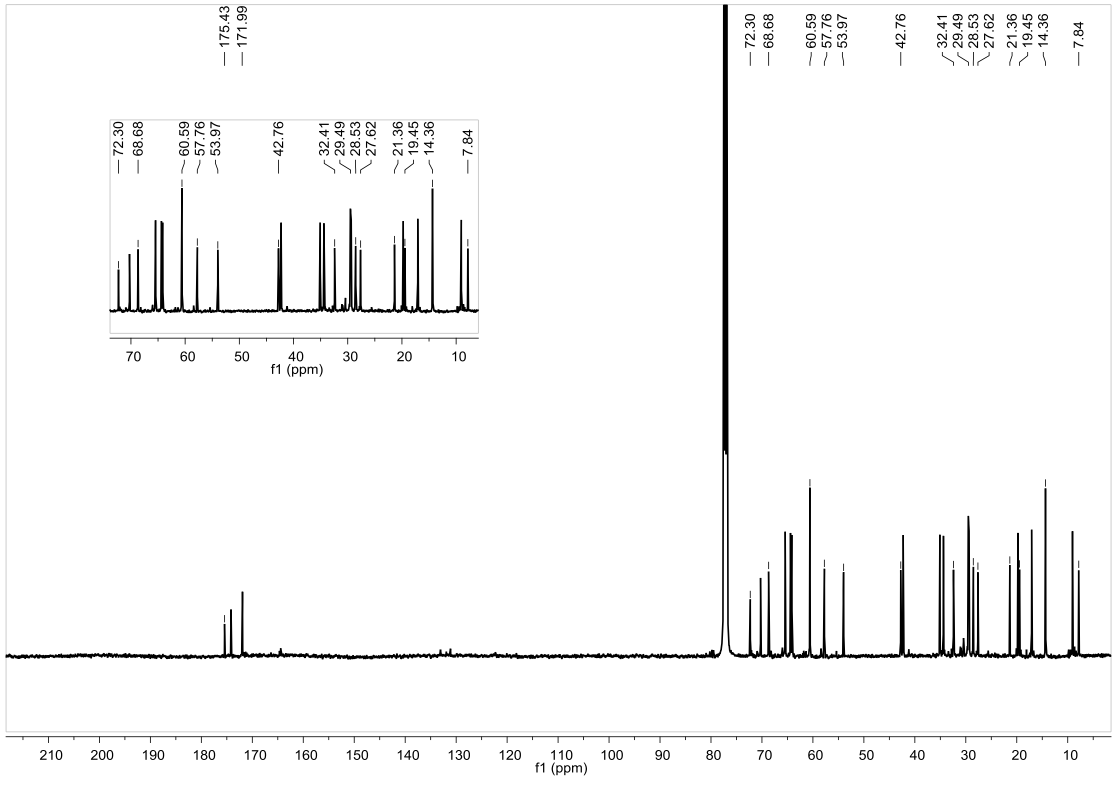

175.43
171.99
72.30
68.68
60.59
57.76
53.97
42.76
32.41
29.49
28.53
27.62
21.36
19.45
14.36
7.84
f1 (ppm)

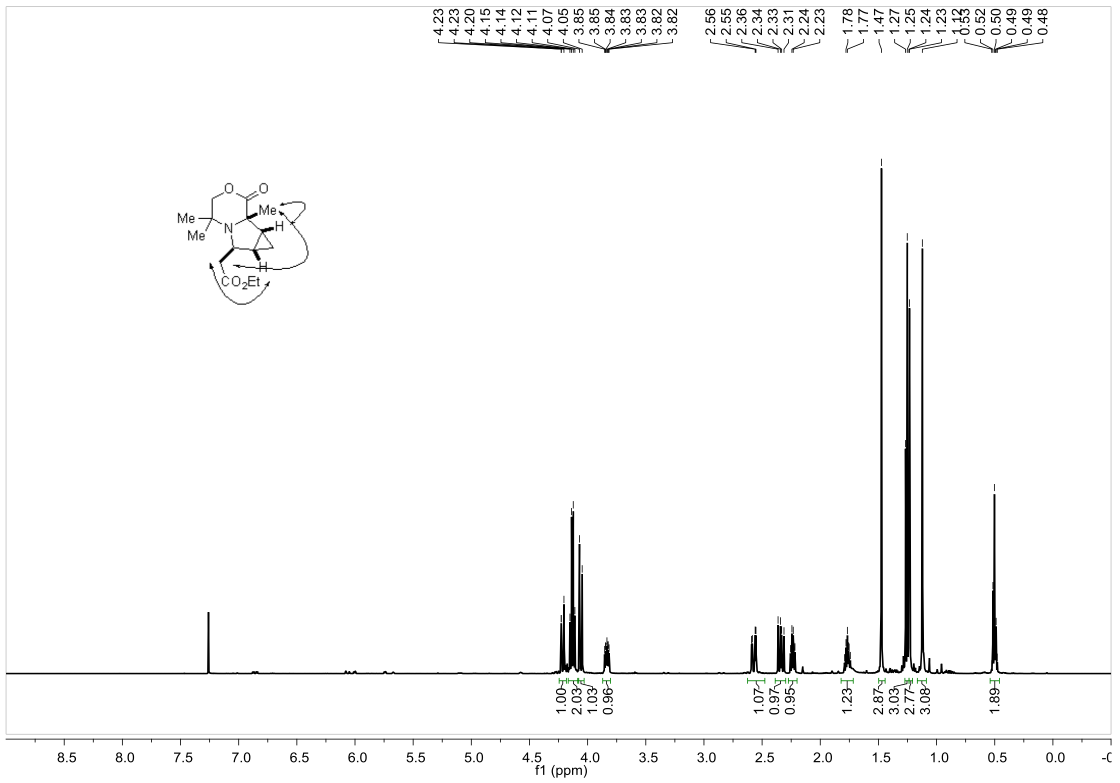

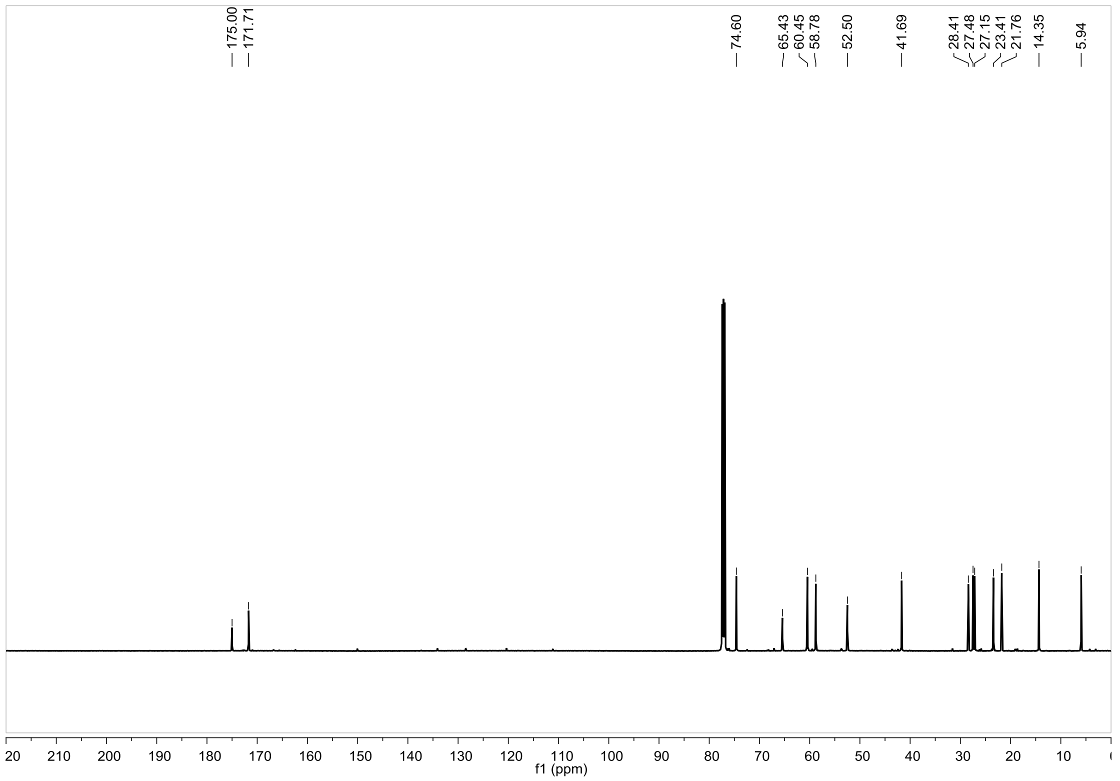

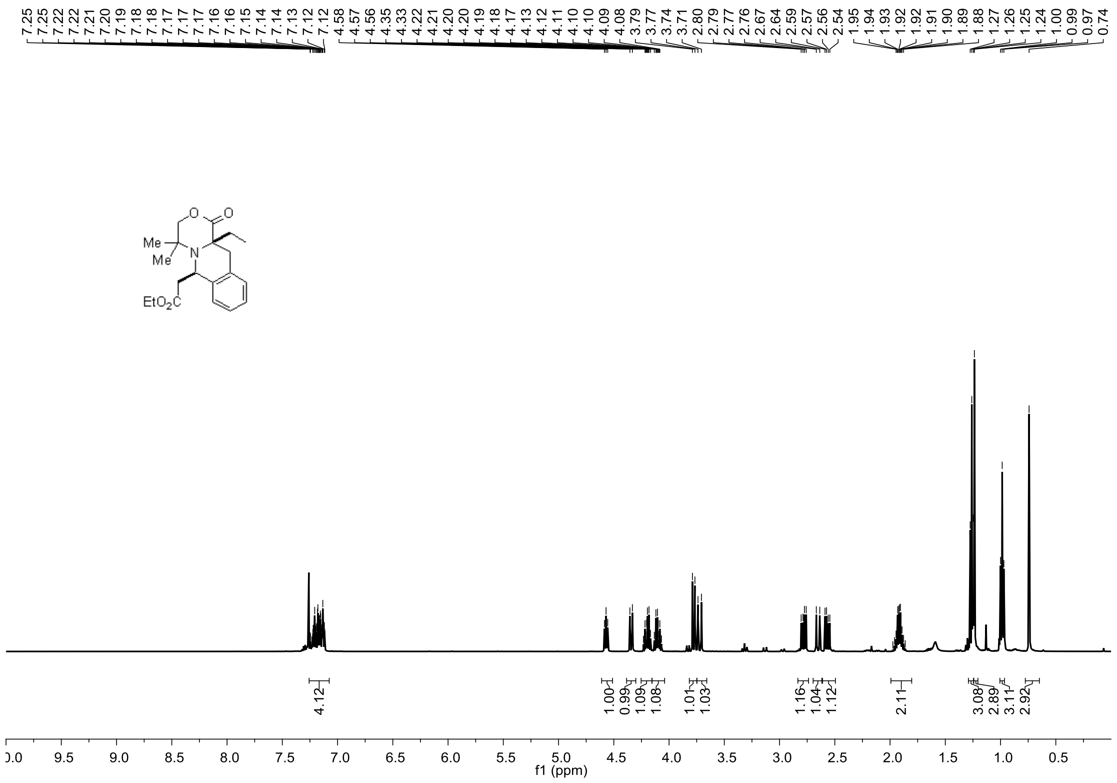
7.25 7.25 7.22 7.22 7.21 7.20 7.19 7.18 7.18 7.17 7.17 7.16 7.15 7.14 7.13 7.12 7.12 4.58 4.57 4.56 4.35 4.33 4.22 4.21 4.20 4.20 4.19 4.18 4.17 4.13 4.12 4.11 4.10 4.09 4.08 3.79 3.77 3.74 3.71 2.80 2.79 2.77 2.76 2.67 2.64 2.59 2.57 2.56 2.54 1.95 1.94 1.93 1.92 1.92 1.91 1.90 1.89 1.88 1.27 1.26 1.25 1.24 1.00 0.99 0.97 0.74
Me
Me
N
O
O
EtO2C
4.12
1.00 0.99 1.09 1.08 1.01 1.03 1.16 1.04 1.12 2.11 3.08 2.89 3.11 2.92
f1 (ppm)

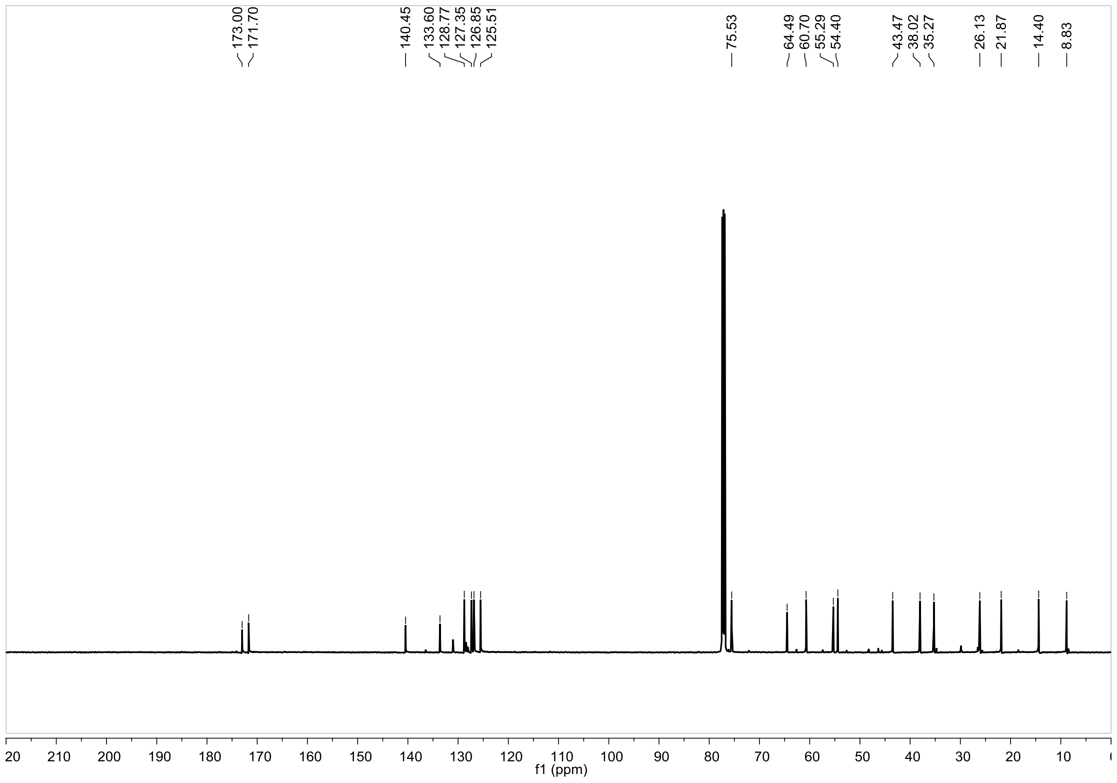
173.00 171.70 140.45 133.60 128.77 127.35 126.85 125.51 75.53 64.49 60.70 55.29 54.40 43.47 38.02 35.27 26.13 21.87 14.40 8.83
f1 (ppm)

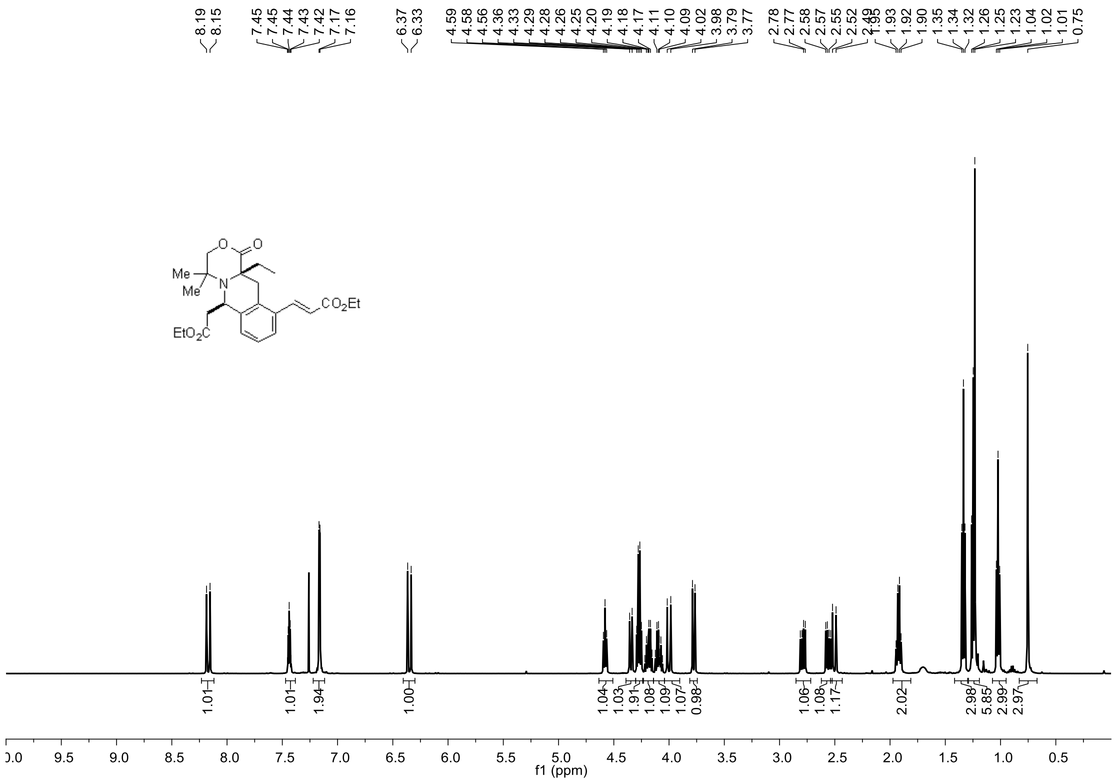
8.19
8.15
7.45
7.45
7.44
7.43
7.42
7.17
7.16
6.37
6.33
4.59
4.58
4.56
4.36
4.33
4.29
4.28
4.26
4.25
4.20
4.19
4.18
4.17
4.11
4.10
4.09
4.02
3.98
3.79
3.77
2.78
2.77
2.58
2.57
2.55
2.52
1.93
1.92
1.90
1.35
1.34
1.32
1.26
1.25
1.23
1.04
1.02
1.01
0.75
O
O
Me
Me
N
CO_2Et
EtO_2C
1.01
1.01
1.94
1.00
1.04
1.03
1.91
1.08
1.09
1.07
0.98
1.06
1.08
1.17
2.02
2.98
5.85
2.99
2.97
9.5 9.0 8.5 8.0 7.5 7.0 6.5 6.0 5.5 5.0 4.5 4.0 3.5 3.0 2.5 2.0 1.5 1.0 0.5
f1 (ppm)

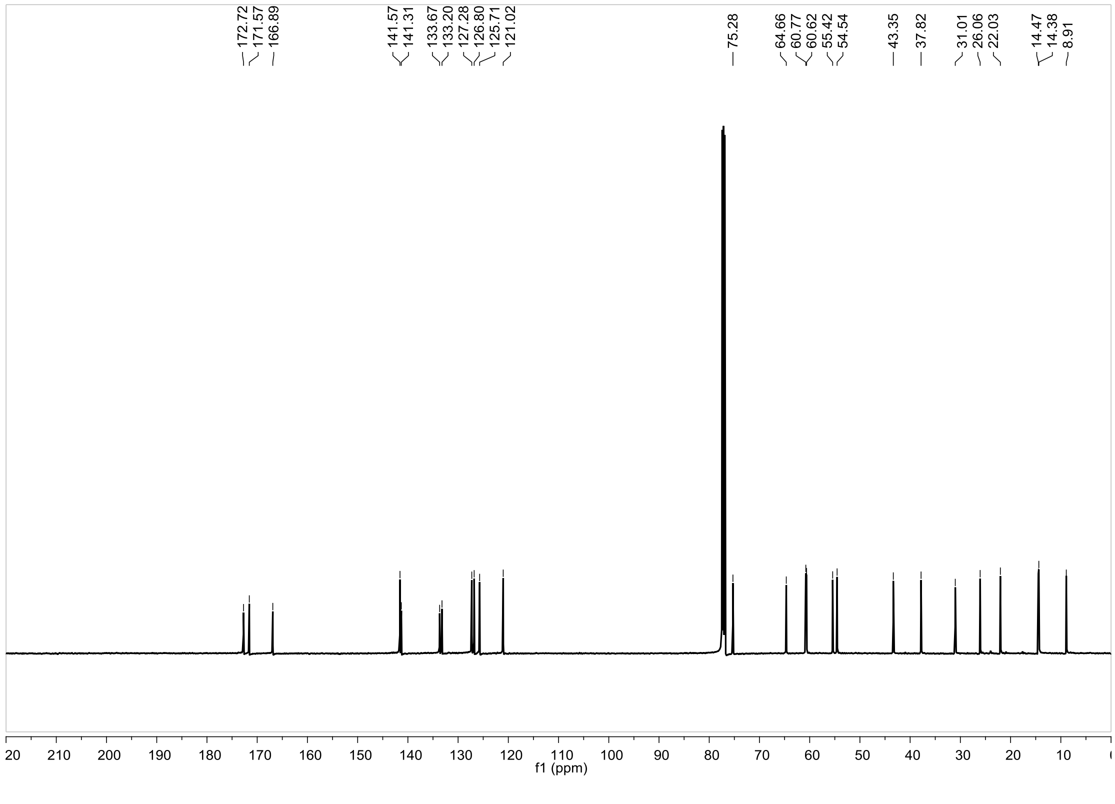
172.72
171.57
166.89
141.57
141.31
133.67
133.20
127.28
126.80
125.71
121.02
75.28
64.66
60.77
60.62
55.42
54.54
43.35
37.82
31.01
26.06
22.03
14.47
14.38
8.91
210 200 190 180 170 160 150 140 130 120 110 100 90 80 70 60 50 40 30 20 10
f1 (ppm)

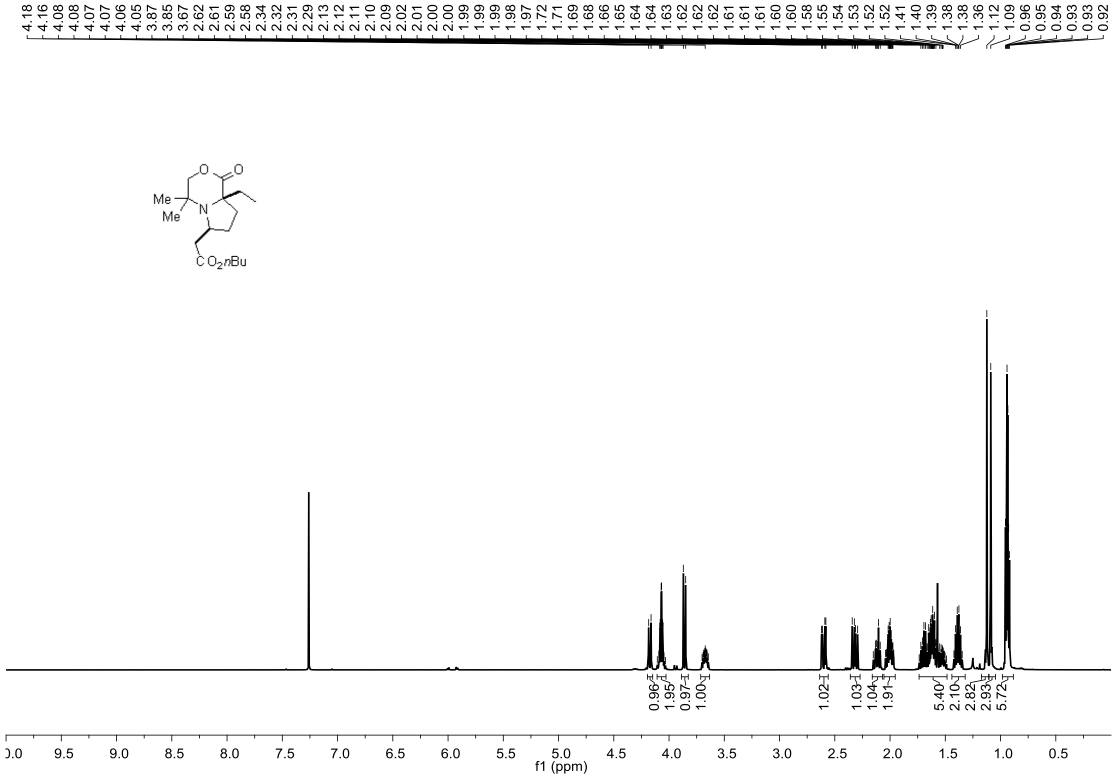
4.18
4.16
4.08
4.08
4.07
4.06
4.05
3.87
3.85
3.67
2.62
2.61
2.59
2.58
2.34
2.32
2.31
2.29
2.13
2.12
2.11
2.10
2.09
2.02
2.01
2.00
2.00
1.99
1.99
1.99
1.98
1.97
1.72
1.71
1.69
1.68
1.66
1.65
1.64
1.64
1.63
1.62
1.62
1.62
1.61
1.61
1.61
1.60
1.60
1.58
1.55
1.54
1.53
1.52
1.52
1.41
1.40
1.39
1.38
1.38
1.36
1.12
1.09
0.96
0.95
0.94
0.93
0.93
0.92
O
O
Me
Me
N
CO2nBu
0.96
1.95
0.97
1.00
1.02
1.03
1.04
1.91
5.40
2.10
2.82
2.93
5.72
f1 (ppm)

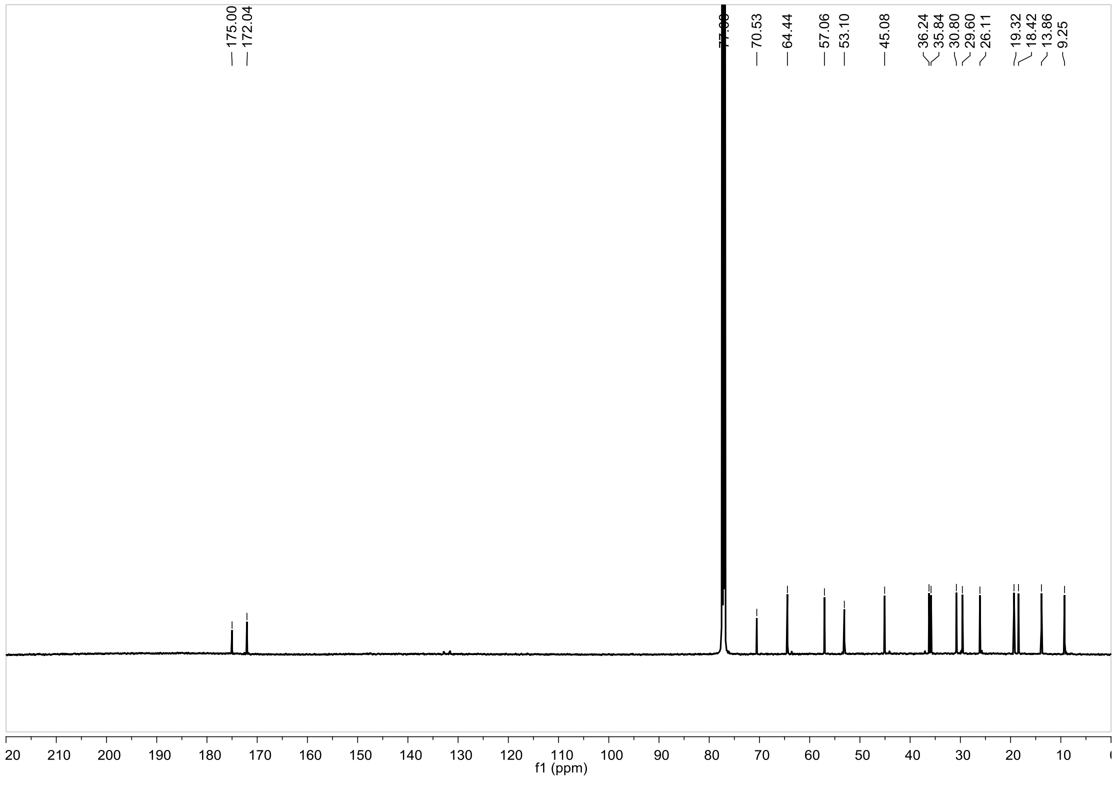
175.00
172.04
70.53
64.44
57.06
53.10
45.08
36.24
35.84
30.80
29.60
26.11
19.32
18.42
13.86
9.25
f1 (ppm)

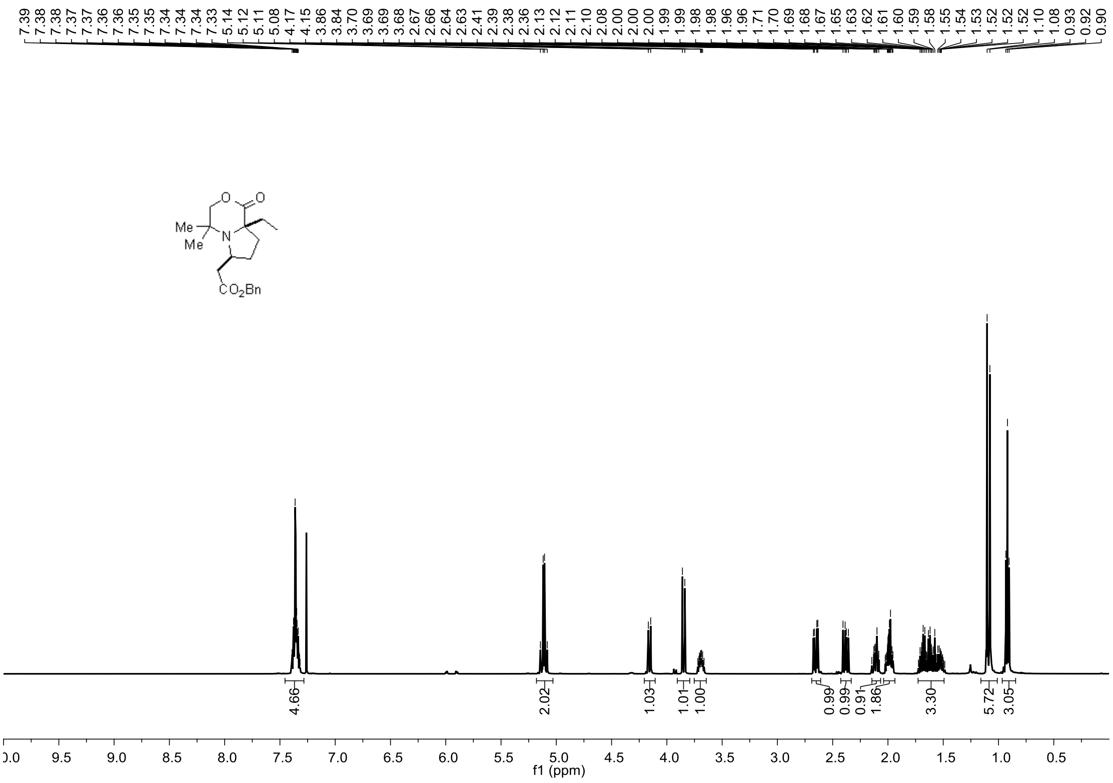
7.39 7.38 7.38 7.37 7.37 7.36 7.36 7.35 7.35 7.34 7.34 7.34 7.33 5.14 5.12 5.11 5.08 4.17 4.15 3.86 3.84 3.70 3.69 3.69 3.68 2.67 2.66 2.64 2.63 2.41 2.39 2.38 2.36 2.13 2.12 2.11 2.10 2.08 2.00 2.00 2.00 1.99 1.99 1.98 1.98 1.96 1.96 1.71 1.70 1.69 1.68 1.67 1.65 1.63 1.62 1.61 1.60 1.59 1.58 1.55 1.54 1.53 1.52 1.52 1.52 1.10 1.08 0.93 0.92 0.90
O
O
Me
Me
N
CO2Bn
4.66
2.02
1.03
1.01
1.00
0.99
0.99
0.91
1.86
3.30
5.72
3.05
9.5 9.0 8.5 8.0 7.5 7.0 6.5 6.0 5.5 5.0 4.5 4.0 3.5 3.0 2.5 2.0 1.5 1.0 0.5
f1 (ppm)

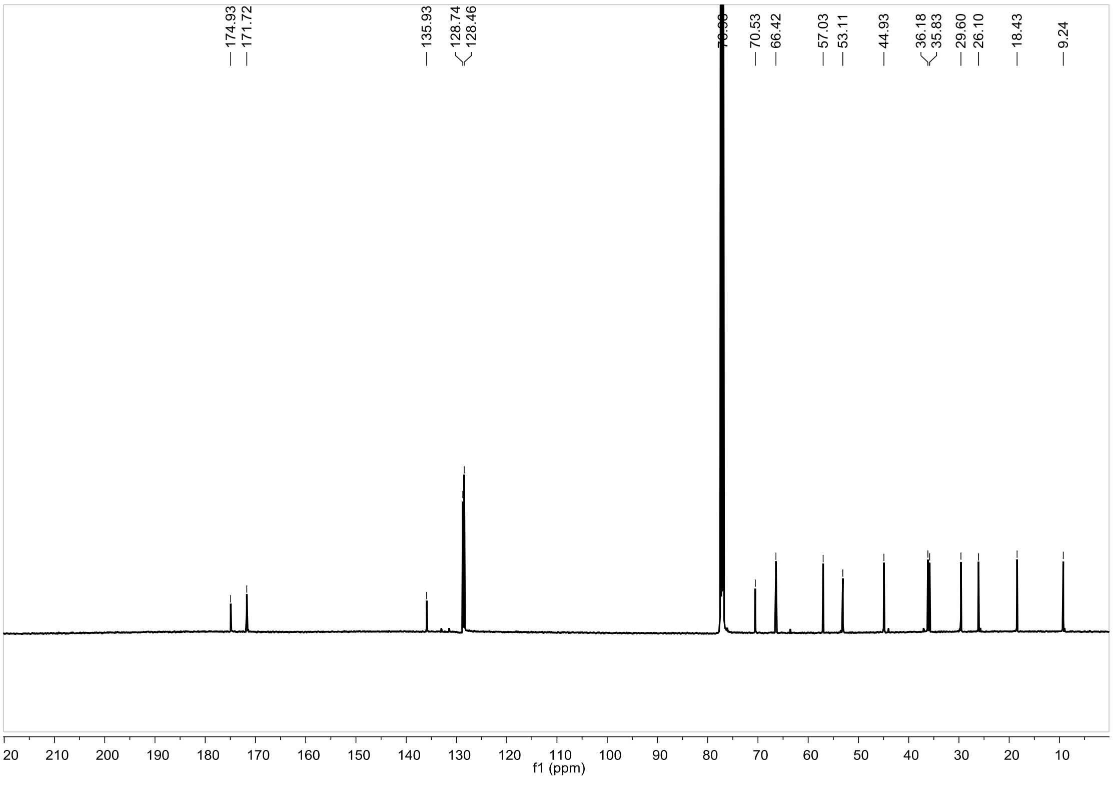
174.93
171.72
135.93
128.74
128.46
70.53
66.42
57.03
53.11
44.93
36.18
35.83
29.60
26.10
18.43
9.24
210 200 190 180 170 160 150 140 130 120 110 100 90 80 70 60 50 40 30 20 10
f1 (ppm)

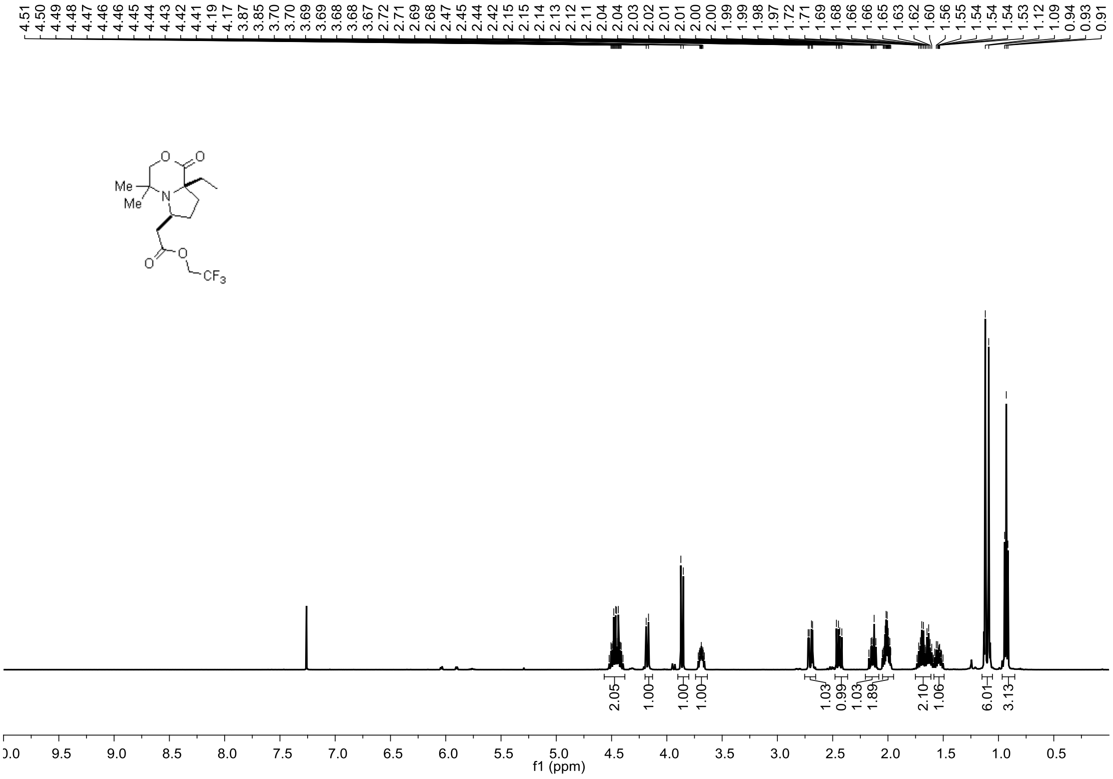
4.51 4.50 4.49 4.48 4.47 4.46 4.45 4.44 4.43 4.42 4.41 4.19 4.17 3.87 3.85 3.70 3.70 3.69 3.69 3.68 3.68 3.67 2.72 2.71 2.69 2.68 2.47 2.45 2.44 2.42 2.15 2.15 2.14 2.13 2.12 2.11 2.04 2.04 2.03 2.02 2.01 2.01 2.00 2.00 1.99 1.99 1.98 1.97 1.72 1.71 1.69 1.68 1.66 1.66 1.65 1.63 1.62 1.60 1.56 1.55 1.54 1.54 1.54 1.53 1.12 1.09 0.94 0.93 0.91
Me
Me
N
O
O
O
O
CF3
2.05
1.00
1.00
1.00
1.03
0.99
1.03
1.89
2.10
1.06
6.01
3.13
f1 (ppm)

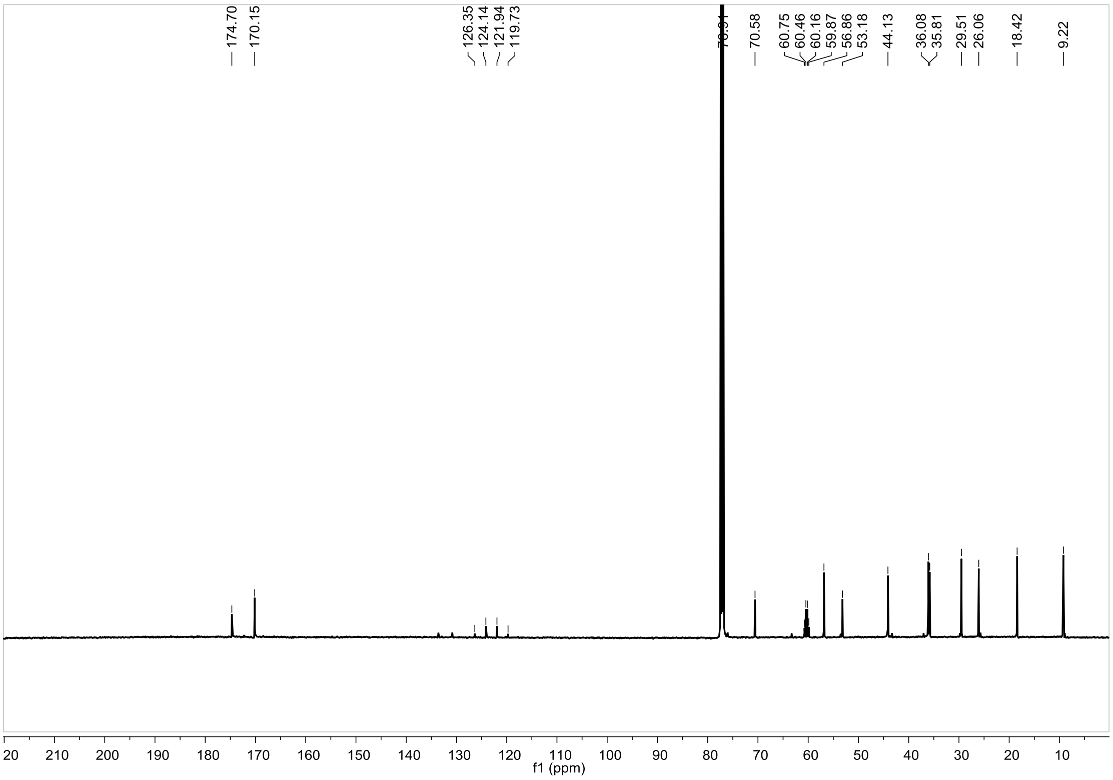
174.70
170.15
126.35
124.14
121.94
119.73
70.58
60.75
60.46
60.16
59.87
56.86
53.18
44.13
36.08
35.81
29.51
26.06
18.42
9.22
f1 (ppm)

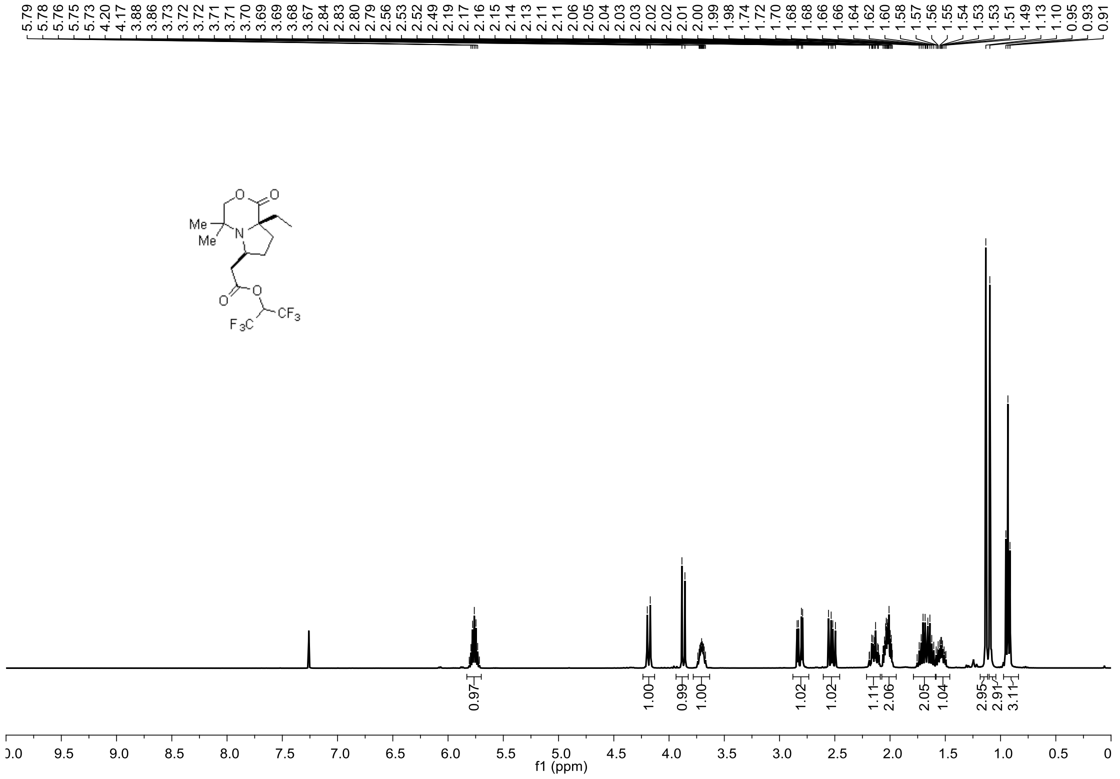
5.79
5.78
5.76
5.75
5.73
4.20
4.17
3.88
3.86
3.73
3.72
3.72
3.71
3.71
3.70
3.69
3.69
3.68
3.67
2.84
2.83
2.80
2.79
2.56
2.53
2.52
2.49
2.19
2.17
2.16
2.15
2.14
2.13
2.11
2.06
2.05
2.04
2.03
2.03
2.02
2.02
2.01
2.00
1.99
1.98
1.74
1.72
1.70
1.68
1.68
1.66
1.66
1.64
1.62
1.60
1.58
1.57
1.56
1.55
1.54
1.53
1.53
1.51
1.49
1.13
1.10
0.95
0.93
0.91
Me
Me
N
O
O
O
O
CF3
F3C
0.97
1.00
0.99
1.00
1.02
1.02
1.11
2.06
2.05
1.04
2.95
2.91
3.11
f1 (ppm)

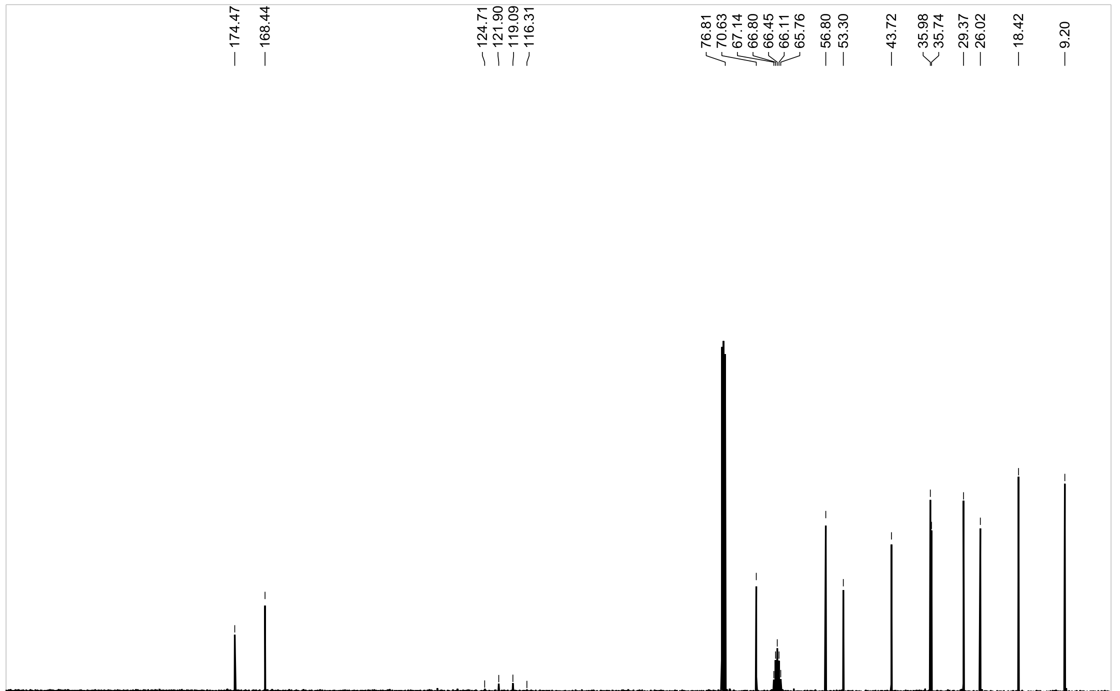
174.47
168.44
124.71
121.90
119.09
116.31
76.81
70.63
67.14
66.80
66.45
66.11
65.76
56.80
53.30
43.72
35.98
35.74
29.37
26.02
18.42
9.20
f1 (ppm)

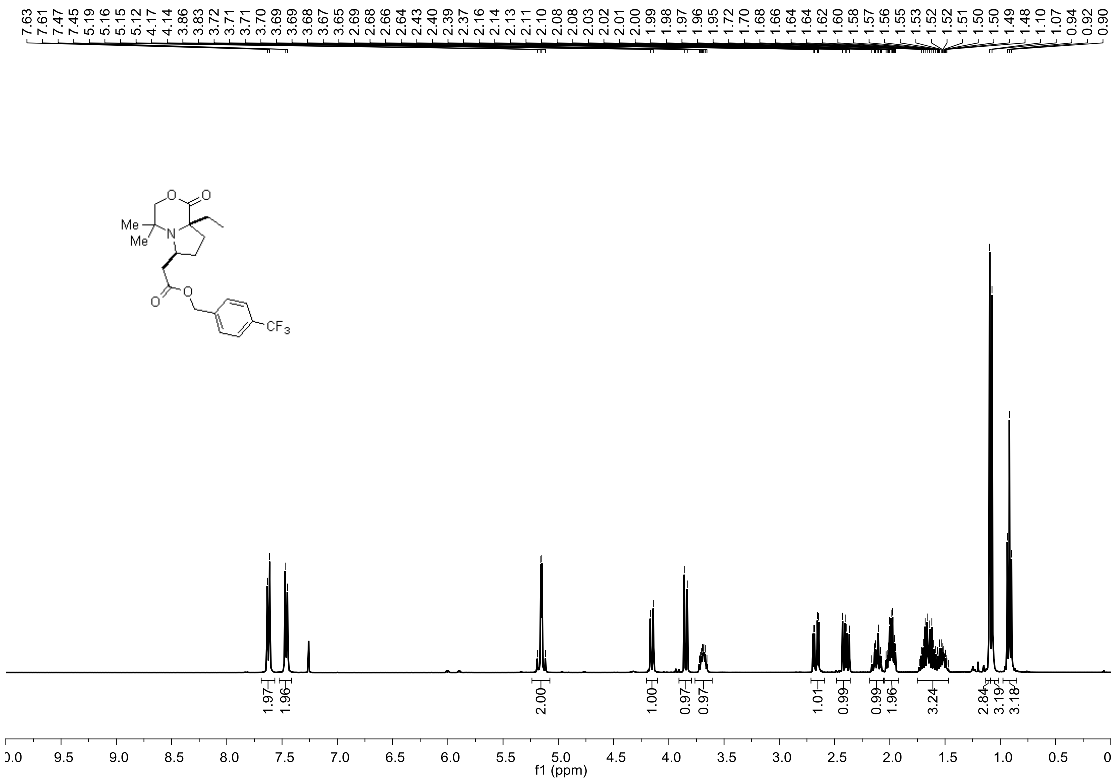
7.63
7.61
7.47
7.45
5.19
5.16
5.15
5.12
4.17
4.14
3.86
3.83
3.72
3.71
3.70
3.69
3.69
3.68
3.67
3.65
2.69
2.68
2.66
2.64
2.43
2.40
2.39
2.37
2.16
2.14
2.13
2.11
2.10
2.08
2.08
2.03
2.02
2.01
2.00
1.99
1.98
1.97
1.96
1.95
1.72
1.70
1.68
1.66
1.64
1.64
1.62
1.60
1.58
1.57
1.56
1.55
1.53
1.52
1.52
1.51
1.50
1.50
1.49
1.48
1.10
1.07
0.94
0.92
0.90
Me
Me
N
O
O
O
O
CF3
1.97
1.96
2.00
1.00
0.97
0.97
1.01
0.99
0.99
1.96
3.24
2.84
3.19
3.18
f1 (ppm)

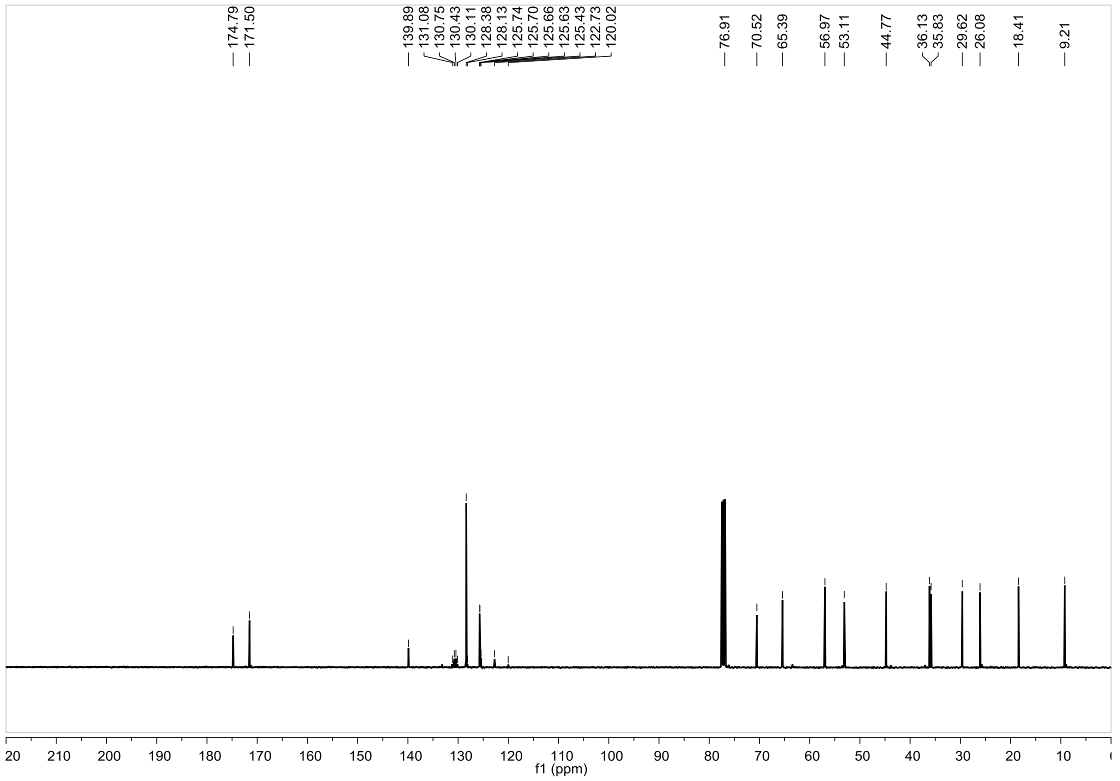
174.79
171.50
139.89
131.08
130.75
130.43
130.11
128.38
128.13
125.74
125.70
125.66
125.63
125.43
122.73
120.02
76.91
70.52
65.39
56.97
53.11
44.77
36.13
35.83
29.62
26.08
18.41
9.21
f1 (ppm)

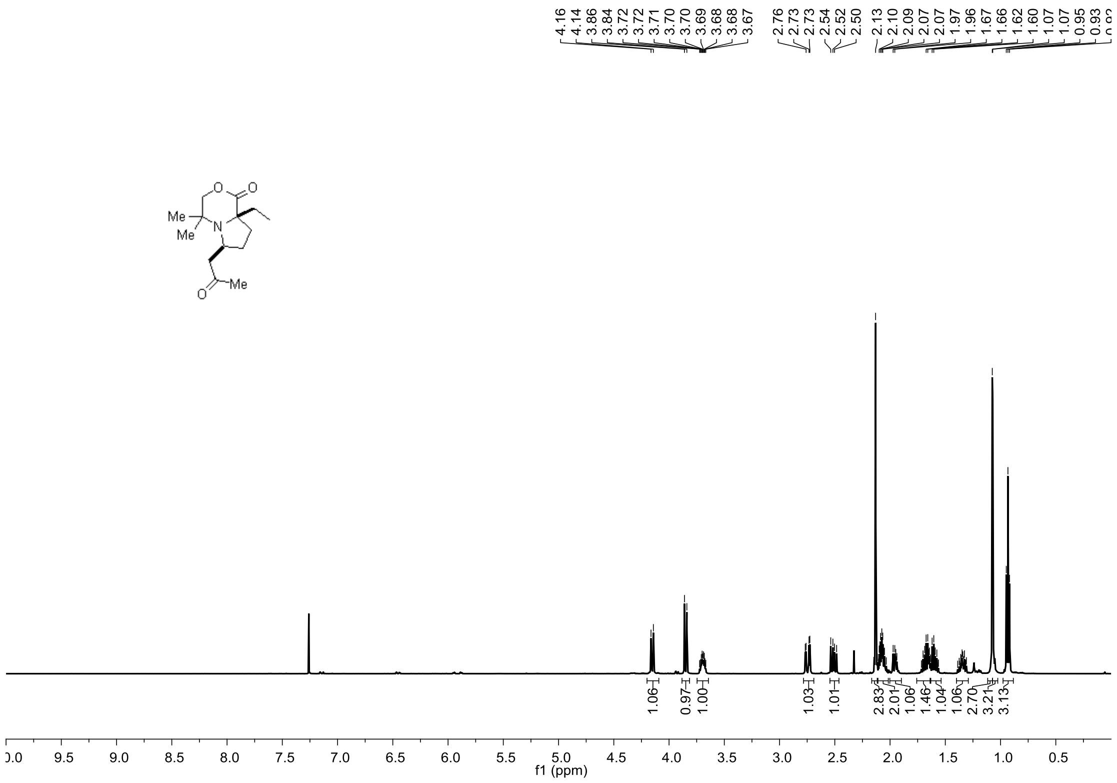
4.16
4.14
3.86
3.84
3.72
3.72
3.71
3.70
3.69
3.68
3.68
3.67
2.76
2.73
2.73
2.54
2.52
2.50
2.13
2.10
2.09
2.07
2.07
1.97
1.96
1.67
1.66
1.62
1.60
1.07
0.95
0.93
O
O
Me
Me
N
O
Me
1.06
0.97
1.00
1.03
1.01
2.83
2.01
1.06
1.46
1.04
1.06
2.70
3.21
3.13
9.5
9.0
8.5
8.0
7.5
7.0
6.5
6.0
5.5
5.0
4.5
4.0
3.5
3.0
2.5
2.0
1.5
1.0
0.5
f1 (ppm)

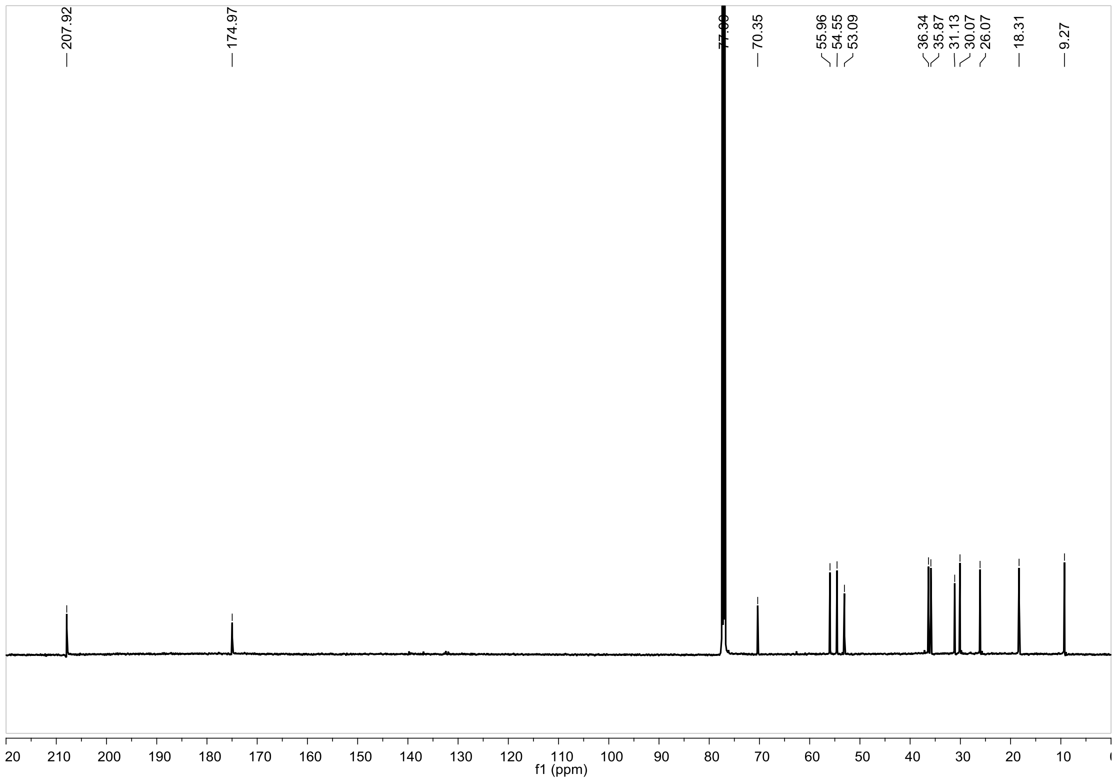
207.92
174.97
70.35
55.96
54.55
53.09
36.34
35.87
31.13
30.07
26.07
18.31
9.27
210
200
190
180
170
160
150
140
130
120
110
100
90
80
70
60
50
40
30
20
10
f1 (ppm)

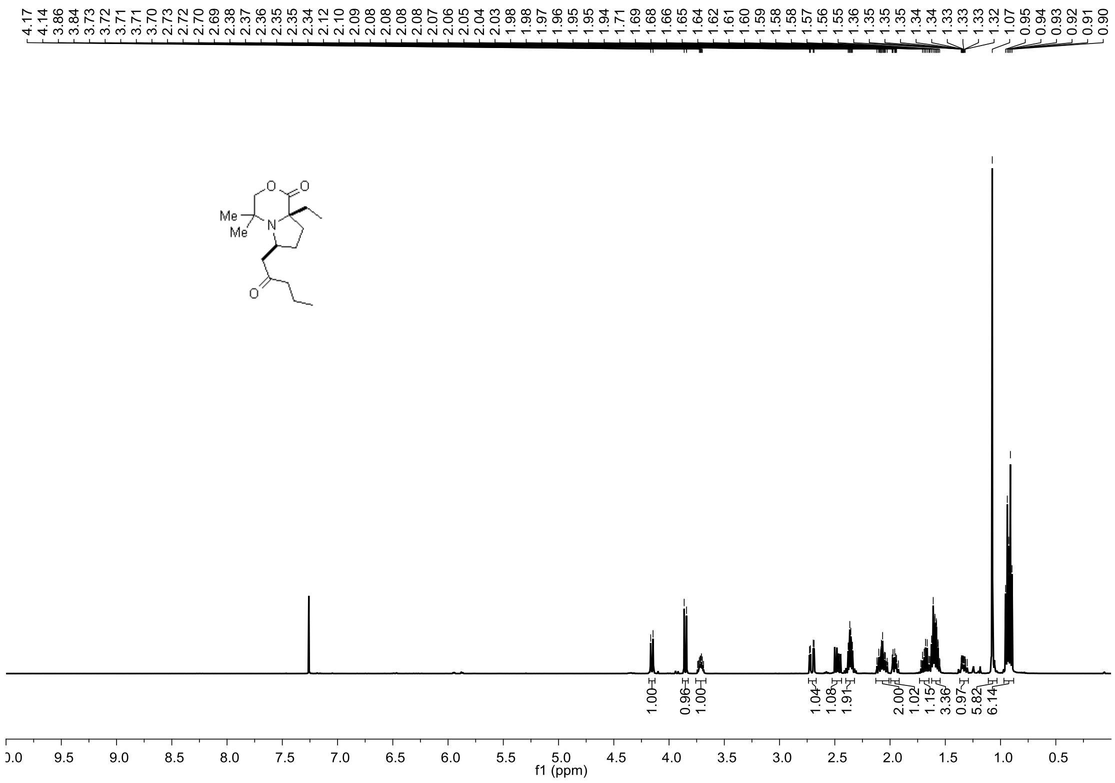
4.17 4.14 3.86 3.84 3.73 3.72 3.71 3.70 2.73 2.72 2.70 2.69 2.38 2.37 2.36 2.35 2.35 2.34 2.12 2.10 2.09 2.08 2.08 2.08 2.08 2.07 2.06 2.05 2.04 2.03 1.98 1.98 1.97 1.96 1.95 1.95 1.94 1.71 1.69 1.68 1.65 1.64 1.62 1.61 1.60 1.59 1.58 1.58 1.57 1.56 1.55 1.36 1.35 1.35 1.35 1.34 1.33 1.33 1.33 1.32 1.07 0.95 0.94 0.93 0.92 0.91 0.90
O
O
Me
Me
N
O
1.00 0.96 1.00 1.04 1.08 1.91 2.00 1.02 1.15 3.36 0.97 5.82 6.14
).0 9.5 9.0 8.5 8.0 7.5 7.0 6.5 6.0 5.5 5.0 4.5 4.0 3.5 3.0 2.5 2.0 1.5 1.0 0.5
f1 (ppm)

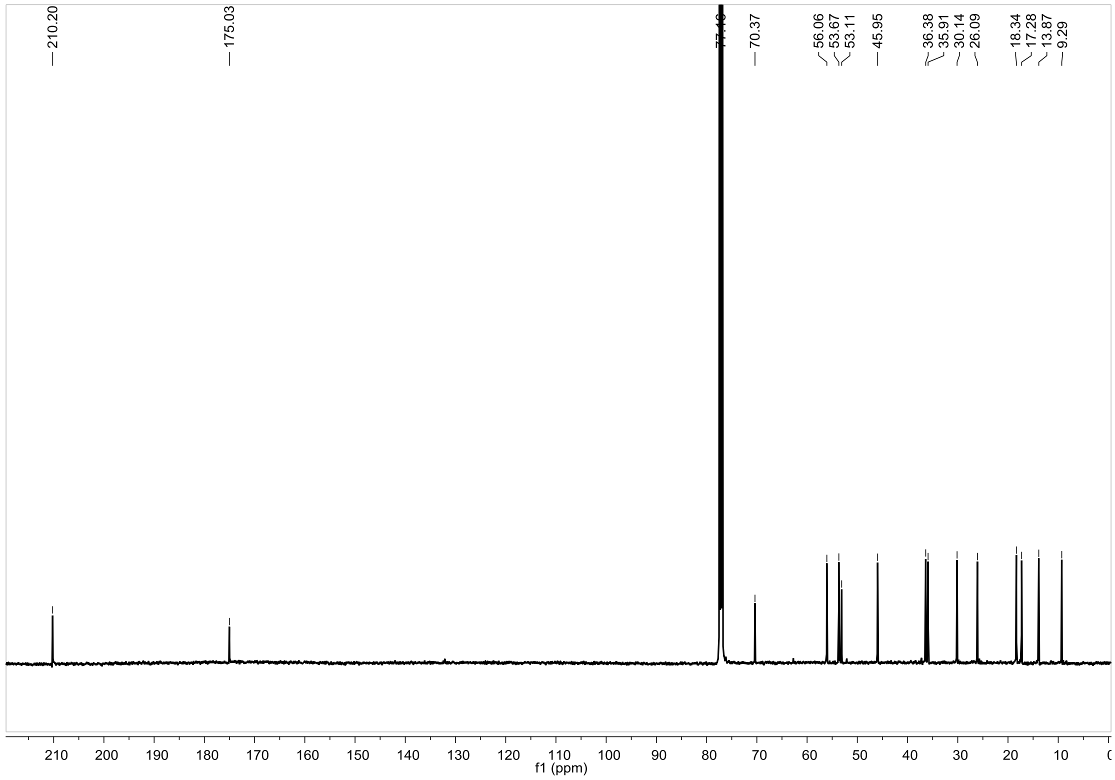
210.20
175.03
70.37
56.06 53.67 53.11
45.95
36.38 35.91 30.14 26.09
18.34 17.28 13.87 9.29
210 200 190 180 170 160 150 140 130 120 110 100 90 80 70 60 50 40 30 20 10
f1 (ppm)

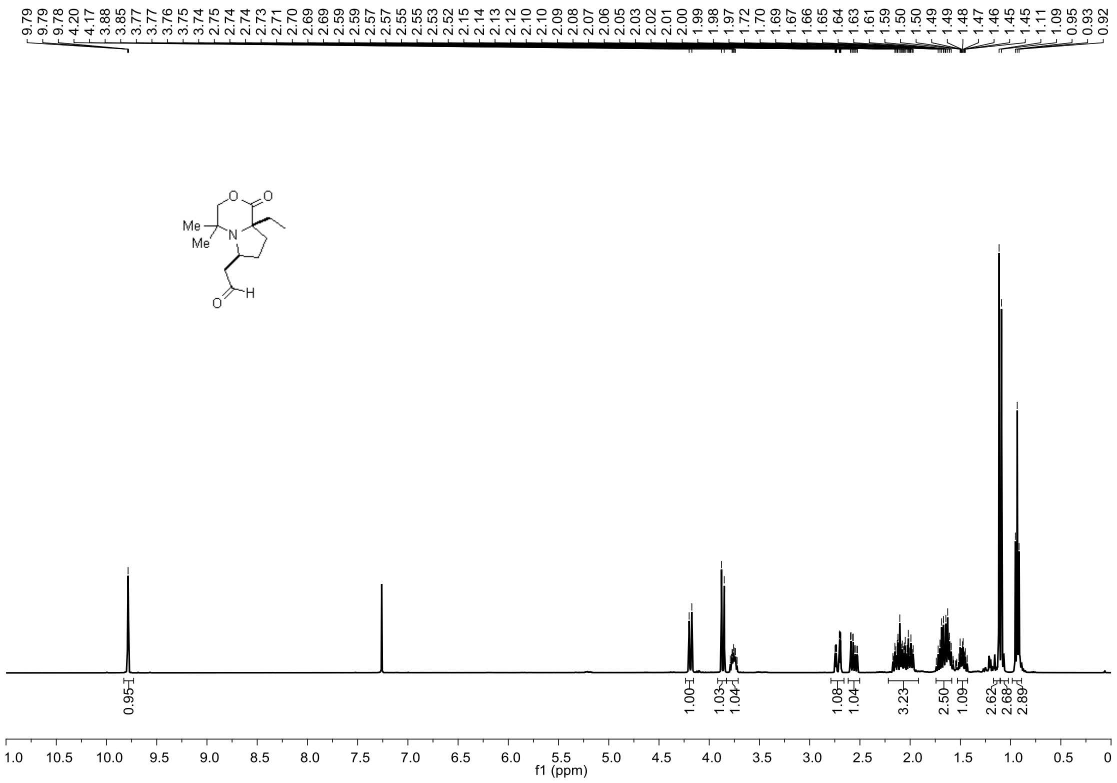
9.79 9.79 9.78 4.20 4.17 3.88 3.85 3.77 3.77 3.76 3.75 3.74 2.75 2.74 2.74 2.73 2.71 2.70 2.69 2.69 2.59 2.59 2.57 2.57 2.55 2.53 2.52 2.15 2.14 2.13 2.12 2.10 2.09 2.08 2.07 2.06 2.05 2.03 2.02 2.01 2.00 1.99 1.98 1.97 1.72 1.70 1.69 1.67 1.66 1.65 1.64 1.63 1.61 1.59 1.50 1.50 1.49 1.48 1.47 1.46 1.45 1.45 1.11 1.09 0.95 0.93 0.92
Me
Me
N
O
O
O
H
0.95
1.00
1.03
1.04
1.08
1.04
3.23
2.50
1.09
2.62
2.68
2.89
f1 (ppm)

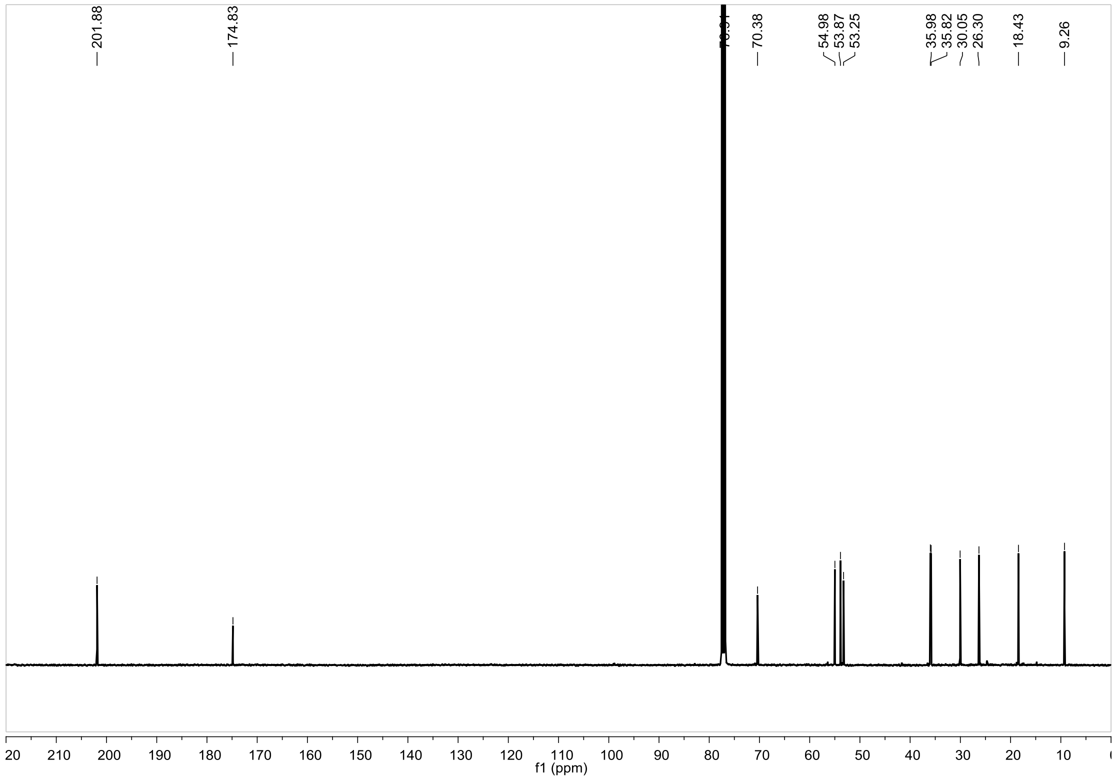
201.88
174.83
70.38
54.98
53.87
53.25
35.98
35.82
30.05
26.30
18.43
9.26
f1 (ppm)

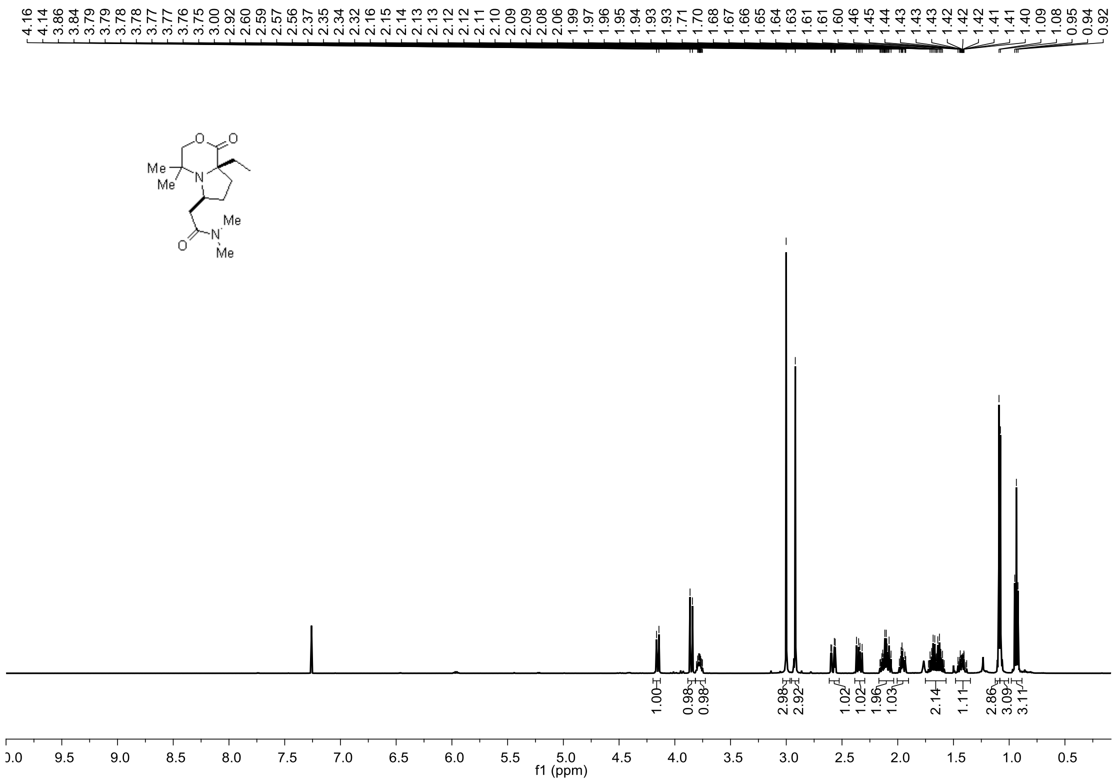
4.16 4.14 3.86 3.84 3.79 3.78 3.78 3.77 3.76 3.75 3.00 2.92 2.60 2.59 2.57 2.56 2.37 2.35 2.34 2.32 2.16 2.15 2.14 2.13 2.13 2.12 2.12 2.11 2.10 2.09 2.09 2.08 2.06 1.99 1.97 1.96 1.95 1.94 1.93 1.93 1.71 1.70 1.68 1.67 1.66 1.65 1.64 1.63 1.61 1.61 1.60 1.46 1.45 1.44 1.43 1.43 1.42 1.42 1.42 1.41 1.40 1.09 1.08 0.95 0.94 0.92
Me
Me
N
O
O
N
Me
Me
O
1.00
0.98
0.98
2.98
2.92
1.02
1.02
1.96
1.03
2.14
1.11
2.86
3.09
3.11
f1 (ppm)

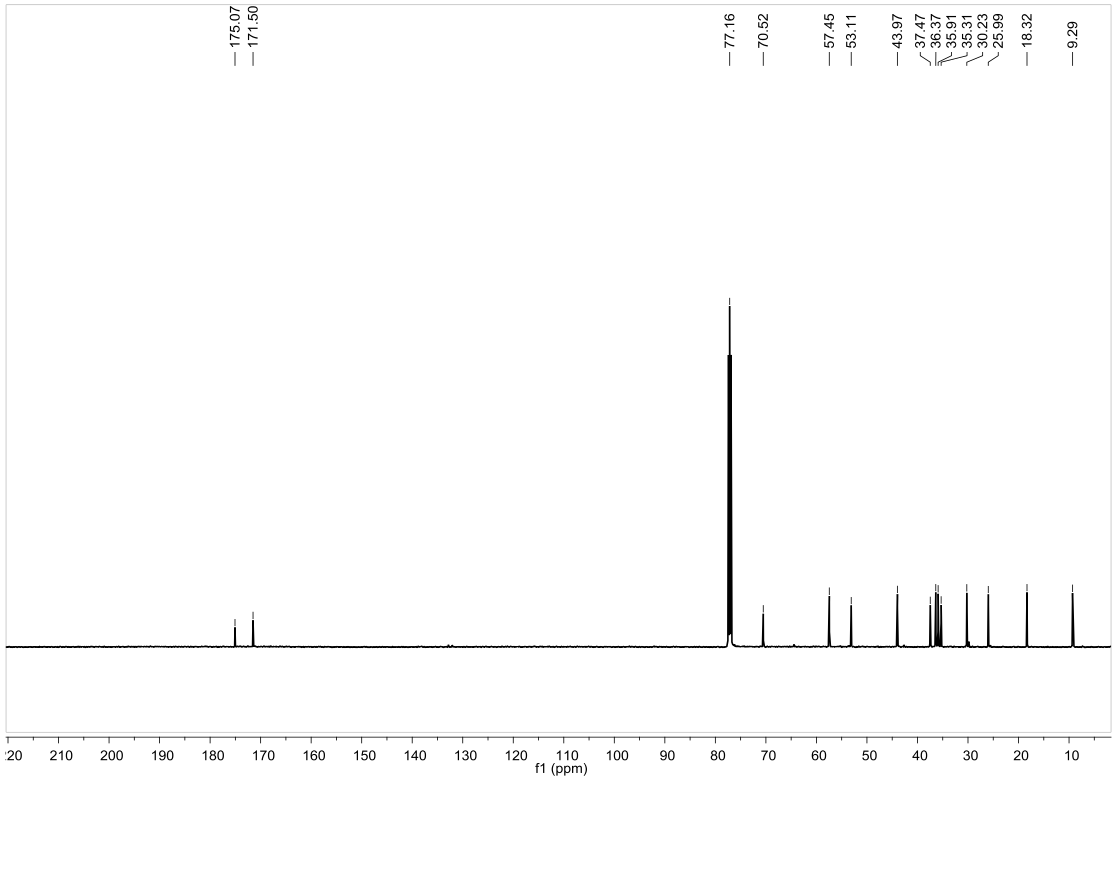
175.07
171.50
77.16
70.52
57.45
53.11
43.97
37.47
36.37
35.91
35.31
30.23
25.99
18.32
9.29
f1 (ppm)

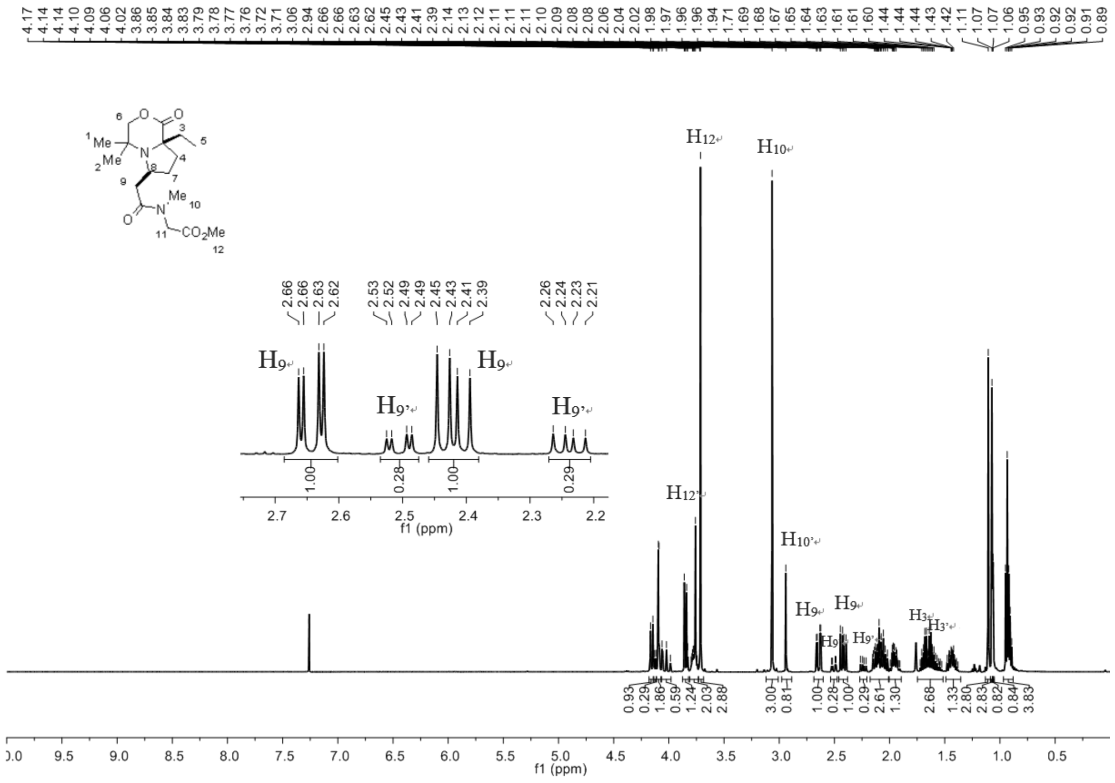
H12
H10
H9
H9'
H12'
H10'
H3
H3'
f1 (ppm)

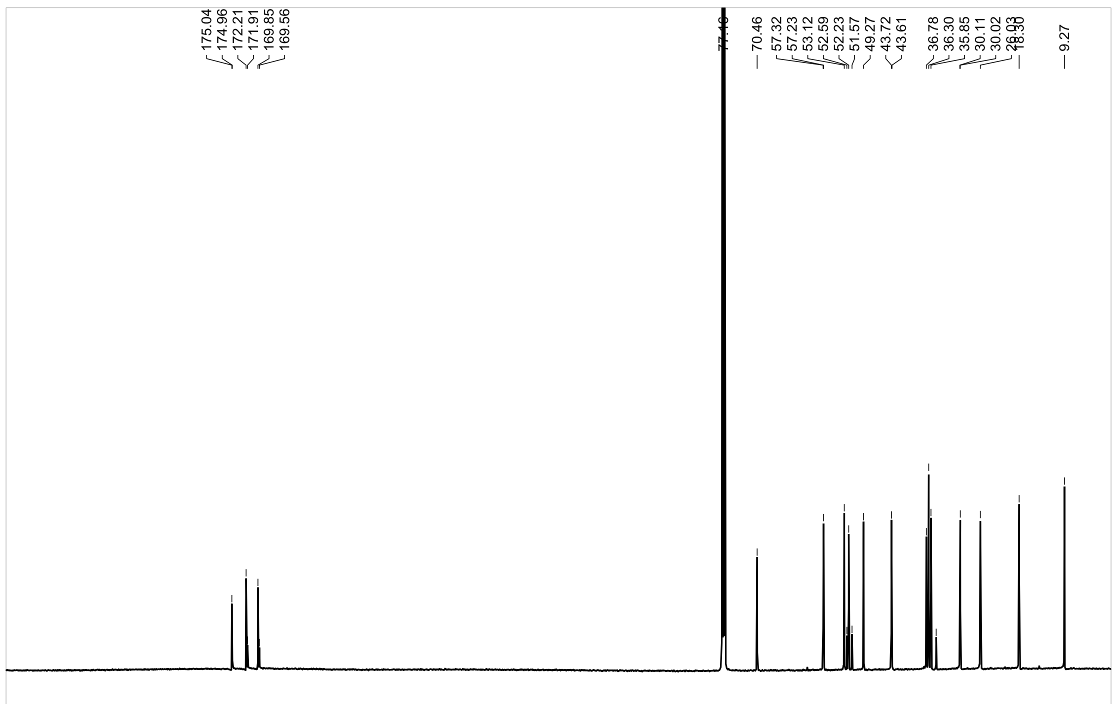
175.04
174.96
172.21
171.91
169.85
169.56
70.46
57.32
57.23
53.12
52.59
52.23
51.57
49.27
43.72
43.61
36.78
36.30
35.85
30.11
30.02
9.27
f1 (ppm)

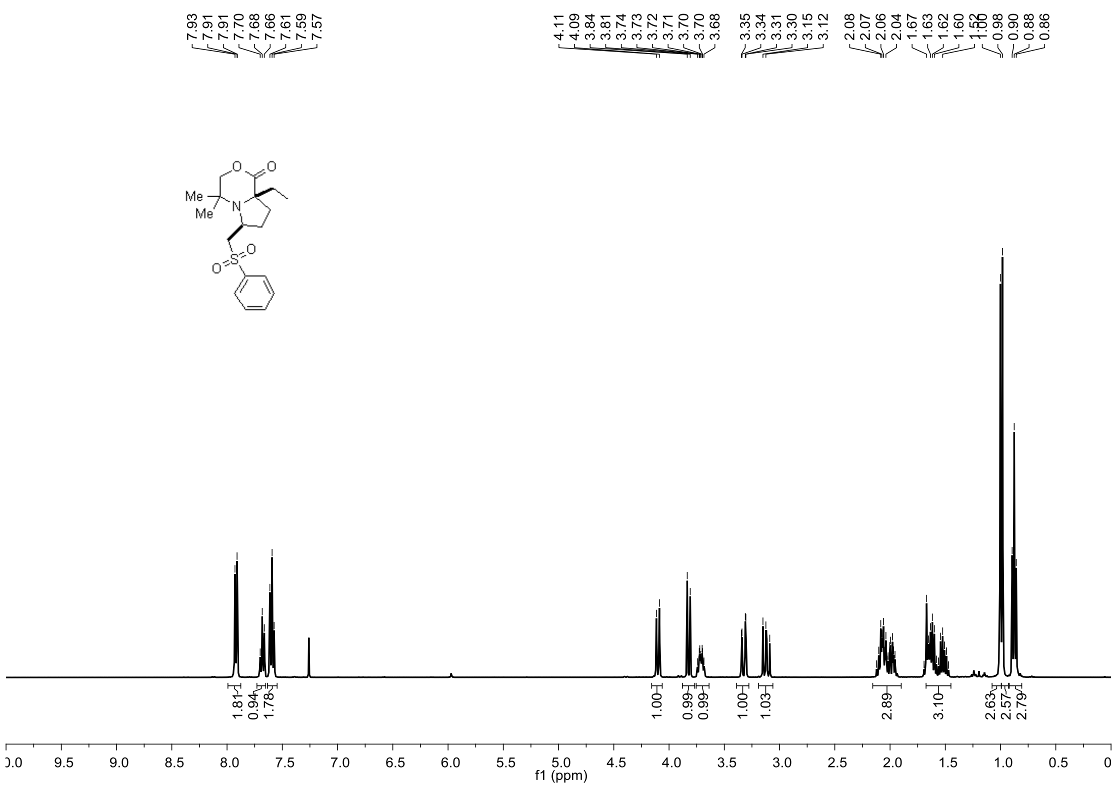
7.93 7.91 7.91 7.70 7.68 7.66 7.61 7.59 7.57
4.11 4.09 3.84 3.81 3.74 3.73 3.72 3.71 3.70 3.70 3.68
3.35 3.34 3.31 3.30 3.15 3.12
2.08 2.07 2.06 2.04 1.67 1.63 1.62 1.60 0.98 0.90 0.88 0.86
O
O
Me
Me
N
S
O
O
1.81 0.94 1.78
1.00 0.99 0.99 1.00 1.03
2.89 3.10 2.63 2.57 2.79
9.5 9.0 8.5 8.0 7.5 7.0 6.5 6.0 5.5 5.0 4.5 4.0 3.5 3.0 2.5 2.0 1.5 1.0 0.5
f1 (ppm)

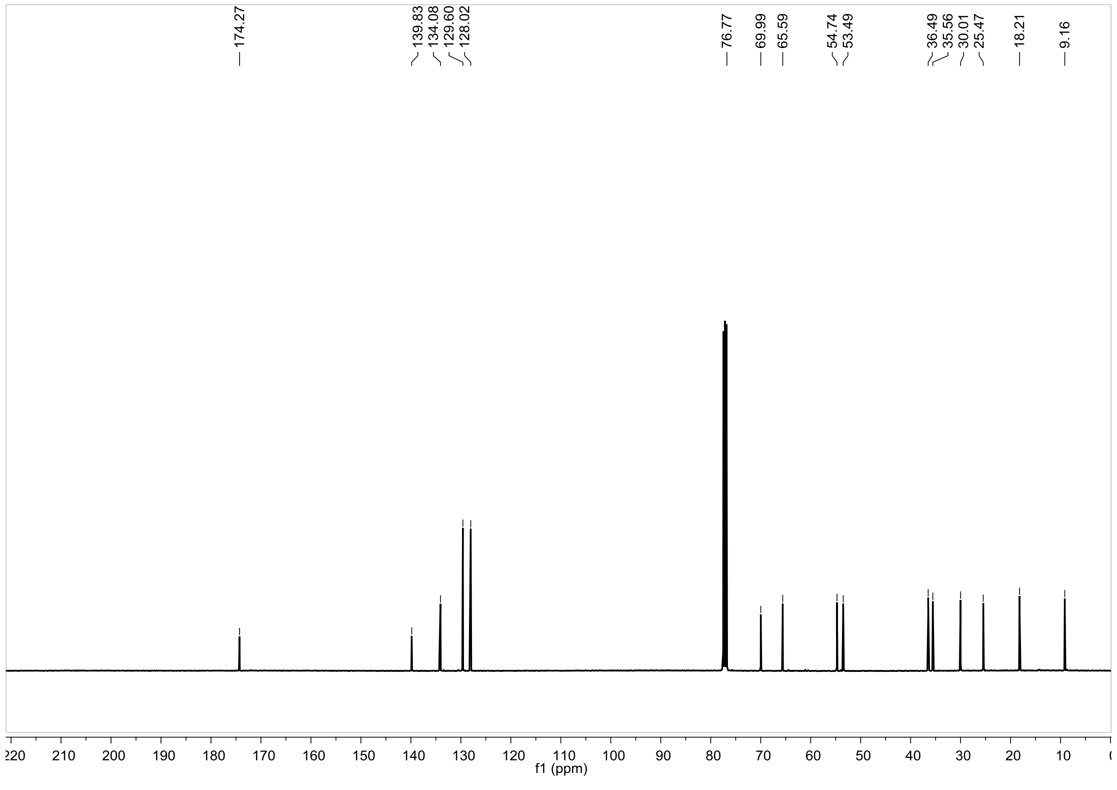
174.27
139.83 134.08 129.60 128.02
76.77 69.99 65.59
54.74 53.49
36.49 35.56 30.01 25.47 18.21 9.16
210 200 190 180 170 160 150 140 130 120 110 100 90 80 70 60 50 40 30 20 10
f1 (ppm)

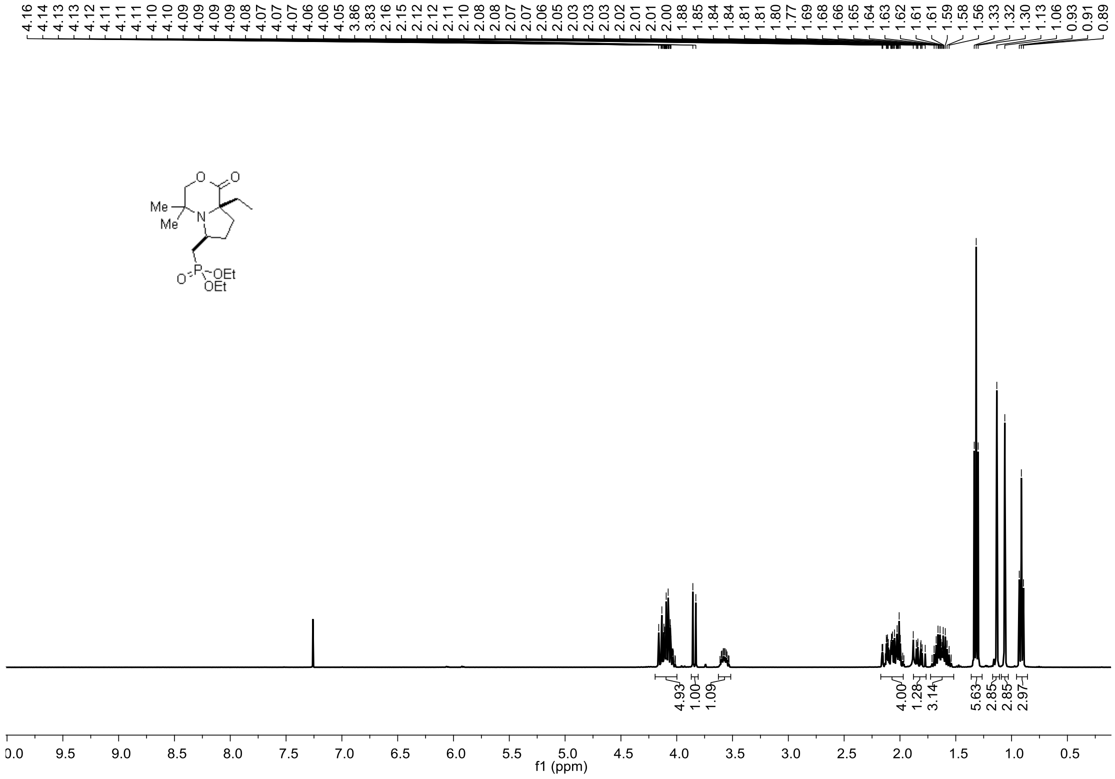
4.16 4.14 4.13 4.12 4.11 4.11 4.10 4.09 4.09 4.09 4.08 4.07 4.07 4.06 4.05 3.86 3.83 2.16 2.15 2.12 2.12 2.11 2.10 2.08 2.08 2.07 2.06 2.05 2.03 2.03 2.03 2.02 2.01 2.01 2.00 1.88 1.85 1.84 1.84 1.81 1.80 1.77 1.69 1.68 1.66 1.65 1.64 1.63 1.62 1.61 1.61 1.59 1.58 1.56 1.33 1.32 1.30 1.13 1.06 0.93 0.91 0.89
Me
Me
N
O
O
P
OEt
OEt
4.93
1.00
1.09
4.00
1.28
3.14
5.63
2.85
2.85
2.97
f1 (ppm)

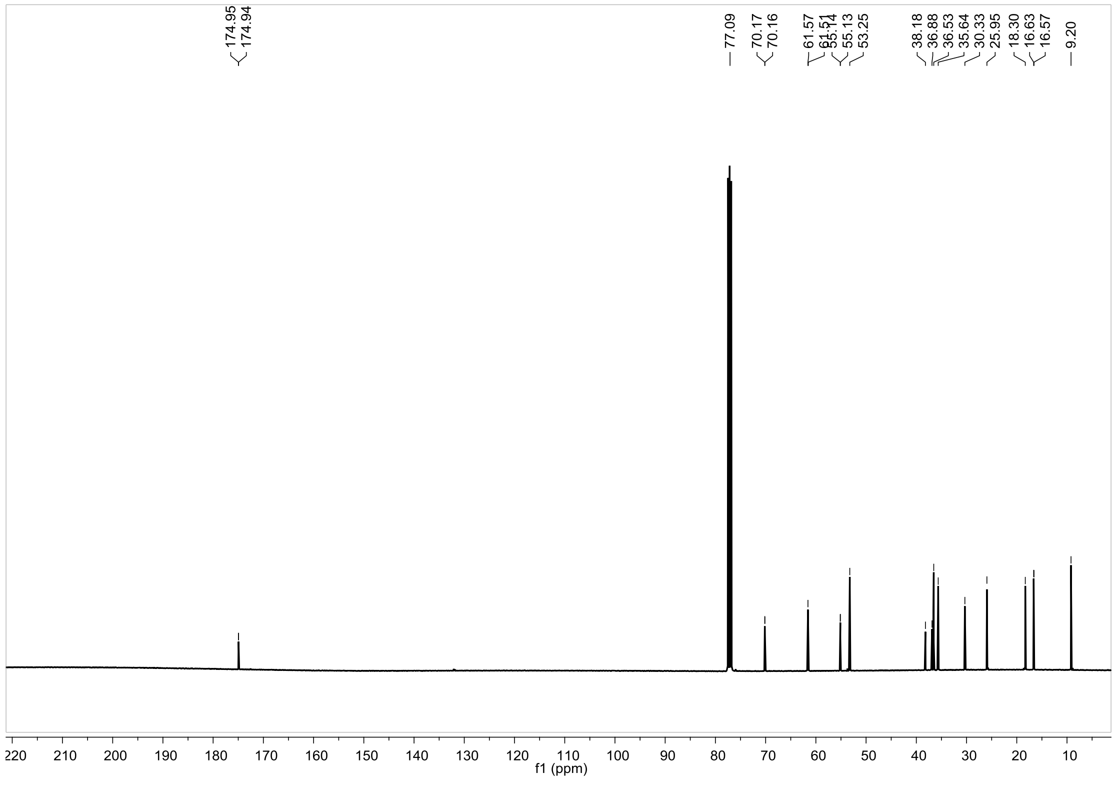
174.95 174.94
77.09
70.17 70.16
61.57 61.51 55.14 55.13 53.25
38.18 36.88 36.53 35.64 30.33 25.95 18.30 16.63 16.57
9.20
f1 (ppm)

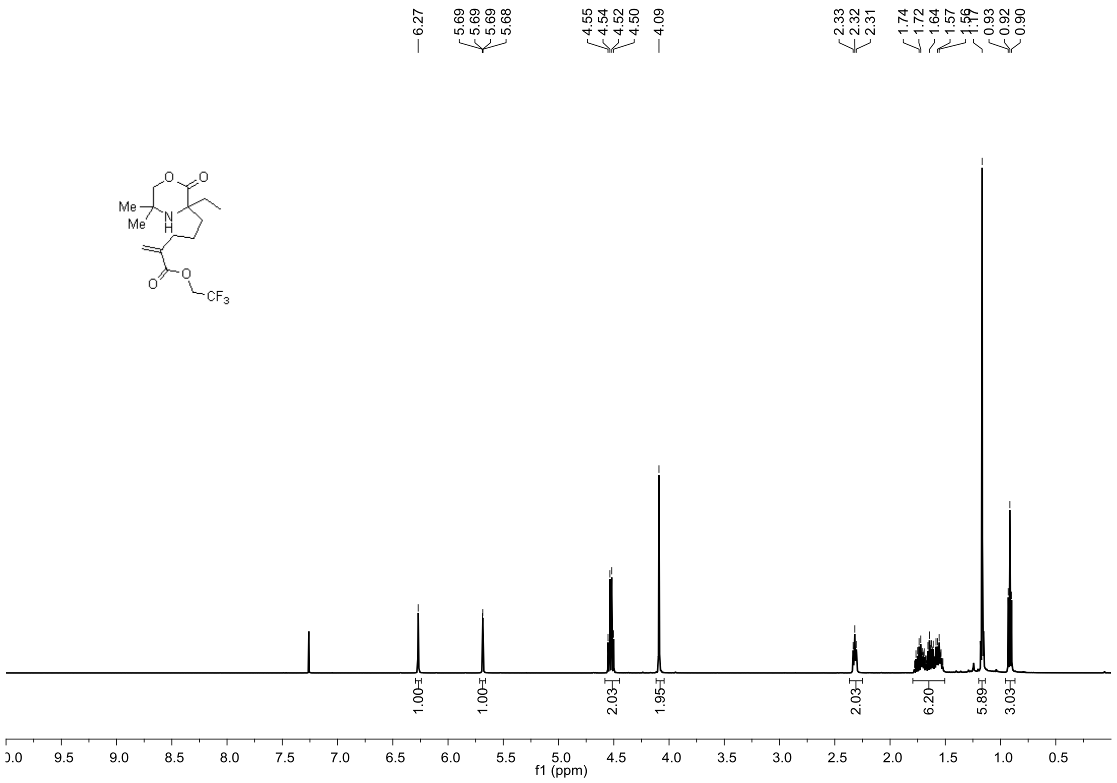
6.27
5.69
5.69
5.69
5.68
4.55
4.54
4.52
4.50
4.09
2.33
2.32
2.31
1.74
1.72
1.64
1.57
1.56
1.17
0.93
0.92
0.90
Me
Me
H
N
O
O
O
O
CF_3
1.00
1.00
2.03
1.95
2.03
6.20
5.89
3.03
f1 (ppm)

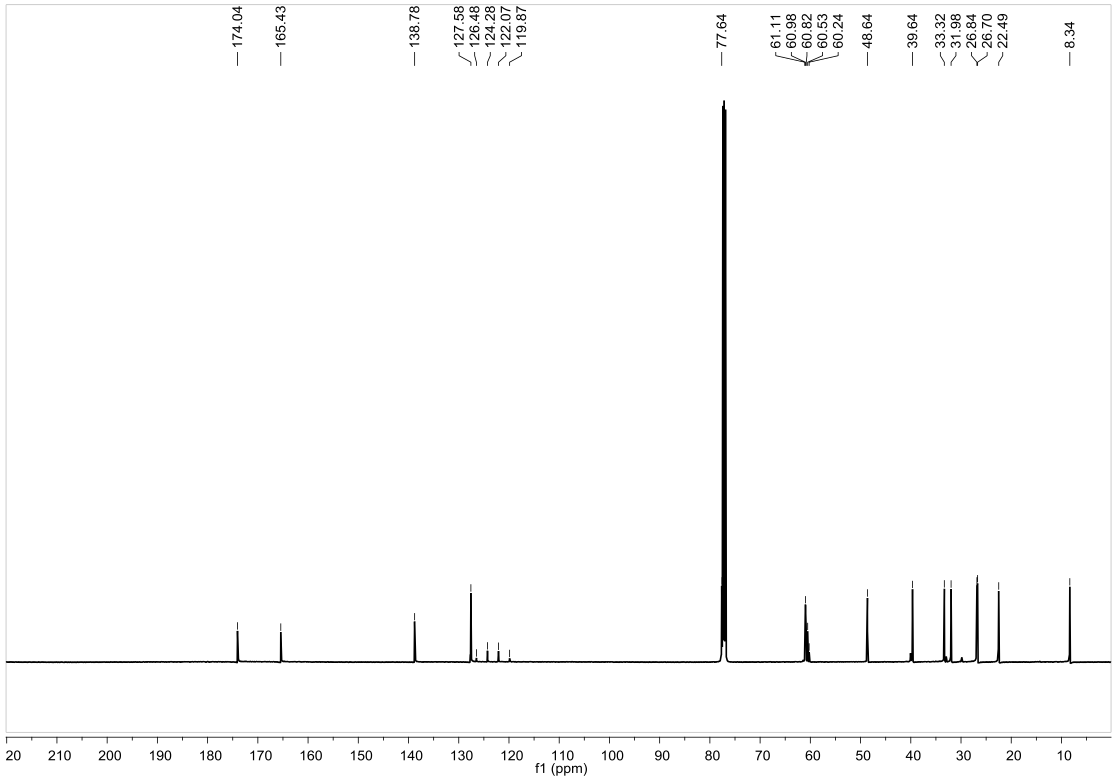
174.04
165.43
138.78
127.58
126.48
124.28
122.07
119.87
77.64
61.11
60.98
60.82
60.53
60.24
48.64
39.64
33.32
31.98
26.84
26.70
22.49
8.34
f1 (ppm)

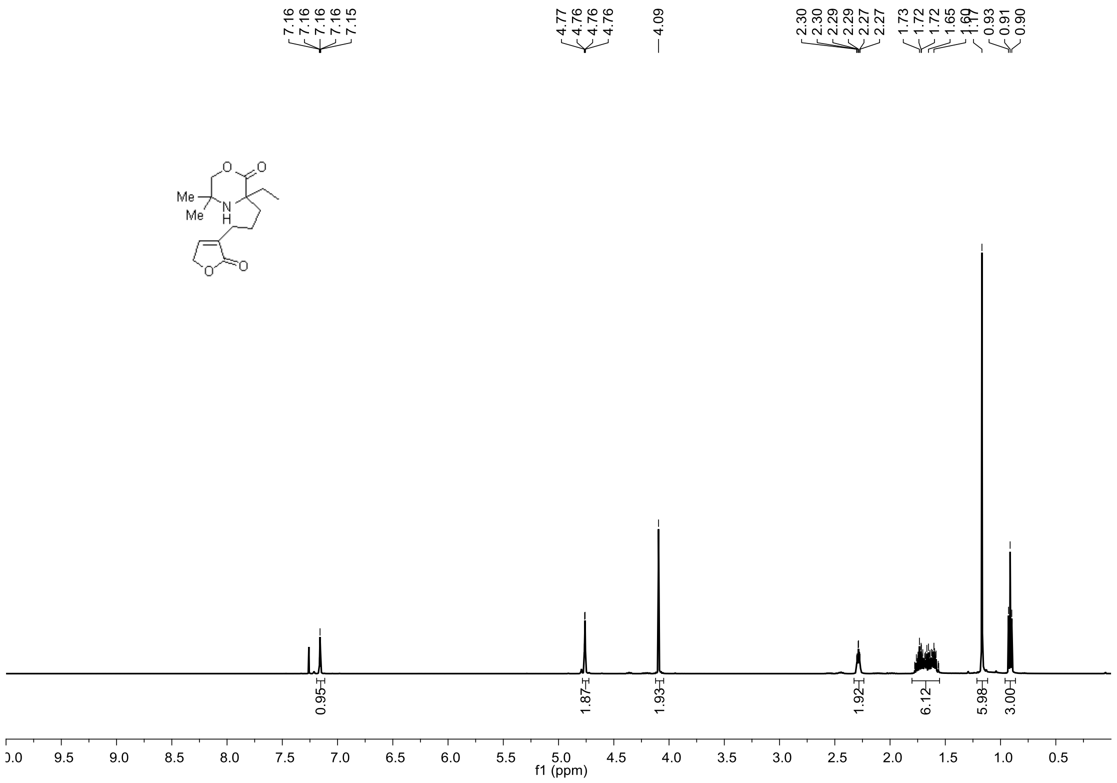
7.16
7.16
7.16
7.16
7.15
4.77
4.76
4.76
4.76
4.09
2.30
2.30
2.29
2.29
2.27
2.27
1.73
1.72
1.72
1.65
1.60
1.17
0.93
0.91
0.90
Me
Me
N
H
O
O
O
O
0.95
1.87
1.93
1.92
6.12
5.98
3.00
f1 (ppm)

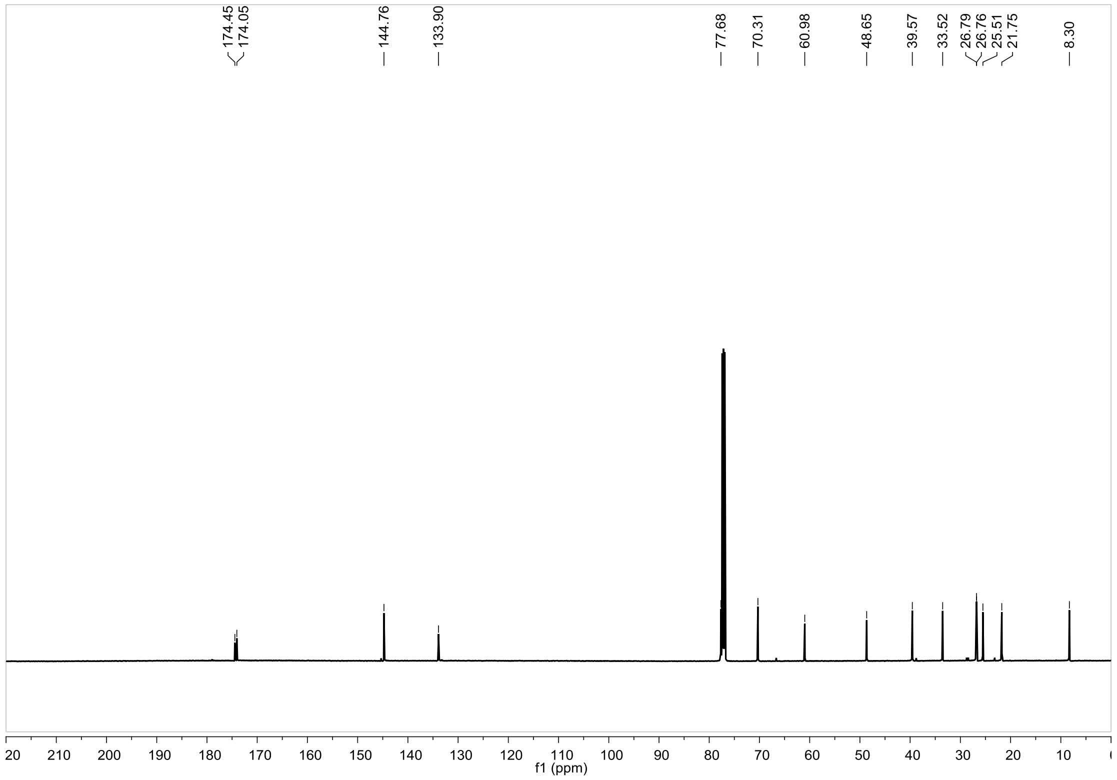
174.45
174.05
144.76
133.90
77.68
70.31
60.98
48.65
39.57
33.52
26.79
26.76
25.51
21.75
8.30
f1 (ppm)

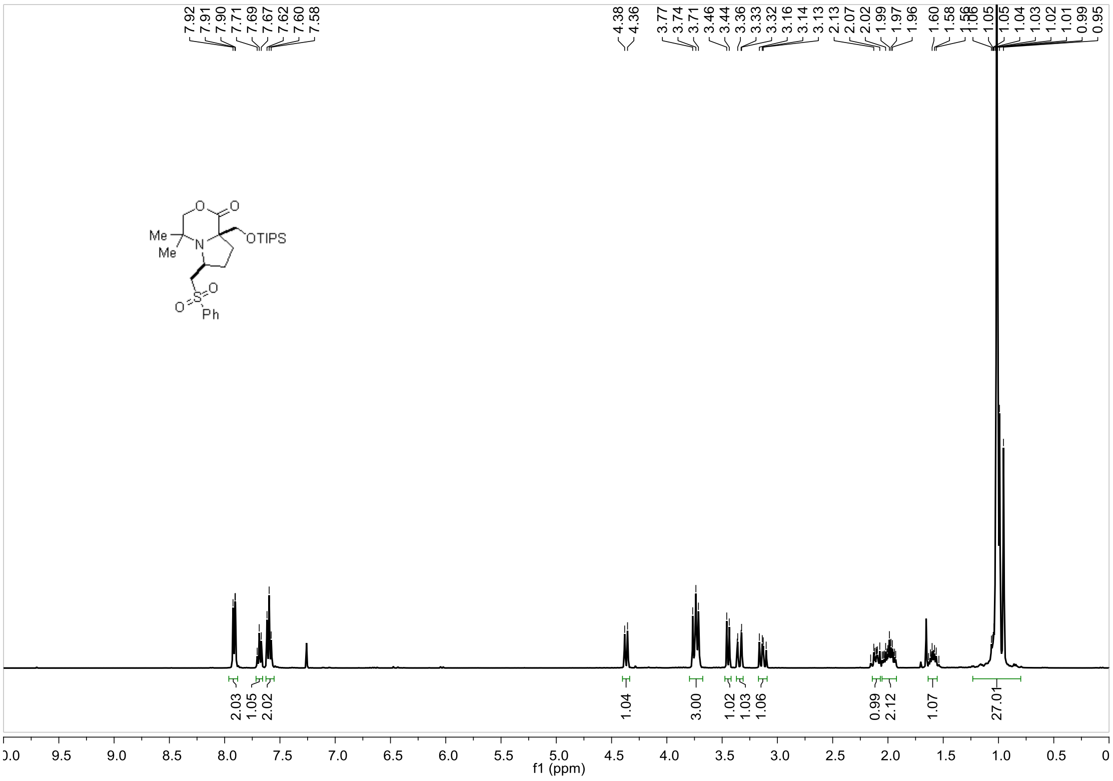
7.92 7.91 7.90 7.71 7.69 7.67 7.62 7.60 7.58
4.38 4.36
3.77 3.74 3.71 3.46 3.44 3.36 3.33 3.32 3.16 3.14 3.13 2.13 2.07 2.02 1.99 1.97 1.96 1.60 1.58 1.56 1.06 1.05 1.05 1.04 1.03 1.02 1.01 0.99 0.95
O
O
Me
Me
N
OTIPS
O=S=O
Ph
2.03 1.05 2.02
1.04
3.00 1.02 1.03 1.06
0.99 2.12 1.07 27.01
f1 (ppm)

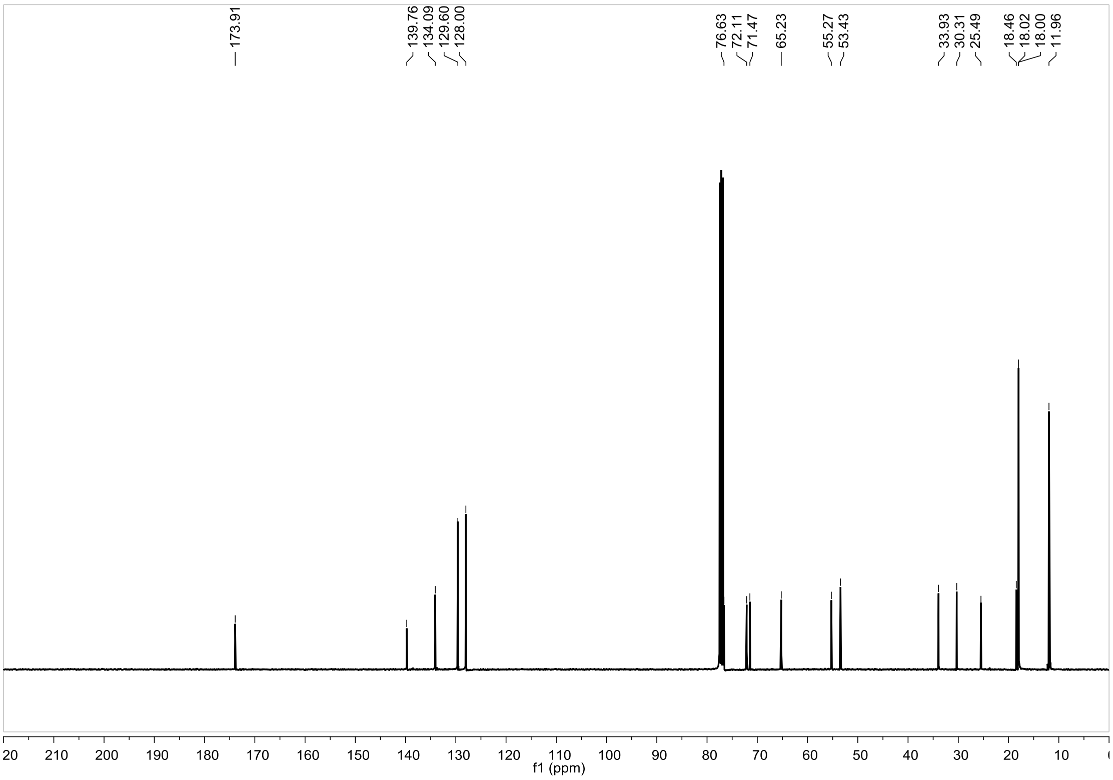
173.91
139.76 134.09 129.60 128.00
76.63 72.11 71.47 65.23 55.27 53.43
33.93 30.31 25.49 18.46 18.02 18.00 11.96
f1 (ppm)

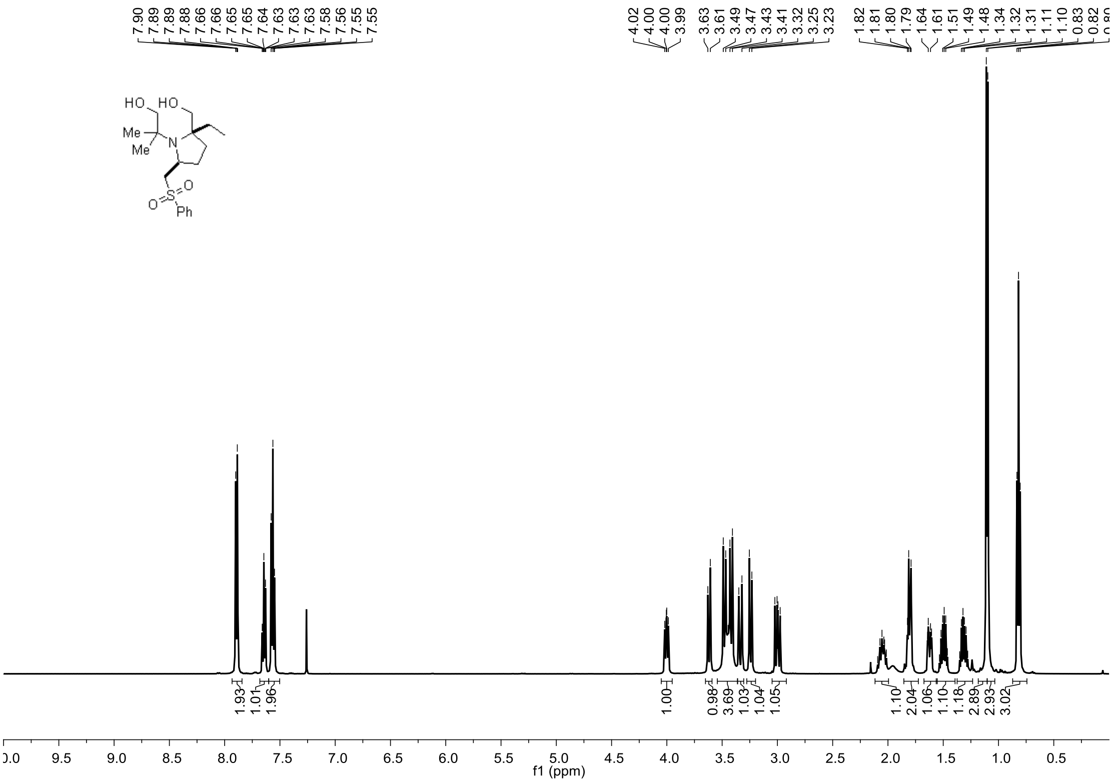

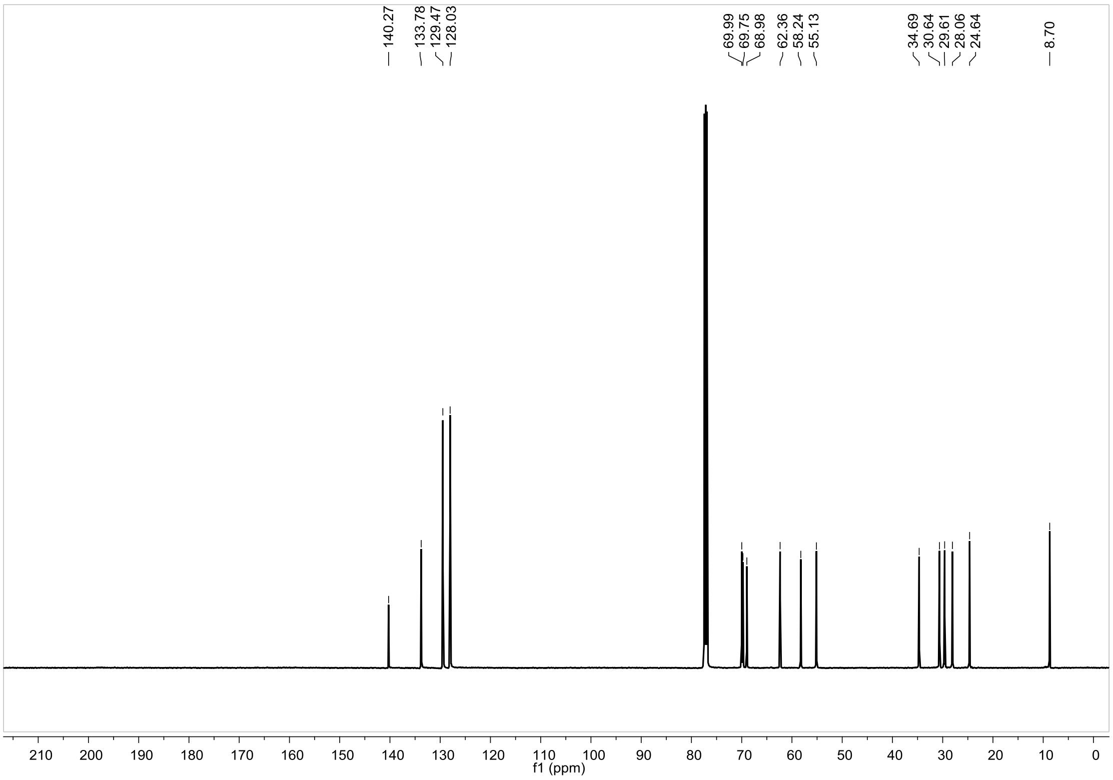

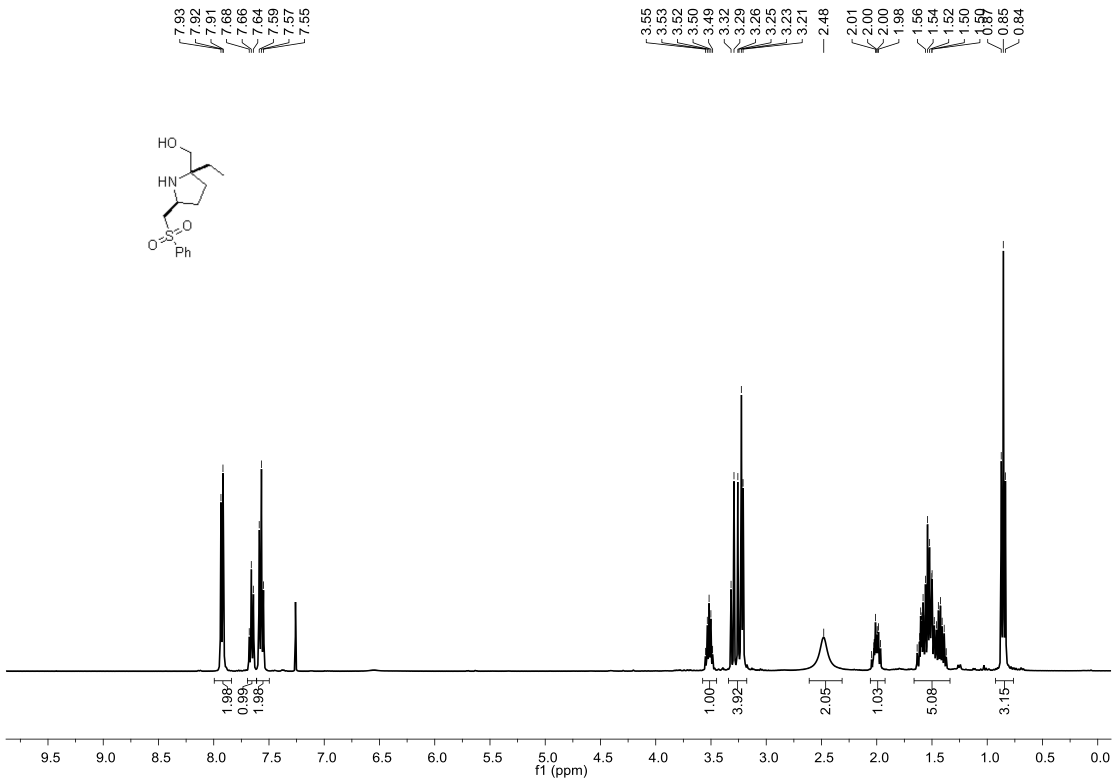
7.93
7.92
7.91
7.68
7.66
7.64
7.59
7.57
7.55
3.55
3.53
3.52
3.50
3.49
3.32
3.29
3.26
3.25
3.23
3.21
2.48
2.01
2.00
2.00
1.98
1.56
1.54
1.52
1.50
HO
HN
O
S
O
Ph
1.98
0.99
1.98
1.00
3.92
2.05
1.03
5.08
3.15
f1 (ppm)

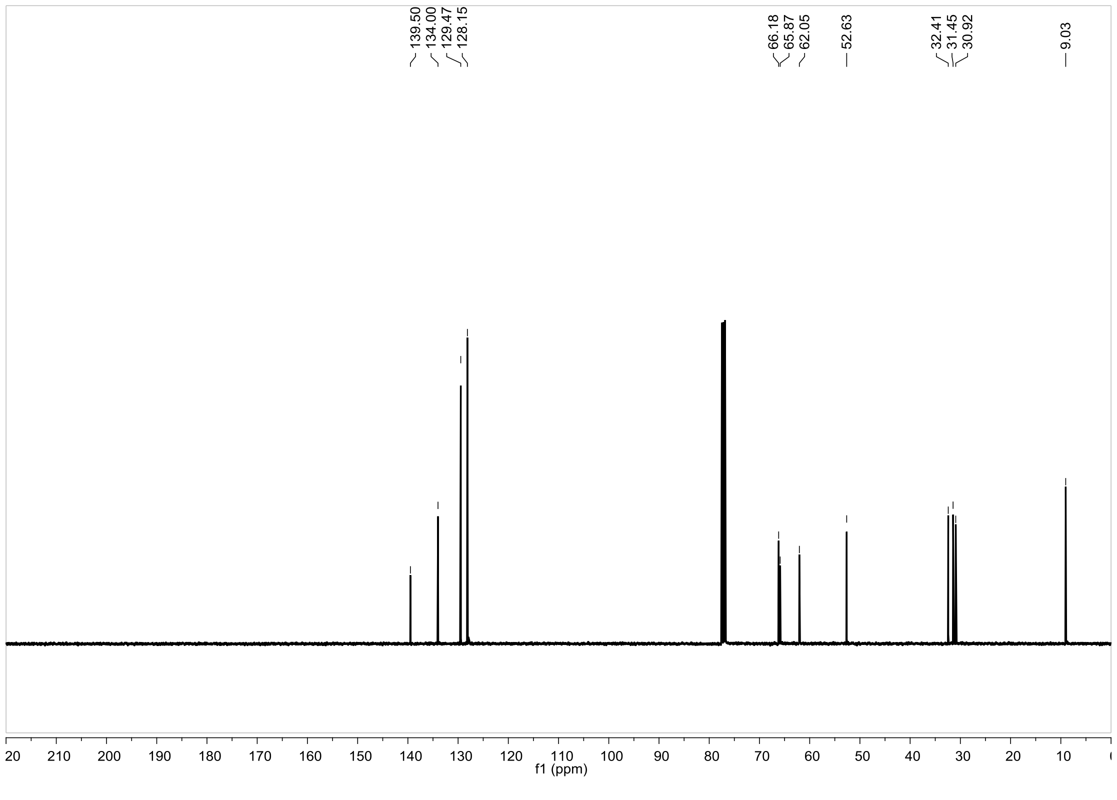
139.50
134.00
129.47
128.15
66.18
65.87
62.05
52.63
32.41
31.45
30.92
9.03
f1 (ppm)

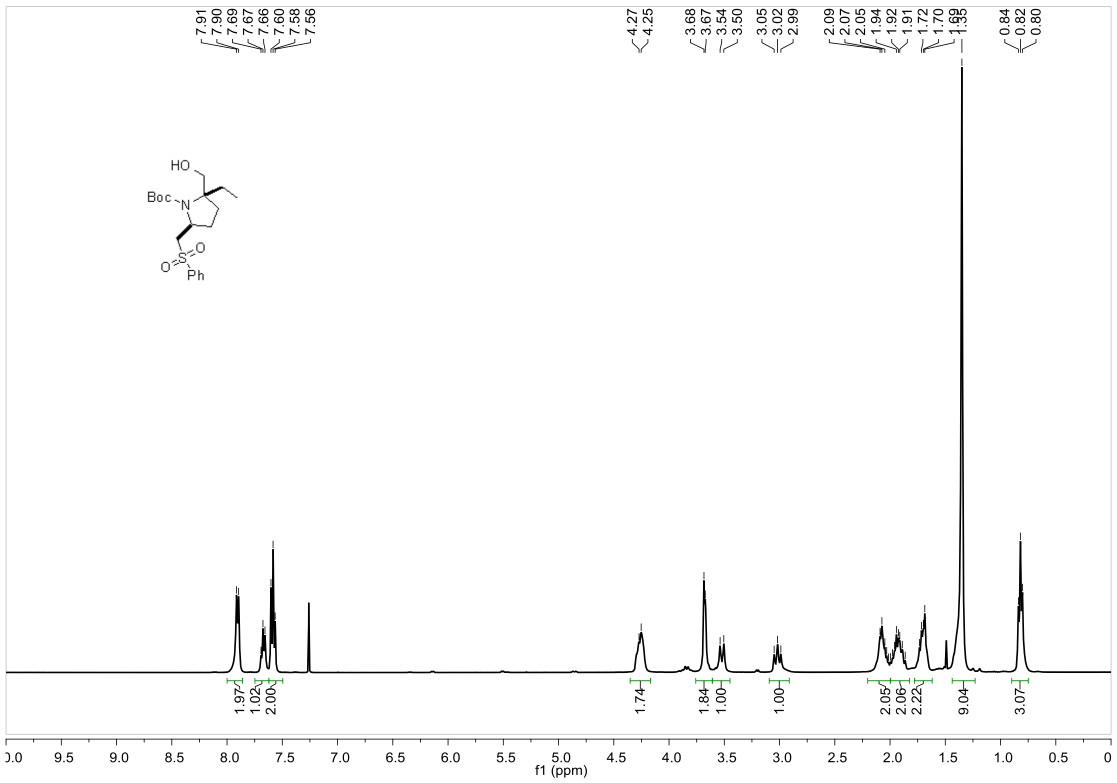
7.91
7.90
7.69
7.67
7.66
7.60
7.58
7.56
4.27
4.25
3.68
3.67
3.54
3.50
3.05
3.02
2.99
2.09
2.07
2.05
1.94
1.92
1.91
1.72
1.70
1.69
1.35
0.84
0.82
0.80
HO
Boc
N
O
S
O
Ph
1.97
1.02
2.00
1.74
1.84
1.00
1.00
2.05
2.06
2.22
9.04
3.07
f1 (ppm)

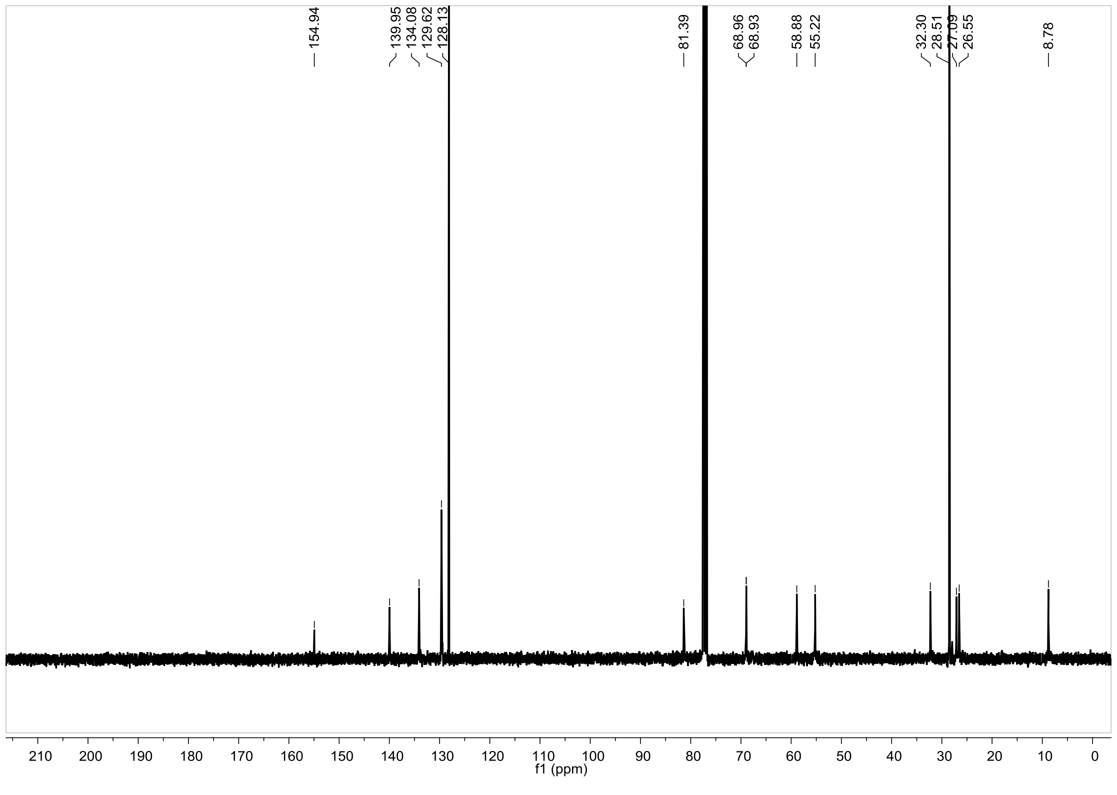
154.94
139.95
134.08
129.62
128.13
81.39
68.96
68.93
58.88
55.22
32.30
28.51
27.09
26.55
8.78
f1 (ppm)

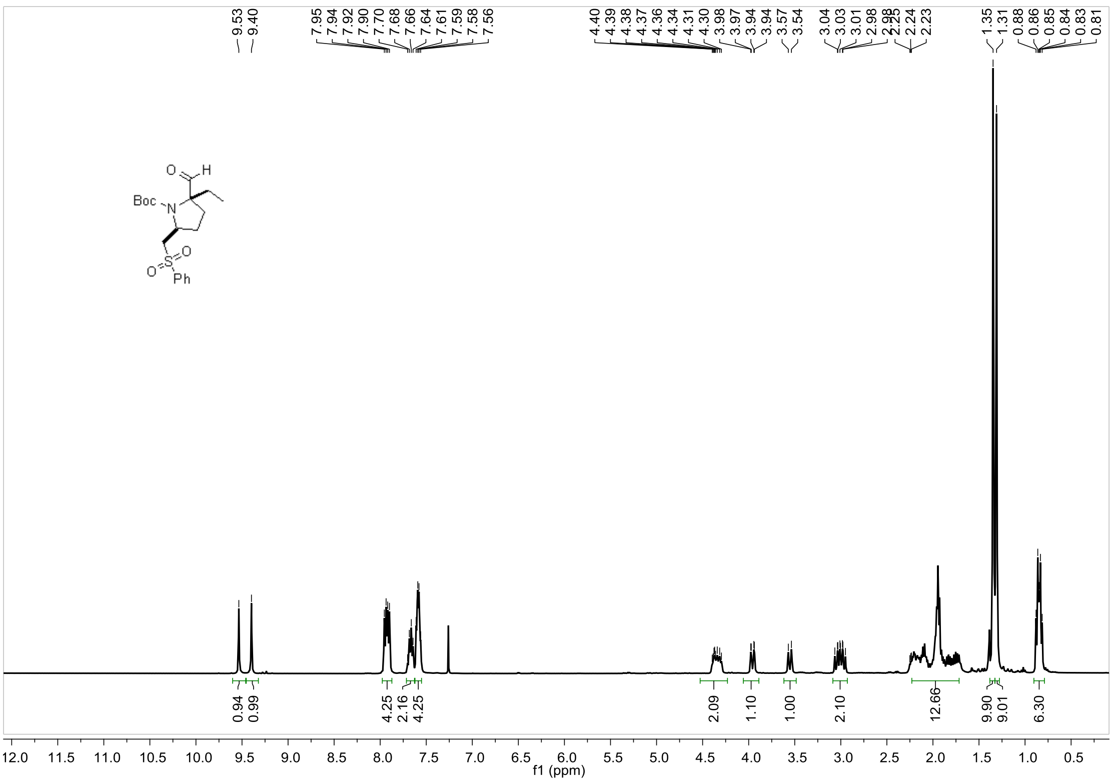
9.53
9.40
7.95
7.94
7.92
7.90
7.70
7.68
7.66
7.64
7.61
7.59
7.58
7.56
4.40
4.39
4.38
4.37
4.36
4.34
4.31
4.30
3.98
3.97
3.94
3.94
3.57
3.54
3.04
3.03
3.01
2.98
2.24
2.23
1.35
1.31
0.88
0.86
0.85
0.84
0.83
0.81
O
H
Boc
N
S
O
O
Ph
0.94
0.99
4.25
2.16
4.25
2.09
1.10
1.00
2.10
12.66
9.90
9.01
6.30
12.0 11.5 11.0 10.5 10.0 9.5 9.0 8.5 8.0 7.5 7.0 6.5 6.0 5.5 5.0 4.5 4.0 3.5 3.0 2.5 2.0 1.5 1.0 0.5
f1 (ppm)

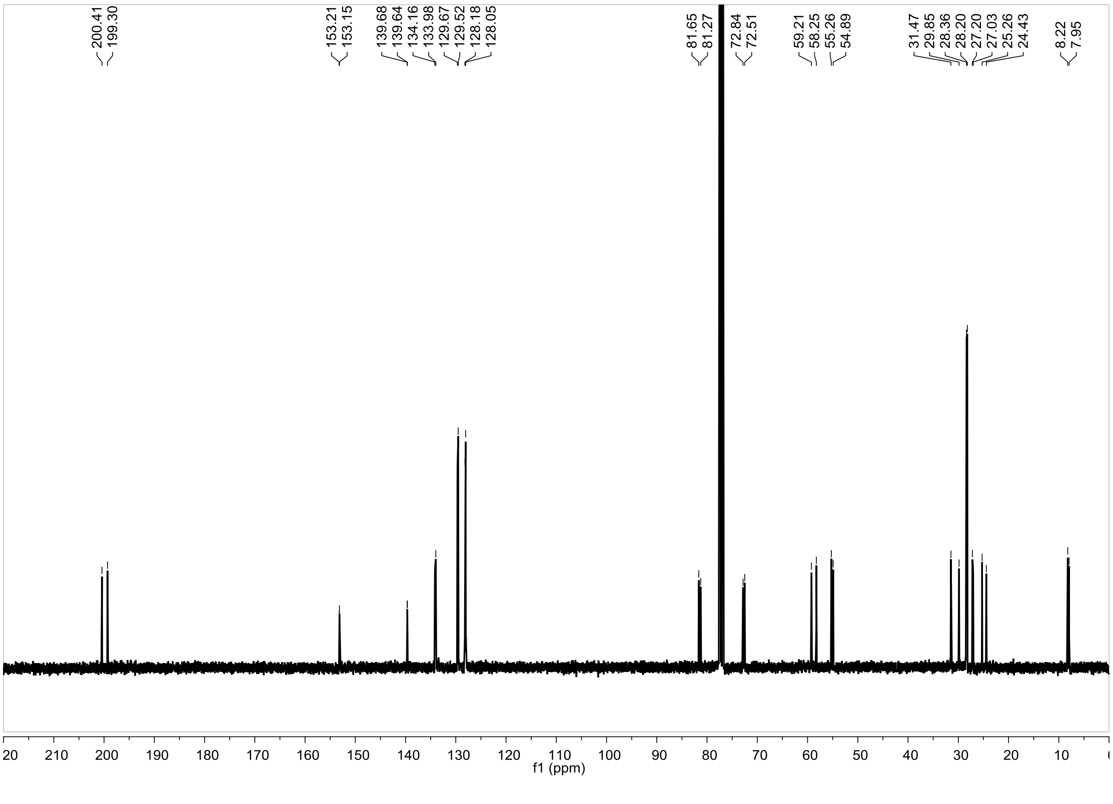
200.41
199.30
153.21
153.15
139.68
139.64
134.16
133.98
129.67
129.52
128.18
128.05
81.65
81.27
72.84
72.51
59.21
58.25
55.26
54.89
31.47
29.85
28.36
28.20
27.20
27.03
25.26
24.43
8.22
7.95
210 200 190 180 170 160 150 140 130 120 110 100 90 80 70 60 50 40 30 20 10
f1 (ppm)

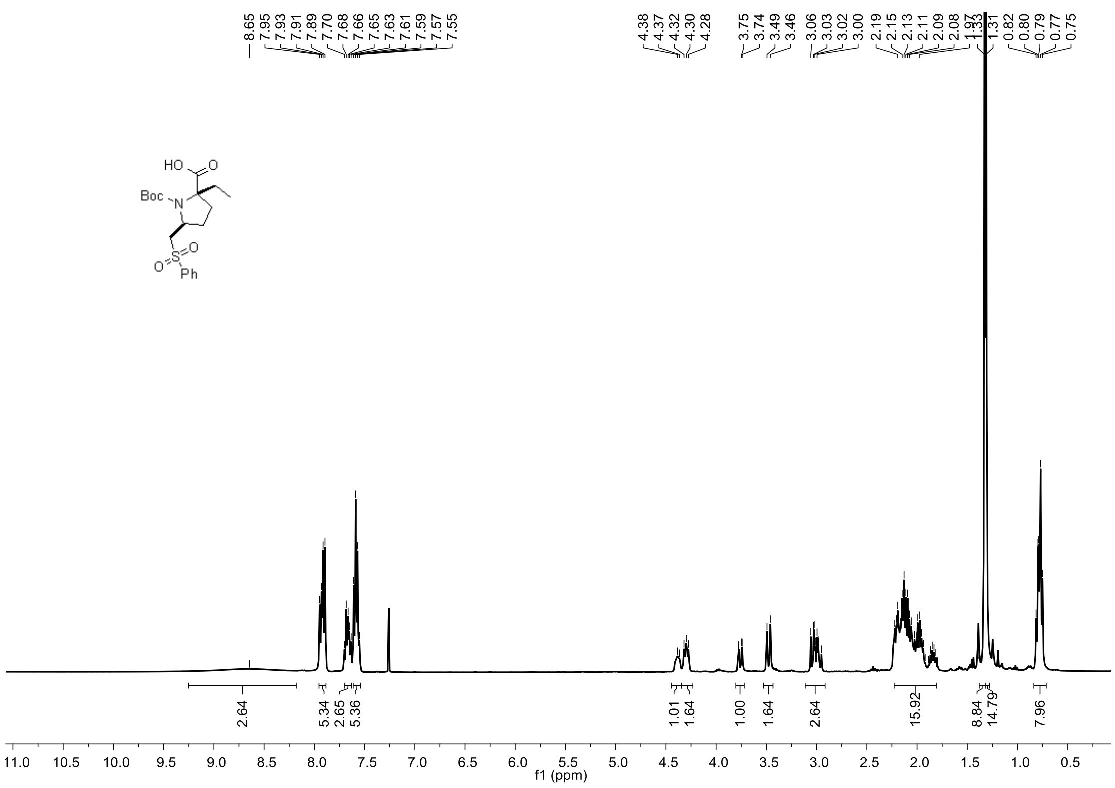
8.65
7.95
7.93
7.91
7.89
7.70
7.68
7.66
7.65
7.63
7.61
7.59
7.57
7.55
4.38
4.37
4.32
4.30
4.28
3.75
3.74
3.49
3.46
3.06
3.03
3.02
3.00
2.19
2.15
2.13
2.11
2.09
2.08
1.97
1.33
1.31
0.82
0.80
0.79
0.77
0.75
HO
O
Boc
N
O
S
O
Ph
2.64
5.34
2.65
5.36
1.01
1.64
1.00
1.64
2.64
15.92
8.84
14.79
7.96
f1 (ppm)

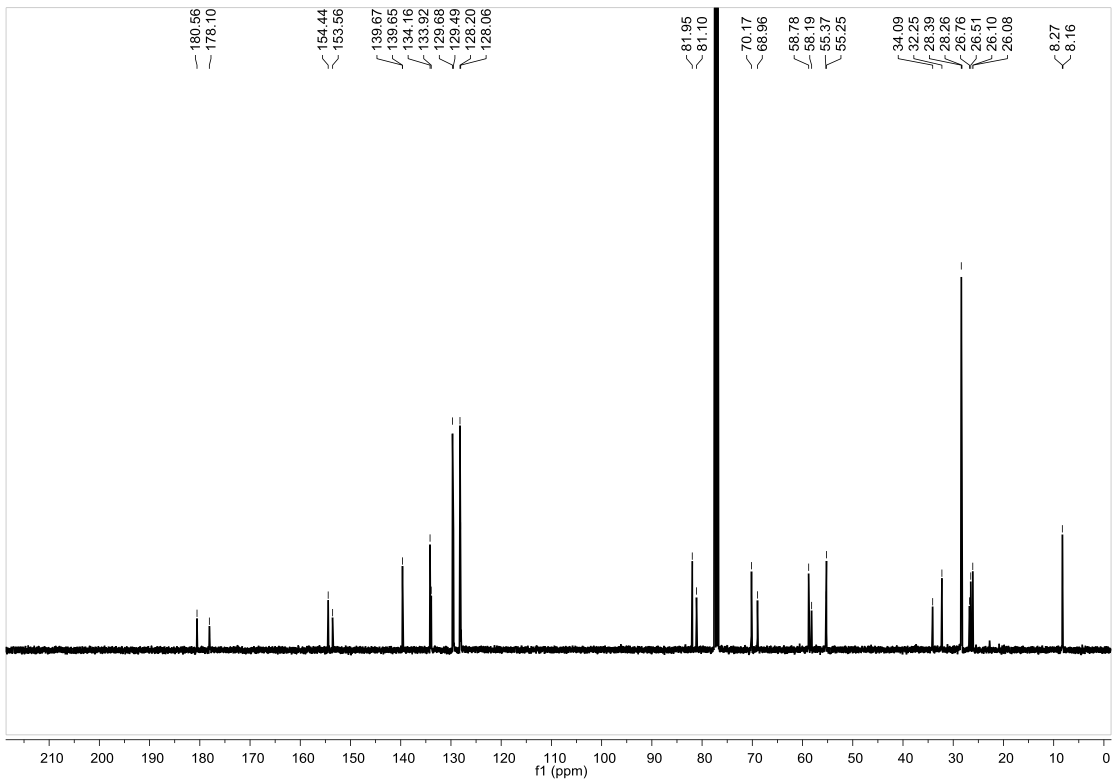
180.56
178.10
154.44
153.56
139.67
139.65
134.16
133.92
129.68
129.49
128.20
128.06
81.95
81.10
70.17
68.96
58.78
58.19
55.37
55.25
34.09
32.25
28.39
28.26
26.76
26.51
26.10
26.08
8.27
8.16
f1 (ppm)

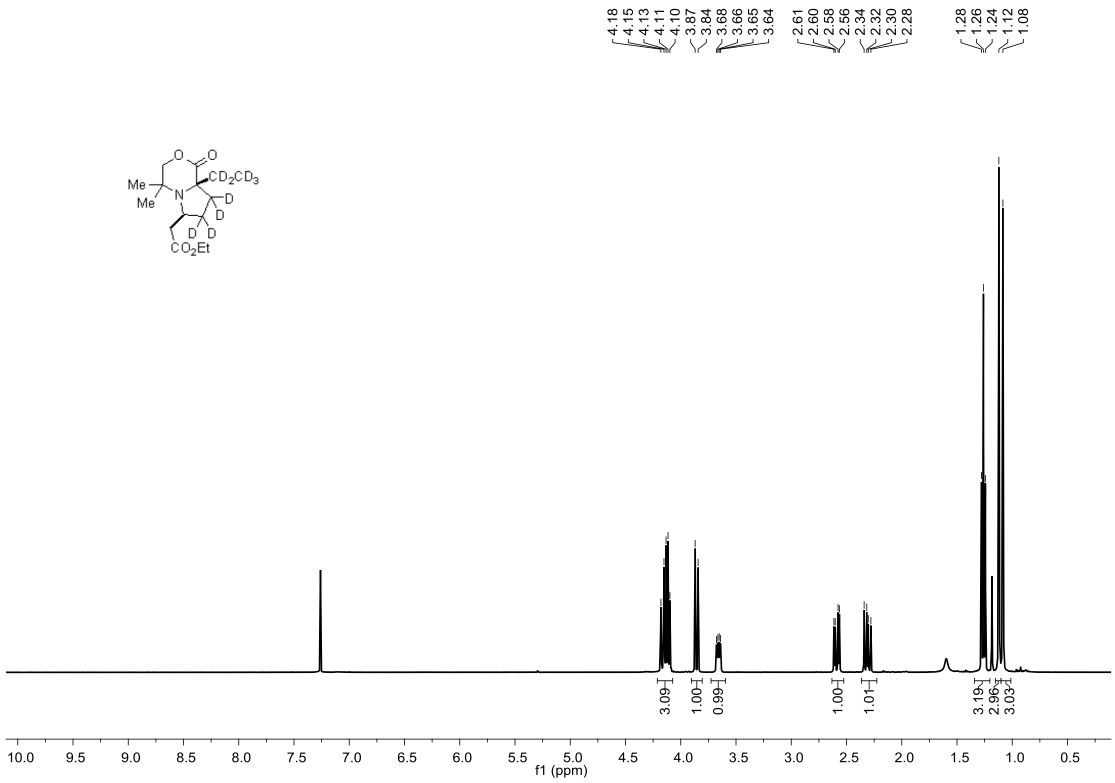

4.18
4.15
4.13
4.11
4.10
3.87
3.84
3.68
3.66
3.65
3.64
2.61
2.60
2.58
2.56
2.34
2.32
2.30
2.28
1.28
1.26
1.24
1.12
1.08
Me
Me
N
O
O
CD2CD3
D
D
D
D
CO2Et
3.09
1.00
0.99
1.00
1.01
3.19
2.96
3.03
10.0 9.5 9.0 8.5 8.0 7.5 7.0 6.5 6.0 5.5 5.0 4.5 4.0 3.5 3.0 2.5 2.0 1.5 1.0 0.5
f1 (ppm)

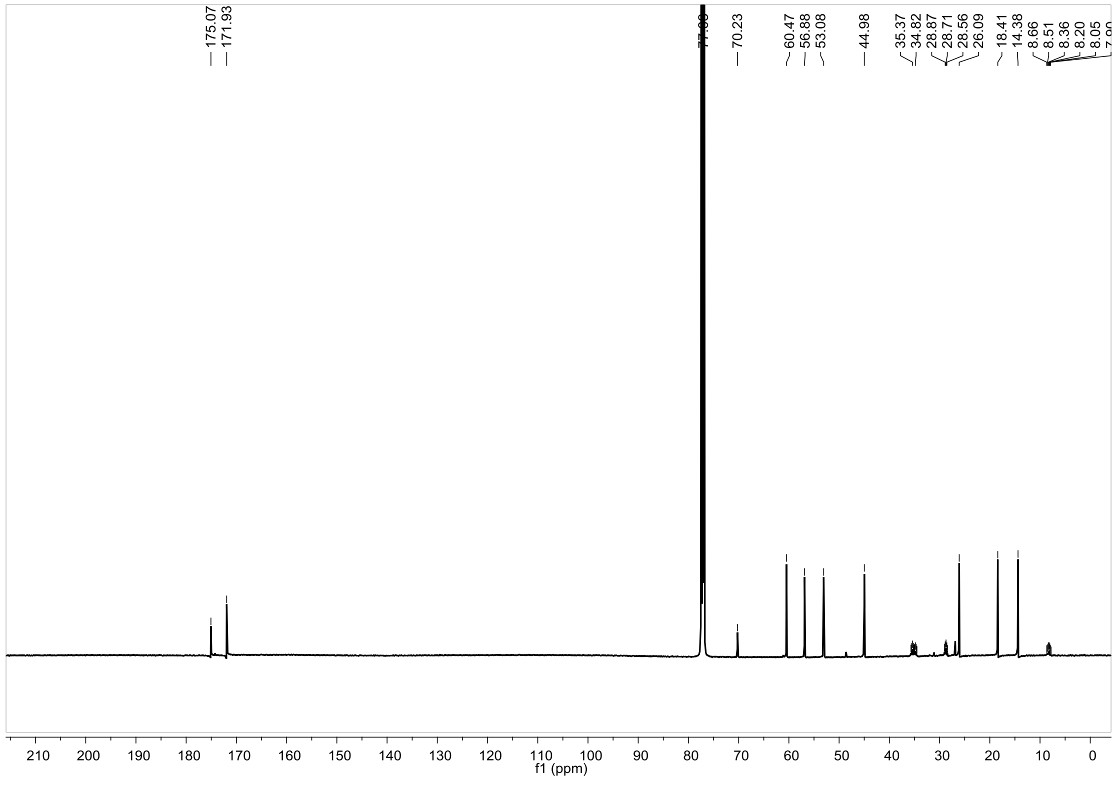

175.07
171.93
70.23
60.47
56.88
53.08
44.98
35.37
34.82
28.87
28.71
28.56
26.09
18.41
14.38
8.66
8.51
8.36
8.20
8.05
210 200 190 180 170 160 150 140 130 120 110 100 90 80 70 60 50 40 30 20 10 0
f1 (ppm)
